# Synthesis of Fungal Cell Wall Oligosaccharides and Their Ability to Trigger Plant Immune Responses

**DOI:** 10.1002/ejoc.202200313

**Published:** 2022-07-15

**Authors:** Manishkumar A. Chaube, Nino Trattnig, Du‐Hwa Lee, Youssef Belkhadir, Fabian Pfrengle

**Affiliations:** ^1^ Department of Biomolecular Systems Max Planck Institute of Colloids and Interfaces Am Mühlenberg 1 14476 Potsdam Germany; ^2^ Department of Chemistry University of Natural Resources and Life Sciences,Vienna Muthgasse 18 1190 Vienna Austria; ^3^ Gregor Mendel Institute (GMI) Austrian Academy of Sciences Vienna Biocenter (VBC) Dr Bohr Gasse 3 1030 Vienna Austria

**Keywords:** Automated glycan assembly, Carbohydrate chemistry, Fungal cell wall, Pattern-triggered immunity, Plant immunity

## Abstract

Oligosaccharide fragments of fungal cell wall glycans are important molecular probes for studying both the biology of fungi and fungal infections of humans, animals, and plants. The fungal cell wall contains large amounts of various polysaccharides that are ligands for pattern recognition receptors (PRRs), eliciting an immune response upon recognition. Towards the establishment of a glycan array platform for the identification of new ligands of plant PRRs, tri‐, penta‐, and heptasaccharide fragments of different cell wall polysaccharides were prepared. Chito‐ and β‐(1→6)‐gluco‐oligosaccharides were synthesized by automated glycan assembly (AGA), and α‐(1→3)‐ and α‐(1→4)‐gluco‐oligosaccharides were synthesized in solution using a recently reported highly α‐selective glycosylation methodology. Incubation of plants with the synthesized oligosaccharides revealed i) length dependence for plant activation by chito‐oligosaccharides and ii) β‐1,6‐glucan oligosaccharides as a new class of glycans capable of triggering plant activation.

## Graphical abstract

Automated glycan assembly and solution‐phase synthesis provided a set of fungal cell wall oligosaccharides that were investigated for their ability to activate the plant immune system. Synthetic chitin and β‐1,6‐glucan oligosaccharides triggered mitogen‐activated protein kinase phosphorylation and a reactive oxygen burst in Arabidopsis seedlings.

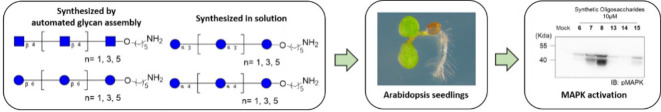

## Introduction

The cell wall of pathogenic fungi is the first cellular component to interact with the host immune system in infections of humans, animals, and plants. Plants sense and respond to pathogen attacks by detecting microbe‐associated molecular patterns (MAMPs) and danger‐associated molecular patterns (DAMPs) by pattern‐recognition‐receptors (PRRs).^[1]^ Despite the large number of PRRs in plants and the dominating presence of glycans in the cell walls of plants, bacteria, and fungi, only a handful of glycans were found to elicit plant immune responses through activation of pattern triggered immunity (PTI), and even fewer of the cognate receptors have been described.[^2^, ^3^] Typical glycan ligands for the PRRs include chitin,^[4]^ chitosan,^[5]^ and β‐glucans[^6^, ^7^] as microbial patterns (MAMPs) and oligogalacturonides,^[8]^ cello‐oligomers,[^9^, ^10^] xyloglucans,^[11]^ arabinoxylans,^[12]^ and mannans^[13]^ as DAMPS. These endogenous glycan structures are produced by enzymatic hydrolysis of plant cell wall polysaccharides. Efforts towards the identification of additional glycan ligands for plant PRRs must consider both plant and microbial glycans such as derived from fungi.

The fungal cell wall is mainly constructed from different classes of polysaccharides, all representing potential ligands for PRRs. One of the core saccharides of fungal cell walls is a β‐(1→4)‐linked *N*‐acetyl glucosamine (GlcNAc) oligomer, also known as chitin.^[14]^ It plays a key role in fungal morphogenesis during infection, but is also its blind spot, since smaller chitin fragments, resulting from plant or fungal enzymatic degradation, activate plant defense mechanisms already in the subnanomolar range.^[15]^ β‐Glucans are the most abundant types of glycans in fungal cell walls, mostly constructed from a backbone of β‐(1→3)‐linked glucans, which are branched with β‐(1→6)‐linked glucosides.^[16]^ There is evidence that β‐(1→3)‐glucans are pivotal for fungal cell wall maintenance and tensile‐strength, but less is known about the role of β‐(1→6)‐branches.^[14]^ Additionally, there are pathogenic fungi where β‐(1→6)‐glucans represent the major cell wall polymer, such as *Cryptococcus neoformans*, where they are crucial for cell integrity.^[17]^ β‐Glucosides are present in the fungal cell wall independently of their morphological stage. In contrast, α‐(1→3)‐glucosides in plant pathogenic fungi are mainly expressed during plant infection.^[14]^ These glycans form a shield that protects the fungus from plant innate immune responses, such as chitinases that consequently cannot reach their target.^[18]^ Other important glycans that can be found in some fungal species are glycogen‐like α‐(1→4) and (1→6)‐linked glucans, mannans, including phospho‐, rhamno‐, and galactomannans, and galactosaminogalactans.^[19]^

We have recently introduced a plant glycan microarray, which enabled us to determine the acceptor substrate specificities of plant glycosyltransferases and the epitopes of a large number of cell wall glycan‐directed antibodies.^[20]^ This array consists mostly of synthetic plant cell wall‐derived oligosaccharides and is constantly further developed.^[21]^ To procure fungal oligosaccharides for investigating plant‐activatory molecules and the corresponding receptors, we applied recently developed methodologies in automated glycan assembly (AGA)^[22]^ and 1,2‐*cis*‐selective glucan synthesis.^[23]^ In total, we synthesized twelve oligosaccharides including chito‐, β‐(1→6)‐gluco‐, α‐(1→3)‐gluco‐, and α‐(1→4)‐glucosides either by AGA or classical solution‐phase synthesis and report their abilities to trigger plant immune responses as assessed by hallmarks of activation such as mitogen‐activated protein kinases (MAPKs) phosphorylation and reactive oxygen species (ROS) bursts.

## Results and Discussion

### Synthesis of β‐(1→4)‐GlcNAc‐oligomers

**Scheme 1:**
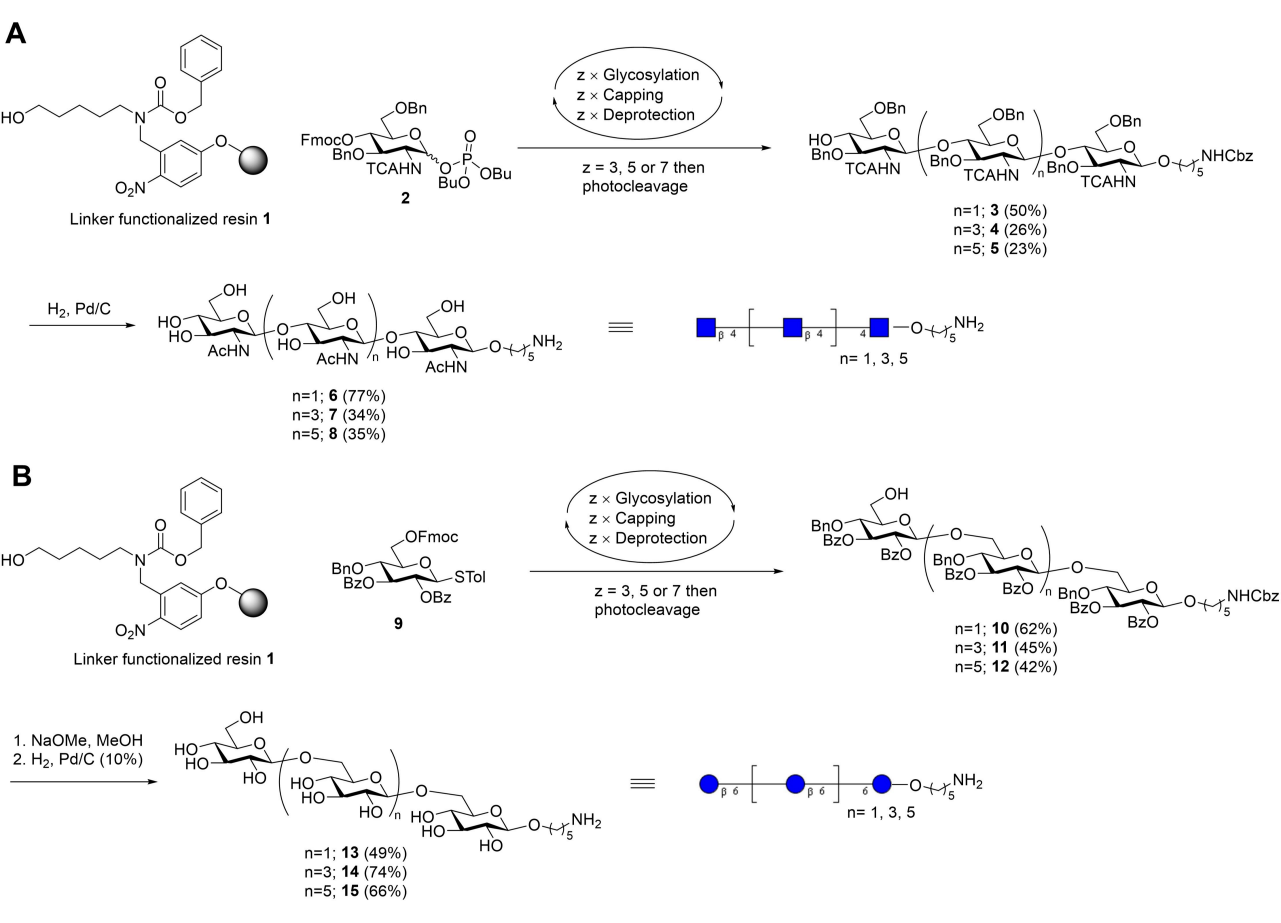
Automated glycan assembly of chito‐oligosaccharides (A) and β‐(1→6)‐glucooligosaccharides (B).

In order to obtain chitin fragments^[24]^ of different chain lengths, we applied AGA.^[25]^ In AGA, glycans are assembled stepwise on a solid support in a fully automated manner, enabling the preparation of well‐defined oligosaccharides in a short amount of time.[^22^, ^26^, ^27^] Linker‐functionalized resin **1**, providing a primary amine upon UV‐cleavage, was iteratively glycosylated with glycosyl phosphate donor **2** (Scheme 1A). Donor **2** is furnished with a base‐labile fluorenylmethoxycarbonyl (Fmoc) group that enables chain elongation after cleavage. The amine in the 2‐position is masked as a trichloroacetyl (TCA) group to ensure β‐selectivity and avoid side product formation during the glycosylation reactions.[^28^, ^29^] The remaining positions are permanently protected as benzyl ethers (Bn). Chain elongation was initiated by treatment of resin **1** with donor **2** and TMSOTf, followed by capping of unreacted acceptor with acetic anhydride (Ac_2_O). Then, cleavage of the Fmoc group with 20 % piperidine in DMF exposed the next hydroxyl group for glycosylation. This cycle of reactions was repeated 3–7 times to obtain tri‐, penta‐, and heptamers **3**, **4**, and **5** after UV‐induced cleavage of the linker and HPLC purification in 50 %, 26 % and 23 % yield, respectively. The fully protected saccharides were subjected to hydrogenolysis in the presence of 10 % Pd/C to obtain the final products **6**–**8** in 34–77 % yield.

### Synthesis of β‐(1→6)‐glucosides

Next, we utilized the AGA‐technology to produce a set of three β‐(1→6)‐linked oligoglucosides.^[30]^ In this case, linker‐equipped resin **1** was elongated using thioglycoside donor **9** (Scheme 1B).^[31]^ The donor is equipped with a Fmoc‐group at position 6 for temporary protection and a participating benzoyl ester (Bz) in position 2. The remaining positions are permanently protected with a Bz ester in position 3 and a Bn ether in position 4. Glycan assembly was performed by consecutive cycles of glycosylation, capping, and Fmoc deprotection, as described for synthesis of the chitin oligosaccharides. After cleavage from the resin and HPLC purification, tri‐, penta‐ and heptamers **10**, **11** and **12** were afforded in 62 %, 45 % and 42 % yield. Deprotected oligosaccharides **13**, **14** and **15** were obtained after Zemplen methanolysis and hydrogenolysis in 49–74 % yield.

### Synthesis of α‐(1→3)‐glucosides

**Scheme 2:**
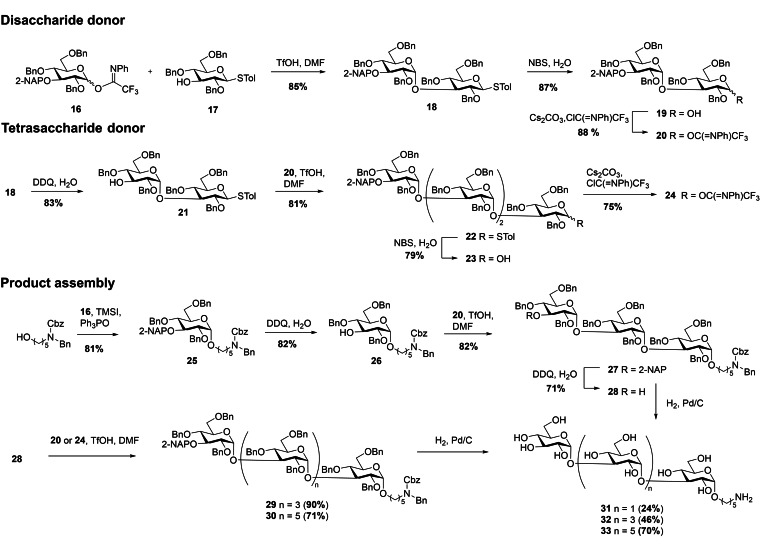
Assembly of α‐(1→3)‐gluco‐oligosaccharides.

α‐Glucosides are connected through 1,2‐*cis*‐glycosidic linkages, which makes their stereoselective synthesis more challenging compared to their 1,2‐*trans*‐linked cognates.^[32]^ A promising method for the synthesis of α‐linked glucans was reported by Codée and coworkers.[^23^, ^33^] Using DMF as an additive in the glycosylation reaction at low temperature provided high α‐selectivities and excellent yields. This is facilitated by the formation of an intermediary covalent glycosyl imidate.^[34]^ We utilized this methodology to produce three α‐(1→3)‐glucosides, with a chain length of 3, 5 and 7 monosaccharides.^[35]^ *N*‐Phenyltrifluoroacetimidate donor **16**,^[33]^ containing a temporary (2‐methyl)naphthyl (NAP) protecting group at position 3, was reacted with acceptor **17** ^[36]^ in the presence of 16 eq DMF and 1 eq TfOH at −78 °C (Scheme 2). After 45 min the donor had been fully transformed into the DMF‐imidate intermediate, which reacted further with the acceptor when warming the reaction mixture to 0 °C, affording disaccharide **18** with complete α‐selectivity and in an excellent yield of 85 %. The disaccharide was then transformed into *N*‐phenyltrifluoroacetimidate donor **20** by hydrolysis of the thioether with NBS/water and treatment of the resulting anomeric alcohol with Cs_2_CO_3_ and 2,2,2‐trifluoro‐*N*‐phenylacetimidoyl chloride (77 % yield over two steps). In addition to disaccharide donor **20**, tetrasaccharide donor **24** was prepared. Therefore, compound **18** was subjected to NAP deprotection using DDQ and was coupled with the previously produced disaccharide donor **20**, providing tetrasaccharide **22** with excellent α‐selectivity in a yield of 81 %. This fragment was then transformed into *N*‐phenyltrifluoroacetimidate donor **24** (60 % yield over two steps). With **16**, **20** and **24**, we had all donors for glycan assembly in hand. Donor **16** was coupled with a Cbz/benzyl protected 5‐amino pentanol linker,^[37]^ using TMSI and Ph_3_PO,^[23]^ which afforded compound **25** with an excellent α/β ratio of 10/1 in 81 % yield. Subsequently, the NAP group was removed and acceptor **26** was reacted with the previously prepared donor **20**, providing α‐linked trisaccharide **27** in 82 % yield. This fragment represented the first target compound and was subjected to global deprotection. To receive larger products, the NAP group in **27** was selectively removed to give glycosyl acceptor **28**. **28** was then glycosylated with either the previously prepared donor **20** or **24** to assemble penta‐ and heptasaccharides **29** and **30**. Strikingly, there was no drop in α‐selectivity or yield in the DMF‐mediated glycosylations, even when large donor molecules such as tetrasaccharide **24** were used. Consequently, excellent yields for the penta‐ (90 %) and heptasaccharides (71 %) were obtained. Eventually, **27**, **29**, and **30** were deprotected using hydrogen in the presence of 10 % Pd/C to afford the final products **31** (24 %), **32** (46 %) and **33** (70 %).

### Synthesis of α‐(1→4)‐glucosides

**Scheme 3:**
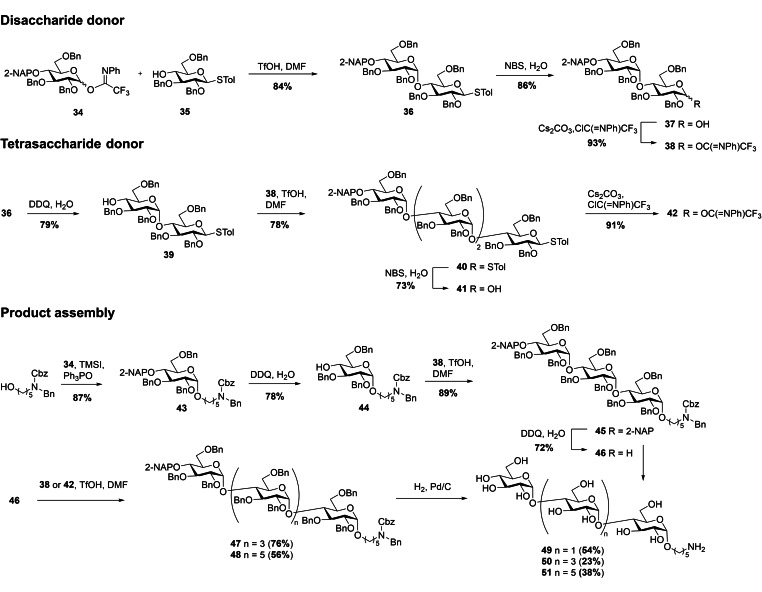
Assembly of α‐(1→4)‐gluco‐oligosaccharides.

The synthesis of tri‐, penta‐, and hepta‐α‐(1→4)‐linked glucans was performed following the same strategy as for the α‐(1→3)‐linked glucans.^[23]^ To that end, donor **34** was coupled with acceptor **35** followed by thioether hydrolysis and imidate formation to obtain donor **38** (Scheme 3). After NAP‐cleavage in compound **36** using DDQ and subsequent glycosylation with donor **38**, tetrasaccharide **40** was obtained in 78 % yield. **40** was then transformed into imidate donor **42** as described for **38**. Product assembly was initiated by installing the aminopentyl‐linker in **34** using TMSI and Ph_3_PO in 87 % yield (α/β=16 : 1). The resulting monosaccharide **43** was subjected to NAP‐cleavage and coupled with previously prepared disaccharide donor **38** to afford fully protected trisaccharide **45** with nearly complete α‐selectivity. **45** was either deprotected to produce α‐(1→4)‐triglucoside **49** or treated with DDQ to produce glycosyl acceptor **46** for the production of larger products. Glycosylation of **46** with either disaccharide donor **38** or tetrasaccharide donor **42** gave α‐configured products **47** and **48** in 76 % and 56 % yield, respectively. The latter donor was less reactive, resulting in slightly reduced yield. Final deprotections with H_2_ and 10 % Pd/C afforded the final products **49**, **50** and **51** with 23–54 % yield.

### Activation of pattern‐triggered immunity (PTI)

**Figure 1:**
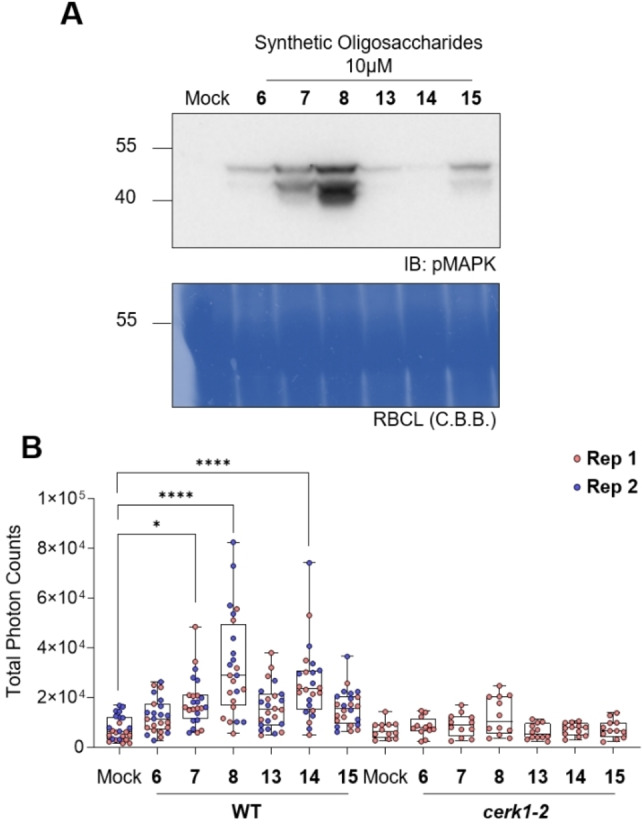
**Synthetic oligosaccharides induce immune responses in plants. A**. Synthetic oligosaccharides induced activation of MAPKs. The activated MPK3/6 proteins were detected by immunoblotting with an antibody detecting MAPK phosphorylation (anti‐pMAPK). Coomassie brilliant blue (C.B.B.) represents Rubisco large subunits to show equal loading of the samples. Similar band patterns were reproduced in a biologically independent experiment (see SI). **B**. Synthetic oligosaccharides induced ROS burst measured as total photon counts over 1 hour. 12 data points from two biologically independent experiments are displayed in WT. * indicates statistical significance (one‐way ANOVA followed by Tukey's multiple comparison test; **** p<0.001 and * p<0.05).

Next, we tested if our synthetic oligosaccharides can activate plant immune system outputs such as MAP kinases (MAPKs) phosphorylation and ROS bursts. Chito‐oligosaccharides are known to elicit an immune response mediated by the receptor‐like kinase CHITIN ELICITOR RECEPTOR KINASE 1 (CERK1).^[38]^ As a hallmark of immune activation, we first determined MAPKs activation by treating Arabidopsis seedlings with our chito‐oligosaccharides **6**–**8** (Figure 1). Notably, we observed that MAPK phosphorylation increased concomitantly with an increase in chito‐oligosaccharides chain length. In addition to chito‐oligosaccharides, treatments with β‐1,6‐linked heptaglucoside **15** were also able to induce MAPK activation. Next, we used reactive oxygen species (ROS) bursts to confirm MAPK activation patterns. Chito‐pentasaccharide **7** and more strongly chito‐heptasaccharide **8** triggered a ROS‐burst when applied at 10 μM. Additionally, β‐1,6‐linked pentaglucoside **14** induced robust ROS production. As opposed to MAPK activation, the shorter pentasaccharide **14** reproducibly induced a stronger ROS burst than the respective heptasaccharide **15**. The reasons for the different effects of **14** and **15** on MAPK activation and ROS production remain unclear and further investigations into determining 1,6‐β‐glucan binding receptors and the associated downstream signaling pathways are required. As oxidative burst and MAPK activation are regarded as two independent signaling events, non‐concordant downstream events are possible in specific ligand‐receptor pairs. We were not able to detect any immunogenic activities in the *cerk1‐2* mutants, indicating thus that not only the responses elicited by chito‐, but also β‐1,6‐gluco‐oligosaccharides are CERK1‐dependent. Unlike β‐1,3‐,^[6]^ β‐1,4‐,^[10]^ and mixed‐linkage glucans,[^39^, ^40^] β‐1,6‐glucans have not yet been reported as plant immune response elicitors. Together, our results indicate that these oligosaccharides are immunogenic and can as such be considered as a new class of potential MAMPs. The synthesized α‐glucan oligosaccharides did not elicit immune responses.

## Conclusion

We have performed the syntheses of twelve oligosaccharides that represent potential MAMPS/DAMPS in fungal infections of plants. We showed that AGA is well suited for the construction of β‐(1→4)‐GlcNAc and β‐(1→6)‐Glc oligomers, and we synthesized oligosaccharides with 3, 5 and 7 repeating units. Additionally, α‐(1→3)‐ and α‐(1→4)‐Glc oligomers were synthesized by DMF‐mediated glycosylation reactions. We observed that not only monosaccharide, but also larger donors (tetrasaccharide donors) are amenable for that methodology and provide excellent α‐selectivities in the glycosylation reactions. The differently sized synthetic glycans are powerful molecular tools to reveal which PRRs are capable of recognizing these molecular structures, also disclosing the minimum chain length necessary for binding. We have tested the ability of the synthetic oligosaccharides to activate immune defense responses in Arabidopsis seedlings and found not only the expected activity for chito‐oligosacccharides **6**–**8**, but also for β‐1,6‐linked penta‐ and heptaglucosides **14** and **15**. The linker‐equipped products will be included in our glycan microarray printings for future screenings, aiming at identifying plant receptor‐like kinases that can recognize the synthesized oligosaccharides, and thus act as potential immune receptors.

## Experimental Section

### Materials and Methods

The automated syntheses were performed on a self‐built synthesizer developed in the Max Planck Institute of Colloids and Interfaces. Linker‐functionalized resin **1** was prepared and resin loading was determined as previously reported.^[41]^ Solvents and reagents were used as supplied without further purification. Anhydrous solvents were taken from a dry solvent system (JC‐Meyer Solvent Systems). Column chromatography was carried out using Fluka silica gel 60 (230–400 mesh). NMR spectra were recorded on a Bruker Avance III 600 or a Bruker 400 instrument spectrometer using solutions of the respective compound in CDCl_3,_ CD_2_Cl_2_ or D_2_O. NMR chemical shifts (δ) are reported in ppm and coupling constants (*j*) in Hz. ^1^H spectra were referenced to 7.26 (CDCl_3_), 5.30 (CD_2_Cl_2_) and 0.00 (D_2_O, external calibration to 2,2‐dimethyl‐2‐silapentane‐5‐sulfonic acid) ppm. ^13^C spectra were referenced to 77.00 (CDCl_3_), 53.52 (CD_2_Cl_2_) and 67.40 (D_2_O, external calibration to 1,4‐dioxane) ppm. High resolution mass spectra were obtained on a 6210 ESI‐TOF mass spectrometer (Agilent) or a Micromass Q‐TOF Ultima Global instrument. Analytical HPLC was performed on an Agilent 1200 series coupled to a quadrupole ESI LC/MS 6130 using a YMC‐Diol‐300 column (150×4.6 mm), a Phenomenex Luna C5 column (250×4.6 mm), or a Thermo Scientific Hypercarb column (150×4.6 mm). Preparative HPLC was performed on an Agilent 1200 series using a preparative YMC‐Diol‐ 300 column (150×20 mm), a semi‐preparative Phenomenex Luna C5 column (250×10 mm), a semi‐preparative SIC‐HILIC (150×20 mm) or a semi‐preparative Thermo Scientific Hypercarb column (150×10 mm). For filtration syringe filters (RC, 0.45 μm) from Roth were used.

### Synthesizer modules and conditions

Linker‐functionalized resin **1** (12.5 μmol of hydroxyl groups) was placed in the reaction vessel of the automated oligosaccharide synthesizer and swollen for at least 30 min in DCM. Before every reaction step the resin was washed with DMF, THF and DCM. Subsequently, the glycosylation (Module A), Capping (Module B) and deprotection (Module C) steps were performed. Mixing of the components was accomplished by bubbling Argon through the reaction mixture.

### Module A1: Glycosylation with glycosyl phosphates

The resin **1** (12.5 μmol of hydroxyl groups) was swollen in DCM (2 mL) and the temperature of the reaction vessel was adjusted to −30 °C. Prior to the glycosylation reaction the resin was washed with 62 mM TMSOTf in DCM and then DCM only. For the glycosylation reaction the DCM was drained and a solution of phosphate BB (6.5 eq, 60.0 mM DCM) was delivered to the reaction vessel at −30 °C. The reaction was initiated by the addition of 62 mM TMSOTf in DCM (1 mL). The glycosylation was performed for 5 min at −30 °C and then for 40 minutes at −10 °C. Subsequently, the solution was drained and the resin washed three times with DCM at 25 °C.

### Module A2: Glycosylation with glycosyl thioglycosides

The resin **1** (12.5 μmol of hydroxyl groups) was swollen in DCM (2 mL) and the temperature of the reaction vessel was adjusted to −20 °C. Prior to the glycosylation reaction the resin was washed with 62 mM TMSOTf in DCM and then DCM only. For the glycosylation reaction the DCM was drained and a solution of thioglycoside BB (1 mL; 80.0 mM in DCM) was delivered to the reaction vessel. After the set temperature was reached, the reaction was started by the dropwise addition of the activator solution (1 mL; 0.15 M NIS/15 mM TfOH in DCM/dioxane=2/1). The glycosylation was performed for 5 min at −20 °C and then for 30 min at 0 °C. Subsequently, the solution was drained and the resin was washed with DCM (2 mL), DCM:dioxane (1 : 2, 3 mL for 20 s) and DCM (two times, each with 2 mL for 25 s). The temperature of the reaction vessel was increased to 25 °C for the next module.

### Module B: Capping

The temperature of the reaction vessel was adjusted to 25 °C. 10 % pyridine in dry DMF (2 mL) was delivered. After 1 min, the reaction solution was drained and the resin washed with DCM (three times with 3 mL for 25 s). Then a solution of 10 % acetic anhydride and 2 % methanesulfonic acid in DCM (4 mL) was delivered to the reaction vessel. After 20 min, the solution was drained and the resin washed with DCM (three times with 3 mL for 25 s).

### Module C: Fmoc deprotection

The resin was washed with DMF, swollen in 2 mL DMF and the temperature of the reaction vessel was adjusted to 25 °C. Prior to the deprotection reaction DMF was drained and the resin was washed with DMF three times. For Fmoc deprotection 2 mL of a solution of 20 % piperidine in DMF was delivered to the reaction vessel. After 5 min, the reaction solution was drained and the resin washed with DMF (three times with 3 mL for 25 s) and DCM (five times each with 2 mL for 25 s). The temperature of the reaction vessel was then decreased to −20 °C for the next step

### Cleavage from the solid support

After assembly of the oligosaccharides, cleavage from the solid support was accomplished by UV irradiation at 305 nm in a continuous flow photoreactor as previously described.^[41]^

### Global deprotection A

The purified glycan obtained after resin cleavage was dissolved in a mixture of DCM/^*t*^BuOH/H_2_O (2 : 1 : 1, 2 mL) and 300 w % Pd−C (10 %) was added. Then, the reaction was stirred in a H_2_ bomb with 60 psi pressure for the indicated time. The reaction solution was filtered through Celite, washed with DCM, ^*t*^BuOH and H_2_O followed by CH_3_CN/H_2_O (1 : 1). The filtrates were concentrated *in vacuo* and the crude product was purified by reversed phase HPLC (Synergy column) to obtain the fully deprotected product

### Global deprotection B

The purified glycan obtained after resin cleavage was dissolved in a mixture of anhydrous DCM/methanol (1.5 mL, 1 : 1) and NaOMe (0.5 M solution in MeOH; 3 eq/OBz) was added to the reaction solution and it was stirred until completion. Then, Amberlite IR‐120 (acidic form) was added until pH=7. The solution was filtered using methanol and DCM and concentrated under reduced pressure. The crude product was dissolved in a mixture of DCM/^*t*^BuOH/H_2_O (2 : 1 : 1, 2 mL) followed by the addition of 300 w % Pd−C (10 %). Then, the reaction was stirred in a H_2_ bomb with 60 psi pressure for 48 h. The reaction solution was filtered through Celite, washed with DCM, ^*t*^BuOH and H_2_O followed by CH_3_CN/H_2_O (1 : 1). The filtrates were concentrated *in vacuo* and the crude product was purified by reversed phase HPLC (Synergy column) to obtain the fully deprotected product.

**MAPK assays**: Sixteen 5‐day old wild‐type (Col‐0) *Arabidopsis* seedlings grown on 1/2 MS plates containing 0.8 % plant agar and 1 % sucrose, were transferred to a 6‐well plate (Griner Bio one, Cat. No. 657185) containing 2 mL of liquid 1/2 MS medium and incubate further 1 weeks. For treatment of oligosaccharides, the previous liquid medium was replaced by new liquid 1/2 MS medium containing each oligosaccharide (10 μM) and incubate for 15 minutes. After treatment, the seedlings were immediately transferred to 2 mL tubes containing glass beads and homogenized with TissueLyser II (Qiagen) in frozen with liquid N_2_. Tissues were further homogenized in protein extraction buffer (50 mM TRIS pH 7.5, 100 mM NaCl, 10 % glycerol, 5 mM EDTA, 1 mM Na_2_MoO_4_, 20 mM NaF, 1 mM DTT, EDTA‐free protease inhibitor cocktail (Roche, Cat. No. 45–5056489001). After centrifugation (10 min, 20,000 *g*, 4 °C), the supernatant was boiled for 2 min, 95 °C with Laemmli sample buffer, and then the samples were subjected to SDS‐PAGE. Immunoblotting was performed using an anti‐p44/p42 MAPK (anti‐ ERK1/2) antibodies (Cell Signalling, Antibody #9102) with anti‐Rabbit‐HRP antibodies (Sigma‐Aldrich, Cat. No. A6154).

**ROS burst assays**: ROS burst assays were performed as previously described with minor modifications.^[42]^ Briefly, leaf discs (diameter 4 mm) were punched out from 6‐week‐old healthy wild‐type (Col‐0) or *cerk1‐2* plants. The discs were placed in a 96‐well luminescence assay plate (Griner Bio one, Cat. No. 675 074) containing 200 μL sterile MonoQ H_2_O, with the adaxial side up. Discs were vacuum infiltrated for 10 min and incubated for 12 h in darkness at 21 °C. The water was carefully removed and replaced with 100 μL of each oligosaccharide (10 μM) eliciting solution with 0.02 mg/mL Horse Radish Peroxidase (Sigma‐Aldrich) and 0.034 mg/mL luminol (Wako Chemicals). The plate was immediately measured luminescence using a BiTec Synergy 4 microplate reader. Total light units of 60 time points during 1 hour were integrated for the analysis.

## Conflict of interest

The authors declare no conflict of interest.

1

## Supporting information

As a service to our authors and readers, this journal provides supporting information supplied by the authors. Such materials are peer reviewed and may be re‐organized for online delivery, but are not copy‐edited or typeset. Technical support issues arising from supporting information (other than missing files) should be addressed to the authors.

Supporting Information

## Data Availability

The data that support the findings of this study are available from the corresponding author upon reasonable request.
