# Supplementary material for: Synthesis of Fungal Cell Wall Oligosaccharides and Their Ability to Trigger Plant Immune Responses
Source: European J Org Chem. 2022 Jul 15;2022(27):e202200313. doi: 10.1002/ejoc.202200313 (PMC9401017; doi:10.1002/ejoc.202200313)
Supplement: Supplementary file 1 — Supporting Information [file EJOC-2022-0-s001.pdf]

European Journal of Organic Chemistry

Supporting Information

Synthesis of Fungal Cell Wall Oligosaccharides and Their Ability to Trigger Plant Immune Responses

Manishkumar A. Chaube[+], Nino Trattnig[+], Du-Hwa Lee, Youssef Belkhadir, and Fabian Pfrengle*

Analytical data

Contents

1.1 Synthetic methods 2
1.2 Analytical data of Chitin series 21
1.3 Analytical data of β-[1→6]-series 33
1.4 Analytical data of α-[1→3]-series 44
1.4 Analytical data of α-[1→4]-series 63
1.5 Biological replicate of MAPK phosphorylation assay 82
1.6 References 83

1.1 Synthetic methods

Dibutoxyphosphoryloxy 2-deoxy (trichloroacetyl carbonylamino)-3,6-di-*O*-benzyl-4-*O*-fluorenylcarboxymethyl-α/β-D-glucopyranoside (2): To a suspension of ethyl 2-deoxy (trichloroacetyl carbonylamino)-3,6-di-*O*-benzyl-4-*O*-fluorenylcarboxymethyl-1-thio-α/β-D-glucopyranoside[1] (2.00 g, 2.59 mmol) and powdered molecular sieves (1.00 g) in anhydrous DCM (30 mL) was added dibutyl phosphate (0.56 mL, 2.84 mmol) and the mixture was stirred for 20 minutes. Then the reaction mixture was cooled to -40 °C, NIS (0.81 g, 3.62 mmol) and TfOH (46.0 µL, 0.52 mmol) were added and the reaction solution was allowed to warm to -20 °C. After stirring for 30 minutes the reaction was quenched by the addition of NEt_3 (150 µL) and filtered through a bed of Celite. The organic layer was washed with saturated aqueous $Na_2S_2O_3$-solution (20 mL), dried over $MgSO_4$, filtered and the solvent was evaporated. The residue was subjected to silica gel column chromatography (Hex:EtOAc = 2:1) to give glycosyl phosphate **2** (1.97 g, 2.14 mmol, 83% yield, α/β = 1/3.6) as a light yellowish oil. R_f: 0.27 (Hex:EtOAc = 7:3); ^{1}H NMR (400 MHz, $CDCl_3$, α/β): δ 8.16-8.05, 7.88-7.76, 7.67-7.19, 7.01-6.94 (4 m, 18H), 5.81 (dd, *J* = 6.2, 3.2 Hz, 0.23H), 5.61 (t, *J* = 7.6 Hz, 0.77H), 5.17 (t, *J* = 9.6 Hz, 0.23H), 5.07 (t, *J* = 8.9 Hz, 0.79H), 4.74-4.43 (m, 2H), 4.61-4.48 (m, 2H), 4.43-4.32 (m, 2H), 4.27-4.49 (m, 7H), 3.91-3.83 (m, 1H), 3.72-3.62 (m, 2H), 1.72-1.63 (m, 2H), 1.47-1.37 (m, 2H), 1.02-1.92 (m, 3H) ppm ; ^{13}C NMR (100 MHz, $CDCl_3$): δ 162.4 162.0, 154.3, 154.1, 143.3, 143.2, 143.18, 143.14, 141.4, 141.3, 137.7, 137.6, 137.4, 137.2, 128.6, 128.48, 128.45, 128.2, 128.09, 128.06, 128.02, 127.9, 127.87, 127.82, 127.6, 127.3, 127.2, 125.17, 125.12, 125.10, 125.0, 120.2, 96.5, 95.5, 78.3, 75.9, 74.7, 74.1, 73.9, 73.6, 70.1, 69.0, 68.4, 60.1, 46.5, 32.2, 18.7, 13.6 ppm; ESI-HRMS: m/z $[M+Na]^+$ calc. for $C_{45}H_{51}Cl_3NNaO_{11}P$: 940.2158; found 940.2194.

Benzyloxycarbonylaminopentyl 3,6-di-*O*-benzyl-2-deoxy-2-*N*-2,2,2-trichloroacetamido-β-D-glucopyranosyl-(1→4)-3,6-di-*O*-benzyl-2-deoxy-2-*N*-2,2,2-trichloroacetamido-β-D-glucopyranosyl-(1→4)-3,6-di-*O*-benzyl-2-deoxy-2-*N*-2,2,2-trichloroacetamido-β-D-glucopyranoside (3): The synthesizer modules were applied as follows: A(**2**, 3 cycles)-B-C-A(**2**, 3 cycles)-B-C-A(**2**, 3 cycles)-B-C followed by resin cleavage. The crude product was purified by HPLC (YMC-diol column, Hex/EtOAc = 100/0 → 0/100) to obtain the trisaccharide **3** (10.7 mg, 6.30 µmol, 50% over 6 steps). ^{1}H NMR (400 MHz, $CDCl_3$): δ 7.28-7.11 (m, 35H), 6.86 (d, *J* = 7.8 Hz, 1H, NH), 6.22 (d, *J* = 8.6 Hz, 1H, NH), 6.18 (d, *J* = 8.7 Hz, 1H, NH), 5.00 (s, 2H), 4.90 (d, *J* = 11.4 Hz, 1H), 4.76 (t, *J* = 11.4 Hz, 2H,), 4.68 (d, *J* = 8.0 Hz, 1H), 4.64 (t, *J* = 11.5 Hz, 2H), 4.50 (t, *J* = 11.5 Hz, 1H), 4.45 (d, *J* = 11.0 Hz, 1H), 4.44 (d, *J* = 11.8 Hz, 1H), 4.39 (d, *J* = 8.3 Hz, 1H), 4.34 (d, *J* = 6.6 Hz, 1H), 4.31 (s, 2H), 4.13 (d, *J* = 11.9 Hz, 1H), 3.94 (dd, *J* = 9.3, 8.9 Hz, 2H), 3.86 (t, *J* = 8.5 Hz, 1H), 3.78-3.76 (m, 1H), 3.67-3.58 (m, 5H), 3.54-3.45 (m, 3H), 3.41-3.31 (m, 3H), 3.29-3.22 (m, 3H), 3.18-3.04 (m, 5H), 1.51-1.44 (m, 2H), 1.42-1.35 (m, 2H), 1.31-1.23 (m, 2H) ppm; ^{13}C NMR (100 MHz, $CDCl_3$): δ 161.8, 161.7, 156.5, 138.6, 138.4, 138.1, 137.8, 137.6, 137.3, 136.7, 129.0, 128.8, 128.75, 128.71, 128.6, 128.49, 128.42, 128.27, 128.22, 128.16, 128.10, 128.07, 127.89, 127.87, 127.6, 127.5, 99.7, 99.3, 99.0, 92.6, 92.5, 80.0, 78.6, 78.1, 77.4, 77.3, 77.2, 77.1, 76.8, 76.0, 75.7, 75.2, 74.6, 74.59, 74.51, 74.4, 73.9, 73.7, 73.5, 72.5, 71.4, 69.7, 68.2, 66.7, 57.6, 57.4, 57.3, 41.0, 29.8, 29.7, 29.0, 23.3 ppm; MALDI-TOF m/z $[M+Na]^+$ calc. for $C_{79}H_{85}Cl_9NaN_4O_{18}$: 1715.295; found 1716.873.

Benzyloxycarbonylaminopentyl 3,6-di-*O*-benzyl-2-deoxy-2-*N*-2,2,2-trichloroacetamido-β-D-glucopyranosyl-(1→4)-3,6-di-*O*-benzyl-2-deoxy-2-*N*-2,2,2-trichloroacetamido-β-D-glucopyranosyl-(1→4)-3,6-di-*O*-benzyl-2-deoxy-2-*N*-2,2,2-trichloroacetamido-β-D-glucopyranosyl-(1→4)-3,6-di-*O*-benzyl-2-deoxy-2-*N*-2,2,2-trichloroacetamido-β-D-glucopyranosyl-(1→4)-3,6-di-*O*-benzyl-2-deoxy-2-*N*-2,2,2-trichloroacetamido-β-D-glucopyranoside (4) The synthesizer modules were applied as follows: A(**2**, 3 cycles)-B-C-A(**2**, 3

cycles)-B-C-A(**2**, 3 cycles)-B-C-A(**2**, 3 cycles)-B-C-A(**2**, 3 cycles)-B-C followed by resin cleavage. The crude product was purified by HPLC (YMC-diol column, Hex/EtOAc = 100/0 → 0/100) to obtain the pentasaccharide **4** (8.70 mg, 3.26 µmol, 26% over 10 steps). ^{1}H NMR (400 MHz, $CDCl_3$): δ 7.29-7.11 (m, 55H), 6.85 (d, *J* = 7.7 Hz, 1H, NH), 6.12 (d, *J* = 8.9 Hz, 2H, NH), 6.05 (d, *J* = 8.7 Hz, 1H, NH), 5.96 (d, *J* = 8.7 Hz, 1H, NH) 5.00-4.89 (m, 6H), 4.75 (t, *J* = 12.0 Hz, 3H), 4.71 (d, *J* = 6.4 Hz, 1H), 4.67 (d, *J* = 7.4 Hz, 1H), 4.62 (t, *J* = 11.4 Hz, 3H), 4.51 (d, *J* = 11.4 Hz, 1H), 4.48 (d, *J* = 8.8 Hz, 1H), 4.43 (d, *J* = 11.2 Hz, 4H), 4.39 (d, *J* = 11.4 Hz, 1H), 4.35 (d, *J* = 7.6 Hz, 1H), 4.32 (d, *J* = 10.9 Hz, 2H), 4.29 (d, *J* = 12.1 Hz, 4H), 4.24 (d, *J* = 8.1 Hz, 1H), 4.15 (t, *J* = 7.9 Hz, 2H), 4.06-3.99 (m, 4H), 3.90 (t, *J* = 9.1 Hz, 5H), 3.78-3.57 (m, 9H), 3.54-3.33 (m, 11H), 3.27-3.14 (m, 4H), 3.09-2.95 (m, 8H), 1.49-1.45 (m, 2H), 1.43-1.36 (m, 2H), 1.28-1.25 (m, 2H) ppm; ^{13}C NMR (100 MHz, $CDCl_3$): δ 161.73, 161.71, 161.67, 161.64, 156.3, 138.64, 138.61, 138.5, 138.3, 138.0, 137.6, 137.5, 137.3, 137.2, 136.6, 129.07, 129.05, 128.9, 128.87, 128.83, 128.77, 128.71, 128.59, 128.54, 128.4, 128.37, 128.35, 128.31, 128.2, 128.16, 128.11, 128.04, 128.02, 127.9, 127.78, 127.76, 127.6, 127.4, 127.3, 99.6, 99.3, 99.0, 92.67, 92.60, 92.5, 92.4, 80.0, 79.1, 78.9, 78.7, 78.0, 77.3, 77.2, 77.0, 76.7, 76.0, 75.9, 75.7, 75.0, 74.5, 74.3, 74.2, 74.0, 73.8, 73.6, 73.5, 73.4, 72.4, 71.3, 69.6, 68.0, 67.8, 66.6, 57.4, 57.1, 57.0, 56.9, 40.9, 29.7, 29.5, 28.9, 23.2 ppm; MALDI-TOF m/z $[M+Na]^+$ calc. for $C_{167}H_{173}Cl_{21}NaN_8O_{38}$: 2692.401; found 2692.311.

Benzyloxycarbonylaminopentyl 3,6-di-*O*-benzyl-2-deoxy-2-*N*-2,2,2-trichloroacetamido-β-D-glucopyranosyl-(1→4)-3,6-di-*O*-benzyl-2-deoxy-2-*N*-2,2,2-trichloroacetamido-β-D-glucopyranosyl-(1→4)-3,6-di-*O*-benzyl-2-deoxy-2-*N*-2,2,2-trichloroacetamido-β-D-glucopyranosyl-(1→4)-3,6-di-*O*-benzyl-2-deoxy-2-*N*-2,2,2-trichloroacetamido-β-D-glucopyranosyl-(1→4)-3,6-di-*O*-benzyl-2-deoxy-2-*N*-2,2,2-trichloroacetamido-β-D-glucopyranosyl-(1→4)-3,6-di-*O*-benzyl-2-deoxy-2-*N*-2,2,2-trichloroacetamido-β-D-glucopyranosyl-(1→4)-3,6-di-*O*-benzyl-2-deoxy-2-*N*-2,2,2-trichloroacetamido-β-D-glucopyranoside (5): The synthesizer modules were applied as follows: A(**2**, 3 cycles)-B-C-A(**2**, 3 cycles)-B-C-A(**2**, 3 cycles)-B-C-A(**2**, 3 cycles)-B-C-A(**2**, 3 cycles)-B-C-A(**2**, 3 cycles)-B-C-A(**2**, 3 cycles)-B-C followed by resin cleavage. The crude product was purified by HPLC (YMC-diol column, Hex/EtOAc = 100/0 → 0/100) to obtain the heptasaccharide **5** (10.9 mg, 2.99 µmol, 23% over 14 steps). ^{1}H NMR (400 MHz, $CDCl_3$): δ 7.36-7.26 (m, 44H), 7.25-7.14 (m, 31H), 6.91 (d, *J* = 8.4 Hz, 1H, NH), 6.19 (d, *J* = 8.4 Hz, 1H, NH), 6.16 (d, *J* = 8.4 Hz, 1H, NH),6.07 (d, *J* = 8.9 Hz, 1H, NH), 6.00 (d, *J* = 9.1 Hz, 2H, NH), 5.97 (d, *J* = 8.7 Hz, 1H, NH), 5.07-4.97 (m, 7H), 4.81 (t, *J* = 11.8 Hz, 2H), 4.76 (d, *J* = 6.9 Hz, 1H), 4.70 (t, *J* = 11.1 Hz, 3H), 4.58-4.46 (m, 7H), 4.43-4.33 (m, 7H), 4.42 (d, *J* = 6.7 Hz, 1H), 4.30 (d, *J* = 8.1 Hz, 1H), 4.21 (d, *J* = 8.2 Hz, 1H), 4.18 (d, *J* = 8.1 Hz, 1H), 4.16 (d, *J* = 8.2 Hz, 1H), 4.14 (d, *J* = 8.0 Hz, 1H), 4.12-3.93 (m, 12H), 3.86-3.76 (m, 6H), 3.74-3.63 (m, 5H), 3.61-3.49 (m, 8H), 3.45-3.41 (m, 7H), 3.33-3.20 (m, 5H), 3.16-2.98 (m, 11H), 1.56-1.52 (m, 2H), 1.49-1.45 (m, 2H), 1.35-1.30 (m, 2H) ppm; ^{13}C NMR (100 MHz, $CDCl_3$): δ 161.8, 161.7, 156.4, 138.79, 138.76, 138.73, 138.6, 138.4, 138.1, 137.7, 137.5, 137.4, 137.3, 136.7, 129.2, 129.1, 129.07, 129.00, 128.9, 128.88, 128.83, 128.7, 128.6, 128.5, 128.47, 128.42, 128.3, 128.26, 128.21, 128.14, 128.13, 128.0, 127.8, 127.7, 127.68, 127.65, 127.59, 127.50, 99.7, 99.58, 99.50, 99.1, 92.78, 92.72, 92.6, 92.5, 80.1, 79.2, 79.0, 78.8, 77.4, 77.3, 77.1, 76.8, 76.2, 76.1, 76.0, 75.8, 75.2, 74.6, 74.4, 74.3, 74.0, 73.9, 73.7, 73.6, 73.5, 72.5, 71.4, 69.7, 68.1, 67.8, 66.7, 57.5, 57.1, 56.9, 41.0, 29.8, 29.6, 29.0, 23.3, 22.8 ppm; MALDI-TOF m/z $[M+Na]^+$ calc. for $C_{167}H_{173}Cl_{21}NaN_8O_{38}$: 3666.508; found 3665.875.

5-Aminopentyl 2-*N*-acetylamino-2-deoxy-β-D-glucopyranosyl-(1→4)-2-*N*-acetylamino-2-deoxy-β-D-glucopyranosyl-(1→4)-2-*N*-acetylamino-2-deoxy-β-D-glucopyranoside (6): Compound **3** (8.30 mg, 4.88 µmol) was treated with global deprotection A for 48 h to obtain fully deprotected trisaccharide **6** (2.70 mg, 3.80 µmol, 77%). ^{1}H NMR (600 MHz, D_2O): δ 4.54 (d, *J* = 8.1 Hz, 1H, H-1^B or H-1^C), 4.53 (d, *J* = 8.1 Hz, 1H, H-1^B or H-1^C), 4.44 (d, *J* = 8.1 Hz, 1H, H-1^A), 3.90-3.83 (m, 2 H, H-$6a^C$/O$CH_a$$CH_2$),

3.82-3.78 (m, 2 H, H-6a^A/H-6a^B), 3.75-3.60 (m, 9 H, H-2^B/H-2^C/H-6b^C/H-5^A/H-5^B/H-5^C/H-2^A/H-6b^B/H-6^C), 3.60-3.48 (m, 4 H, H-4^A/H-4^B/O*CH$_a$*CH$_2$/H-4^C), 3.47-3.40 (m, 3 H, H-3^A/H-3^B/H-3^C), 2.94 (t, *J*=7.7 Hz, 2 H, *CH$_2$*NH$_2$), 2.02 (2 x s, 6 H, CH$_3$), 1.98 (s, 3 H, CH$_3$), 1.62 (dt, *J*=15.2, 7.7 Hz, 2 H, OCH$_2$*CH$_2$*), 1.54 (dt, *J*=14.0, 6.9 Hz, 2 H, *CH$_2$*CH$_2$NH$_2$), 1.38-1.31 (m, 2H, OCH$_2$CH$_2$*CH$_2$*) ppm; ^{13}C NMR (100 MHz, D$_2$O): δ 175.8 (2 C, C=O), 175.6 (C=O), 102.7, 102.5 (C-1^B/C-1^C), 102.3 (C-1^A), 80.4, 80.3 (C-4^A/C-4^B), 77.1 (C-3^B), 75.7 (2 C, C-3^A/C-4^C), 74.6 (C-5^C), 73.6, 73.3 (C-5^A/C-5^B), 71.3 (OCH$_2$*CH$_2$*), 70.8 (C-3^C), 61.7 (C-6^C), 60.9 (2 C, C-6^B/C-6^A), 56.7 (2 C, C-2^B/C-2^C), 56.1 (C-2^A), 40.6 (*CH$_2$*NH$_2$), 29.3 (*CH$_2$*CH$_2$NH$_2$), 27.5 (OCH$_2$*CH$_2$*), 23.2 (OCH$_2$CH$_2$*CH$_2$*), 23.0 (3 C, CH$_3$) ppm; ESI-HRMS: m/z [M+H]$^+$ calcd. for $C_{29}H_{53}N_4O_{16}$: 713.3451; found 713.3443.

5-Aminopentyl 2-*N*-acetylamino-2-deoxy-β-D-glucopyranosyl-(1→4)-2-*N*-acetylamino-2-deoxy-β-D-glucopyranosyl-(1→4)-2-*N*-acetylamino-2-deoxy-β-D-glucopyranosyl-(1→4)-2-*N*-acetylamino-2-deoxy-β-D-glucopyranosyl-(1→4)-2-*N*-acetylamino-2-deoxy-β-D-glucopyranoside (7): Compound **4** (8.70 mg, 3.26 µmol) was treated with global deprotection A for 72 h to obtain fully deprotected pentasaccharide **7** (1.20 mg, 1.10 µmol, 34%). ^{1}H NMR (600 MHz, D$_2$O): δ 4.56-4.50 (m, 4H, H-1^{B-E}), 4.44 (d, *J* = 8.1 Hz, 1H, H-1^A), 3.91-3.83 (m, 2H, H-6a^E/O*CH$_a$*CH$_2$), 3.82-3.76 (m, 4H, H-6a^{A-D}), 3.75-3.40 (m, 26H H-2^{B-E}/H-6b^E/H-5^{A-D}/H-5^E/H-2^A/H-6b^{A-D}/H-4^{A-D}/O*CH$_a$*CH$_2$/H-4^E/H-3^{A-E}), 2.94 (t, *J* = 7.7 Hz, 2H, *CH$_2$*NH$_2$), 2.02-2.0 (4 × s, 12H, CH$_3$), 1.98 (s, 3H, CH$_3$), 1.62 (dt, *J*=15.2, 7.7 Hz, 2H, OCH$_2$*CH$_2$*), 1.54 (dt, *J*=14.0, 6.9 Hz, 2H, *CH$_2$*CH$_2$NH$_2$), 1.40-1.26 (m, 2H, OCH$_2$CH$_2$*CH$_2$*) ppm; ^{13}C NMR (100 MHz, D$_2$O): δ 175.8 (4 C, C=O), 175.6 (C=O), 102.7, 102.5, 102.4 (4C,C-1^{B-E}), 102.3 (C-1^A), 80.4, 80.3, 80.03 (4 C, C-4^{A-D}), 77.1 (C-3^A), 75.7 (4 C, C-3^B/C-3^C/C-3^D/C-4^E), 74.6 (C-5^E), 73.6-73.2 (4 C, C-5^{A-D}), 71.3 (OCH$_2$*CH$_2$*), 70.9 (C-3^E), 61.7 (C-6^E), 61.3-61.0 (4 C, C-6^{A-D}), 56.7-56.2 (5 C, C-2^{A-E}), 40.5 (*CH$_2$*NH$_2$), 29.3 (*CH$_2$*CH$_2$NH$_2$), 27.6 (OCH$_2$*CH$_2$*), 23.3 (6 C, 5 × CH$_3$/OCH$_2$CH$_2$*CH$_2$*) ppm; ESI-HRMS: m/z [M+H]$^+$ calcd. for $C_{45}H_{79}N_6O_{26}$: 1119.5039; found 1119.5059.

5-Aminopentyl 2-*N*-acetylamino-2-deoxy-β-D-glucopyranosyl-(1→4)-2-*N*-acetylamino-2-deoxy-β-D-glucopyranosyl-(1→4)-2-*N*-acetylamino-2-deoxy-β-D-glucopyranosyl-(1→4)-2-*N*-acetylamino-2-deoxy-β-D-glucopyranosyl-(1→4)-2-*N*-acetylamino-2-deoxy-β-D-glucopyranosyl-(1→4)-2-*N*-acetylamino-2-deoxy-β-D-glucopyranosyl-(1→4)-2-*N*-acetylamino-2-deoxy-β-D-glucopyranoside (8): Compound **5** (8.20 mg, 2.25 µmol) was treated with global deprotection A for 72 h to obtain fully deprotected heptasaccharide **8** (1.20 mg, 0.78 µmol, 35%). ^{1}H NMR (600 MHz, D$_2$O): δ 4.54-4.51 (m, 6H, H-1^B/H-1^C/H-1^D/H-1^E/H-1^F/H-1^G), 4.44 (d, *J* = 8.1 Hz, 1H, H-1^A), 3.90-3.82 (m, 2H, H-6a^G/O*CH$_a$*CH$_2$), 3.82-3.76 (m, 6H, H-6a^A/H-6a^B/H-6a^C/H-6a^D H-6a^E/H-6a^F), 3.75-3.40 (m, 36H, H-2^B/H-2^C/H-2^D/H-2^E/H-2^F/H-2^G/H-6b^E/H-5^A/H-5^B/H-5^C/H-5^D/H-5^E/H-5^F/H-5^G/H-2^A/H-6b^A/H-6b^B/H-6b^C/H-6b^D/H-6b^E/H-6b^F/H-4^A/H-4^B/H-4^C/H-4^D/H-4^E/H-4^F/O*CH$_a$*CH$_2$/H-4^G/H-3^A/H-3^B/H-3^C/H-3^D/H-3^E/H-3^F/H-3^G), 2.94 (t, *J*=7.7 Hz, 2H, *CH$_2$*NH$_2$), 2.02-2.0 (6 x s, 16H, CH$_3$), 1.98 (s, 3H, CH$_3$), 1.62 (dt, *J*=15.2, 7.7 Hz, 2H, OCH$_2$*CH$_2$*), 1.54 (dt, *J*=14.0, 6.9 Hz, 2H, *CH$_2$*CH$_2$NH$_2$), 1.40-1.26 (m, 2H, OCH$_2$CH$_2$*CH$_2$*) ppm; ^{13}C NMR (100 MHz, D$_2$O): δ 175.8 (6 C, C=O), 175.6 (C=O), 102.7-102.3 (7C, C-1^A/C-1^B/C-1^C/C-1^D/C-1^E/C-1^F/C-1^G), 80.4, 80.3, 80.0 (6 C, C-4^A/C-4^B/C-4^C/C-4^D/C-4^E/C-4^F), 77.1 (C-3^A), 75.7 (6 C, C-3^B/C-3^C/C-3^D/C-3^E/C-3^F/C-4^G), 74.6 (C-5^G), 73.6-73.2 (6 C, C-5^A/C-5^B/C-5^C/C-5^D/C-5^E/C-5^F), 71.3 (OCH$_2$*CH$_2$*), 70.9 (C-3^G), 61.7 (C-6^E), 61.3-61.0 (6 C, C-6^A/C-6^B/C-6^C/C-6^D/C-6^E/C-6^F), 56.7-56.2 (7 C, C-2^A/C-2^B/C-2^C/C-2^D/C-2^E/C-2^F/C-2^G), 40.5 (*CH$_2$*NH$_2$), 29.3 (*CH$_2$*CH$_2$NH$_2$), 27.6 (OCH$_2$*CH$_2$*), 23.3 (8 C, 7 x CH$_3$/OCH$_2$CH$_2$*CH$_2$*) ppm; ESI-HRMS: m/z [M+H]$^+$ calcd. for $C_{61}H_{105}N_8O_{36}$: 1525.6625; found 1525.6630.

Benzyloxycarbonylaminopentyl 2,3-di-*O*-benzoyl-4-*O*-benzyl-β-D-glucopyranosyl-(1→6)-2,3-di-*O*-benzoyl-4-*O*-benzyl-β-D-glucopyranosyl-(1→6)-2,3-di-*O*-benzoyl-4-*O*-benzyl-β-D-glucopyranoside (10): The synthesizer modules were applied as follows: A(**9**, 3 cycles)-B-C- A(**9**, 3

cycles)-B-C-A(**9**, 3 cycles)-B-C followed by resin cleavage. The crude product was purified by HPLC (YMC-diol column, Hex/EtOAc = 100/0 → 0/100) to obtain the trisaccharide **10** (12.7 mg, 7.84 µmol, 62% over 6 steps). ^{1}H NMR (400 MHz, $CDCl_3$): δ 7.95-7.85 (m, 12H), 7.52-7.23 (m, 23H), 7.16-7.10 (m, 11H), 6.96-6.92 (m, 4H), 5.76 (t, *J* = 9.3 Hz, 1H), 5.64 (t, *J* = 9.3 Hz, 2H), 5.48 (t, *J* = 8.6 Hz, 1H), 5.39 (t, *J* = 8.6 Hz, 1H), 5.29 (t, *J* = 8.6 Hz, 1H), 5.06 (bs, 2H), 4.80 (d, *J* = 7.6 Hz, 1H), 4.66 (d, *J* = 8.2 Hz, 1H), 4.62-4.61 (bs, 2H), 4.58 (d, *J* = 7.5 Hz, 1H), 4.34-4.24 (m, 3H), 4.19-4.10 (m, 2H), 4.02 (t, *J* = 9.8 Hz, 2H), 3.89-3.74 (m, 6H), 3.68-3.59 (m, 3H), 3.37 (bs, 1H), 2.90 (bs, 2H), 1.49-1.38 (m, 2H), 1.33-1.28 (m, 2H), 1.19-1.16 (m, 2H) ppm; ^{13}C NMR (100 MHz, $CDCl_3$): δ 165.9, 165.8, 165.7, 165.4, 165.3, 165.2, 156.4, 137.3, 137.2, 137.1, 136.8, 133.3, 133.28, 133.26, 133.23, 129.87, 129.81, 129.7, 129.6, 129.5, 129.49, 129.44, 129.3, 128.6, 128.55, 128.50, 128.47, 128.46, 128.40, 128.3, 128.18, 128.13, 128.11, 128.0, 127.9, 127.8, 101.7, 101.4, 101.0, 77.4, 77.3, 77.1, 76.8, 76.1, 76.0, 75.9, 75.7, 75.25, 75.23, 75.0, 74.99, 74.91, 74.8, 74.7, 72.4, 72.17, 72.14, 69.8, 68.6, 68.3, 66.5, 61.5, 41.0, 29.8, 29.4, 29.0, 23.2 ppm; MALDI-TOF m/z $[M+Na]^+$ calc. for $C_{94}H_{91}NNaO_{24}$: 1641.734; found 1641.091

Benzyloxycarbonylaminopentyl 2,3-di-*O*-benzoyl-4-*O*-benzyl-β-D-glucopyranosyl-(1→6)-2,3-di-*O*-benzoyl-4-*O*-benzyl-β-D-glucopyranosyl-(1→6)-2,3-di-*O*-benzoyl-4-*O*-benzyl-β-D-glucopyranosyl-(1→6)-2,3-di-*O*-benzoyl-4-*O*-benzyl-β-D-glucopyranosyl-(1→6)-2,3-di-*O*-benzoyl-4-*O*-benzyl-β-D-glucopyranoside (11): The synthesizer modules were applied as follows: A(**9**, 3 cycles)-B-C-A(**9**, 3 cycles)-B-C-A(**9**, 3 cycles)-B-C-A(**9**, 3 cycles)-B-C-A(**9**, 3 cycles)-B-C followed by resin cleavage. The crude product was purified by HPLC (YMC-diol column, Hex/EtOAc = 100/0 → 0/100) to obtain the pentasaccharide **11** (14.5 mg, 5.70 µmol, 45% over 10 steps). ^{1}H NMR (400 MHz, $CDCl_3$): δ 8.28 (d, *J* = 7.3 Hz, 2H), 8.18 (d, *J* = 7.7 Hz, 2H), 8.11-7.94 (m, 14H), 7.90 (d, *J* = 7.7 Hz, 2H), 7.53-7.27 (m, 21H), 7.18-6.99 (m, 20H), 6.96-6.82 (m, 10H), 6.68 (d, *J* = 7.4 Hz, 2H), 6.62-6.49 (m, 7H), 6.00-5.78 (m, 5H), 5.72-5.65 (m, 2H), 5.60 (t, *J* = 8.9 Hz, 1H), 5.53-5.49 (m, 1H), 5.52 (d, *J* = 8.1 Hz, 1H), 5.37 (d, *J* = 7.8 Hz, 1H), 5.23 (d, *J* = 8.0 Hz, 1H), 5.07-4.99 (m, 2H), 4.96-4.87 (m, 2H), 4.74 (d, *J* = 10.8 Hz, 2H), 4.67 (d, *J* = 7.8 Hz, 1H), 4.65 (d, *J* = 8.1 Hz, 1H), 4.55 (d, *J* = 10.9 Hz, 1H), 4.51 (t, *J* = 9.1 Hz, 1H), 4.43 (d, *J* = 10.4 Hz, 1H), 4.37-4.22 (m, 8H), 4.15-4.09 (m, 3H), 4.05-3.74 (m, 8H), 3.61-3.50 (m, 3H), 3.35-3.29 (m, 1H), 2.90 (bs, 1H), 1.97-1.90 (m, 2H), 1.67-1.54 (m, 2H), 1.39-1.34 (m, 2H) ppm; ^{13}C NMR (100 MHz, $CDCl_3$): δ 165.98, 165.95, 165.90, 165.88, 165.84, 165.7, 165.6, 164.9, 164.76, 164.74, 156.5, 138.2, 137.5, 137.4, 137.0, 136.7, 136.6, 133.35, 133.31, 133.2, 132.98, 132.93, 132.7, 132.5, 132.3, 130.6, 130.3, 130.2, 130.1, 130.06, 130.00, 129.9, 129.83, 129.80, 129.76, 129.73, 129.68, 129.64, 129.60, 128.99, 128.92, 128.8, 128.6, 128.57, 128.53, 128.45, 128.42, 128.37, 128.33, 128.30, 128.2, 128.09, 128.06, 128.01, 127.9, 127.8, 127.7, 104.9, 103.8, 102.4, 101.9, 100.5, 79.6, 79.3, 79.1, 78.5, 77.3, 76.5, 76.3, 76.2, 76.1, 75.8, 75.76, 75.72, 75.3, 75.24, 75.20, 75.1, 74.3, 73.7, 73.2, 72.9, 72.7, 72.47, 72.41, 72.2, 69.9, 66.3, 61.8, 29.2 , 28.8, 23.2 ppm; ESI-HRMS: m/z $[M+H]^+$ calcd. for $C_{149}H_{139}NO_{38}$: 2538.9047; found 2538.8938.

Benzyloxycarbonylaminopentyl 2,3-di-*O*-benzoyl-4-*O*-benzyl-β-D-glucopyranosyl-(1→6)-2,3-di-*O*-benzoyl-4-*O*-benzyl-β-D-glucopyranosyl-(1→6)-2,3-di-*O*-benzoyl-4-*O*-benzyl-β-D-glucopyranosyl-(1→6)-2,3-di-*O*-benzoyl-4-*O*-benzyl-β-D-glucopyranosyl-(1→6)-2,3-di-*O*-benzoyl-4-*O*-benzyl-β-D-glucopyranosyl-(1→6)-2,3-di-*O*-benzoyl-4-*O*-benzyl-β-D-glucopyranosyl-(1→6)-2,3-di-*O*-benzoyl-4-*O*-benzyl-β-D-glucopyranoside (12): The synthesizer modules were applied as follows: A(**9**, 3 cycles)-B-C-A(**9**, 3 cycles)-B-C-A(**9**, 3 cycles)-B-C-A(**9**, 3 cycles)-B-C-A(**9**, 3 cycles)-B-C-A(**9**, 3 cycles)-B-C-A(**9**, 3 cycles)-B-C followed by resin cleavage. The crude product was purified by HPLC (YMC-diol column, Hex/EtOAc = 100/0 → 0/100) to obtain the heptasaccharide **12** (18.4 mg, 5.32 µmol, 42% over 14 steps). ^{1}H NMR (400 MHz, $CDCl_3$): δ 8.34-8.27 (m, 8H), 8.24 (d, *J* = 7.7 Hz, 2H), 8.21-8.16 (m, 8H), 8.13 (d, *J* = 7.7 Hz, 2H), 8.08 (d, *J* = 7.2 Hz, 2H), 7.98-7.91 (m, 6H), 7.55-7.40 (m, 11H), 7.38-7.27 (m, 13H), 7.25-7.09 (m, 23H), 7.05 (t, *J* = 7.6 Hz, 2H),

6.99-6.87 (m, 11H), 6.83-6.77 (m, 4H), 6.70-6.64 (m, 5H), 6.61-6.37 (m, 12H), 6.07-5.99 (m, 4H), 5.93-5.85 (m, 3H), 5.83 (t, *J* = 8.6 Hz, 1H, H), 5.77 (t, *J* = 7.8 Hz, 1H), 5.72-5.63 (m, 2H), 5.57 (d, *J* = 6.9 Hz, 1H), 5.55-5.52 (m, 2H), 5.32 (d, *J* = 8.1 Hz, 1H), 5.30 (d, *J* = 7.9 Hz, 1H), 5.29 (d, *J* = 7.9 Hz, 1H), 5.23-5.18 (m, 1H), 5.09-5.01 (m, 4H), 4.95-4.81 (m, 2H), 4.70 (d, *J* = 8.5 Hz, 1H), 4.66-4.61 (m, 1H), 4.65 (d, *J* = 8.1 Hz, 1H), 4.50 (d, *J* = 9.4 Hz, 1H), 4.45-3.94 (m, 23H), 3.89-3.79 (m, 3H), 3.71-3.67 (m, 1H), 3.63-3.48 (m, 7H), 3.21-3.14 (m, 2H), 2.94 (bs, 1H), 1.91-1.86 (m, 2H), 1.68-1.64 (m, 2H), 1.46-1.41 (m, 2H) ppm; ^{13}C NMR (100 MHz, $CDCl_3$): δ 165.95, 165.92, 165.90, 165.85, 165.81, 165.7, 165.6, 165.1, 165.0, 164.3, 156.5, 138.1, 137.6, 137.49, 137.43, 137.1, 136.79, 136.74, 133.2, 133.1, 132.9, 132.8, 132.7, 132.57, 132.51, 132.4, 130.7, 130.6, 130.5, 130.4, 130.3, 130.2, 130.19, 130.16, 130.12, 130.10, 130.05, 130.02, 129.96, 129.93, 129.90, 129.8, 129.7, 129.37, 129.34, 129.0, 128.9, 128.8, 128.54, 128.51, 128.46, 128.44, 128.41, 128.34, 128.32, 128.2, 128.1, 128.0, 127.97, 127.94, 127.8, 127.74, 127.71, 104.3, 103.4, 103.2, 102.6, 102.0, 100.5, 79.8, 79.7, 78.9, 78.8, 78.5, 77.4, 77.3, 77.1, 76.8, 76.2, 76.1, 76.0, 75.86, 75.80, 75.5, 75.4, 75.2, 75.1, 73.9, 73.8, 73.4, 73.2, 72.9, 72.8, 72.6, 72.5, 72.4, 72.2, 69.9, 66.3, 61.8, 41.2, 29.8, 29.2, 28.8, 23.2 ppm; MALDI-TOF m/z $[M+Na]^+$ calc. for $C_{202}H_{187}NNaO_{52}$: 3482.195; found 3481.861.

5-Aminopentyl β-D-glucopyranosyl-(1→6)-β-D-glucopyranosyl-(1→6)-β-D-glucopyranoside (13): Compound **10** (8.30 mg, 4.88 µmol) was treated with global deprotection B to obtain fully deprotected trisaccharide **13** (2.30 mg, 3.90 µmol, 49% over 2 steps); ^{1}H NMR (600 MHz, D_2O): δ 4.51 (d, *J* = 7.9 Hz, 1H, H-1^{B} or H-1^{C}), 4.49 (d, *J* = 7.9 Hz, 1H, H-1^{B} or H-1^{C}), 4.46 (d, *J* = 8.1 Hz, 1H, H-1^{A}), 4.19 (m, 2H, H-6a^{A}/H-6a^{B}), 3.94-3.88 (m, 2H, H-6a^{C}/OCH_aCH$_2$), 3.87-3.81 (m, 2H, H-6b^{A}/H-6b^{B}), 3.73-3.64 (m, 2H, H-6b^{C}/OCH_bCH$_2$), 3.63-3.58 (m, 2H, H-5^{B}/H-5^{C}), 3.50-3.35 (m, 7H, H-5^{A}/H-3^{A}/H-3^{B}/H-3^{C}/H-4^{A}/H-4^{B}/H-4^{C}), 3.33-3.27 (m, 2H, H-2^{B}/H-2^{C}), 3.24 (t, *J*=8.1 Hz, 1H, H-2^{A}), 2.99 (t, *J*=7.0 Hz, 2H, CH_2NH$_2$), 1.71-1.62 (m, 4H, OCH$_2$$CH_2CH_2$$CH_2$), 1.48-1.40 (m, 2H, OCH$_2CH_2$$CH_2$) ppm. ^{13}C NMR (150 MHz, D_2O): δ 103.6 (2 C, C-1^{B}/C-1^{C}), 102.9 (C-1^{A}), 76.6, 76.4, 76.3 (4 C, H-5^{A}/C-3^{A}/C-3^{B}/C-3^{C}), 75.6 (2 C, C-5^{B}/C-5^{C}), 73.7 (3 C, C-2^{A}/C-2^{B}/C-2^{C}), 70.9 (OCH_2CH$_2$), 70.3, 70.1 (3 C, C-4^{A}/C-4^{B}/C-4^{C}), 69.5, 69.2 (C-6^{A}/C-6^{B}), 61.4 (C-6^{C}), 40.2 (CH_2NH$_2$), 28.8, 26.8 (OCH$_2$$CH_2CH_2$$CH_2$), 22.7 (OCH$_2CH_2$$CH_2$) ppm; ESI-HRMS: m/z $[M+H]^+$ calcd. for $C_{23}H_{43}NO_{16}$: 590.2654; found 590.2664.

5-Aminopentyl β-D-glucopyranosyl-(1→6)-β-D-glucopyranosyl-(1→6)-β-D-glucopyranosyl-(1→6)-β-D-glucopyranosyl-(1→6)-β-D-glucopyranoside (14) Compound **11** (14.5 mg, 5.70 µmol) was treated with global deprotection B to obtain fully deprotected pentasaccharide **14** (3.86 mg, 3.90 µmol, 74% over 2 steps).^{1}H NMR (600 MHz, D_2O): δ 4.50-4.47 (m, 4H, H-1^{B-E}), 4.43 (d, *J* = 8.1 Hz, 1H, H-1^{A}), 4.20-4.16 (m, 4H, H-6a^{A-D}), 3.92-3.87 (m, 2H, H-6a^{E}/OCH_aCH$_2$), 3.86-3.79 (m, 4H, H-6b^{A-D}), 3.73-3.64 (m, 2H, H-6b^{E}/OCH_bCH$_2$), 3.61-3.56 (m, 4H, H-5^{B-E}), 3.48-3.34 (m, 11H, H-5^{A}/H-3^{A-E}/H-4^{A-E}), 3.31-3.26 (m, 4H, H-2^{B-E}), 3.22 (t, *J*=8.1 Hz, 1H, H-2^{A}), 2.97 (t, *J*=7.0 Hz, 2H, CH_2NH$_2$), 1.71-1.62 (m, 4H, OCH$_2$$CH_2CH_2$$CH_2$), 1.48-1.40 (m, 2H, OCH$_2CH_2$$CH_2$) ppm; ^{13}C NMR (150 MHz, D_2O): δ 103.9, 103.8, 103.7 (2 C) (C-1^{B-E}), 103.0 (C-1^{A}), 76.8, 76.6, 76.5 (6 C, H-5^{A}/C-3^{A-E}), 75.8 (4 C, C-5^{B-E}), 73.9 (5 C, C-2^{A-E}), 71.0 (OCH_2CH$_2$), 70.5, 70.4 (5 C, C-4^{A-E}), 69.7-69.5 (4 C, C-6^{A-D}), 61.7 (C-6^{E}), 40.2 (CH_2NH$_2$), 29.0, 27.3 (OCH$_2$$CH_2CH_2$$CH_2$), 22.9 (OCH$_2CH_2$$CH_2$) ppm; ESI-HRMS: m/z $[M+H]^+$ calcd. for $C_{35}H_{64}NO_{26}$: 914.3710; found 914.3707

5-Aminopentyl β-D-glucopyranosyl-(1→6)-β-D-glucopyranosyl-(1→6)-β-D-glucopyranosyl-(1→6)-β-D-glucopyranosyl-(1→6)-β-D-glucopyranosyl-(1→6)-β-D-glucopyranosyl-(1→6)-β-D-glucopyranoside (15) Compound **12** (18.0 mg, 5.20 µmol) was treated with global deprotection B to obtain fully deprotected heptasaccharide **15** (4.28 mg, 3.46 µmol, 66% over 2 steps). ^{1}H NMR (600 MHz, D_2O): δ 4.51-4.47 (m, 6H, H-1^{B-G}), 4.44 (d, *J* = 8.1 Hz, 1H, H-1^{A}), 4.21-4.16 (m, 6H, H-6a^{A-F}), 3.92-3.87 (m, 2H, H-6a^{G}/OCH_aCH$_2$), 3.86-3.79 (m, 6H, H-6b^{A-G}), 3.73-3.64 (m, 2H, H-6b^{G}/OCH_bCH$_2$), 3.62-

3.56 (m, 6H, H-5^{B-G}), 3.48-3.34 (m, 15H, H-5^{A}/H-3^{A-G}/H-4^{A-G}), 3.31-3.26 (m, 6H, H-2^{B-G}), 3.23 (t, *J*=8.1 Hz, 1H, H-2^{A}), 2.97 (t, *J*=7.0 Hz, 2H, CH_2NH_2), 1.71-1.62 (m, 4H, $OCH_2CH_2CH_2CH_2$), 1.48-1.40 (m, 2H, $OCH_2CH_2CH_2$) ppm; ^{13}C NMR (150 MHz, D_2O): δ 103.9-103.7 (6 C) (C-1^{B-G}), 103.1 (C-1^{A}), 76.8, 76.6, 76.5 (8 C, H-5^{A}/C-3^{A-G}), 75.8 (6 C, C-5^{B-G}), 73.9 (7 C, C-2^{A-G}), 71.0 (OCH_2CH_2), 70.5-70.3 (7 C, C-4^{A-G}), 69.7-69.5 (6 C, C-6^{A-F}), 61.7 (C-6^{G}), 40.2 (CH_2NH_2), 29.0, 27.3 ($OCH_2CH_2CH_2CH_2$), 22.9 ($OCH_2CH_2CH_2$) ppm; ESI-HRMS: m/z $[M+H]^+$ calcd. for $C_{47}H_{84}NO_{36}$: 1238.4788; found 1238.4768.

4-Methylphenyl 2,4,6-tri-*O*-benzyl-3-*O*-(2-naphthylmethyl)-α-D-glucopyranosyl-(1→3)-2,4,6-tri-*O*-benzyl-1-thio-α-D-glucopyranoside (18): A mixture of donor **16**[33] (0.94 g, 1.23 mmol) and the acceptor **17**[36] (0.48 g, 0.86 mmol) were co-evaporated with toluene. Anhydrous DCM (13.7 mL) and anhydrous DMF (1.52 mL, 19.70 mmol) were added and the solution was stirred over fresh flame-dried MS 4 Å (1.40 g, powdered) under nitrogen atmosphere for 20 minutes. The reaction flask was cooled to -78 °C and triflic acid (0.11 mL, 1.23 mmol) was added dropwise. After 45 minutes, the reaction solution was stirred at 0 °C till completion as indicated by TLC analysis. The reaction was quenched by adding NEt_3 (0.17 mL, 1.23 mmol). The reaction solution was filtered through a bed of Celite and concentrated under reduced pressure. The product was purified by silica gel column chromatography (EtOAc/toluene = 1:99 – 1:32) and compound **18** (0.83 g, 85% yield) was obtained as a yellow foam. R_f = 0.3 (toluene/EtOAc = 19/1); ^{1}H NMR (400 MHz, $CDCl_3$): δ 7.42-7.40 (m, 2H), 7.37-7.33 (m, 4H), 7.32-7.26 (m, 3H), 7.24-6.93 (m, 32H), 5.60 (d, *J* = 3.6 Hz, 1H), 5.01 (d, *J* = 11.1 Hz, 1H), 4.96 (d, *J* = 11.2 Hz, 1H), 4.82 (d, *J* = 11.9 Hz, 2H), 4.74 (d, *J* = 10.8 Hz, 1H), 4.62 (d, *J* = 11.8 Hz, 1H), 4.58 (d, *J* = 9.7 Hz, 1H), 4.53 (d, *J* = 11.8 Hz, 1H), 4.51 (d, *J* = 9.6 Hz, 1H), 4.51 (d, *J* = 7.8 Hz, 1H), 4.51 (d, *J* = 10.0 Hz, 1H), 4.47 (d, *J* = 11.8 Hz, 1H), 4.42 (d, *J* = 12.2 Hz, 2H), 4.32 (d, *J* = 10.8 Hz, 1H), 4.14 (d, *J* = 12.0 Hz, 1H), 4.09-4.05 (m, 1H), 4.03 (t, *J* = 9.5 Hz, 1H), 3.96 (t, *J* = 9.0 Hz, 1H), 3.74 (t, *J* = 9.4 Hz, 1H), 3.68-3.58 (m, 3H), 3.51 (dd, *J* = 3.5, 9.8 Hz, 1H), 3.44 (t, *J* = 9.2 Hz, 1H), 3.41-3.37 (m, 1H), 3.24-3.17 (m, 2H), 2.23 (s, 3H) ppm; ^{13}C NMR (100 MHz, $CDCl_3$): δ 138.6, 138.4, 138.2, 138.0, 137.88, 137.82, 137.9, 136.1, 133.2, 132.9, 132.5, 129.88, 129.81, 129.8, 128.8, 128.7, 128.47, 128.44, 128.40, 128.38, 128.35, 128.29, 128.23, 128.1, 128.03, 128.00, 127.8, 127.77, 127.73, 127.71, 127.67, 127.61, 127.4, 126.8, 126.4, 126.08, 126.01, 125.8, 125.4, 97.3, 87.9, 82.5, 79.4, 79.2, 78.96, 78.90, 78.2, 77.4, 77.3, 77.1, 76.8, 75.6, 75.2, 74.0, 73.47, 73.44, 70.0, 68.8, 68.0, 21.2 ppm. ESI-HRMS: m/z $[M+Na]^+$ calcd. for $C_{72}H_{72}NaO_{10}S$: 1152.4810; found 1152.4768.

2,4,6-tri-*O*-benzyl-3-*O*-(2-naphthylmethyl)-α-D-glucopyranosyl-(1→3)-2,4,6-tri-*O*-benzyl-α/β-D-glucopyrnoside (19): To a solution of disaccharide **18** (0.56 g, 0.50 mmol) in THF/H_2O (7.1 mL, 4/1) *N*-bromosuccinimide (0.26 g, 1.5 mmol) was added at 0 °C. After 10 minutes, the reaction was warmed to 23 °C and stirred for 3 h more. Then it was quenched by adding satd. aq. $NaHCO_3$ and diluted with EtOAc. The organic layer was washed with $NaHCO_3$ solution and brine, dried over $MgSO_4$, filtered and concentrated under reduced pressure. The product was purified by silica gel column chromatography (EtOAc/hexane = 1:3 – 1:2) and compound **19** (0.44 g, 87% yield, α/β = 2:1) was obtained as a white foam. R_f = 0.25 (EtOAc/hexane = 1:2); ^{1}H NMR (400 MHz, $CDCl_3$): δ 7.46-7.37 (m, 4H), 7.34-7.26 (m, 13H), 7.25-7.13 (m, 15H), 7.11-7.01 (m, 5H), 5.59 (d, *J* = 3.5 Hz, 1H), 5.31 (d, *J* = 3.6 Hz, 0.66H), 5.07 (d, *J* = 11.2 Hz, 1H), 5.02 (t, *J* = 10.4 Hz, 1H), 4.96-4.91 (m, 1H), 4.84 (d, *J* = 11.0 Hz, 1H), 4.74 (d, *J* = 7.5 Hz, 0.36H), 4.70-4.54 (m, 6H), 4.51-4.40 (m, 4H), 4.34-4.25 (m, 2H), 4.22 (t, *J* = 9.2 Hz, 1H), 4.13-4.08 (m, 1H), 4.04-3.95 (m, 1H), 3.82 (t, *J* = 9.5 Hz, 1H), 3.77-3.51 (m, 6H), 3.44-3.33 (m, 3H) ppm; ^{13}C NMR (100 MHz, $CDCl_3$): δ 138.7, 138.4, 138.07, 138.03, 137.9, 137.2, 136.3, 133.4, 132.9, 129.0, 128.9, 128.6, 128.54, 128.51, 128.43, 128.42, 128.39, 128.37, 128.26, 128.22, 128.1, 128.08, 128.06, 128.04, 127.94, 127.91, 127.84, 127.81, 127.7, 127.63, 127.60, 127.4, 127.1, 126.8, 126.5, 126.0, 125.8, 97.9, 97.5, 97.4, 90.7, 82.4, 81.4, 79.5, 78.7, 78.6, 78.3, 78.2, 77.4, 77.3, 77.1, 76.8, 76.0, 75.6,

75.0, 74.7, 73.9, 73.7, 73.6, 73.5, 73.1, 70.2, 70.1, 68.8, 68.5, 68.3, 29.8 ppm. ESI-HRMS: m/z $[M+Na]^+$ calcd. for $C_{65}H_{66}NaO_{11}$: 1046.4569; found 1046.4530.

2,4,6-Tri-*O*-benzyl-3-*O*-(2-naphthylmethyl)-α-D-glucopyranosyl-(1→3)-2,4,6-tri-*O*-benzyl-α/β-D-glucopyranosyl-1-(*N*-phenyl)-2,2,2-trifluoroacetimidate (20): To a solution of disaccharide hemiacetal **19** (0.37 g, 0.36 mmol) and Cs_2CO_3 (0.24 g, 0.72 mmol) in acetone (7.5 mL), 2,2,2-trifluoro-*N*-phenylacetimidoyl chloride (0.12 mL, 0.72 mmol) was added at 23 °C and the reaction was stirred under nitrogen atmosphere for 3 h. The reaction solution was filtered through a bed of Celite and concentrated under reduced pressure. The product was purified by silica gel column chromatography (EtOAc/hexane = 1:9 – 1:4) and compound **20** (0.38 g, 88% yield, α/β = 2:1) was obtained as a yellow viscous liquid. R_f = 0.5 (EtOAc/hexane = 1/4); ^{1}H NMR (400 MHz, $CDCl_3$): δ 7.80-7.77 (m, 1H), 7.73 (d, *J* = 8.4 Hz, 1H), 7.68-7.66 (m, 2H), 7.56 (d, *J* = 8.0 Hz, 1H), 7.46-7.36 (m, 4H), 7.32-7.02 (m, 31H), 6.79 (d, *J* = 7.7 Hz, 1H), 6.68 (br. d, *J* = 7.7 Hz, 1H), 5.59 (d, *J* = 3.5 Hz, 1H), 5.09-4.97 (m, 3H), 4.93 (d, *J* = 11.6 Hz, 1H), 4.85-4.70 (m, 4H), 4.65-4.56 (m, 4H), 4.54-4.43 (m, 4H), 4.35-4.26 (m, 3H), 4.21 (d, *J* = 10.3 Hz, 1H), 4.14-4.06 (m, 2H), 3.93-3.89 (m, 2H), 3.75-3.58 (m, 6H), 3.43-3.36 (m, 2H) ppm; ^{13}C NMR (100 MHz, $CDCl_3$): δ 155.0, 154.7, 138.7, 138.6, 138.18, 138.12, 138.03, 138.00, 137.9, 137.7, 137.3, 136.3, 136.2, 135.1, 133.4, 132.9, 129.4, 129.2, 128.8, 128.79, 128.75, 128.57, 128.54, 128.51, 128.44, 128.40, 128.37, 128.31, 128.26, 128.20, 128.15, 128.11, 128.07, 128.02, 127.98, 127.94, 127.91, 127.8, 127.78, 127.75, 127.67, 127.65, 127.5, 126.97, 126.95, 126.5, 126.4, 126.06, 126.04, 125.88, 125.86, 124.4, 120.7, 120.6, 120.5, 119.4, 117.2, 114.3, 97.6, 82.4, 82.3, 79.57, 79.50, 79.3, 78.1, 77.8, 77.7, 77.3, 75.7, 75.6, 75.4, 75.1, 75.0, 74.9, 73.9, 73.8, 73.7, 73.6, 73.5, 73.4, 73.2, 72.9, 70.3, 70.2, 68.09, 68.00, 32.0, 29.8, 29.5, 22.8, 14.2 ppm; ESI-HRMS: m/z $[M+Na]^+$ calcd. for $C_{73}H_{70}F_3NNaO_{11}$: 1217.4865; found 1217.4825;

4-Methyphenyl 2,4,6-tri-*O*-benzyl-α-D-glucopyranosyl-(1→3)-2,4,6-tri-*O*-benzyl-1-thio-α-D-glucopyranoside (21): To the solution of compound **18** (0.13 g, 0.11 mmol) in DCM/H_2O (1.2 mL, 10:1) at 0 °C, DDQ (28.7 mg, 0.12 mmol) was added. After 10 minutes, the reaction was warmed to 23 °C and stirred for 2 h more. The reaction was quenched by adding satd. aq. $NaHCO_3$ (1 mL) and diluted with DCM. The organic layer was washed with brine, dried over $MgSO_4$, filtered and concentrated under reduced pressure. The product was purified by silica gel column chromatography (EtOAc/hexane = 1:5 – 2:7). Compound **21** (94.5 mg, 83% yield), was obtained as a white foam. R_f = 0.25 (EtOAc/hexane = 1:4); ^{1}H NMR (400 MHz, $CDCl_3$): δ 7.50 (d, *J* = 7.8 Hz, 2H), 7.45 (d, *J* = 7.6 Hz, 2H), 7.36-7.28 (m, 9H), 7.22-7.12 (m, 15H), 7.09 (d, *J* = 7.5 Hz, 3H), 7.03 (d, *J* = 7.8 Hz, 3H), 5.69 (d, *J* = 3.4 Hz, 1H), 4.92 (d, *J* = 9.7 Hz, 1H), 4.88 (d, *J* = 11.9 Hz, 1H), 4.73 (d, *J* = 11.2 Hz, 1H), 4.66-4.49 (m, 8H, $ArCH_2$), 4.46 (d, *J* = 11.8 Hz, 1H), 4.40 (d, *J* = 11.1 Hz, 1H), 4.23 (d, *J* = 12.1 Hz, 1H), 4.16-4.08 (m, 3H), 4.03 (t, *J* = 9.1 Hz, 1H), 3.82-3.68 (m, 4H), 3.55 (t, *J* = 9.5 Hz, 1H), 3.49 (t, *J* = 8.8 Hz, 2H), 3.40 (dd, *J* = 3.4, 9.9 Hz, 1H), 3.26 (br. d, *J* = 3.4 Hz, 2H), 2.32 (s, 3H) ppm; ^{13}C NMR (150 MHz, $CDCl_3$): δ 138.6, 138.3, 138.1, 137.9, 137.8, 137.7, 132.5, 129.7, 128.7, 128.4, 128.3, 128.2, 128.1, 128.0, 127.9, 127.8, 127.6, 127.5, 127.4, 126.6 (42 C), 96.7, 87.7, 79.4, 79.0, 78.9 (2 C), 78.8, 78.0, 75.0, 74.7, 73.8, 73.4, 73.3 (3 C), 69.5, 68.8, 68.0, 29.7, 21.1 ppm; ESI-HRMS: m/z $[M+Na]^+$ calcd. for $C_{61}H_{64}NaO_{10}S$: 1012.4184; found 1012.4147.

4-Methylphenyl 2,4,6-tri-*O*-benzyl-3-*O*-(2-naphthylmethyl)-α-D-glucopyranosyl-(1→3)-2,4,6-tri-*O*-benzyl-α-D-glucopyranosyl-(1→3)-2,4,6-tri-*O*-benzyl-α-D-glucopyranosyl-(1→3)-2,4,6-tri-*O*-benzyl-1-thio-α-D-glucopyranoside (22): A mixture of disaccharide donor **20** (128 mg, 107 µmol) and the disaccharide acceptor **21** (74.1 mg, 74.9 µmol) was co-evaporated with toluene. Anhydrous DCM (2.0 mL) and anhydrous DMF (132 µL, 1.71 mmol) were added to the flask and the solution was stirred over fresh flame-dried MS 4 Å (0.2 g, powdered) under nitrogen atmosphere for 20 minutes. The

reaction flask was cooled to -78 °C and triflic acid (9.62 µL, 107 µmol) was added dropwise. After 45 minutes, the reaction solution was allowed to warm to 0 °C and stirred at this temperature for 14 h more. Then, the reaction was quenched by adding NEt_3 (18.0 µL, 127 µmol), filtered through a bed of Celite and concentrated under reduced pressure. The product was purified by silica gel column chromatography (EtOAc/toluene = 1:99 – 1:24) and compound **22** (121 mg, 81% yield) was obtained as a white foam. R_f = 0.25 (EtOAc/toluene = 1:19); ^{1}H NMR (400 MHz, $CDCl_3$): δ 7.71-7.66 (m, 2H), 7.61 (d, *J* = 8.4 Hz, 1H), 7.57-7.52 (m, 2H), 7.42-7.34 (m, 7H), 7.25-6.93 (m, 53H), 6.90-6.83 (m, 3H), 6.78-6.75 (m, 3H), 5.69 (d, *J* = 3.5 Hz, 1H), 5.59 (d, *J* = 3.7 Hz, 1H), 5.55 (d, *J* = 3.5 Hz, 1H), 4.93-4.58 (m, 11H), 4.56 (d, *J* = 7.0 Hz, 1H), 4.53-4.17 (m, 22H), 4.15-4.05 (m, 6H), 3.98-3.91 (m, 2H), 3.77-3.53 (m, 7H), 3.48-3.19 (m, 13H), 2.24 (s, 3H) ppm; ^{13}C NMR (100 MHz, $CDCl_3$): δ 138.9, 138.6, 138.4, 138.29, 138.21, 138.1, 138.02, 138.00, 137.8, 137.77, 137.71, 136.3, 133.3, 132.9, 132.4, 130.2, 129.8, 128.9, 128.7, 128.5, 128.47, 128.45, 128.41, 128.37, 128.35, 128.31, 128.27, 128.21, 128.19, 128.16, 128.13, 128.12, 128.09, 128.07, 128.02, 127.92, 127.90, 127.86, 127.81, 127.79, 127.76, 127.71, 127.69, 127.65, 127.63, 127.5, 127.48, 127.47, 127.43, 127.2, 127.1, 126.9, 126.7, 126.54, 126.51, 126.14, 126.10, 125.9, 125.7, 97.3, 96.29, 96.24, 88.3, 82.2, 79.7, 79.5, 79.4, 79.3, 79.0, 78.8, 78.6, 78.0, 77.4, 77.3, 77.1, 76.8, 75.8, 75.6, 75.4, 75.2, 74.9, 74.6, 73.7, 73.5, 73.4, 73.39, 73.37, 73.2, 73.07, 73.01, 71.2, 70.3, 70.1, 69.9, 69.8, 69.6, 68.9, 68.4, 68.2, 21.2 ppm; ESI-HRMS: m/z $[M+Na]^+$ calcd. for $C_{126}H_{128}NaO_{20}S$: 2015.8612; found 2015.8890.

2,4,6-tri-*O*-benzyl-3-*O*-(2-naphthylmethyl)-α-D-glucopyranosyl-(1→3)-2,4,6-tri-*O*-benzyl-α-D-glucopyranosyl-(1→3)-2,4,6-tri-*O*-benzyl-α-D-glucopyranosyl-(1→3)-2,4,6-tri-*O*-benzyl-α/β-D-glucopyranoside (23): To a solution of tetrasaccharide **22** (100 mg, 50.1 µmol) in THF/H_2O (1.0 mL, 4/1) *N*-bromosuccinimide (26.8 mg, 150 µmol) was added at 0 °C. After 10 minutes, the reaction was allowed to warm to 23 °C and stirred for 3 h more. The reaction was quenched by the addition of aq. satd. $NaHCO_3$ and diluted with EtOAc. The organic layer was washed with $NaHCO_3$ solution and brine, dried over $MgSO_4$, filtered and concentrated under reduced pressure. The product was purified by silica gel column chromatography (EtOAc/hexane = 1:3 – 7:13) and compound **23** (74.8 mg, 79% yield, α/β = 2:1) was obtained as a colorless viscous liquid. R_f = 0.2 (EtOAc/hexane = 1/2); ^{1}H NMR (400 MHz, $CDCl_3$): δ 7.78-7.76 (m, 1H), 7.68 (d, *J* = 8.4 Hz, 1H), 7.64-7.59 (m, 1H), 7.46-7.41 (m, 2H), 7.36-7.29 (m, 9H), 7.23-7.05 (m, 46H), 6.94-6.89 (m, 4H), 6.87-6.81 (m, 3H), 5.72 (d, *J* = 3.9 Hz, 1H), 5.63 (d, *J* = 3.5 Hz, 1H), 5.58 (d, *J* = 3.9 Hz, 1H), 5.28 (d, *J* = 3.6 Hz, 0.61H), 4.97 (d, *J* = 11.1 Hz, 1H), 4.89 (t, *J* = 11.2 Hz, 1H), 4.80 (d, *J* = 12.7 Hz, 2H), 4.74-4.67 (m, 4H), 4.63 (d, *J* = 12.0 Hz, 1H), 4.60 (d, *J* = 11.1 Hz, 1H), 4.55-4.08 (m, 24 H), 4.04-3.96 (m, 2H), 3.84-3.24 (m, 19H) ppm; ^{13}C NMR (100 MHz, $CDCl_3$): δ 138.9, 138.7, 138.5, 138.3, 138.2, 138.1, 138.0, 137.89, 137.85, 137.82, 137.3, 136.3, 133.3, 132.9, 128.79, 128.76, 128.6, 128.59, 128.54, 128.51, 128.44, 128.41, 128.36, 128.31, 128.29, 128.26, 128.22, 128.20, 128.16, 128.12, 128.09, 128.02, 127.97, 127.90, 127.87, 127.82, 127.78, 127.71, 127.66, 127.63, 127.5, 127.44, 127.41, 127.2, 127.0, 126.9, 126.58, 126.54, 126.51, 126.1, 125.9, 125.7, 97.8, 97.3, 96.3, 96.2, 90.6, 82.2, 79.4, 79.3, 79.0, 78.9, 78.7, 78.1, 78.0, 77.3, 75.6, 75.5, 75.4, 74.6, 74.4, 73.6, 73.58, 73.50, 73.4, 73.3, 73.2, 73.1, 72.9, 69.8, 69.77, 69.73, 69.5, 68.5, 29.8 ppm; ESI-HRMS: m/z $[M+Na]^+$ calcd. for $C_{119}H_{122}NaO_{21}$: 1910.8443; found 1910.8416.

2,4,6-tri-*O*-benzyl-3-*O*-(2-naphthylmethyl)-α-D-glucopyranosyl-(1→3)-2,4,6-tri-*O*-benzyl-α-D-glucopyranosyl-(1→3)-2,4,6-tri-*O*-benzyl-α-D-glucopyranosyl-(1→3)-2,4,6-tri-*O*-benzyl-α/β-D-glucopyranosyl-1-(*N*-phenyl)-2,2,2-trifluoroacetimidate (24): To a solution of tetrasaccharide hemiacetal **23** (51.0 mg, 27.0 µmol) and Cs_2CO_3 (17.6 mg, 54.0 µmol) in acetone (1.0 mL), 2,2,2-trifluoro-*N*-phenylacetimidoyl chloride (8.8 µL, 54.0 µmol) was added at 23 °C and the reaction was stirred under nitrogen atmosphere for 17 h. Then, the reaction solution was filtered through a bed of Celite and concentrated under reduced pressure. The crude product was purified by silica gel column

chromatography (EtOAc/hexane = 1:9 – 1:4) and compound **24** (41.7 mg, 75% yield, α/β = 5.5:4.5) was obtained as a white foam. R_f = 0.44 (Tol/EtOAc = 20:1); ^{1}H NMR (600 MHz; CD_2Cl_2): δ 7.78-7.74 (m, 1 H), 7.69-7.61 (m, 3 H), 7.43-7.39 (m, 2 H), 7.34-7.89 (m, 65 H), 6.77 (d, *J* = 7.3 Hz, 1 H), 6.65 (bs, 1 H), 5.67 (d, *J* = 3.8 Hz, 0.45 H), 5.66 (d, *J* = 3.7 Hz, 0.55 H), 5.64 (d, *J* = 3.7 Hz, 0.55 H), 5.58 (d, *J* = 3.4 Hz, 0.45 H), 5.50 (d, *J* = 3.7 Hz, 0.55 H), 5.48 (d, *J* = 3.3 Hz, 0.45 H), 4.96-4.87 (m, 2 H), 4.85-4.66 (m, 7 H), 4.64-4.58 (m, 2 H), 4.55-4.11 (m, 24 H), 4.01-3.95 (m, 1 H), 3.81 (t, *J* = 10.1 Hz, 1 H), 3.76-3.56 (m, 7 H), 3.50-3.43 (m, 5 H), 3.37-3.27 (m, 4 H) ppm; ^{13}C NMR (100 MHz, $CDCl_3$): δ 143.8, 139.0, 138.8, 138.7, 138.6, 138.5, 138.4, 138.3, 138.2, 138.1, 138.0, 137.9, 136.7, 133.4, 132.9, 128.8, 128.6, 128.4, 128.3, 128.2, 128.1, 128.0, 127.9, 127.8, 127.7, 127.6, 127.5, 127.4, 127.3, 127.2, 127.1, 127.0, 126.9, 126.2, 126.1, 126.0, 125.7 (89 C), 119.4, 119.3, 97.1, 96.5, 96.1, 96.0, 82., 80.1, 79.8, 79.4, 79.3, 79.2, 79.1, 78.4, 78.1, 77.7, 75.7, 75.3, 75.2, 74.7, 74.0, 73.9, 73.6, 73.5, 73.4, 73.3, 73.2, 73.1, 72.7, 70.2, 70.1, 70.0, 69.9, 69.0, 68.9, 68.8, 68.5; ESI-HRMS: m/z [M hydrolyzed + NH_4]$^+$ calcd. for $C_{119}H_{126}NO_{21}$: 1905.8851; found 1905.8873.

5-Aminopentyl-*N*-(Benzyl)benzyloxycarbonyl 2,4,6-tri-*O*-benzyl-3-*O*-(2-naphthylmethyl)-α-D-glucopyranoside (25): A mixture of donor **16**[33] (0.52 g, 0.68 mmol) and Cbz/Benzyl protected 5-amino pentanol linker[37] (0.16 g, 0.48 mmol) was co-evaporated with toluene. Ph_3PO (1.14 g, 4.09 mmol) and anhydrous DCM (10.7 mL) were added to the flask. The solution was stirred over fresh flame-dried MS 4 Å (0.68 g, powdered) under nitrogen atmosphere for 20 minutes. TMSI (0.10 mL, 0.15 mmol) was added dropwise to the reaction mixture. The reaction was stirred at room temperature (23 °C) till completion as indicated by TLC analysis. The reaction was quenched by adding aq. satd. $Na_2S_2O_3$. The organic phase was washed with brine, dried over $MgSO_4$, filtered and concentrated under reduced pressure. The product was purified by silica gel column chromatography (EtOAc/hexane = 1:5 – 1:4) and compound **25** (0.36 g, 81% yield, α/β = 10:1) was obtained as a yellowish oil. R_f = 0.2 (Hex/EtOAc = 5/1); ^{1}H NMR (400 MHz, $CDCl_3$): δ 7.84-7.75 (m, 4H), 7.48-7.45 (m, 3H), 7.36-7.26 (m, 19H), 7.25-7.16 (m, 5H), 7.12-7.10 (m, 2H), 5.18 (d, *J* = 11.8 Hz, 2H), 5.15 (d, *J* = 11.1 Hz, 1H), 4.97 (d, *J* = 11.0 Hz, 1H), 4.85 (d, *J* = 10.5 Hz, 1H), 4.79 (d, *J* = 12.1 Hz, 1H), 4.76 (d, *J* = 3.1 Hz, 1H), 4.67 (d, *J* = 11.9 Hz, 1H), 4.62 (d, *J* = 12.1 Hz, 1H), 4.51-4.46 (m, 4H), 4.38 (d, *J* = 8.4 Hz, 0.1H), 4.04 (t, *J* = 9.2 Hz, 1H), 3.78-3.70 (m, 2H), 3.67 (t, *J* = 9.5 Hz, 1H), 3.64-3.58 (m, 2H), 3.41-3.33 (m, 1H), 3.29-3.25 (m, 1H), 3.21-3.17 (m, 1H), 1.65-1.48 (m, 4H), 1.38-1.29 (m, 2H) ppm; ^{13}C NMR (100 MHz, $CDCl_3$): δ 156.8, 156.2, 138.39, 138.30, 137.9, 136.5, 133.4, 133.0, 128.66, 128.60, 128.5, 128.4, 128.19, 128.15, 128.07, 128.05, 127.98, 127.95, 127.92, 127.8, 127.7, 127.3, 126.6, 126.5, 126.18, 126.10, 125.8, 103.7, 97.0, 84.7, 82.2, 80.1, 77.8, 77.4, 77.3, 77.1, 76.8, 75.8, 75.2, 73.6, 73.2, 70.2, 68.5, 68.1, 67.2, 60.5, 50.6, 50.3, 47.2, 46.2, 29.2, 28.1, 27.6, 23.6 ppm. ESI-HRMS: m/z [M+Na]$^+$ calcd. for $C_{58}H_{61}NNaO_8$: 923.4361; found 923.4323.

Benzylbenzyloxycarbonylaminopentyl 2,4,6-tri-*O*-benzyl-α-D-glucopyranoside (26): To the solution of compound **25** (0.33 g, 0.36 mmol) in DCM/H_2O (3.7 mL, 10:1) at 0 °C, DDQ (91.6 mg, 0.40 mmol) was added. After 10 minutes, the reaction was warmed to 23 °C and stirred till completion. The reaction was quenched by adding aq. $NaHCO_3$ (2 mL) and diluted with DCM. The organic layer was washed with brine, dried over $MgSO_4$, filtered and concentrated under reduced pressure. The product was purified by silica gel column chromatography (EtOAc/hexane = 1:4 – 1:3) to yield compound **26** (0.23 g, 82% yield) as colorless oil. R_f = 0.2 (hexane/EtOAc = 3/1); ^{1}H NMR (400 MHz, $CDCl_3$): δ 7.29-7.13 (m, 24H), 7.09 (d, *J* = 7.2 Hz, 1H), 5.10 (d, *J* = 10.6 Hz, 2H), 4.78 (d, *J* = 11.0 Hz, 1H), 4.67 (d, *J* = 3.4 Hz, 1H), 4.61 (d, *J* = 12.0 Hz, 1H), 4.55 (d, *J* = 12.2 Hz, 2H,), 4.44 (d, *J* = 10.9 Hz, 1H), 4.41 (d, *J* = 11.8 Hz, 3H), 4.00 (t, *J* = 9.1 Hz, 1H), 3.66-3.62 (m, 2H), 3.56-3.46 (m, 3H), 3.33 (d, *J* = 9.6, 3.4 Hz, 1H), 3.22-3.15 (m, 2H), 3.12-3.08 (m, 1H), 1.53-1.40 (m, 4H), 1.27-1.20 (m, 2H) ppm; ^{13}C NMR (100 MHz, $CDCl_3$): δ 156.8, 156.2, 138.4, 138.1, 137.9, 128.6, 128.57, 128.50, 128.4, 128.1, 128.07, 128.01,

127.9, 127.88, 127.83, 127.4, 127.3, 127.2, 96.4, 79.6, 77.4, 77.3, 77.1, 76.8, 74.77, 74.74, 73.5, 72.9, 69.8, 68.5, 68.0, 67.2, 50.5, 50.2, 47.2, 46.2, 29.8, 29.2, 28.0, 27.6, 23.5 ppm; ESI-HRMS: m/z $[M+Na]^+$ calcd. for $C_{47}H_{53}NNaO_8$: 783.3735; found 783.3702.

Benzylbenzyloxycarbonylaminopentyl 2,4,6-tri-*O*-benzyl-3-*O*-(2-naphthylmethyl)-α-D-glucopyranosyl-(1→3)-2,4,6-tri-*O*-benzyl-α-D-glucopyranosyl-(1→3)-2,4,6-tri-*O*-benzyl-α-D-glucopyranoside (27): A mixture of disaccharide donor **20** (0.18 g, 0.15 mmol) and the acceptor **26** (80.3 mg, 0.10 mmol) was co-evaporated with toluene. Anhydrous DCM (2.5 mL) and anhydrous DMF (0.19 mL, 2.40 mmol) were added to the flask and the solution was stirred over fresh flame-dried MS 4 Å (0.25 g, powdered) under nitrogen atmosphere for 20 minutes. The reaction flask was cooled to -78 °C and triflic acid (13.6 µL, 0.15 mmol) was added dropwise. After 45 minutes, the reaction solution was allowed to warm to 0 °C and stirred for 16 h at that temperature. Then, it was quenched by adding NEt_3 (25.0 µL, 0.18 mmol). The reaction solution was filtered through a bed of Celite and concentrated under reduced pressure. The product was purified by silica gel column chromatography (EtOAc/toluene = 1:50 – 1:12) and compound **27** (0.15 g, 82% yield) was obtained as a colorless viscous liquid. R_f = 0.35 (EtOAc/toluene = 1:9); ^1H NMR (400 MHz, $CDCl_3$): δ 7.79-7.76 (m, 1H), 7.69 (d, *J* = 8.4 Hz, 1H), 7.65-7.62 (m, 2H), 7.45-7.41 (m, 2H), 7.34-7.28 (m, 14H), 7.24-7.11 (m, 34H), 7.09-7.04 (m, 3H), 6.98-6.94 (m, 4H,), 6.89-6.86 (m, 2H), 5.68 (d, *J* = 3.5 Hz, 1H), 5.64 (d, *J* = 3.5 Hz, 1H), 5.17 (d, *J* = 10.4 Hz, 2H), 5.01 (d, *J* = 11.2 Hz, 1H), 4.94 (d, *J* = 11.1 Hz, 1H), 4.89 (d, *J* = 12.0 Hz, 1H), 4.80 (d, *J* = 11.6 Hz, 1H), 4.74 (d, *J* = 3.9 Hz, 1H), 4.73 (d, *J* = 11.1 Hz, 1H), 4.69 (d, *J* = 11.1 Hz, 1H), 4.68 (d, *J* = 11.9 Hz, 1H), 4.60-4.52 (m, 5H), 4.51-4.46 (m, 4H), 4.44-4.38 (m, 4H), 4.37-4.20 (m, 7H), 4.09-4.04 (m, 2H), 3.86-3.82 (m, 1H), 3.75-3.73 (m, 2H), 3.68-3.43 (m, 10H), 3.26-3.15 (m, 5H), 1.59-1.48 (m, 4H), 1.31-1.28 (m, 2H) ppm; ^{13}C NMR (100 MHz, $CDCl_3$): δ 138.9, 138.7, 138.4, 138.24, 138.22, 138.1, 138.0, 137.8, 136.3, 133.3, 132.9, 129.1, 128.66, 128.63, 128.60, 128.5, 128.44, 128.40, 128.36, 128.33, 128.30, 128.28, 128.23, 128.20, 128.17, 128.14, 128.07, 128.05, 128.03, 127.98, 127.95, 127.89, 127.85, 127.7, 127.6, 127.5, 127.4, 127.3, 127.1, 126.6, 126.5, 126.1, 126.0, 125.9, 125.7, 125.3, 97.4, 96.39, 96.30, 82.2, 79.5, 79.2, 79.0, 78.9, 78.5, 78.0, 77.4, 77.3, 77.1, 76.8, 75.8, 75.5, 74.7, 73.6, 73.4, 73.2, 73.0, 69.9, 69.2, 68.6, 68.5, 68.3, 68.0, 67.2, 50.3, 29.8, 29.2, 28.0, 27.6, 23.5, 21.6 ppm; ESI-HRMS: m/z $[M+Na]^+$ calcd. for $C_{112}H_{117}NNaO_{18}$: 1787.8235; found 1787.8208.

Benzylbenzyloxycarbonylaminopentyl 2,4,6-tri-*O*-benzyl-α-D-glucopyranosyl-(1→3)-2,4,6-tri-*O*-benzyl-α-D-glucopyranosyl-(1→3)-2,4,6-tri-*O*-benzyl-α-D-glucopyranoside (28): To the solution of compound **27** (129 mg, 73.1 µmol) in DCM/H_2O (1.10 mL, 10:1) at 0 °C, DDQ (18.3 mg, 80.4 µmol) was added. After 10 minutes, the reaction was allowed to warm to 23 °C and stirred for 3 h more. The reaction was quenched by adding aq. satd. $NaHCO_3$ (1 mL) and diluted with DCM. The organic layer was washed with brine, dried over $MgSO_4$, filtered and concentrated under reduced pressure. The product was purified by silica gel column chromatography (EtOAc/hexane = 1:4 – 2:5) and compound **28** (84.3 mg, 71% yield), was obtained as a white foam. R_f = 0.25 (EtOAc/hexane = 3:7); ^1H NMR (400 MHz, $CDCl_3$): δ 7.32-7.27 (m, 7H), 7.24-7.04 (m, 42H), 6.99-6.97 (m, 4H), 6.83 (d, *J* = 7.0 Hz, 2H), 5.64 (d, *J* = 3.5 Hz, 1H), 5.59 (d, *J* = 3.6 Hz, 1H), 5.14 (d, *J* = 10.0 Hz, 2H), 4.80 (d, *J* = 11.9 Hz, 1H), 4.76-4.71 (m, 2H), 4.65 (d, *J* = 11.2 Hz, 1H), 4.58-4.50 (m, 5H), 4.47-4.18 (m, 15H), 4.13-4.02 (m, 3H), 3.78 (t, *J* = 9.5 Hz, 1H), 3.71 (br. d, *J* = 5.2 Hz, 1H), 3.59-3.38 (m, 8H), 3.33 (dd, *J* = 9.8, 3.5 Hz, 1H), 3.25-3.12 (m, 5H), 1.58-1.44 (m, 4H), 1.29-1.23 (m, 2H) ppm; ^{13}C NMR (100 MHz, $CDCl_3$): δ 138.8, 138.6, 138.2, 138.0, 137.8, 128.6, 128.59, 128.55, 128.50, 128.47, 128.45, 128.39, 128.34, 128.28, 128.24, 128.20, 128.15, 128.13, 128.0, 127.95, 127.91, 127.89, 127.86, 127.78, 127.70, 127.6, 127.44, 127.40, 127.29, 127.20, 127.0, 126.5, 96.9, 96.36, 96.31, 79.2, 79.1, 78.9, 78.5, 77.8, 77.3, 75.7, 74.2, 73.6, 73.4, 73.3, 73.1, 72.9, 69.9, 69.4, 69.2, 68.5, 68.4, 68.2, 67.2, 50.6, 50.3, 47.2, 46.2, 29.2, 28.0, 27.6, 23.5 ppm; ESI-HRMS: m/z $[M+Na]^+$ calcd. for $C_{101}H_{109}NNaO_{18}$: 1647.7609; found 1647.7588.

Benzylbenzyloxycarbonylaminopentyl 2,4,6-tri-*O*-benzyl-3-*O*-(2-naphthylmethyl)-α-D-glucopyranosyl-(1→3)-2,4,6-tri-*O*-benzyl-α-D-glucopyranosyl-(1→3)-2,4,6-tri-*O*-benzyl-α-D-glucopyranosyl-(1→3)-2,4,6-tri-*O*-benzyl-α-D-glucopyranosyl-(1→3)-2,4,6-tri-*O*-benzyl-α-D-glucopyranoside (29): A mixture of disaccharide donor **20** (30.0 mg, 25.1 µmol) and the trisaccharide acceptor **28** (28.6 mg, 17.6 µmol) was co-evaporated with toluene. Anhydrous DCM (0.6 mL) and anhydrous DMF (31.1 µL, 0.40 mmol) were added to the flask and the solution was stirred over fresh flame-dried MS 4 Å (60.0 mg, powdered) under nitrogen atmosphere for 20 minutes. The reaction flask was cooled to -78 °C and triflic acid (2.3 µL, 25.1 µmol) was added dropwise. After 45 minutes, the reaction solution was allowed to warm to 0 °C and stirred at that temperature for 9 h more. The reaction was quenched by adding NEt_3 (4.3 µL, 31.0 µmol), filtered through a bed of Celite and concentrated under reduced pressure. The product was purified by silica gel column chromatography (EtOAc/toluene = 1:50 – 1:12) and compound **29** (41.7 mg, 90% yield) was obtained as a colorless viscous liquid. R_f = 0.5 (EtOAc/toluene = 1:9); ^{1}H NMR (400 MHz, $CDCl_3$): δ 7.81-7.79 (m, 1H), 7.70 (d, *J* = 8.3 Hz, 2H), 7.67-7.61 (m, 2H), 7.48-7.28 (m, 32H), 7.21-7.08 (m, 45H), 6.93-6.91 (m, 2H), 6.89 (d, *J* = 7.4 Hz, 2H), 6.86 (d, *J* = 6.9 Hz, 2H), 6.80 (d, *J* = 7.2 Hz, 2H), 6.68 (d, *J* = 7.3 Hz, 2H), 5.72 (d, *J* = 3.7 Hz, 1H), 5.69 (d, *J* = 3.6 Hz, 1H), 5.65 (d, *J* = 3.6 Hz, 1H), 5.60 (d, *J* = 3.5 Hz, 1H), 5.20 (d, *J* = 9.7 Hz, 2H), 4.98 (d, *J* = 11.1 Hz, 1H), 4.90 (d, *J* = 11.1 Hz, 1H), 4.81-4.66 (m, 9H), 4.64-4.10 (m, 46H), 4.00 (t, *J* = 9.4 Hz, 1H), 3.86-3.75 (m, 6H), 3.67-3.18 (m, 26H), 1.64-1.52 (m, 4H), 1.38-1.32 (m, 2H) ppm; ^{13}C NMR (100 MHz, $CDCl_3$): δ 138.9, 138.7, 138.5, 138.3, 138.2, 138.19, 138.12, 138.10, 138.0, 137.96, 137.90, 136.38, 133.36, 132.9, 128.66, 128.60, 128.55, 128.50, 128.45, 128.40, 128.35, 128.33, 128.30, 128.27, 128.24, 128.22, 128.16, 128.13, 128.08, 128.06, 128.02, 127.98, 127.95, 127.91, 127.8, 127.77, 127.72, 127.70, 127.6, 127.5, 127.44, 127.40, 127.37, 127.31, 127.2, 127.1, 126.8, 126.55, 126.52, 126.3, 126.2, 126.1, 125.9, 125.7, 97.3, 96.3, 96.1, 96.0, 82.1, 79.4, 79.2, 79.1, 78.9, 78.8, 78.59, 78.54, 78.4, 78.0, 77.4, 77.3, 77.1, 76.8, 75.6, 75.5, 75.4, 75.1, 74.5, 73.6, 73.5, 73.3, 73.1, 72.9, 72.6, 72.5, 69.7, 69.5, 69.3, 69.2, 68.4, 68.3, 68.0, 67.2, 50.6, 50.3, 47.2, 46.2, 32.0, 29.8, 29.4, 29.2, 28.0, 27.6, 23.5, 22.8, 14.2 ppm; ESI-HRMS: m/z $[M+Na]^+$ calcd. for $C_{166}H_{173}NNaO_{28}$: 2651.2036; found 2651.2380.

Benzylbenzyloxycarbonylaminopentyl 2,4,6-tri-*O*-benzyl-3-*O*-(2-naphthylmethyl)-α-D-glucopyranosyl-(1→3)-2,4,6-tri-*O*-benzyl-α-D-glucopyranosyl-(1→3)-2,4,6-tri-*O*-benzyl-α-D-glucopyranosyl-(1→3)-2,4,6-tri-*O*-benzyl-α-D-glucopyranosyl-(1→3)-2,4,6-tri-*O*-benzyl-α-D-glucopyranosyl-(1→3)-2,4,6-tri-*O*-benzyl-α-D-glucopyranosyl-(1→3)-2,4,6-tri-*O*-benzyl-α-D-glucopyranoside (30): A mixture of tetrasaccharide donor **24** (35.0 mg, 17.0 µmol) and the trisaccharide acceptor **28** (19.3 mg, 11.9 µmol) was co-evaporated with toluene. Anhydrous DCM (0.5 mL) and anhydrous DMF (21.0 µL, 0.27 mmol) were added to the flask and the solution was stirred over fresh flame-dried MS 4 Å (50.0 mg, powdered) under nitrogen atmosphere for 20 minutes. The reaction flask was cooled to -78 °C and triflic acid (1.5 µL, 17.0 µmol) was added dropwise. After 45 minutes, the reaction solution was allowed to warm to 0 °C and stirring was continued for 9 h at this temperature. The reaction was quenched by adding NEt_3 (2.8 µL, 20.0 µmol), filtered through a bed of Celite and concentrated under reduced pressure. The product was purified by silica gel column chromatography (EtOAc/toluene = 1:50 – 1:12) and compound **30** (29.5 mg, 71% yield) was obtained as a colorless viscous liquid. R_f = 0.3 (EtOAc/toluene = 1:9); ^{1}H NMR (400 MHz, $CDCl_3$): δ 7.81-7.79 (m, 1H), 7.70 (d, *J* = 8.4 Hz, 1H), 7.67-7.60 (m, 2H), 7.48-7.43 (m, 3H), 7.40-7.30 (m, 25H), 7.22-7.07 (m, 77H), 6.95-6.85 (m, 7H), 6.78 (d, *J* = 7.1 Hz, 2H), 6.69 (d, *J* = 7.5 Hz, 2H), 6.64 (d, *J* = 7.4 Hz, 1H), 6.60 (d, *J* = 7.3 Hz, 1H), 5.72 (d, *J* = 3.5 Hz, 1H), 5.66 (d, *J* = 3.9 Hz, 2H), 5.62 (d, *J* = 3.6 Hz, 2H), 5.58 (d, *J* = 3.5 Hz, 1H), 5.21 (d, *J* = 9.5 Hz, 2H), 4.97 (d, *J* = 10.9 Hz, 1H), 4.89 (d, *J* = 11.1 Hz, 1H), 4.83-4.76 (m, 3H), 4.73-4.09 (m, 66H), 3.99 (t, *J* = 9.3 Hz, 1H), 3.87-3.72 (m, 8H), 3.67-3.18 (m, 32H), 1.63-1.51 (m, 4H),

1.37-1.35 (m, 2H) ppm; ^{13}C NMR (175 MHz, $CDCl_3$): δ 139.0, 138.8, 138.65, 138.62, 138.4, 138.3, 138.26, 138.21, 138.18, 138.15, 138.13, 138.0, 137.99, 137.98, 136.4, 133.4, 132.9, 128.6, 128.58, 128.50, 128.46, 128.41, 128.37, 128.34, 128.29, 128.27, 128.23, 128.18, 128.15, 128.13, 128.10, 128.07, 128.05, 128.03, 128.01, 127.99, 127.97, 127.92, 127.90, 127.8, 127.76, 127.71, 127.6, 127.5, 127.47, 127.44, 127.39, 127.38, 127.36, 127.32, 127.29, 127.23, 127.19, 127.15, 126.9, 126.8, 126.7, 126.6, 126.55, 126.52, 126.46, 126.44, 126.41, 126.36, 126.30, 126.2, 126.1, 125.9, 125.7, 97.2, 96.3, 96.2, 96.1, 96.07, 96.05, 96.01, 82.2, 79.5, 79.3, 79.19, 79.13, 79.0, 78.9, 78.6, 78.5, 78.4, 78.1, 77.3, 77.1, 76.9, 75.6, 75.5, 75.4, 75.2, 75.1, 74.5, 73.6, 73.5, 73.47, 73.41, 73.3, 73.1, 72.9, 72.7, 72.6, 72.49, 72.41, 69.8, 69.6, 69.5, 69.4, 69.3, 69.25, 69.20, 68.7, 68.6, 68.4, 67.2, 29.8, 29.4, 23.5, 22.8, 14.2 ppm. ESI-HRMS: m/z $[M+2Na]^{2+}$ calcd. for $C_{220}H_{229}NNa_2O_{38}$: 1769.2901; found 1769.0386.

5-Aminopentyl α-D-glucopyranosyl-(1→3)-α-D-glucopyranosyl-(1→3)-α-D-glucopyranoside (31): The trisaccharide **27** (25.0 mg, 14.2 µmol) was dissolved in a mixture of DCM/^{t}BuOH/H_2O (2:1:1, 2.0 mL). Pd-C (25 mg; 10 w% Pd) was added and the reaction was stirred at 23 °C under H_2 atmosphere for 48 hours. The reaction solution was filtered through Celite, washed with DCM, ^{t}BuOH and H_2O. The filtrates were concentrated *in vacuo* and the crude product was purified by reversed phase HPLC using a Synergy column to obtain the fully deprotected trisaccharide **31** (2.00 mg, 3.39 µmol, 24% yield). ^{1}H NMR (600 MHz, D_2O): δ 5.36 (d, J = 3.9 Hz, 1H, H-1^B), 5.35 (d, J = 3.9 Hz, 1H, H-1^C), 4.91 (d, J = 3.7 Hz, 1H, H-1^A), 4.03-3.98 (m, 2H, H-5^B/H-5^C), 3.88 (t, J = 9.4 Hz, 1H, H-3^A), 3.86-3.81 (m, 4H, H-3^B/H-6a^A/H-6a^B/H-6a^C), 3.78-3.71 (m, 5H, H-6b^A-H-6b^C/H-3^C/O*CH$_a$*CH_2), 3.68-3.63 (m, 5H, H-2^A/H-2^B/H-4^A/H-4^B/H-5^A), 3.57-3.51 (m, 2 H, H-2^C/O*CH$_b$*CH_2), 3.42 (t, J = 9.8 Hz, 1H, H-4^C), 2.95 (t, J = 7.2 Hz, 2H, CH_2NH_2), 1.67-1.59 (m, 4H, CH_2 Linker), 1.45-1.39 (m, 2H, CH_2 Linker) ppm; ^{13}C NMR (150 MHz, D_2O): δ 99.9 (2 C, C-1^B/C-1^C), 98.9 (C-1^A), 80.7, 80.6 (C-3^A/C-3^B), 73.6 (C-3^C), 72.5 (C-2^C), 72.4 (C-2^A), 72.3 (2 C, C-5^B/C-5^C), 71.0, 70.7, 70.7 (4 C, C-2^B/C-4^A/C-4^B/C-5^A), 70.2 (C-4^C), 68.5 (O*CH$_2$*CH_2), 61.2, 61.1, 61.0 (C-6), 40.2 (CH_2NH_2), 28.8 (2 C), 23.2 (CH_2 Linker) ppm; ESI-HRMS: m/z $[M+H]^+$ calcd. for $C_{23}H_{44}NO_{16}$: 590.2654; found 590.2668.

Aminopentyl α-D-glucopyranosyl-(1→3)-α-D-glucopyranosyl-(1→3)-α-D-glucopyranosyl-(1→3)-α-D-glucopyranosyl-(1→3)-α-D-glucopyranoside (32): The pentasaccharide **29** (12.0 mg, 4.6 µmol) was dissolved in ^{t}BuOH (1.2 mL) followed by the addition of water (0.6 mL) and AcOH (0.15 mL). Pd-C (10 w%; 12 mg) was added and the reaction was stirred at 23 °C under H_2 atmosphere for 5 days. The reaction solution was filtered through Celite, washed with MeOH/H_2O (1/1) and the solvent was removed *in vacuo*. The crude product was purified by HPLC using a ZIC-HILIC column (MeCN/ 20 mM aq. NH_4COO = 90/10 → 30/70) to obtain the fully deprotected pentasaccharide **32** (1.9 mg, 46% yield). ^{1}H NMR (600 MHz, D_2O): δ 5.38-5.33 (m, 4H, H-1^{B-E}), 4.90 (d, J = 4.1 Hz, 1H, H-1^A), 4.04-3.97 (m, 4H, H-5^{B-E}), 3.91-3.86 (m, 4 H, H-3^{A-D}), 3.83-3.80 (m, 5 H, H-6a^{A-E}), 3.78-3.70 (m, 7 H, H-6b^{A-E}/H-3^E/O*CH$_a$*CH_2), 3.70-3.63 (m, 9 H, H-2^{A-D}/H-4^{A-D}/H-5^A), 3.57-3.52 (m, 2 H, H-2^E/O*CH$_b$*CH_2), 3.42 (t, J = 10.0 Hz, 1H, H-4^E), 3.0 (t, J = 7.3 Hz, 2H, CH_2NH_2), 1.72-1.62 (m, 4H, CH_2 Linker), 1.52-1.39 (m, 2H, CH_2 Linker) ppm; ^{13}C NMR (150 MHz, D_2O): δ 100.1-99.9 (4 C, C-1^{B-E}), 98.9 (C-1^A), 80.7 (4 C, C-3^{A-D}), 73.6 (C-3^E), 72.6 (C-2^E), 72.5 (C-2^A), 72.3 (4 C, C-5^{B-E}), 71.1, 71.0, 70.7, 70.6 (9 C, C-2^{B-D}/C-4^{A-D}/C-5^A), 70.2 (C-4^E), 68.5 (O*CH$_2$*CH_2), 61.3, 61.2, 61.1, 61.0 (5 C, C-6), 40.1 (CH_2NH_2), 28.8, 27.3, 23.2 (CH_2 Linker) ppm; ESI-HRMS: m/z $[M+H]^+$ calcd. for $C_{35}H_{63}NO_{26}$: 914.3710; found 914.3729.

Aminopentyl α-D-glucopyranosyl-(1→3)-α-D-glucopyranosyl-(1→3)- α-D-glucopyranosyl-(1→3)-α-D-glucopyranosyl-(1→3)-α-D-glucopyranosyl-(1→3)-α-D-glucopyranosyl-(1→3)-α-D-glucopyranoside (33): The heptasaccharide **30** (20.4 mg, 5.84 µmol) was dissolved in a mixture of DCM/^{t}BuOH/H_2O (2:1:1, 2.0 mL). Pd-C (20 mg; 10 w% Pd) was added and the reaction was stirred at 23 °C under H_2 atmosphere for 3 days. Then, the reaction solution was filtered through Celite, washed

with DCM, ^{t}BuOH and H_2O and the filtrates were concentrated *in vacuo.* The crude product was purified by Sephadex G25 column to obtain the fully deprotected heptasaccharide **33** (5.12 mg, 4.13 µmol, 70% yield); ^{1}H NMR (600 MHz, D_2O): δ 5.34-5.31 (m, 6H, H-1^{B-G}), 4.87 (d, *J* = 3.7 Hz, 1H, H-1^A), 4.02-3.96 (m, 6H, H-5^{B-G}), 3.88-3.83 (m, 6 H, H-3^{A-F}), 3.83-3.77 (m, 7 H, H-$6a^{A-G}$), 3.78-3.71 (m, 9 H, H-$6b^{A-G}$/H-3^G/O$CH_a$$CH_2$), 3.68-3.63 (m, 13 H, H-2^{A-F}/H-4^{A-F}/H-5^A), 3.55-3.48 (m, 2 H, H-2^G/O$CH_b$$CH_2$), 3.39 (t, *J* = 9.8 Hz, 1H, H-4^G), 2.97 (t, *J* = 7.2 Hz, 2H, CH_2NH_2), 1.67-1.59 (m, 4H, CH_2 Linker), 1.45-1.39 (m, 2H, CH_2 Linker) ppm; ^{13}C NMR (150 MHz, D_2O): δ 100.1-99.9 (6 C, C-1^{B-G}), 98.9 (C-1^A), 80.7 (6 C, C-3^{A-F}), 73.6 (C-3^G), 72.5 (C-2^G), 72.4 (C-2^A), 72.3 (6 C, C-5^{B-G}), 71.1, 71.0, 70.7, 70.6 (13 C, C-2^{B-F}/C-4^{A-F}/C-5^A), 70.2 (C-4^G), 68.5 (O$CH_2$$CH_2$), 61.2, 61.1, 61.0 (C-6), 40.2 (CH_2NH_2), 28.8, 27.3, 23.2 (CH_2 Linker) ppm; ESI-HRMS: m/z $[M+H]^+$ calcd. for $C_{47}H_{83}NO_{36}$: 1238.4767; found 1238.4775.

4-Methylphenyl 2,3,6-tri-*O*-benzyl-4-*O*-(2-naphthylmethyl)-α-D-glucopyranosyl-(1→4)-2,3,6-tri-*O*-benzyl-1-thio-α-D-glucopyranoside (36): A mixture of the donor **34**[23] (390 mg, 512 µmol) and the acceptor **35** (200 mg, 358 µmol) were co-evaporated with toluene. Then, anhydrous DCM (6.0 mL) and anhydrous DMF (634 µL, 8.19 mmol) were added to the flask and the solution was stirred over fresh flame-dried MS 4 Å (600 mg, powdered) under nitrogen atmosphere for 20 minutes. The reaction flask was cooled to -78 °C and triflic acid (46.0 µL, 512 µmol) was added dropwise. After 45 minutes, the reaction solution was allowed to warm up to 0 °C, was stirred at that temperature for 38 h and was then quenched by adding NEt_3 (71.0 µL, 512 µmol). The reaction solution was filtered through a bed of Celite and concentrated under reduced pressure. The product was purified by silica gel column chromatography (EtOAc/ hexane = 1:25 – 1:10) and compound **36** (342 mg, 303 µmol, 84% yield) was obtained as a viscous yellow liquid. R_f = 0.4 (Hex/EtOAc = 5/1); ^{1}H NMR (400 MHz, $CDCl_3$): δ 7.81-7.79 (m, 1H), 7.74-7.70 (m, 2H), 7.51-7.43 (m, 5H), 7.30-7.26 (m, 13H), 7.25-7.12 (m, 18H), 7.04 (d, *J* = 7.8 Hz, 2H), 5.66 (d, *J* = 3.6 Hz, 1H), 4.93-4.78 (m, 7H), 4.62-4.61 (m, 1H), 4.60 (d, *J* = 7.2 Hz, 1H), 4.56-4.44 (m, 7H), 4.26 (d, *J* = 12.1 Hz, 1H), 4.10 (t, *J* = 9.2 Hz, 1H), 3.93 (t, *J* = 9.2 Hz, 1H), 3.87 (dd, *J* = 11.2, 3.9 Hz, 1H), 3.82-3.68 (m, 5H), 3.59-3.49 (m, 4H), 3.43-3.39 (m, 1H), 2.31 (s, 3H) ppm; ^{13}C NMR (100 MHz, $CDCl_3$): δ 138.8, 138.7, 138.6, 138.0, 137.98, 137.96, 137.8, 135.9, 133.3, 133.0, 132.9, 129.8, 129.5, 128.57, 128.52, 128.48, 128.45, 128.42, 128.39, 128.37, 128.29, 128.25, 128.22, 128.1, 128.08, 128.00, 127.9, 127.86, 127.80, 127.7, 127.68, 127.64, 127.56, 127.53, 127.3, 126.6, 126.5, 126.17, 126.15, 125.9, 97.1, 87.4, 86.9, 82.2, 80.9, 79.3, 78.8, 77.7, 77.4, 77.3, 77.1, 76.8, 75.6, 75.3, 75.1, 74.4, 73.6, 73.5, 73.3, 72.4, 71.1, 69.2, 68.1, 21.2 ppm; ESI-HRMS: m/z $[M+Na]^+$ calcd. for $C_{72}H_{72}NaO_{10}S$: 1152.4810; found 1152.4750.

2,3,6-tri-*O*-benzyl-4-*O*-(2-naphthylmethyl)-α-D-glucopyranosyl-(1→4)-2,3,6-tri-*O*-benzyl-α/β-D-glucopyranoside (37): To a solution of disaccharide **36** (340 mg, 301 µmol) in THF/H_2O (4.25 mL, 4/1) *N*-bromosuccinimide (161 mg, 903 µmol) was added at 0 °C. After 10 minutes, the reaction was warmed to 23 °C and was stirred for 2 h more. The reaction was quenched by adding aq. satd. $NaHCO_3$ and diluted with EtOAc. The organic layer was washed with $NaHCO_3$ solution and brine, dried over $MgSO_4$, filtered, and concentrated under reduced pressure. The product was purified by silica gel column chromatography (EtOAc/ hexane = 1/4 – 3/7) and compound **37** (266 mg, 260 µmol, 86% yield, α/β = 2/1) was obtained as a white foam. R_f = 0.2 (EtOAc/hexane = 1/2); ^{1}H NMR (400 MHz, $CDCl_3$): δ 7.83-7.81 (m, 1H), 7.76-7.71 (m, 2H), 7.53-7.46 (m, 3H), 7.31-7.27 (m, 14H), 7.25-7.16 (m, 17H), 5.68 (d, *J* = 3.7 Hz, 1H), 5.23 (d, *J* = 3.4 Hz, 0.72H), 5.00-4.80 (m, 5H), 4.78 (d, *J* = 6.4 Hz, 0.38H), 4.69-4.46 (m, 8H), 4.28 (d, *J* = 12.4 Hz, 1H), 4.17-3.40 (m, 13H) ppm; ^{13}C NMR (100 MHz, $CDCl_3$): δ 138.9, 138.86, 138.82, 138.7, 138.18, 138.14, 138.0, 137.99, 137.95, 137.7, 135.97, 135.92, 133.3, 133.0, 128.6, 128.48, 128.46, 128.41, 128.39, 128.38, 128.32, 128.25, 128.22, 128.20, 128.13, 128.10, 128.0, 127.99, 127.90, 127.86, 127.83, 127.77, 127.71, 127.6, 127.5, 127.2, 126.8, 126.7, 126.66, 126.62, 126.16, 126.14, 126.0, 125.9, 97.4, 97.0, 96.9, 90.9, 84.4, 83.2, 82.1, 81.4, 80.1, 79.4, 79.3, 77.6, 77.4,

77.3, 77.1, 76.8, 75.6, 75.1, 74.6, 74.4, 74.3, 74.0, 73.6, 73.5, 73.4, 73.39, 73.32, 73.2, 72.8, 72.7, 71.2, 71.1, 69.9, 69.4, 69.1, 68.1 ppm; ESI-HRMS: m/z $[M+NH_4]^+$ calcd. for $C_{65}H_{70}NO_{11}$: 1040.4943; found 1040.4945.

2,3,6-tri-*O*-benzyl-4-*O*-(2-naphthylmethyl)-α-D-glucopyranosyl-(1→4)-2,3,6-tri-*O*-benzyl-α/β-D-glucopyranosyl-1-(*N*-phenyl)-2,2,2-trifluoroacetimidate (38): To a solution of disaccharide hemiacetal **37** (266 mg, 260 µmol) and Cs_2CO_3 (169 mg, 520 µmol) in acetone (5.3 mL), 2,2,2-trifluoro-*N*-phenylacetimidoyl chloride (84.3 µL, 520 µmol) was added at 23 °C and the reaction was stirred under nitrogen atmosphere for 2 h. The reaction solution was filtered through a bed of Celite and concentrated under reduced pressure. The product was purified by silica gel column chromatography (EtOAc/ hexane = 1:9 – 1:4) and compound **38** (290 mg, 243 µmol, 93% yield, α/β = 1/1) was obtained as a yellow viscous liquid. R_f = 0.5 (EtOAc/ hexane = 1:4); ^{1}H NMR (400 MHz, $CDCl_3$): δ 7.83-7.80 (m, 1H), 7.75-7.71 (m, 3H), 7.51-7.46 (m, 5H), 7.30-7.27 (m, 18H), 7.24-7.17 (m, 11H), 7.11-7.07 (m, 2H), 6.78 (d, *J* = 7.7 Hz, 1H), 6.71 (br. d, *J* = 7.7 Hz, 1H), 5.74 (d, *J* = 3.6 Hz, 1H), 5.62 (d, *J* = 3.6 Hz, 1H), 5.05 (d, *J* = 11.6 Hz, 1H), 4.96-4.79 (m, 8H), 4.68-4.50 (m, 12H), 4.31-4.06 (m, 5H), 3.99-3.89 (m, 3H), 3.85-3.67 (m, 8H), 3.62-3.40 (m, 5H) ppm; ^{13}C NMR (100 MHz, $CDCl_3$): δ 143.7, 138.9, 138.8, 138.6, 138.2, 138.1, 138.0, 137.99, 137.95, 137.7, 137.6, 135.9, 135.8, 133.3, 133.0, 129.5, 128.8, 128.6, 128.5, 128.49, 128.47, 128.45, 128.43, 128.40, 128.3, 128.25, 128.20, 128.14, 128.11, 128.0, 127.9, 127.89, 127.85, 127.82, 127.78, 127.74, 127.67, 127.64, 127.61, 127.5, 127.37, 127.31, 126.8, 126.7, 126.6, 126.5, 126.1, 126.0, 120.5, 119.5, 97.19, 97.11, 84.5, 82.1, 81.5, 79.5, 79.4, 79.3, 77.7, 77.6, 77.4, 77.3, 77.1, 76.8, 75.7, 75.6, 75.4, 75.2, 75.0, 74.6, 74.2, 73.5, 73.4, 73.35, 73.30, 73.2, 72.8, 71.7, 71.1, 68.5, 68.1 ppm. ESI-HRMS: m/z $[M\ hydrolyzed + NH_4]^+$ calcd. for $C_{65}H_{70}NO_{11}$: 1040.4943; found 1040.4943.

4-Methylphenyl 2,4,6-tri-*O*-benzyl-α-D-glucopyranosyl-(1→3)-2,4,6-tri-*O*-benzyl-1-thio-α-D-glucopyranoside (39): Compound **38** (96.0 mg, 85.0 µmol) was dissolved in DCM/H_2O (880 µL, 10:1) at 0 °C and DDQ (21.2 mg, 93.5 µmol) was added. After 10 minutes, the reaction was warmed to 23 °C and stirred for 2 h. Then, the reaction was quenched by adding aq. satd. $NaHCO_3$ (1 mL) and it was diluted with DCM. The organic layer was washed with brine, dried over $MgSO_4$, filtered and concentrated under reduced pressure. The product was purified by silica gel column chromatography (EtOAc/ hexane = 1:5 – 1:4) and compound **39** (66.6 mg, 67.3 µmol, 79% yield) was obtained as a white foam. R_f = 0.2 (EtOAc/ hexane = 1/4); ^{1}H NMR (400 MHz, $CDCl_3$): δ 7.51 (d, *J* = 7.8 Hz, 2H), 7.34-7.27 (m, 22H), 7.19-7.13 (m, 8H), 7.05 (d, *J* = 7.7 Hz, 2H), 5.66 (d, *J* = 3.6 Hz, 1H), 4.94 (d, *J* = 11.8 Hz, 1H), 4.89-4.84 (m, 3H), 4.71 (d, *J* = 11.2 Hz, 1H), 4.63 (d, *J* = 7.3 Hz, 1H), 4.59-4.55 (m, 4H), 4.51-4.46 (m, 2H), 4.37 (d, *J* = 12.0 Hz, 1H), 4.10 (t, *J* = 9.2 Hz, 1H), 3.89 (t, *J* = 11.1, 4.0 Hz, 1H), 3.84-3.73 (m, 4H), 3.67-3.43 (m, 7H), 2.32 (s, 3H) ppm; ^{13}C NMR (100 MHz, $CDCl_3$): δ138.7, 138.68, 138.60, 138.0, 137.9, 137.8, 132.9, 129.8, 129.6, 128.6, 128.52, 128.51, 128.48, 128.45, 128.41, 128.40, 128.0, 127.99, 127.90, 127.88, 127.84, 127.80, 127.7, 127.6, 127.56, 127.54, 127.3, 126.5, 97.0, 87.5, 86.9, 81.4, 80.9, 78.9, 78.7, 77.4, 77.3, 77.1, 76.8, 75.4, 75.3, 74.3, 73.6, 73.3, 72.3, 71.4, 70.6, 69.7, 69.1, 21.2 ppm; ESI-HRMS: m/z $[M+NH_4]^+$ calcd. for $C_{61}H_{72}NO_{10}S$: 1007.4592; found 1007.4591

4-Methyphenyl 2,3,6-tri-*O*-benzyl-4-*O*-(2-naphthylmethyl)-α-D-glucopyranosyl-(1→4)-2,3,6-tri-*O*-benzyl-α-D-glucopyranosyl-(1→4)-2,3,6-tri-*O*-benzyl-α-D-glucopyranosyl-(1→4)-2,3,6-tri-*O*-benzyl-1-thio-α-D-glucopyranoside (40): A mixture of disaccharide donor **38** (39.0 mg, 32.7 µmol) and the disaccharide acceptor **39** (25.9 mg, 26.2 µmol) was co-evaporated with toluene. Anhydrous DCM (800 µL) and anhydrous DMF (40.5 µL, 523 µmol) were added to the flask and the solution was stirred over fresh flame-dried MS 4 Å (60.0 mg, powdered) under nitrogen atmosphere for 20 minutes. The reaction flask was cooled to -78 °C and triflic acid (2.94 µL, 32.7 µmol) was added dropwise. After

45 minutes, the reaction was warmed to 0 °C and was stirred at this temperature for 40 h. Then, the reaction was quenched by adding NEt_3 (5.40 µL, 39.0 µmol) and the solution was filtered through a bed of Celite and concentrated under reduced pressure. The product was purified by silica gel column chromatography (EtOAc/ toluene = 1:99 – 1:32) and compound **40** (41.1 mg, 20.6 µmol, 78% yield) was obtained as a colorless viscous liquid. R_f = 0.35 (EtOAc/toluene = 1:19); ^{1}H NMR (400 MHz, $CDCl_3$): δ 7.83-7.81 (m, 1H), 7.74 (d, *J* = 8.4 Hz, 1H), 7.72-7.69 (m, 1H), 7.51-7.45 (m, 5H), 7.33-7.27 (m, 11H), 7.25-7.05 (m, 50H), 7.03 (d, *J* = 7.9 Hz, 2H), 5.73 (d, *J* = 3.6 Hz, 1H), 5.53 (d, *J* = 2.9 Hz, 1H), 5.52 (d, *J* = 3.0 Hz, 1H), 4.95-4.73 (m, 10H), 4.64 (d, *J* = 6.4 Hz, 1H), 4.62-4.37 (m, 16H), 4.23 (d, *J* = 12.1 Hz, 1H), 4.13-3.73 (m, 16H), 3.58-3.49 (m, 8H), 3.38 (d, *J* = 10.5 Hz, 1H), 2.31 (s, 3H) ppm; ^{13}C NMR (100 MHz, $CDCl_3$): δ 138.99, 138.94, 138.91, 138.8, 138.5, 138.4, 138.2, 138.1, 138.06, 138.00, 137.9, 137.8, 136.0, 133.3, 133.0, 132.9, 129.9, 129.8, 129.68, 128.63, 128.5, 128.46, 128.42, 128.40, 128.37, 128.34, 128.29, 128.27, 128.25, 128.08, 128.03, 128.00, 127.98, 127.92, 127.85, 127.82, 127.78, 127.76, 127.73, 127.70, 127.67, 127.60, 127.5, 127.49, 127.45, 127.3, 127.15, 127.12, 126.77, 126.73, 126.6, 126.2, 126.1, 125.9, 97.0, 96.9, 96.7, 87.5, 86.7, 82.2, 81.8, 81.5, 80.8, 79.8, 79.5, 79.2, 78.9, 77.7, 77.4, 77.3, 77.16, 77.11, 76.4, 75.6, 75.3, 75.1, 74.6, 74.3, 74.1, 73.9, 73.8, 73.5, 73.4, 73.3, 73.2, 73.1, 72.9, 72.3, 71.0, 70.97, 70.91, 69.1, 68.9, 68.8, 68.1, 21.2 ppm; ESI-HRMS: m/z $[M+NH_4]^+$ calcd. for $C_{126}H_{132}NO_{20}S$: 2011.9091; found 2011.9074.

2,3,6-Tri-*O*-benzyl-4-*O*-(2-naphthylmethyl)-α-D-glucopyranosyl-(1→4)-2,3,6-tri-*O*-benzyl-α-D-glucopyranosyl-(1→4)-2,3,6-tri-*O*-benzyl-α-D-glucopyranosyl-(1→4)-2,3,6-tri-*O*-benzyl-α/β-D-glucopyranoside (41): To a solution of **40** (26.9 mg, 13.5 µmol) in THF/H_2O (500 µL, 4/1) *N*-bromosuccinimide (7.21 mg, 40.5 µmol) was added at 0 °C. After 10 minutes, the reaction was warmed to 23 °C and stirred for 2 h. Then, the reaction was quenched by adding aqu. satd. $NaHCO_3$ and diluted with EtOAc. The organic layer was washed with aqu. satd. $NaHCO_3$ solution and brine, dried over $MgSO_4$, filtered and concentrated under reduced pressure. The product was purified by silica gel column chromatography (EtOAc/ hexane = 1:3 – 2:3) and compound **41** (18.6 mg, 9.86 µmol, 73% yield, α/β = 0.7/0.3) was obtained as a colorless viscous liquid. R_f = 0.2 (EtOAc/ hexane = 1/2); ^{1}H NMR (600 MHz, $CDCl_3$): δ 7.82-7.79 (m, 1H), 7.73 (d, *J* = 8.3 Hz, 1H), 7.71-7.68 (m, 1H), 7.49-7.45 (m, 4H), 7.29-7.27 (m, 10H), 7.25-7.06 (m, 50H), 5.71 (d, *J* = 3.5 Hz, 1H), 5.57 (d, *J* = 3.5 Hz, 0.7H), 5.54 (d, *J* = 3.5 Hz, 0.3H), 5.50 (d, *J* = 3.4 Hz, 1H, H-1α), 5.22 (d, *J* = 3.6 Hz, 0.7H), 4.96-4.66 (m, 14.3H), 4.60 (d, *J* = 11.6 Hz, 2H), 4.56-4.34 (m, 17 H), 4.21 (d, *J* = 12.1 Hz, 1H), 4.10-3.43 (m, 32H), 3.37 (d, *J* = 10.6 Hz, 1H) ppm; ^{13}C NMR (100 MHz, $CDCl_3$): δ 138.9, 138.8, 138.4, 138.1, 138.0, 137.9, 137.7, 136.0, 133.3, 133.0, 128.6, 128.5, 128.49, 128.45, 128.42, 128.39, 128.35, 128.33, 128.29, 128.24, 128.1, 128.07, 128.00, 127.9, 127.84, 127.81, 127.78, 127.76, 127.72, 127.6, 127.5, 127.4, 127.3, 127.1, 127.0, 126.9, 126.8, 126.7, 126.6, 126.2, 126.1, 125.9, 97.0, 96.8, 96.4, 91.0, 82.2, 81.9, 81.3, 79.8, 79.5, 77.4, 77.3, 77.1, 76.8, 75.6, 75.1, 74.5, 74.2, 73.5, 73.3, 73.2, 73.1, 72.9, 70.9, 70.1, 68.1, 32.0, 29.8, 29.5, 22.8, 14.2 ppm; ESI-HRMS: m/z $[M+NH_4]^+$ calcd. for $C_{119}H_{126}NO_{21}$: 1905.8851; found 1905.8847.

2,3,6-Tri-*O*-benzyl-4-*O*-(2-naphthylmethyl)-α-D-glucopyranosyl-(1→4)-2,3,6-tri-*O*-benzyl-α-D-glucopyranosyl-(1→4)-2,3,6-tri-*O*-benzyl-α-D-glucopyranosyl-(1→4)-2,3,6-tri-*O*-benzyl-α/β-D-glucopyranosyl-1-(*N*-phenyl)-2,2,2-trifluoroacetimidate (42): To a solution of tetrasaccharide hemiacetal **41** (14.0 mg, 7.41 µmol) and Cs_2CO_3 (4.83 mg, 14.8 µmol) in acetone (300 µL), 2,2,2-trifluoro-*N*-phenylacetimidoyl chloride (2.40 µL, 14.8 µmol) was added at 23 °C and the reaction was stirred under nitrogen atmosphere for 2 h. The reaction solution was filtered through a bed of Celite and concentrated under reduced pressure. The product was purified by silica gel column chromatography (EtOAc/ hexane = 1:9 – 1:4) and compound **42** (13.9 mg, 6.75 µmol, 91% yield, α/β = 0.56:0.44) was obtained as a viscous liquid. R_f = 0.5 (EtOAc/ hexane = 3:7); ^{1}H NMR (600 MHz, CD_2Cl_2): δ 7.86-7.82 (m, 1H), 7.79-7.72 (m, 2H), 7.61-7.57 (m, 1H), 7.50-7.45 (m, 2H), 7.32-7.10 (m, 66H), 6.80 (d, *J*=7.6

Hz, 1H), 6.76-6.73 (m, 1H), 5.66 (d, *J* = 3.5 Hz, 1H), 5.61 (d, J = 3.3 Hz, 0.55 H), 5.59-5.57 (m, 0.45H), 5.57 (d, *J* = 3.5 Hz, 0.45 H), 5.46 (d, *J* = 3.5 Hz, 0.55 H), 5.01-4.91 (m, 2 H), 4.91-4.80 (m, 4.5 H), 4.78-4.65 (m, 5.5 H), 4.58-4.36 (m, 13 H), 4.28 (dd, *J* = 11.9, 1.1 Hz, 1 H), 4.15-4.97 (m, 6 H), 3.97-3.87 (m, 4 H), 3.83-3.74 (m, 4 H), 3.64 (t, *J* = 9.0 Hz, 2 H), 3.59-3.51 (m, 5 H), 3.49 (ddd, *J* = 9.7, 3.5, 2.1 Hz, 1 H), 3.41 (dt, *J* = 10.8, 1.9 Hz, 1 H); ^{13}C NMR (150 MHz, CD_2Cl_2): δ 139.2, 139.0, 138.8, 138.6, 138.5, 138.4, 138.3, 138.2, 138.0, 136.4, 133.4, 133.0, 128.5, 128.4, 128.3, 128.1, 128.0, 127.9, 127.8, 127.7, 127.6, 127.5, 127.4, 127.3, 127.2, 127.1, 127.0, 126.9, 126.4, 126.1, 125.9, 119.5, 119.4, 96.8, 96.7, 96.5, 96.2, 96.0, 84.2, 82.1, 81.8, 81.7, 81.4, 79.9, 79.8, 79.6, 79.4, 77.8, 75.6, 75.4, 75.0, 74.7, 74.4, 74.2, 74.1, 73.4, 73.3, 73.2, 73.1, 73.0, 72.9, 72.8, 72.7, 72.3, 71.2, 71.1, 69.2, 69.1, 68.7, 68.5; ESI-HRMS: m/z [M hydrolyzed + NH_4]$^+$ calcd. for $C_{119}H_{126}NO_{21}$: 1905.8851; found 1905.8793.

5-Aminopentyl-*N*-(Benzyl)benzyloxycarbonyl 2,3,6-tri-*O*-benzyl-4-*O*-(2-naphthylmethyl)-α-D-glucopyranoside (43): A mixture of donor **34** (420 mg, 551 µmol) and the Cbz/Benzyl protected 5-amino pentanol linker[37] (126 mg, 386 µmol) were co-evaporated with toluene. Ph_3PO (920 mg, 3.31 mmol) and anhydrous DCM (7.0 mL) were added to the flask. The solution was stirred over fresh flame-dried MS 4 Å (540 mg, powdered) under nitrogen atmosphere for 20 minutes. Then TMSI (82.5 µL, 606 µmol) was added dropwise to the reaction mixture. The reaction was stirred at room temperature (23 °C) for 26 h and was then quenched by adding aqu. satd. $Na_2S_2O_3$. The organic phase was washed with brine, dried over $MgSO_4$, filtered and concentrated under reduced pressure. The product was purified by silica gel column chromatography (EtOAc/Hex = 1:9 – 1:5) and compound **43** (302 mg, 336 µmol, 87% yield; α/β = 16:1), was obtained as a yellow viscous liquid. R_f=0.2 (Hex/EtOAc = 4/1); ^{1}H NMR (400 MHz, $CDCl_3$): δ 7.77-7.67 (m, 3H), 7.50-7.39 (m, 3H), 7.33-7.10 (m, 26H), 5.13 (d, *J* = 11.1 Hz, 2H), 4.97 (d, *J* = 10.8 Hz, 1H), 4.92 (d, *J* = 10.9 Hz, 1H), 4.79 (d, *J* = 10.8 Hz, 1H), 4.74 (d, *J* = 12.0 Hz, 1H), 4.68 (d, *J* = 4.6 Hz, 1H), 4.60 (d, *J* = 11.8 Hz, 1H), 4.57 (d, *J* = 10.8 Hz, 1H), 4.56 (d, *J* = 12.2 Hz, 1H), 4.44 (br. d, *J* = 9.4 Hz, 2H), 4.39 (d, *J* = 12.1 Hz, 1H), 4.32 (d, *J* = 8.1 Hz, 0.06H), 3.96 (t, *J* = 9.1 Hz, 1H), 3.74-3.69 (m, 2H), 3.65 (t, *J* = 9.3 Hz, 1H), 3.58 (br. d, *J* = 10.2 Hz, 1H), 3.53 (dd, *J* = 9.6, 3.6 Hz, 1H), 3.31-3.12 (m, 3H), 1.58-1.44 (m, 4H), 1.31-1.24 (m, 2H) ppm; ^{13}C NMR (100 MHz, $CDCl_3$): δ 139.0, 138.4, 137.9, 135.7, 133.3, 133.0, 128.6, 128.59, 128.55, 128.53, 128.4, 128.2, 128.1, 128.07, 128.04, 127.98, 127.94, 127.8, 127.76, 127.70, 127.3, 126.7, 126.1, 125.9, 103.7, 97.0, 82.2, 80.1, 77.7, 77.4, 77.3, 77.1, 76.8, 75.8, 75.2, 73.6, 73.2, 70.2, 68.5, 68.1, 67.2, 29.2, 23.6 ppm; ESI-HRMS: m/z [M+Na]$^+$ calcd. for $C_{58}H_{61}NNaO_8$: 923.4361; found 923.4319.

5-Aminopentyl-*N*-(benzyl)benzyloxycarbonyl 2,3,6-tri-*O*-benzyl-α-D-glucopyranoside (44): To the solution of compound **43** (250 mg, 278 µmol) in DCM/H_2O (2.75 mL, 10:1) at 0 °C, DDQ (69.4 mg, 306 µmol) was added. After 10 minutes, the reaction was warmed to 23 °C and stirred for 2 h. The reaction was quenched by adding aq. satd. $NaHCO_3$ (2 mL) and was diluted with DCM. The organic layer was washed with brine, dried over $MgSO_4$, filtered and concentrated under reduced pressure. The product was purified by silica gel column chromatography (EtOAc/hexane = 1:4 – 1:3) and compound **44** (165 mg, 217 µmol, 78% yield, α/β = ~16:1) was obtained as a colorless oil. R_f = 0.2 (Hex/EtOAc = 3/1)**;** ^{1}H NMR (400 MHz, $CDCl_3$): δ 7.39-7.27 (m, 24H), 7.17 (d, *J* = 7.2 Hz, 1H), 5.19-5.15 (m, 2H), 5.01 (d, *J* = 11.3 Hz, 1H), 4.76-4.71 (m, 3H), 4.63 (d, *J* = 11.7 Hz, 1H), 4.59 (d, *J* = 12.2 Hz, 1H), 4.56 (d, *J* = 8.8 Hz, 1H), 4.54 (d, *J* = 12.2 Hz, 1H), 4.50 (d, *J* = 10.8 Hz, 1H), 4.37 (d, *J* = 8.6 Hz, 0.1H), 3.82-3.67 (m, 4H), 3.62 (t, *J* = 9.1 Hz, 1H), 3.53 (dd, *J* = 9.5, 3.5 Hz, 1H), 3.45-3.17 (m, 3H), 1.67-1.49 (m, 4H), 1.37-1.29 (m, 2H) ppm; ^{13}C NMR (100 MHz, $CDCl_3$): δ 138.9, 138.3, 138.1, 137.9, 128.66, 128.60, 128.55, 128.50, 128.4, 128.12, 128.10, 128.03, 128.00, 127.95, 127.90, 127.8, 127.74, 127.71, 127.4, 127.2, 103.7, 97.0, 81.7, 81.6, 79.8, 77.4, 77.3, 77.1, 76.8, 75.5, 73.7, 73.6, 73.0, 70.9, 70.1, 69.5, 68.0, 67.2, 50.5, 50.3, 47.3, 46.2, 29.1, 28.0, 27.6, 23.7, 23.5 ppm; ESI-HRMS: m/z [M + NH_4]$^+$ calcd. for $C_{47}H_{57}N_2O_8$: 777.4109; found 777.4116.

Benzylbenzyloxycarbonylaminopentyl 2,3,6-tri-*O*-benzyl-4-*O*-(2-naphthylmethyl)-α-D-glucopyranosyl-(1→4)-2,3,6-tri-*O*-benzyl-α-D-glucopyranosyl-(1→4)-2,3,6-tri-*O*-benzyl-α-D-glucopyranoside (45) A mixture of disaccharide donor **38** (80.0 mg, 67.0 μmol) and the acceptor **44** (40.7 mg, 53.6 μmol) was co-evaporated with toluene. Anhydrous DCM (1.4 mL) and anhydrous DMF (82.9 μL, 1.07 mmol) were added to the flask and the solution was stirred over fresh flame-dried MS 4 Å (120 mg, powdered) under nitrogen atmosphere for 20 minutes. The reaction flask was cooled to -78 °C and triflic acid (6.02 μL, 67.0 μmol) was added dropwise. After 45 minutes, the reaction solution was allowed to warm to 0 °C and stirred for 40 h more. Then, the reaction was quenched by adding NEt_3 (11.0 μL, 80.0 μmol), filtered through a bed of Celite and concentrated under reduced pressure. The product was purified by silica gel column chromatography (EtOAc/hexane = 1:12 – 1:4) and compound **45** (84.7 mg, 47.9 μmol, 89% yield) was obtained as a colorless viscous liquid. R_f = 0.3 (EtOAc/hexane = 1:3); ^{1}H NMR (400 MHz, $CDCl_3$): δ 7.82-7.80 (m, 1H), 7.73 (d, *J* = 8.4 Hz, 1H), 7.71-7.69 (m, 1H), 7.49-7.44 (m, 3H), 7.36-7.28 (m, 11H), 7.25-7.06 (m, 45H), 5.72 (d, *J* = 3.6 Hz, 1H), 5.62 (d, *J* = 3.5 Hz, 1H), 5.18 (d, *J* = 11.5 Hz, 2H), 5.05 (d, *J* = 11.5 Hz, 1H), 4.93 (t, *J* = 11.6 Hz, 2H), 4.84-4.65 (m, 6H, H-1), 4.58-4.39 (m, 13H), 4.23 (d, *J* = 12.2 Hz, 1H), 4.13-4.01 (m, 4H), 3.94-3.84 (m, 4H), 3.73-3.67 (m, 3H), 3.62-3.49 (m, 7H), 3.39-3.18 (m, 4H), 1.58-1.50 (m, 4H), 1.35-1.30 (m, 2H) ppm; ^{13}C NMR (100 MHz, $CDCl_3$): δ 139.1, 138.97, 138.90, 138.4, 138.29, 138.25, 138.1, 138.0, 137.7, 136.0, 133.3, 133.0, 128.68, 128.60, 128.5, 128.4, 128.39, 128.37, 128.35, 128.33, 128.31, 128.2, 128.06, 128.04, 127.99, 127.94, 127.8, 127.78, 127.75, 127.65, 127.62, 127.5, 127.4, 127.2, 127.1, 126.8, 126.68, 126.62, 126.18, 126.14, 125.9, 96.8, 96.5, 96.3, 82.2, 81.9, 81.8, 80.2, 79.6, 77.6, 77.4, 77.3, 77.1, 76.8, 75.5, 75.1, 74.3, 74.1, 73.5, 73.3, 73.1, 73.0, 72.3, 71.0, 70.8, 69.6, 68.9, 68.8, 68.1, 67.2, 29.8, 23.5 ppm; ESI-HRMS: m/z $[M + NH_4]^+$ calcd. for $C_{112}H_{121}N_2O_{18}$: 1782.8642; found 1782.8652.

Benzylbenzyloxycarbonylaminopentyl 2,3,6-tri-*O*-benzyl-α-D-glucopyranosyl-(1→4)-2,3,6-tri-*O*-benzyl-α-D-glucopyranosyl-(1→4)-2,3,6-tri-*O*-benzyl-α-D-glucopyranoside (46): To the solution of compound **45** (60.0 mg, 34 μmol) in DCM (2 mL) and aq. PBS buffer (pH = 7.4, 100 μL), DDQ (9.91 mg, 43.7 μmol) was added at 0 °C. The reaction was stirred at 0 °C for 3 h and was then quenched by adding aqu. satd. $NaHCO_3$ (2 mL). The phases were separated and the aqueous phase was extracted two more times with DCM. The combined organic phases were dried over Na_2SO_4, the solvent was removed *in vacuo,* and the product was purified by silica gel column chromatography (EtOAc/ hexane = 1:4 – 2:5). Compound **46** (40.0 mg, 72% yield) was obtained as a white foam. R_f = 0.4 (EtOAc/hexane = 3:7); ^{1}H NMR (400 MHz, $CDCl_3$): δ 7.37-7.27 (m, 23H), 7.25-7.17 (m, 26H), 7.14 (d, *J* = 7.6 Hz, 4H), 7.09 (d, *J* = 7.2 Hz, 2H), 5.71 (d, *J* = 3.5 Hz, 1H), 5.62 (d, *J* = 3.4 Hz, 1H), 5.19 (d, *J* = 12.3 Hz, 2H), 5.06 (d, *J* = 11.9 Hz, 1H), 4.97 (d, *J* = 11.6 Hz, 1H), 4.83 (d, *J* = 11.3 Hz, 2H), 4.75 (d, *J* = 11.8 Hz, 1H), 4.72 (d, *J* = 5.3 Hz, 1H), 4.67 (t, *J* = 12.0 Hz, 2H), 4.58-4.39 (m, 13H), 4.33 (d, *J* = 11.1 Hz, 1H), 4.10-4.02 (m, 4H), 3.91-3.85 (m, 3H), 3.76-3.51 (m, 10H), 3.46-3.41 (m, 2H), 3.30-3.21 (m, 3H), 1.66-1.49 (m, 4H), 1.36-1.30 (m, 2H) ppm; ^{13}C NMR (100 MHz, $CDCl_3$): δ 139.1, 138.8, 138.4, 138.2, 138.15, 138.13, 137.9, 137.7, 128.67, 128.61, 128.54, 128.52, 128.4, 128.38, 128.34, 128.32, 128.2, 128.0, 127.98, 127.92, 127.8, 127.78, 127.75, 127.68, 127.60, 127.49, 127.47, 127.3, 127.2, 127.1, 126.8, 126.6, 96.6, 96.3, 81.9, 81.8, 81.%, 80.2, 79.6, 79.2, 77.5, 77.4, 77.2, 76.8, 75.3, 74.3, 74.0, 73.6, 73.2, 73.1, 72.99, 72.93, 72.1, 71.6, 70.7, 70.4, 69.8, 69.6, 68.9, 68.8, 67.2, 50.6, 50.3, 47.2, 29.8, 29.2, 27.7, 23.5 ppm; ESI-HRMS: m/z $[M + NH_4]^+$ calcd. for $C_{101}H_{113}N_2O_{18}$: 1641.7983; found 1641.8007.

Benzylbenzyloxycarbonylaminopentyl 2,3,6-tri-*O*-benzyl-4-*O*-(2-naphthylmethyl)-α-D-glucopyranosyl-(1→4)-2,3,6-tri-*O*-benzyl-α-D-glucopyranosyl-(1→4)-2,3,6-tri-*O*-benzyl-α-D-glucopyranosyl-(1→4)-2,4,6-tri-*O*-benzyl-α-D-glucopyranosyl-(1→4)-2,3,6-tri-*O*-benzyl-α-D-glucopyranoside (47): A mixture of disaccharide donor **38** (12.0 mg, 10.1 μmol) and trisaccharide acceptor **46** (13.0 mg, 8.0 μmol) was co-evaporated with toluene. Anhydrous DCM (0.3 mL) and

anhydrous DMF (12.4 µL, 160 µmol) were added to the flask and the solution was stirred over fresh flame-dried MS 4 Å (25 mg, powdered) under nitrogen atmosphere for 20 minutes. The reaction flask was cooled to -78 °C and triflic acid (0.9 µL, 10.1 µmol) was added dropwise. After 45 minutes, the reaction solution was warmed up to 0 °C and stirred at this temperature for 48 h. The reaction was quenched by adding NEt_3 (2 µL), filtered through a bed of Celite and concentrated under reduced pressure. The product was purified by silica gel column chromatography (Toluene/EtOAc = 10/0 - 10:1) and compound **47** (16.0 mg, 76% yield) was obtained as a colorless viscous liquid. R_f = 0.63 (Toluene/EtOAc = 10/1); ^{1}H NMR (600 MHz, $CDCl_3$): δ 7.83-7.79 (m, 1 H), 7.76-7.67 (m, 2 H), 7.39-7.00 (m, 89 H), 5.70 (d, *J* = 3.6 Hz, 1 H), 5.65 (d, *J* = 4.2 Hz, 1 H), 5.62 (d, *J* = 3.3 Hz, 1 H), 5.59 (d, *J* = 3.2 Hz, 1 H), 5.17 (bd, *J* = 18.2 Hz, 2 H), 5.04 (d, *J* = 11.9 Hz, 1 H), 4.93 (d, *J* = 11.0 Hz, 1 H), 4.91 (d, *J* = 11.3 Hz, 1 H), 4.86-4.78 (m, 4 H), 4.77-4.64 (m, 6 H), 4.58-4.34 (m, 22 H), 4.21 (d, *J* = 12.8 Hz, 1 H), 4.11-3.82 (m, 12 H), 3.78-3.68 (m, 5 H), 3.64-3.57 (m, 3 H), 3.56-3.45 (m, 8 H), 3.39-3.17 (m, 5 H), 1.69-1.47 (m, 4 H), 1.39-1.29 (m, 2 H) ppm; ^{13}C NMR (150 MHz, $CDCl_3$): δ 139.1, 138.9, 138.4, 138.3, 138.2, 138.1, 138.0, 137.9, 137.8, 136.0, 133.3, 132.9, 128.5, 128.4, 128.3, 128.2, 128.1, 128.0, 127.9, 127.8, 127.7, 127.6, 127.5, 127.4, 127.3, 127.0, 126.9, 126.8, 126.6, 126.5, 126.0, 125.8, 96.9, 96.5, 96.3, 96.2, 96.1, 82.2, 81.8, 81.6, 60.2, 79.7, 79.6, 79.5, 79.4, 77.6, 75.4, 75.0, 74.1, 73.9, 73.8, 73.5, 73.3, 73.2, 73.0, 72.9, 72.8, 72.7, 72.5, 71.0, 70.9, 70.8, 69.6, 68.9, 68.8, 68.7, 68.2, 67.9, 67.2, 29.7, 29.1, 23.4 ppm; ESI-HRMS: m/z $[M+Na]^+$ calcd. for $C_{166}H_{173}NNaO_{28}$: 2652.2070; found 2652.2156.

Benzylbenzyloxycarbonylaminopentyl 2,3,6-tri-*O*-benzyl-4-*O*-(2-naphthylmethyl)-α-D-glucopyranosyl-(1→4)-2,3,6-tri-*O*-benzyl-α-D-glucopyranosyl-(1→4)-2,3,6-tri-*O*-benzyl-α-D-glucopyranosyl-(1→4)-2,3,6-tri-*O*-benzyl-α-D-glucopyranosyl-(1→4)-2,3,6-tri-*O*-benzyl-α-D-glucopyranosyl-(1→4)-2,3,6-tri-*O*-benzyl-α-D-glucopyranosyl-(1→4)-2,3,6-tri-*O*-benzyl-α-D-glucopyranoside (48): A mixture of tetrasaccharide donor **42** (25.1 mg, 12.0 µmol) and the trisaccharide acceptor **46** (16.5 mg, 10.2 µmol) was co-evaporated with toluene. Anhydrous DCM (1 mL) and anhydrous DMF (15.6 µL, 0.2 mmol) were added to the flask and the solution was stirred over fresh flame-dried MS 4 Å (50.0 mg, powdered) under Argon for 20 minutes. The reaction flask was cooled to -78 °C and triflic acid (1.1 µL, 12.0 µmol) was added. After 45 minutes, the reaction solution was stirred at 0 °C for 42 h. The reaction was quenched by adding NEt_3 (2.8 µL, 20.0 µmol), filtered through a bed of Celite and concentrated under reduced pressure. The product was purified by silica gel column chromatography (Toluene/EtOAc = 20:0 – 20:1) and compound **48** (20.0 mg, 56% yield) was obtained as a colorless oil. R_f = 0.7 (EtOAc/toluene = 1:10)**;** ^{1}H NMR (600 MHz, $CDCl_3$): δ 7.86-7.83 (m, 1 H), 7.76 (d, *J*=8.4 Hz, 1 H), 7.74-7.71 (m, 1 H), 7.54-7.47 (m, 4 H), 7.42-7.01 (m, 115 H), 5.74 (d, *J*=3.4 Hz, 1 H), 5.69 (d, *J*=3.3 Hz, 2 H), 5.68 (d, *J*=3.8 Hz, 1 H), 5.66 (d, *J*=3.4 Hz, 1 H), 5.64 (d, *J*=3.7 Hz, 1 H), 5.21 (d, *J*=19.2 Hz, 2 H), 5.08 (d, *J*=11.6 Hz, 1 H), 4.99-4.93 (m, 2 H), 4.91-4.82 (m, 6 H), 4.80-4.67 (m, 8 H), 4.61-4.36 (m, 30 H), 4.24 (d, *J*=12.5 Hz, 1 H), 4.15-4.00 (m, 12 H), 3.98 (dd, *J*=9.3, 7.9 Hz, 1 H), 3.95-3.86 (m, 7 H), 3.66 (bd, *J*=8.8 Hz, 1 H), 3.63 (dd, *J*=9.0, 3.5 Hz, 1 H), 3.60-3.47 (m, 12 H), 3.39 (d, *J*=9.6 Hz, 1 H), 3.34-3.20 (m, 2 H), 1.71-1.52 (m, 4 H), 1.40-1.33 (m, 2 H), ppm; ^{13}C NMR (150 MHz, $CDCl_3$): δ 139.1, 138.9, 138.8, 138.4, 138.3, 138.2, 138.1, 138.0, 137.9, 137.8, 136.0, 133.2, 132.9, 128.5, 128.4, 28.3, 128.2, 128.1, 128.0, 127.9, 127.8, 127.7, 127.6, 127.5, 127.4, 127.3, 127.0, 126.8, 126.6, 126.5, 126.0, 125.8, 96.9, 96.5, 96.3 (2 C), 96.2, 96.1, 96.0, 82.1, 81.8, 81.7, 81.6, 81.5, 80.2, 79.7, 79.6, 79.5, 79.4, 77.6, 75.4, 75.0, 74.1, 73.9, 73.8, 73.7, 73.5, 73.3, 73.2, 73.0, 72.9, 72.8, 72.7, 72.5, 72.3, 71.0, 70.8, 70.7, 69.6, 68.9, 68.8, 68.6, 68.2, 68.0, 67.2, 50.6, 50.3, 47.2, 29.3, 29.1, 23.4 ppm; ESI-HRMS: m/z $[M+H+NH_4]^{2+}$ calcd. for $C_{221}H_{238}N_2O_{38}$: 1764.8405; found 1764.8418.

5-Aminopentyl α-D-glucopyranosyl-(1→4)-α-D-glucopyranosyl-(1→4)-α-D-glucopyranoside (49): The trisaccharide **45** (20.0 mg, 11.30 µmol) was dissolved in ^{t}BuOH (1.2 mL) followed by the addition of water (0.6 mL) and AcOH (0.15 mL). Pd-C (10 w %; 39 mg) was added and the reaction was stirred at 23 °C under H_2 atmosphere for 4 days. The reaction solution was filtered through Celite and washed with MeOH and H_2O. The filtrate was concentrated *in vacuo* and the crude product was purified by HPLC using a ZIC-HILIC column (MeCN/ 20 mM aq. NH_4COO = 90/10 → 30/70) to obtain the fully deprotected trisaccharide **49** (3.6 mg, 54% yield) as colorless oil. ^{1}H NMR (600 MHz, D_2O): δ 5.39 (d, *J* = 4.0 Hz, 2H, H-1^{B-C}), 4.90 (d, *J* = 3.5 Hz, 1H, H-1^{A}), 3.97-3.91 (m, 2 H, H-3^{A}/H-3^{B}), 3.87-3.71 (m, H-5^{B-C}/H-6a+b^{A-C}/OCH_aCH$_2$), 3.71-56 (H-5^{A}/H-3^{C}/H-4^{A-B}/H-2^{A-C}), 3.53 (dt, *J* = 10.0, 6.2 Hz, OCH$_b$CH$_2$), 3.41 (t, *J* = 9.6 Hz, 1 H, H-4^{C}), 3.00 (t, *J* = 7.6 Hz, 2H, CH_2NH_2), 1.72-1.62 (m, 4H, CH_2 Linker), 1.52-1.40 (m, 2H, CH_2 Linker) ppm; ^{13}C NMR (150 MHz, D_2O): δ 100.5, 100.2 (2 C, C-1^{B}/C-1^{C}), 98.6 (C-1^{A}), 77.8, 77.5 (C-3^{A-B}), 74.3, 74.1 (C-3^{A-B}), 73.6, 73.5 (C-5^{A}/C-3^{C}), 72.4, 72.2, 71.9, 71.8 (C-5^{B}/C-2^{A-C}), 71.0 (C-5^{C}), 70.0 (C-4^{C}), 68.6 (OCH_2CH$_2$), 61.3, 61.2 (3 C, C-6), 40.1 (CH_2NH_2), 28.8, 27.2, 23.1 (CH_2 Linker) ppm; ESI-HRMS: m/z [M+H]$^+$ calcd. for $C_{23}H_{44}NO_{16}$: 590.2654; found 590.2666.

5-Aminopentyl α-D-glucopyranosyl-(1→4)-α-D-glucopyranosyl-(1→4)-α-D-glucopyranosyl-(1→4)- α-D-glucopyranosyl-(1→4)-α-D-glucopyranoside (50): The pentasaccharide **47** (20.0 mg, 7.60 µmol) was dissolved in ^{t}BuOH (1.2 mL) followed by the addition of water (0.6 mL) and AcOH (0.1 mL). Pd-C (10 w%; 20 mg) was added and the reaction was stirred at 23 °C under H_2 atmosphere for 5 days. The reaction solution was filtered through Celite, washed with MeOH and H_2O. The filtrate was concentrated *in vacuo* and the crude product was purified by HPLC using a ZIC-HILIC column (MeCN/ 20 mM aq. NH_4COO = 90/10 → 30/70) to obtain the fully deprotected pentasaccharide **50** (1.6 mg, 23% yield) as colorless oil. ^{1}H NMR (600 MHz, D_2O): δ 5.41-5.39 (m, 4 H, H-1^{B-E}), 4.89 (d, *J* = 3.5 Hz, 1H, H-1^{A}), 3.97-3.90 (m, 4 H, H-3^{A-D}), 3.87-3.55 (m, 21 H, H-5^{A-E}/H-6^{A-E}/OCH_aCH$_2$/H-3^{E}/H-4^{A-D}/H-2^{A-E}), 3.53 (dt, *J* = 10.0, 6.2 Hz, OCH$_b$CH$_2$), 3.41 (t, *J* = 9.6 Hz, 1 H, H-4^{E}), 3.00 (t, *J* = 7.6 Hz, 2H, CH_2NH_2), 1.72-1.62 (m, 4H, CH_2 Linker), 1.52-1.40 (m, 2H, CH_2 Linker) ppm; ^{13}C NMR (150 MHz, D_2O): δ 100.5-100.1 (4 C, C-1^{B-E}), 98.6 (C-1^{A}), 77.6-77.5 (4 C, C-3^{A-D}), 74.3-74.0 (4 C, C-3^{A-D}), 73.6, 73.4 (C-5^{A}/C-3^{E}), 72.4, 72.2, 71.9, 71.8 (7 C, C-5^{B-D}/C-2^{A-E}), 71.0 (C-5^{E}), 70.0 (C-4^{E}), 68.6 (OCH_2CH$_2$), 61.3-61.2 (5 C, C-6), 40.1 (CH_2NH_2), 28.8, 27.2, 23.1 (CH_2 Linker) ppm; ESI-HRMS: m/z [M+H]$^+$ calcd. for $C_{35}H_{63}NO_{26}$: 914.3711; found 914.3731.

5-Aminopentyl α-D-glucopyranosyl-(1→4)-α-D-glucopyranosyl-(1→4)- α-D-glucopyranosyl-(1→4)-α-D-glucopyranosyl-(1→4)-α-D-glucopyranosyl-(1→4)- α-D-glucopyranosyl-(1→4)-α-D-glucopyranoside (51): The heptasaccharide **48** (12.0 mg, 3.4 µmol) was dissolved in ^{t}BuOH (1.2 mL) followed by the addition of water (0.6 mL) and AcOH (0.1 mL). Pd-C (10 w%; 12 mg) was added and the reaction was stirred at 23 °C under H_2 atmosphere for 5 days. The reaction solution was filtered through Celite, washed with MeOH and H_2O. The filtrate was concentrated *in vacuo* and the crude product was purified by HPLC using a ZIC-HILIC column (MeCN/ 20 mM aq. NH_4COO = 90/10 → 30/70) to obtain the fully deprotected pentasaccharide **51** (1.6 mg, 38% yield) as colorless oil. ^{1}H NMR (600 MHz, D_2O): δ 5.41-5.37 (m, 6 H, H-1^{B-G}), 4.90 (d, *J* = 3.5 Hz, 1H, H-1^{A}), 3.97-3.90 (m, 6 H, H-3^{A-F}), 3.87-3.55 (m, 29 H, H-5^{A-G}/H-6^{A-G}/OCH_aCH$_2$/H-3^{G}/H-4^{A-F}/H-2^{A-G}), 3.53 (dt, *J* = 10.0, 6.2 Hz, OCH$_b$CH$_2$), 3.41 (t, *J* = 9.6 Hz, 1 H, H-4^{G}), 3.00 (t, *J* = 7.6 Hz, 2H, CH_2NH_2), 1.72-1.62 (m, 4H, CH_2 Linker), 1.52-1.40 (m, 2H, CH_2 Linker) ppm; ^{13}C NMR (150 MHz, D_2O): δ 100.5-100.1 (6 C, C-1^{B-G}), 98.6 (C-1^{A}), 77.6-77.5 (4 C, C-3^{A-F}), 74.3-74.0 (6 C, C-3^{A-F}), 73.6, 73.4 (C-5^{A}/C-3^{G}), 72.4-71.7 (12 C, C-5^{B-F}/C-2^{A-G}), 71.0 (C-5^{G}), 70.0 (C-4^{G}), 68.6 (OCH_2CH$_2$), 61.3-61.2 (5 C, C-6), 40.1 (CH_2NH_2), 28.8, 27.2, 23.1 (CH_2 Linker) ppm; ESI-HRMS: m/z [M+H]$^+$ calcd. for $C_{47}H_{84}NO_{36}$: 1238.4768; found 1238.4800.

1.2 Analytical data of Chitin series

^{1}H and ^{13}C NMR of glycosyl phosphate **2**

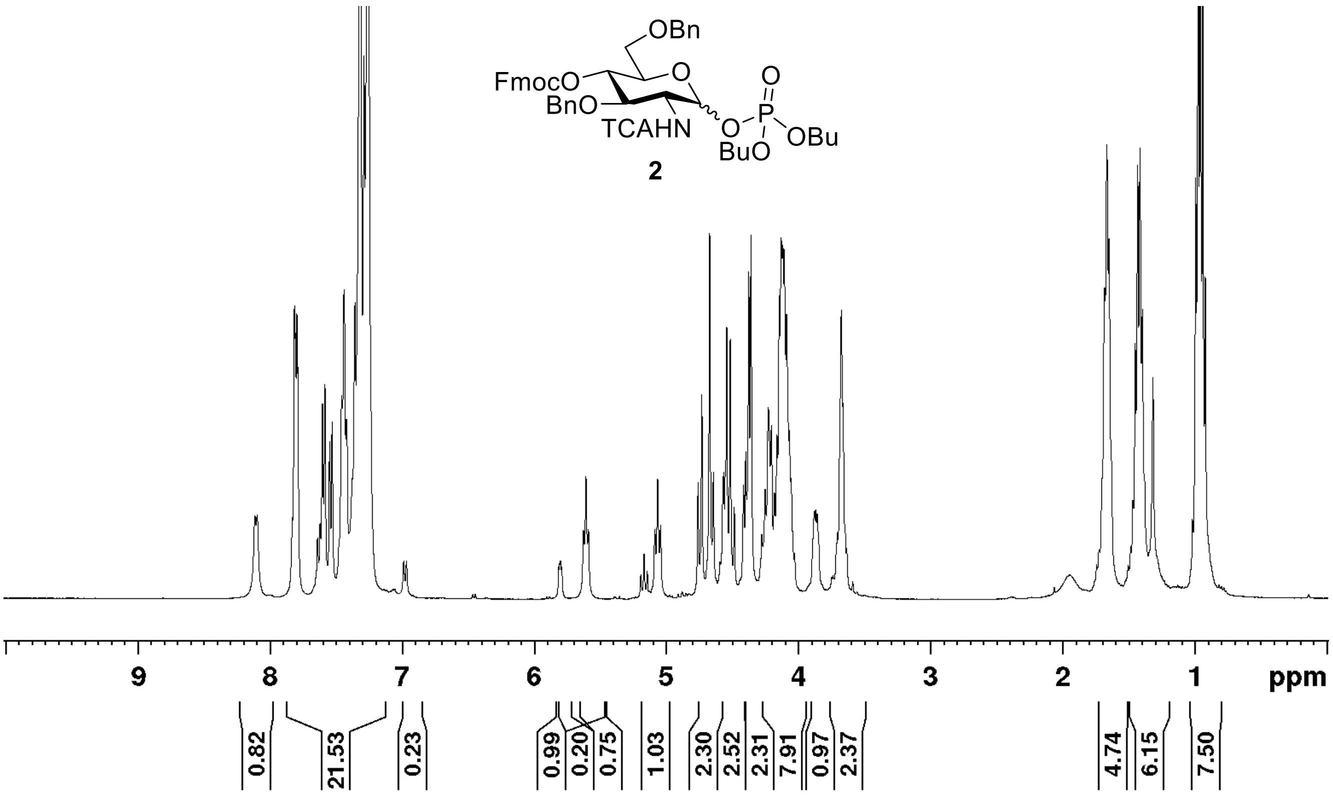

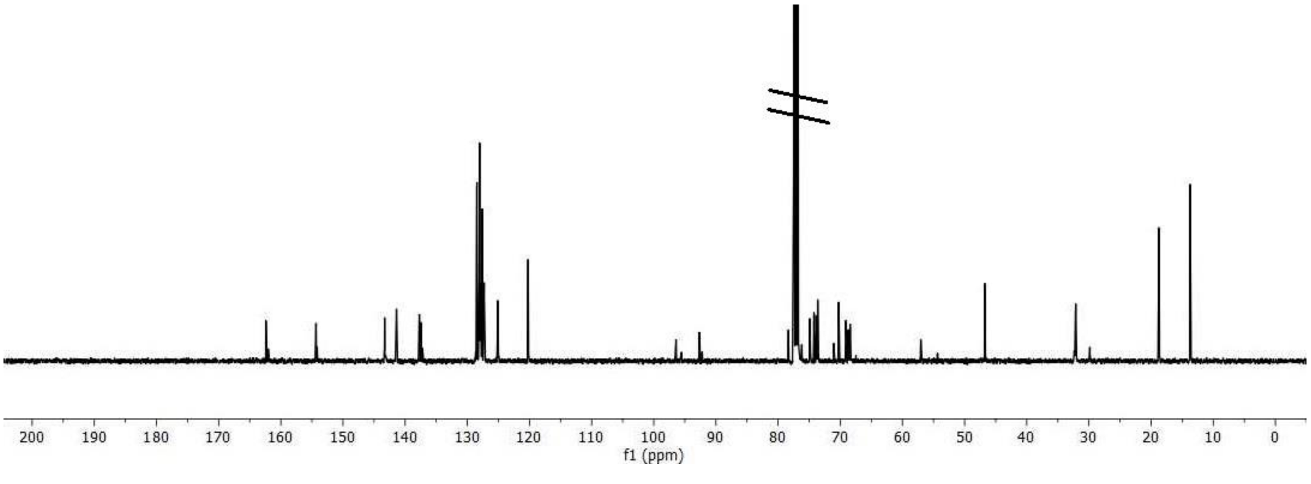

HPLC (diol column, Hex/EtOAc = 80/20 → 0/100 of crude trisaccharide **3**

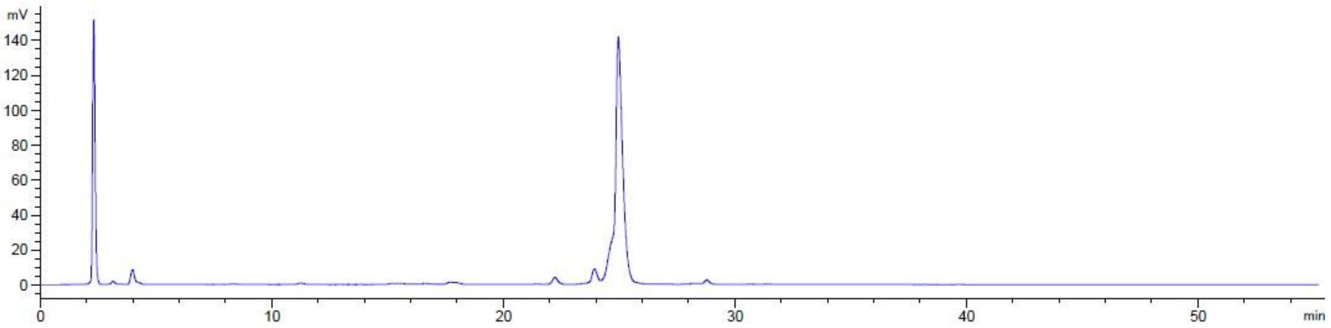

^{1}H and ^{13}C-NMR of purified trisaccharide **3**

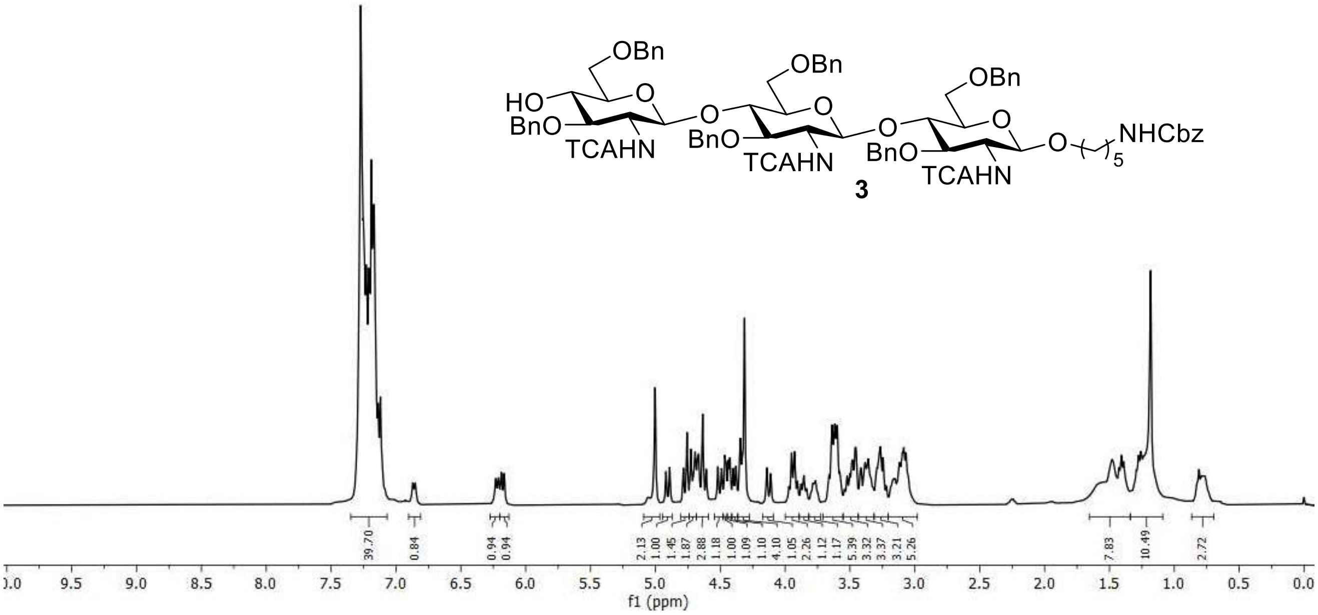

3

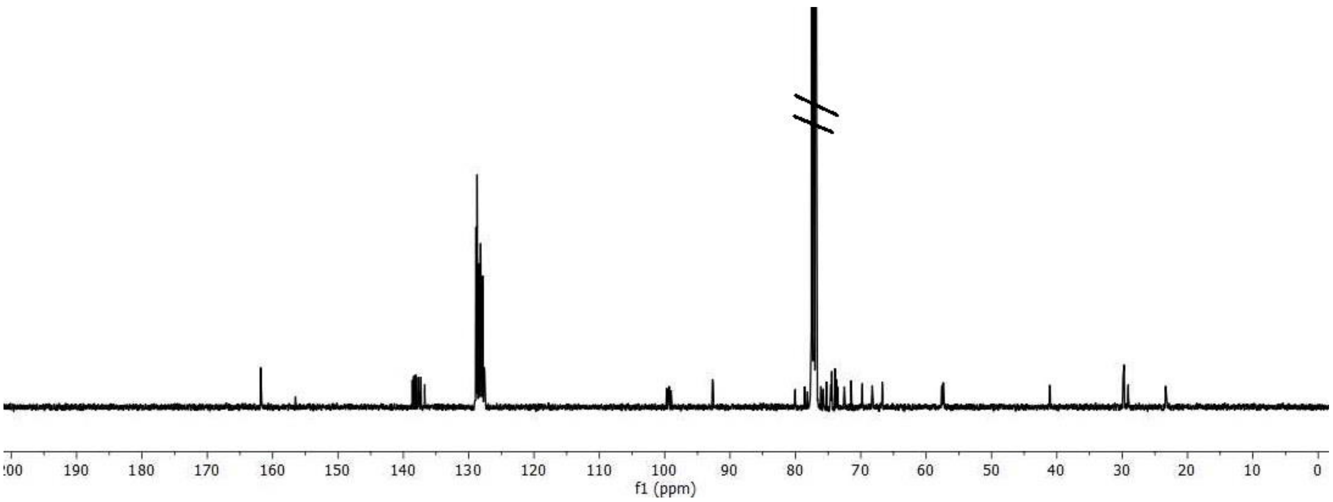

HPLC (diol column, Hex/EtOAc = 80/20 → 0/100) of crude pentasaccharide **4**

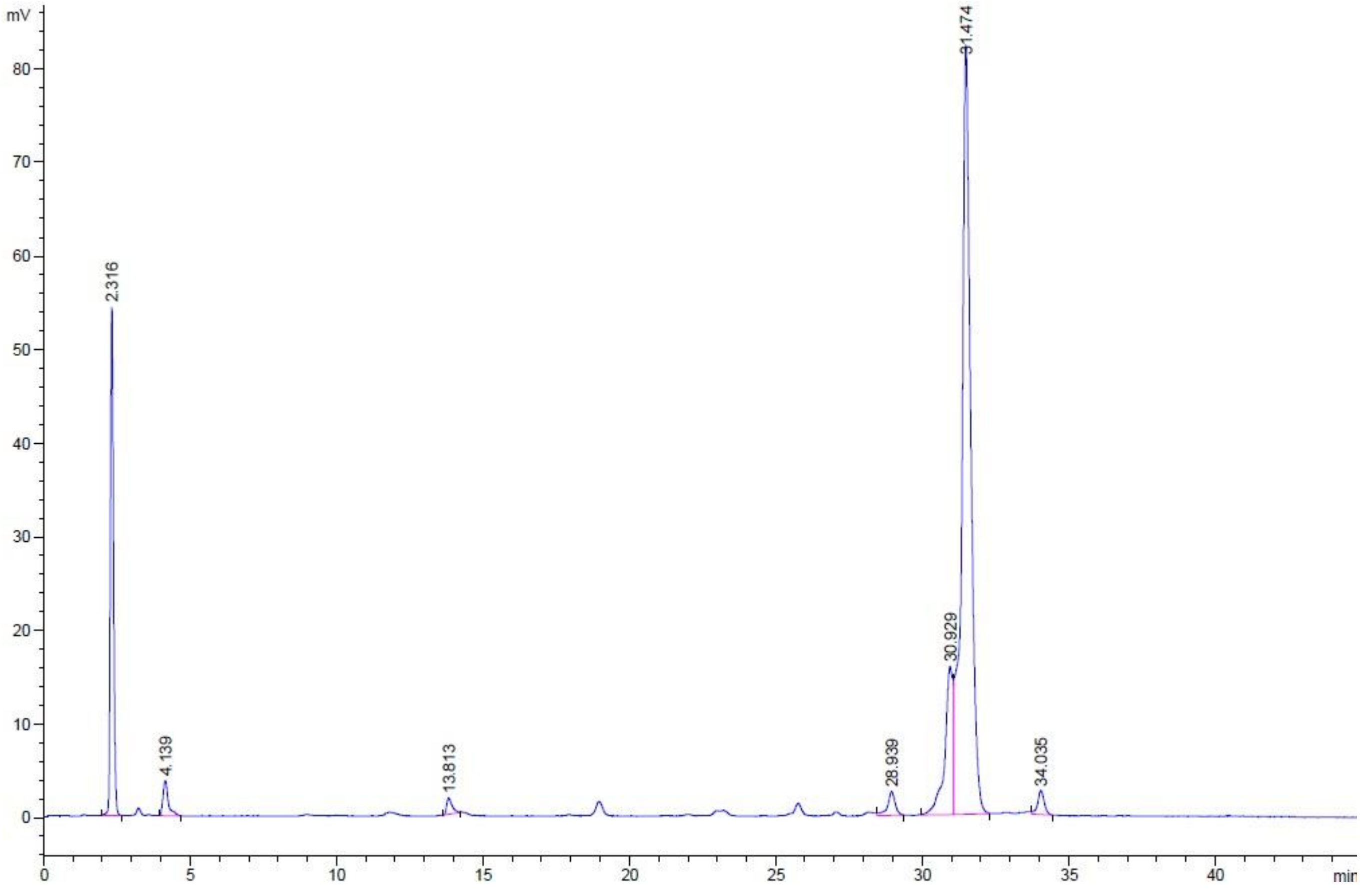

^{1}H, ^{13}C and NMR of purified pentasaccharide **4**

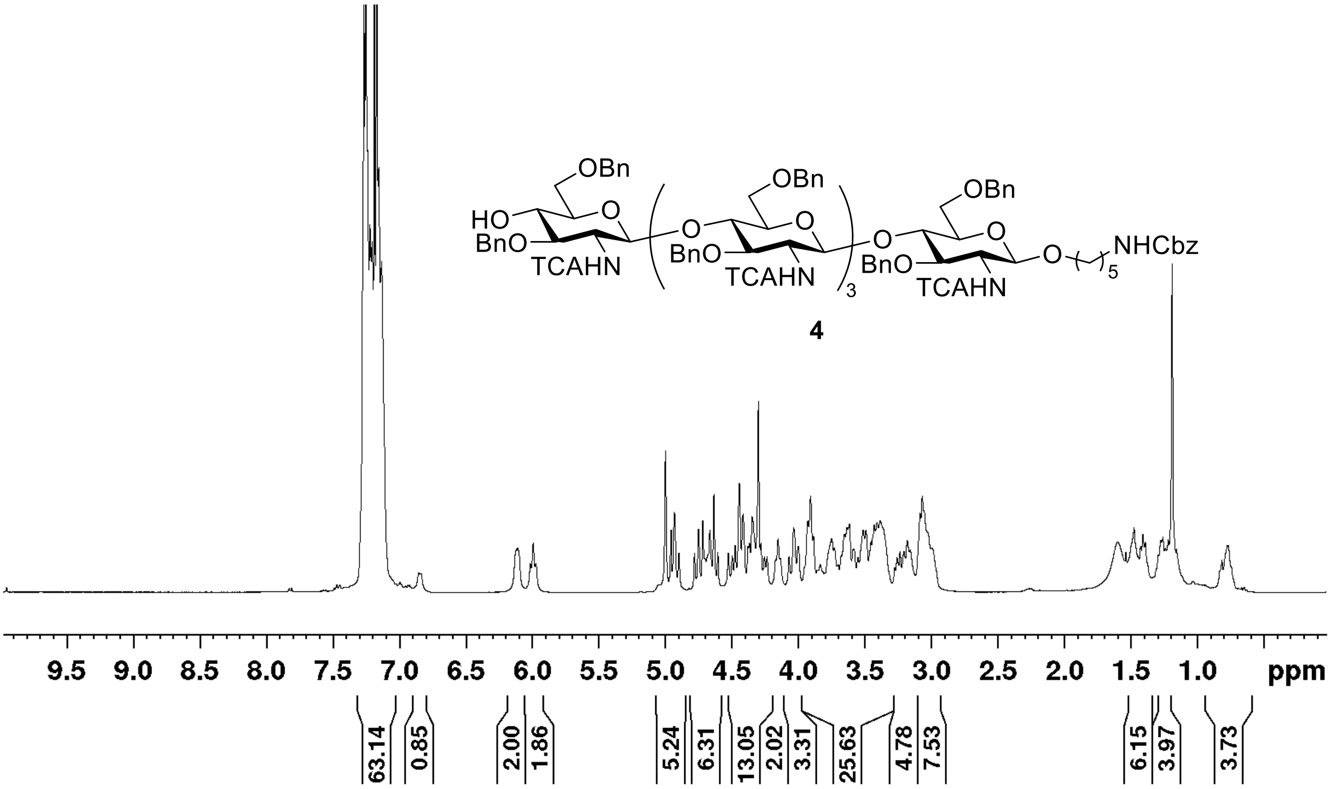

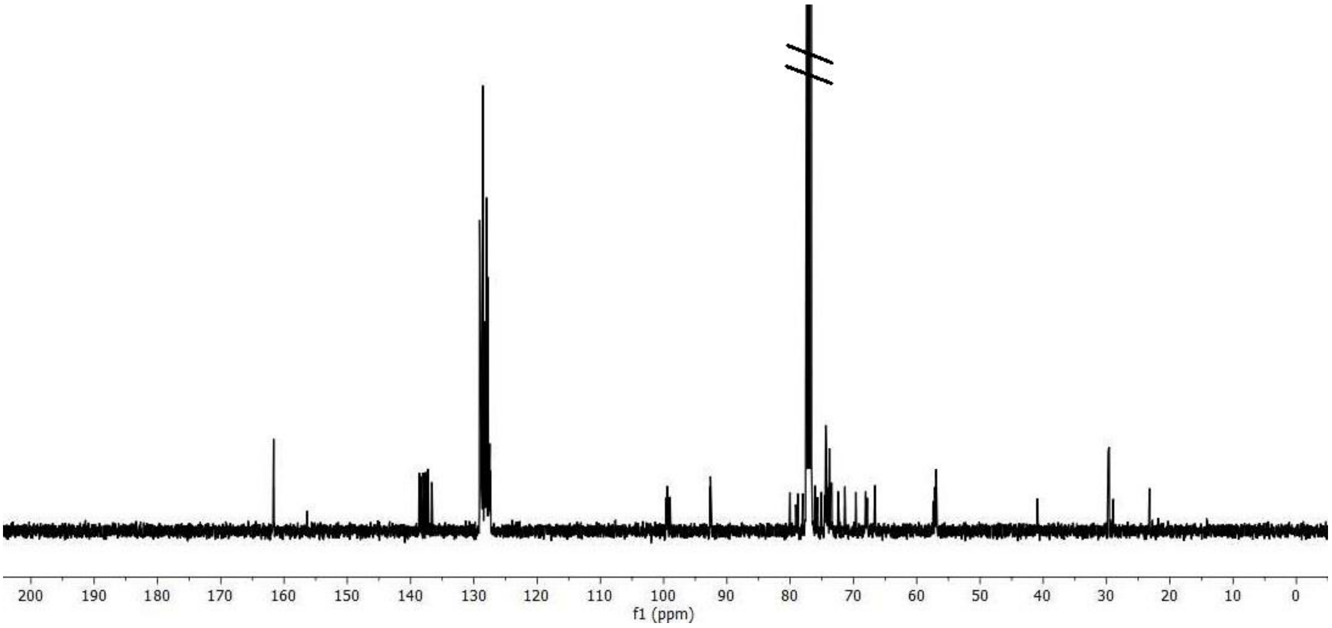

HPLC (diol column, Hex/EtOAc = 80/20 → 0/100) of crude heptasaccharide **5**

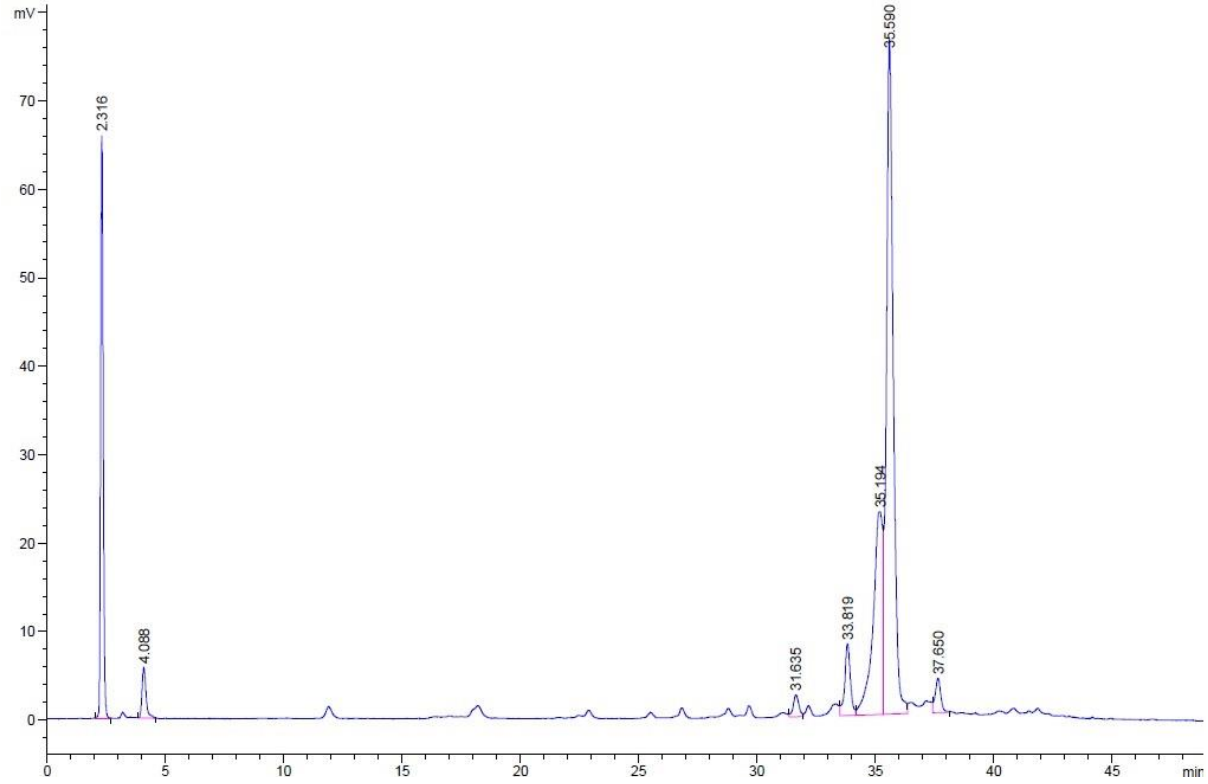

^{1}H and ^{13}C-NMR of purified heptasaccharide **5**

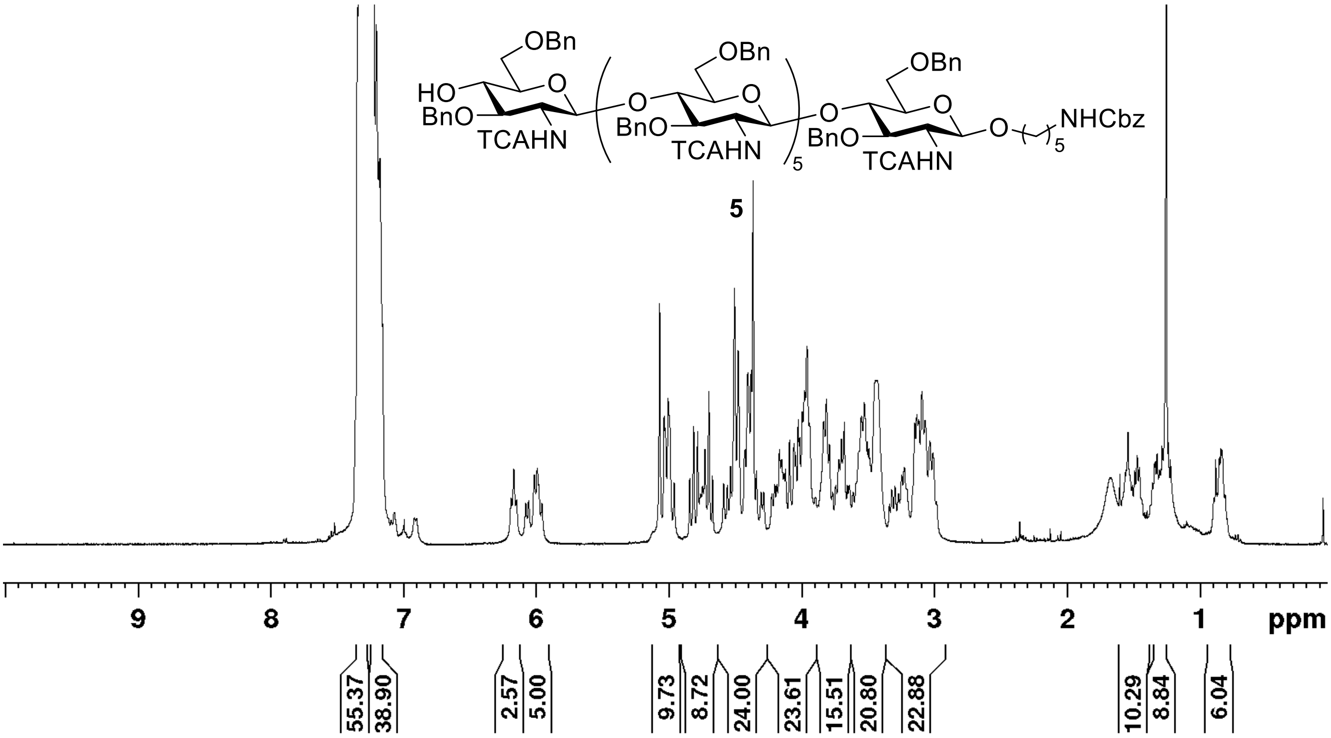

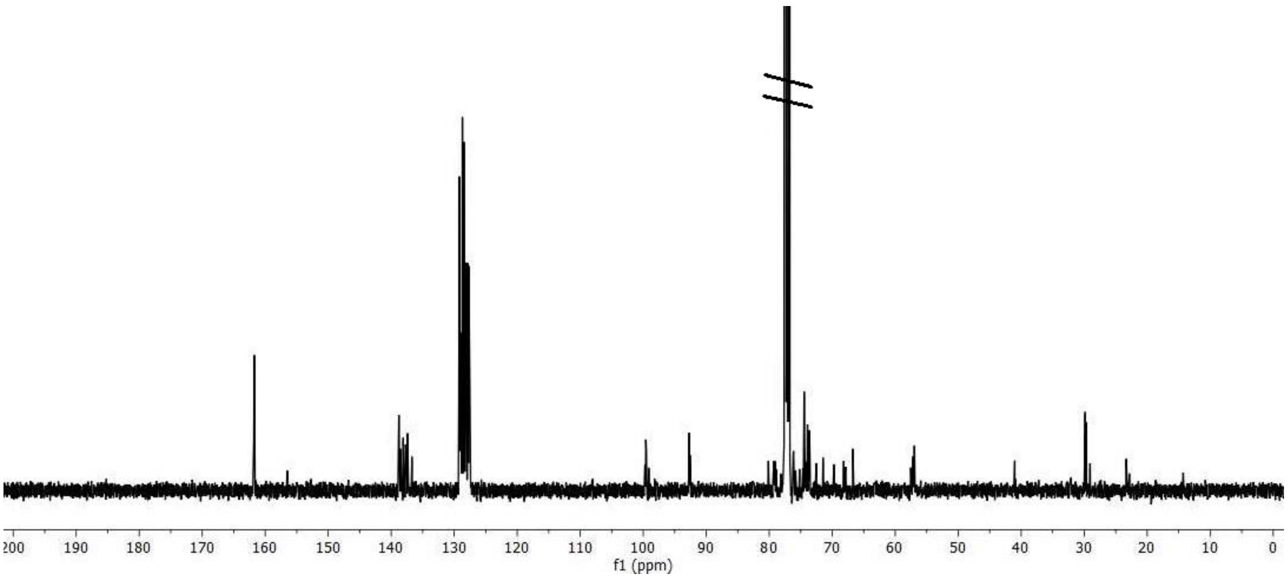

^{1}H, ^{13}C and HSQC-NMR of trisaccharide **6**

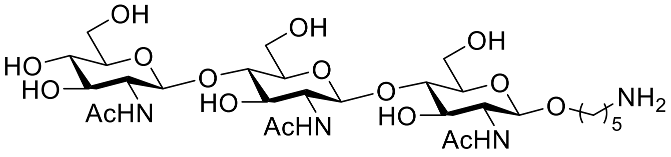

6

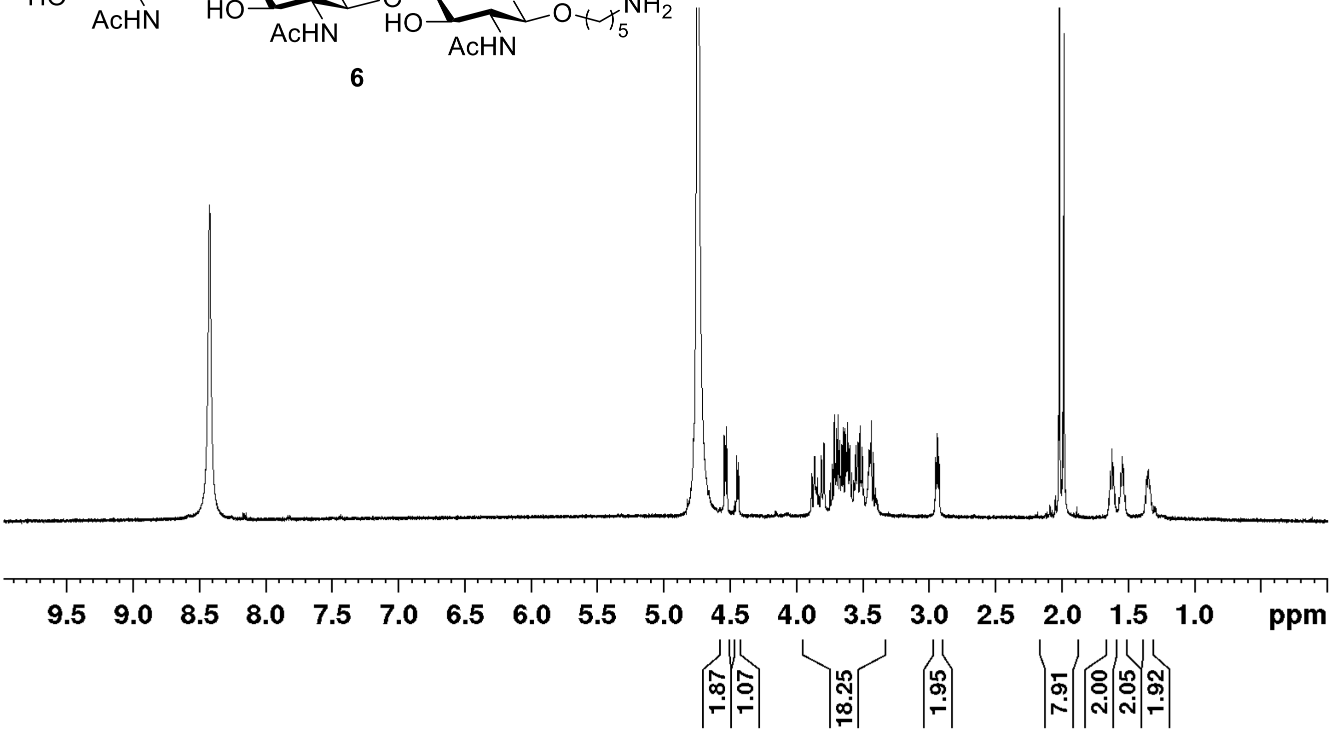

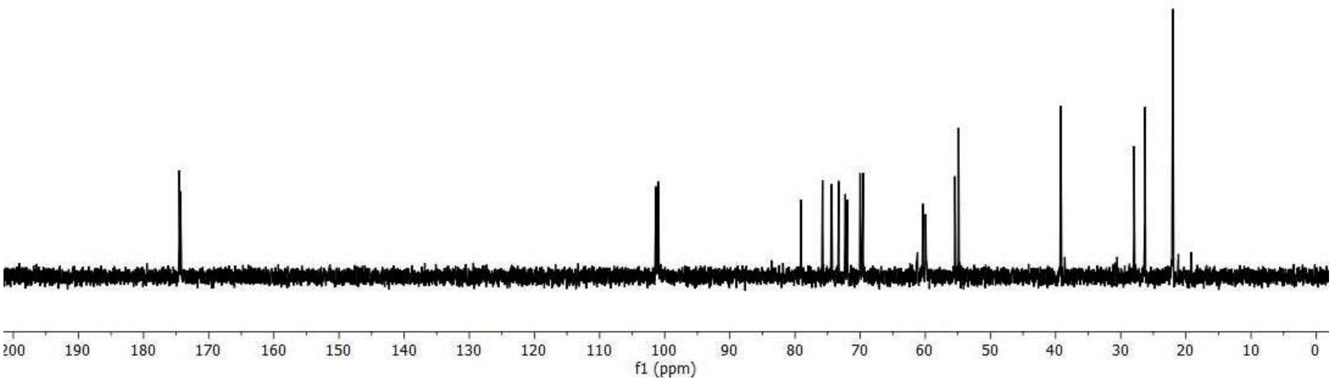
200 190 180 170 160 150 140 130 120 110 100 90 80 70 60 50 40 30 20 10 0
f1 (ppm)

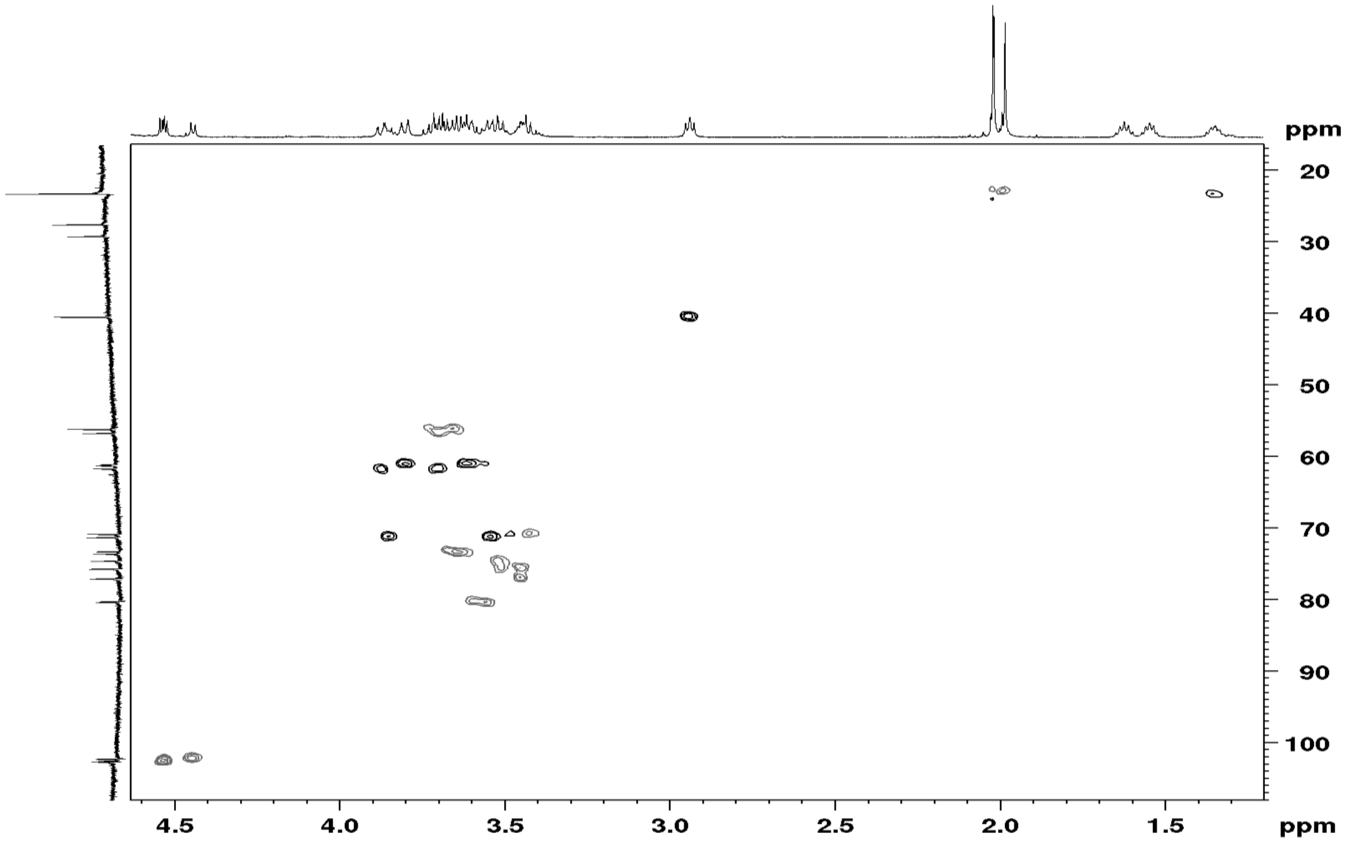
ppm
20 30 40 50 60 70 80 90 100
4.5 4.0 3.5 3.0 2.5 2.0 1.5
ppm

HPLC (synergy column; H_2O (0.1% FA)/CH_3CN = 100/0 → 0/100 (in next 10 mins)) of purified trisaccharide **6**

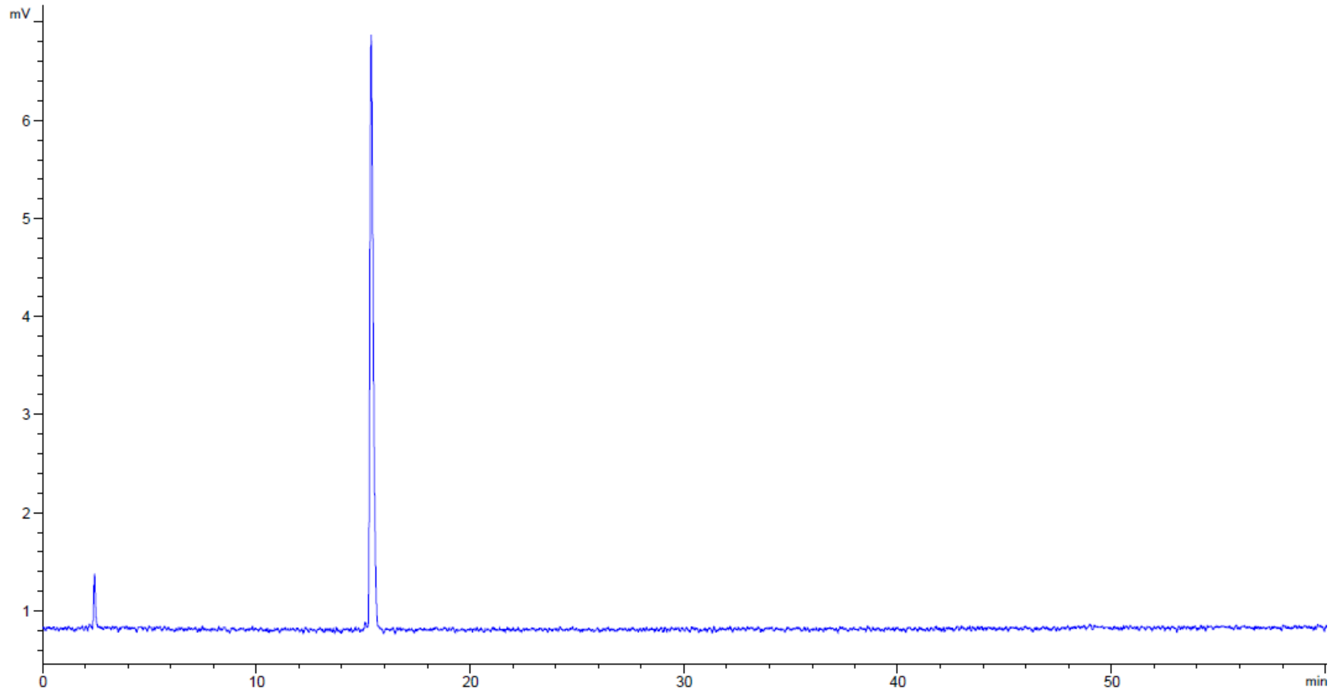

^{1}H, ^{13}C and HSQC-NMR of pentasaccharide **7**

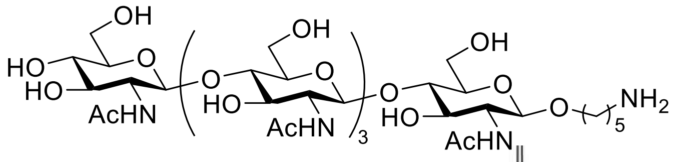

7

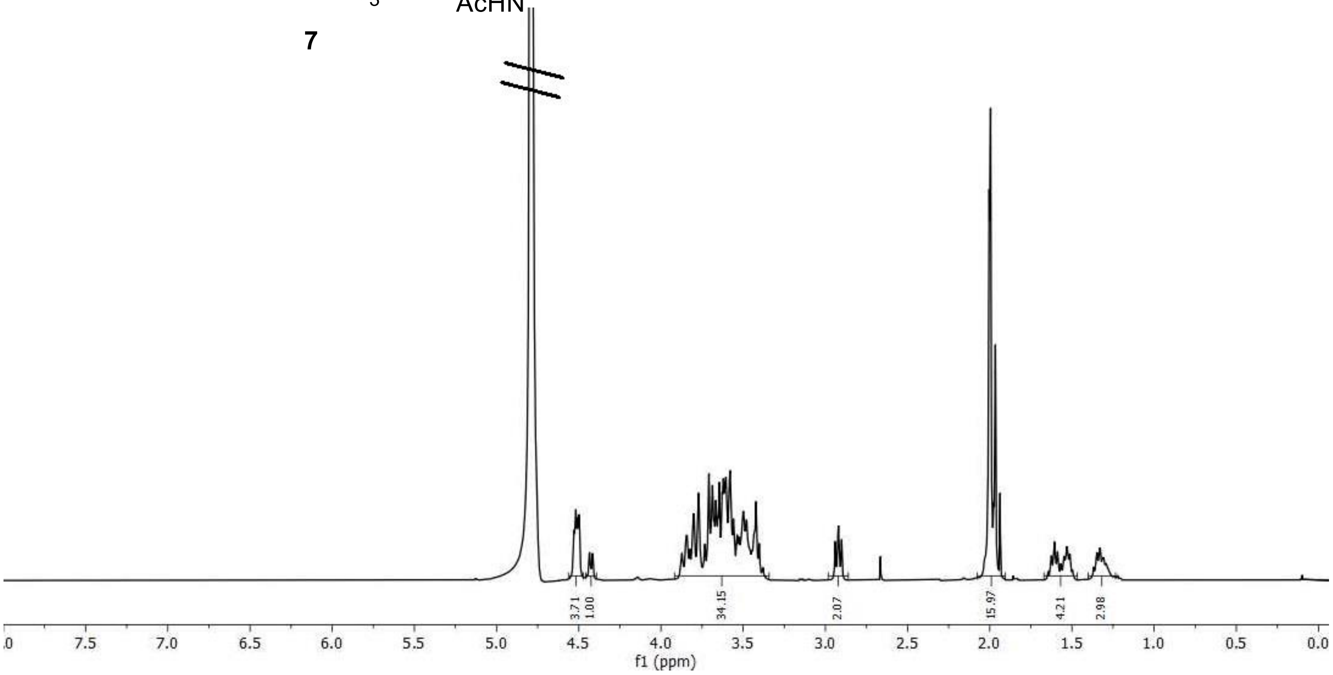

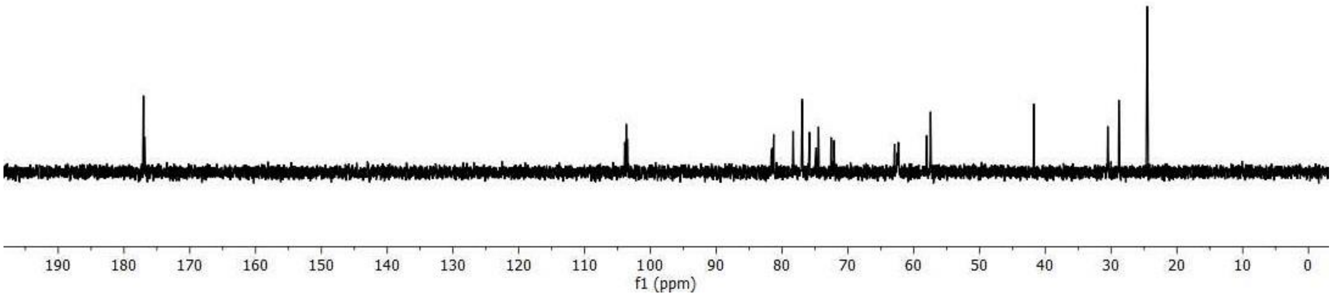

190 180 170 160 150 140 130 120 110 100 90 80 70 60 50 40 30 20 10 0
f1 (ppm)

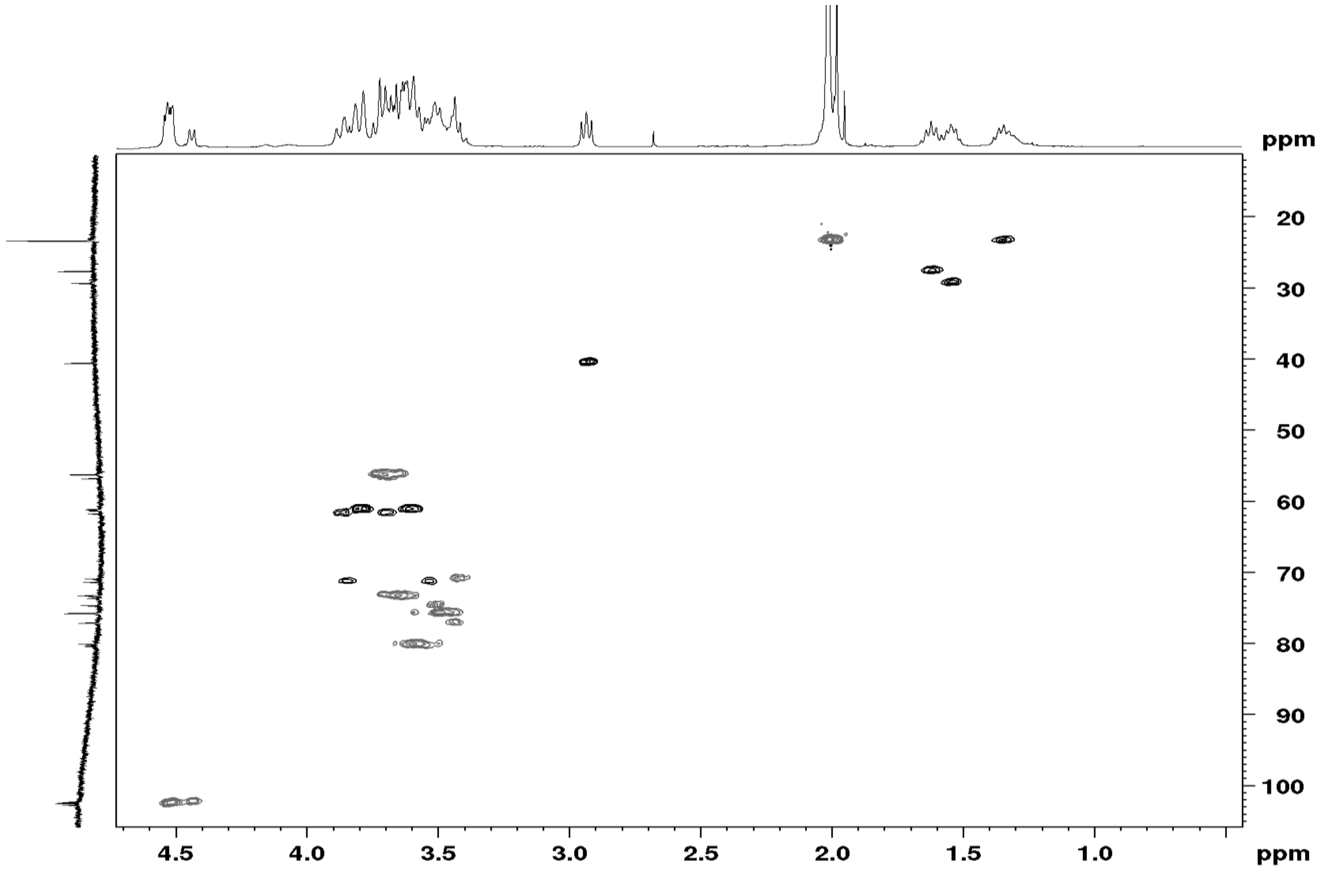

ppm
20
30
40
50
60
70
80
90
100
4.5 4.0 3.5 3.0 2.5 2.0 1.5 1.0
ppm

HPLC (synergy column; H_2O (0.1% FA)/CH_3CN = 100/0 → 0/100) of purified pentasaccharide **7**

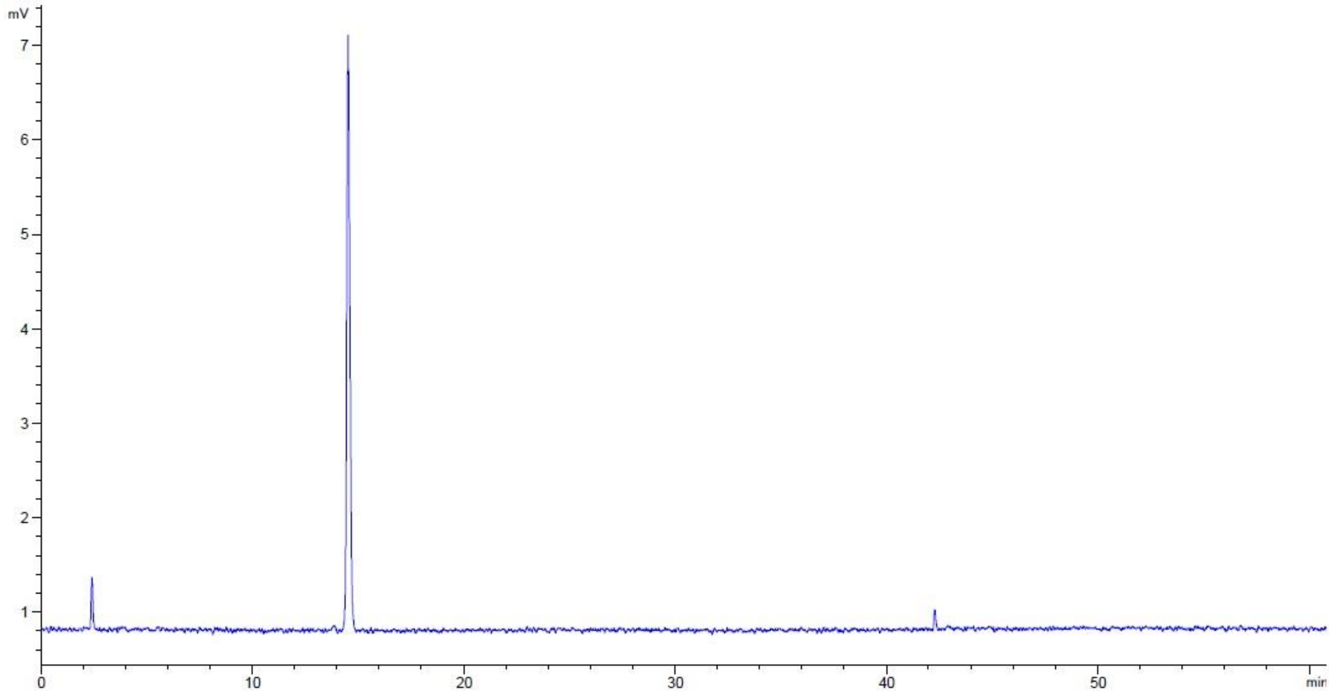

^{1}H, ^{13}C and HSQC-NMR of heptasaccharide **8**

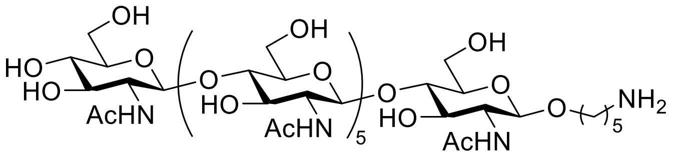

8

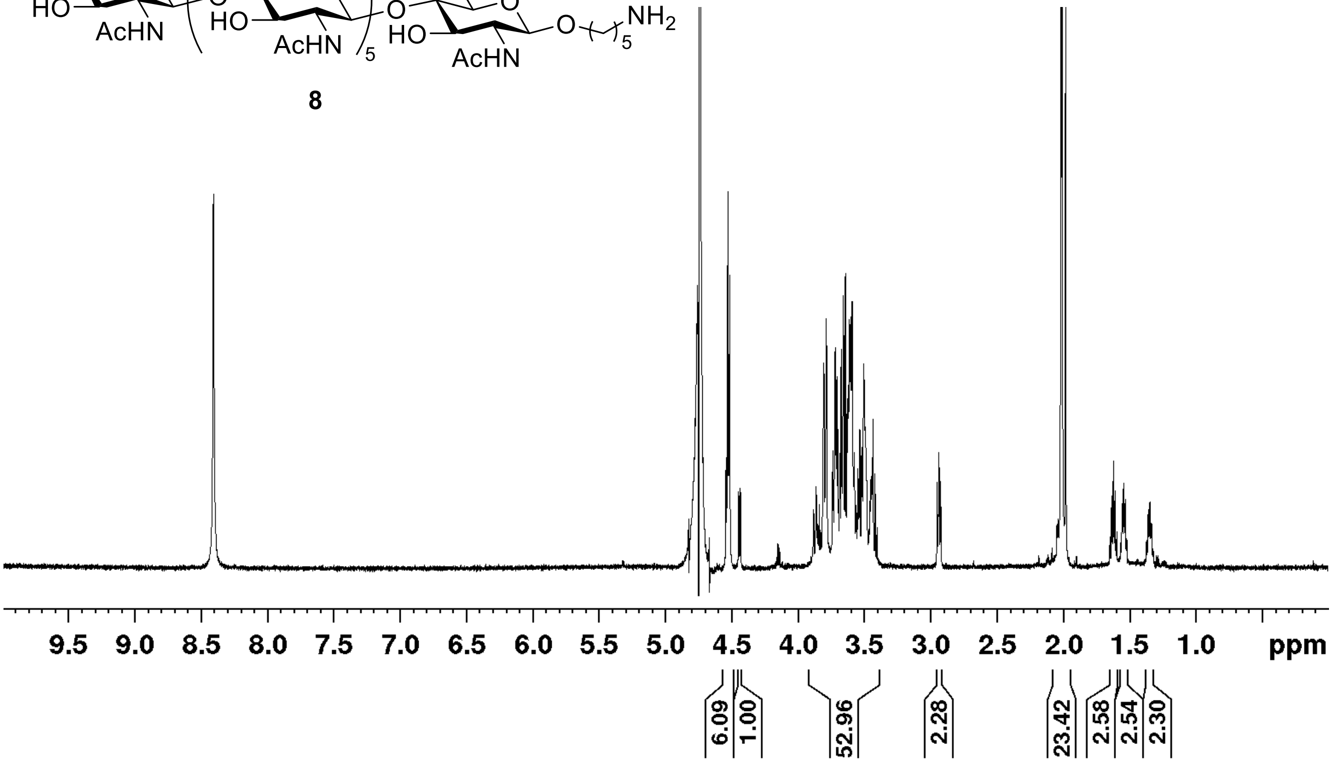

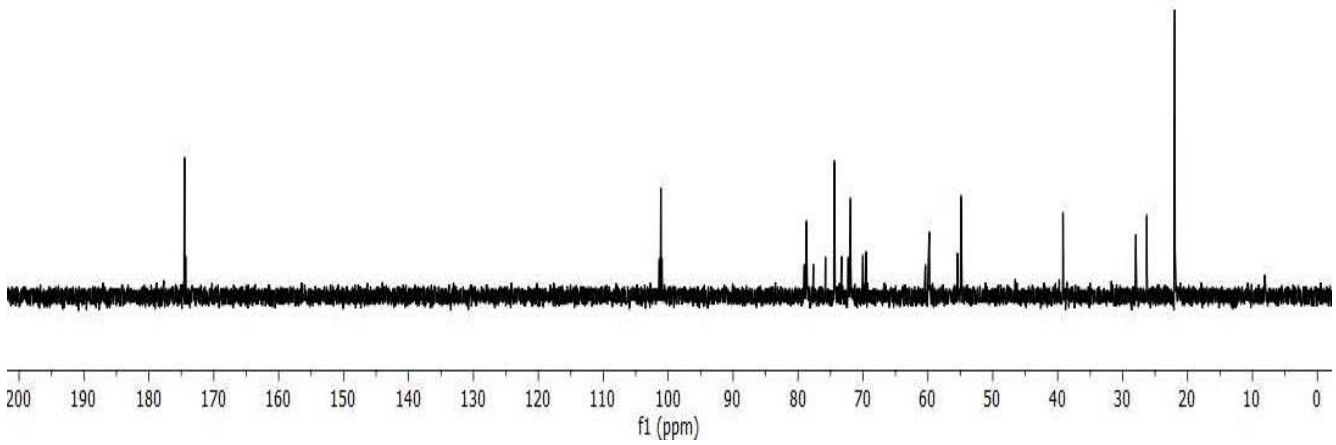
200
190
180
170
160
150
140
130
120
110
100
90
80
70
60
50
40
30
20
10
0
f1 (ppm)

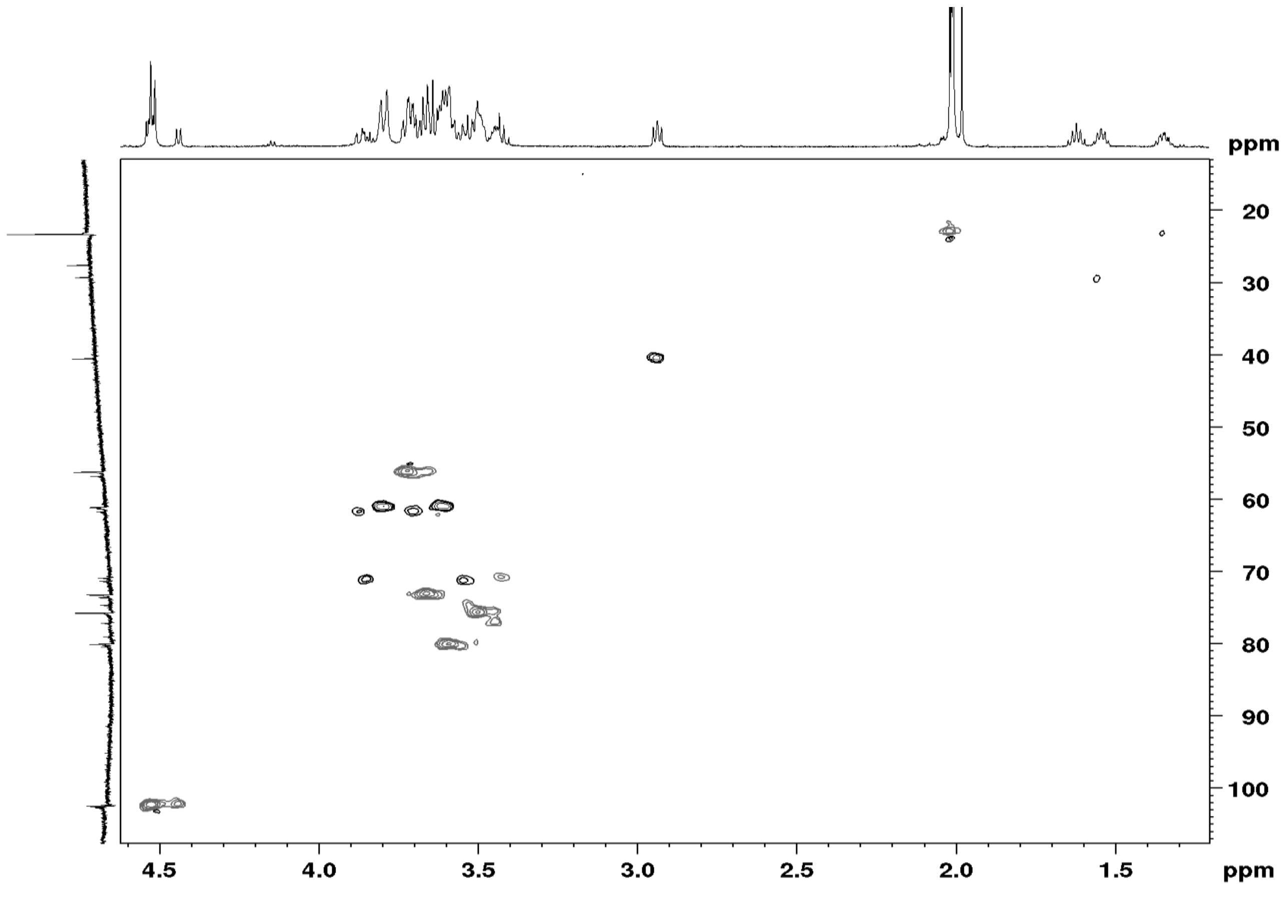
ppm
20
30
40
50
60
70
80
90
100
4.5
4.0
3.5
3.0
2.5
2.0
1.5
ppm

HPLC (synergy column; H_2O (0.1% FA)/CH_3CN = 100/0 → 0/100) of purified heptasaccharide **8**

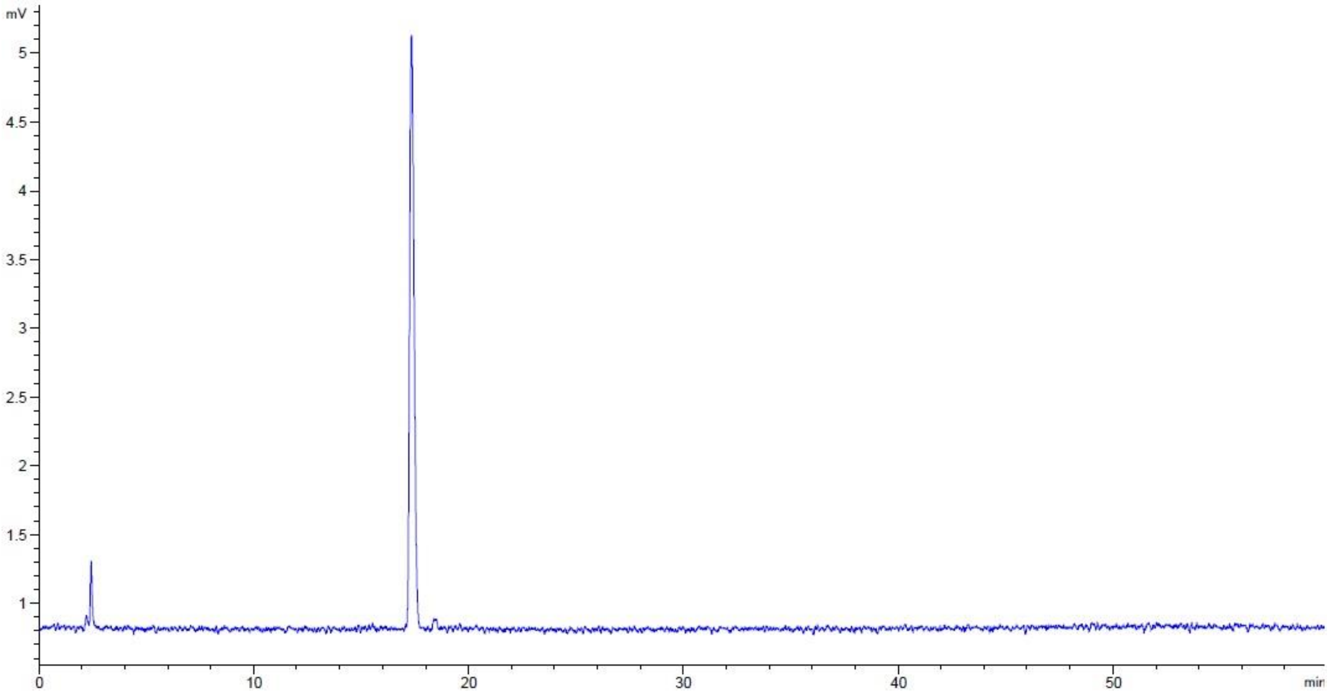

1.3 Analytical data of β-[1→6]-series

HPLC (diol column, Hex/EtOAc = 80/20 → 0/100) of crude trisaccharide **10**

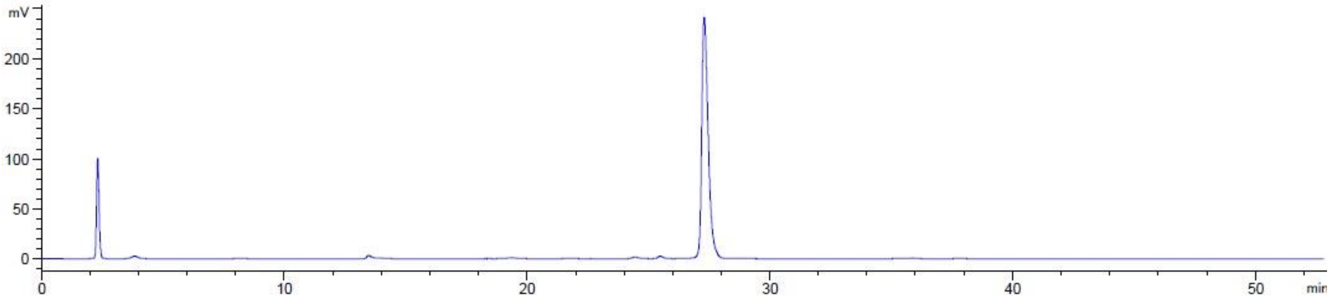

^{1}H and ^{13}C NMR spectra of purified trisaccharide **10**

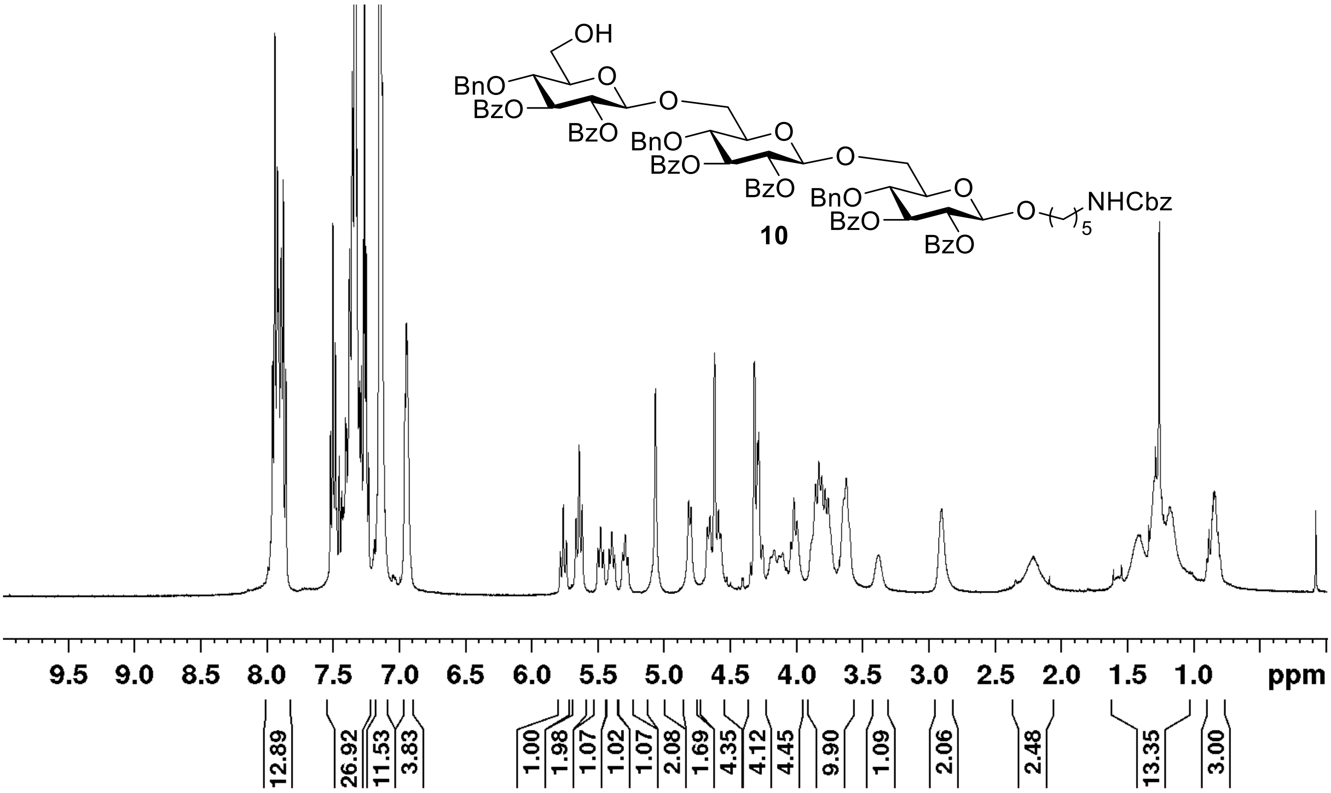

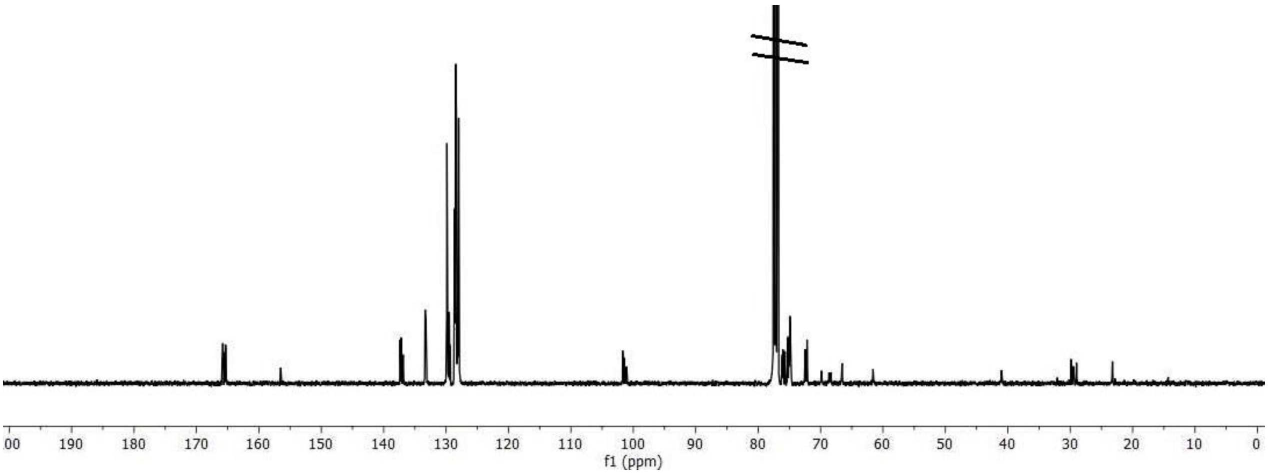

HPLC (diol column, Hex/EtOAc = 80/20 → 0/100) of crude pentasaccharide **11**

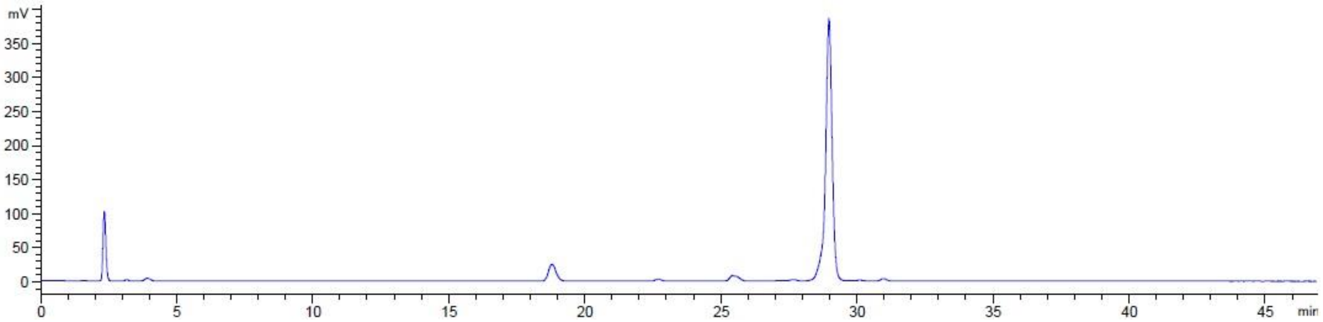

^{1}H and ^{13}C NMR spectra of purified pentasaccharide **11**

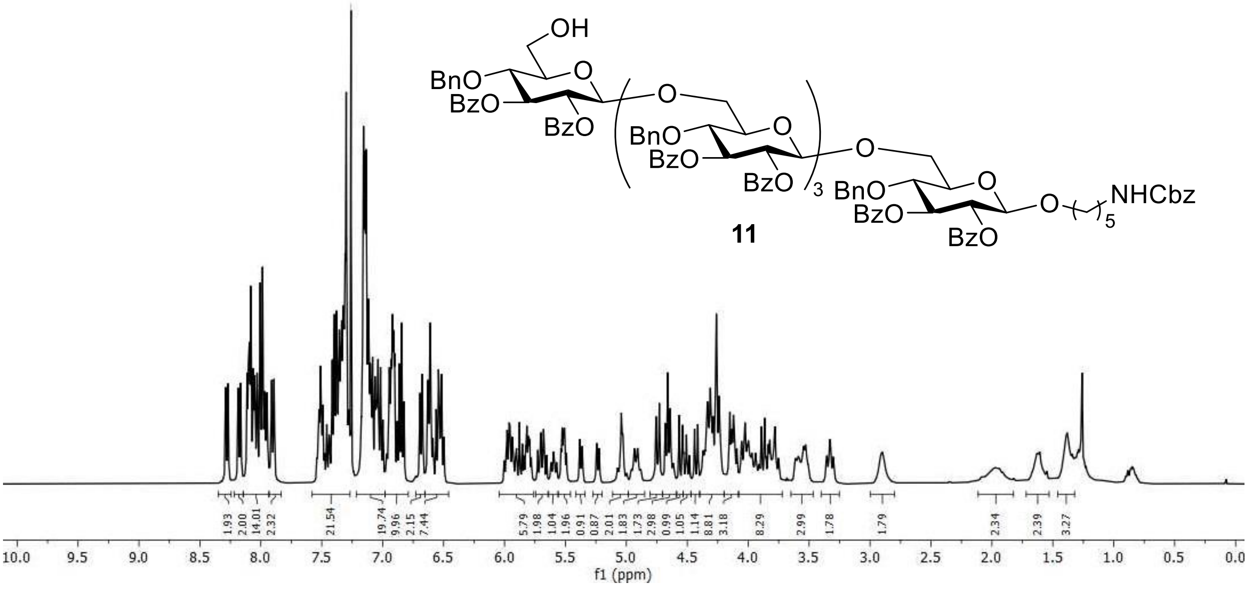

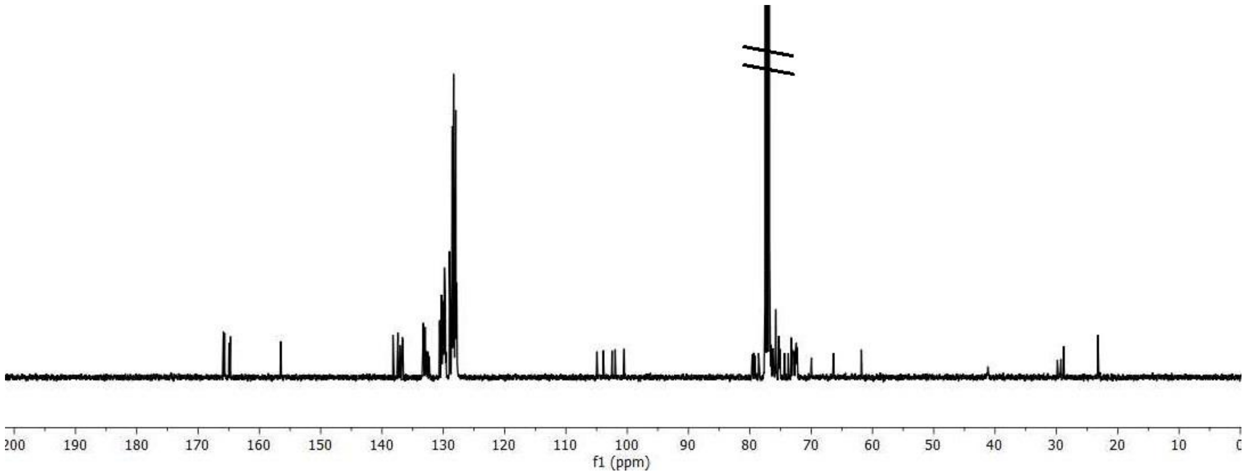
200 190 180 170 160 150 140 130 120 110 100 90 80 70 60 50 40 30 20 10
f1 (ppm)

HPLC (diol column, Hex/EtOAc = 80/20 → 0/100) of crude heptasaccharide **12**

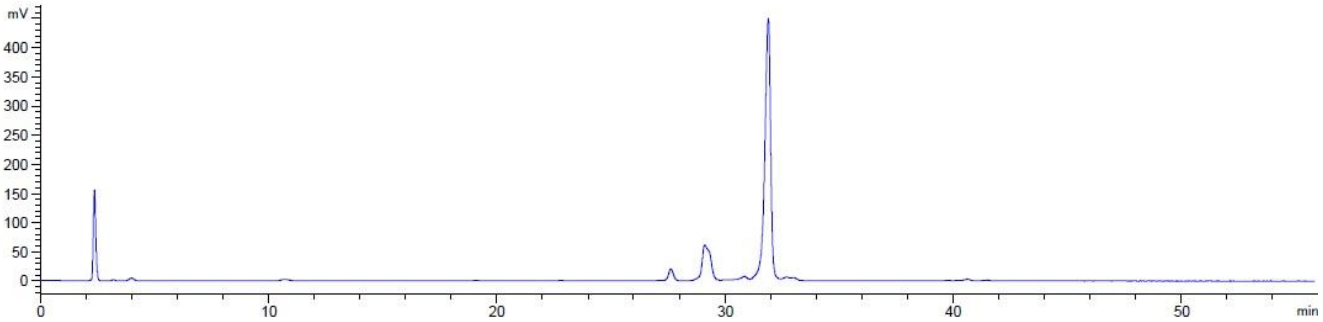

^{1}H and ^{13}C NMR spectra of purified heptasaccharide **12**

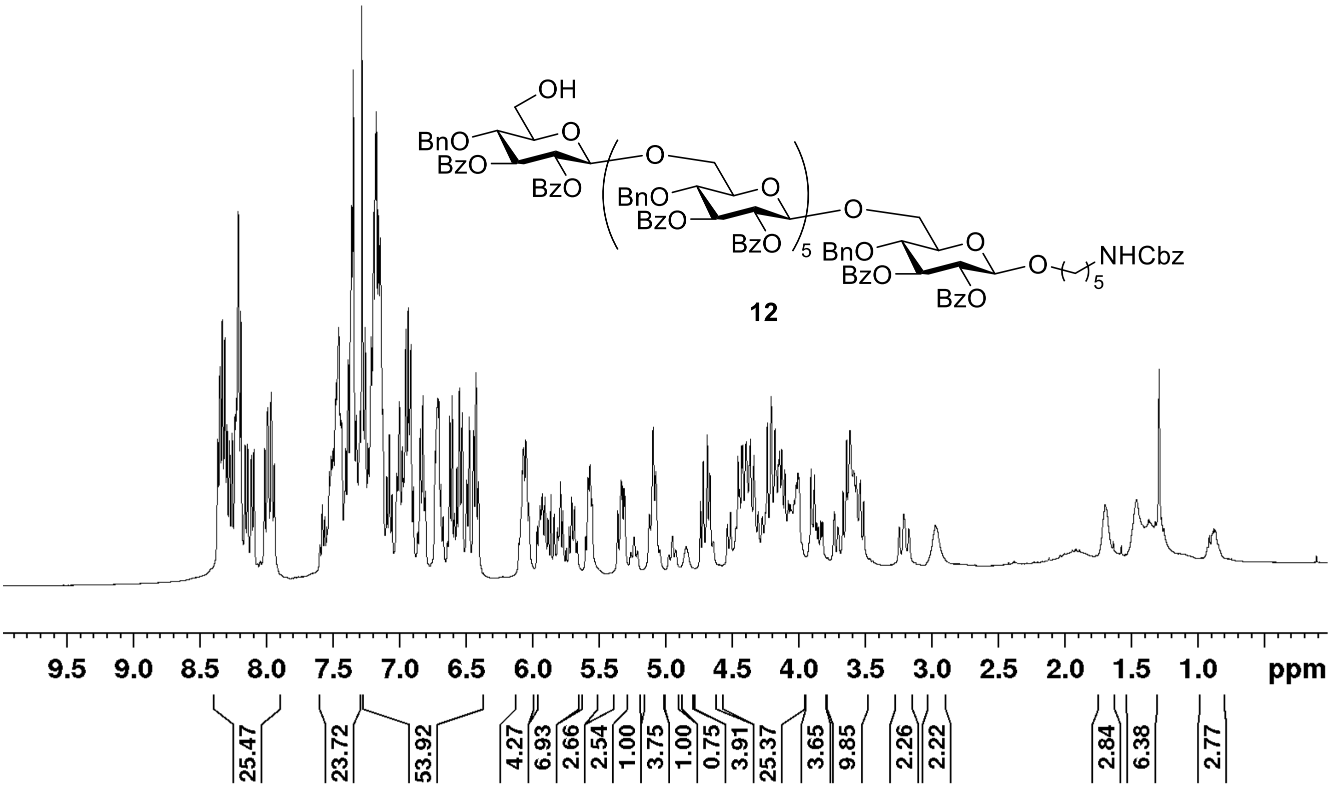

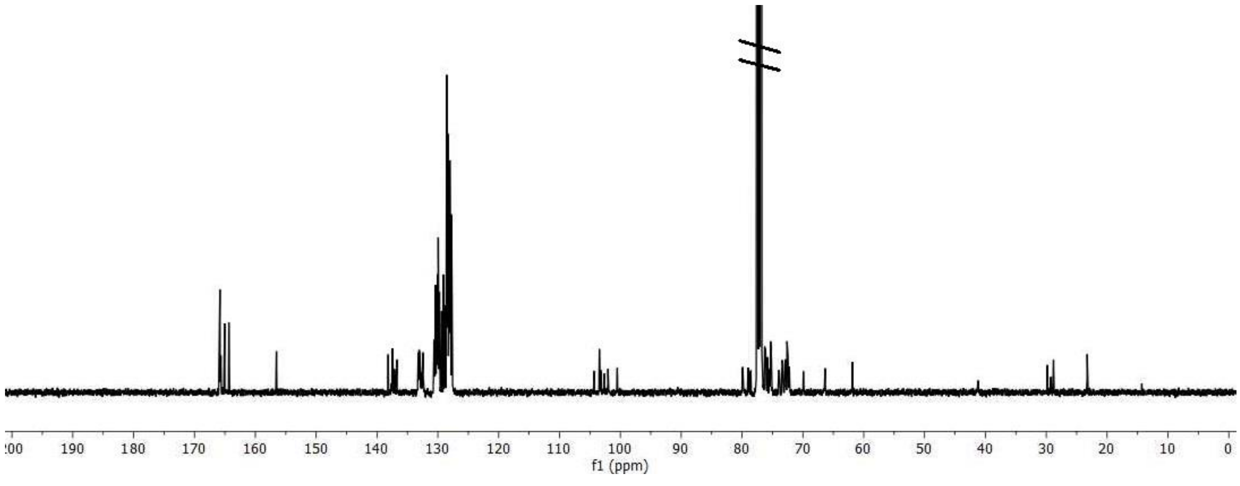

^{1}H, ^{13}C and HSQC NMR spectra of trisaccharide **13**

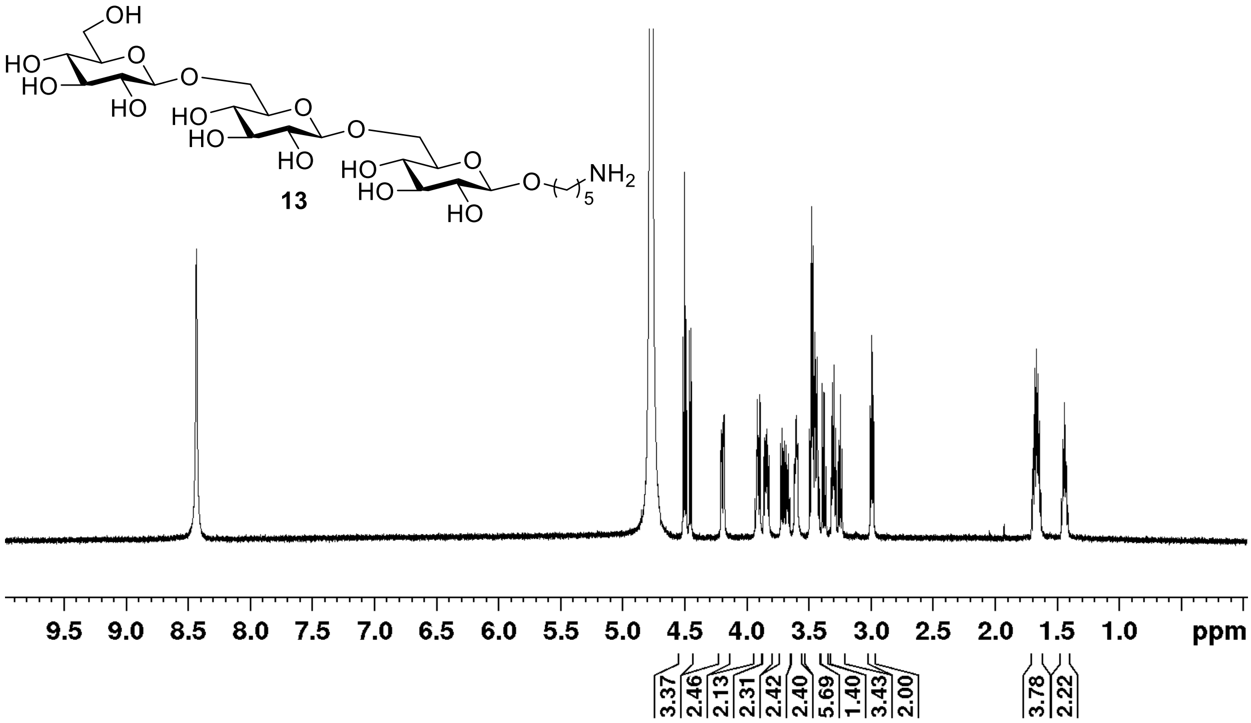

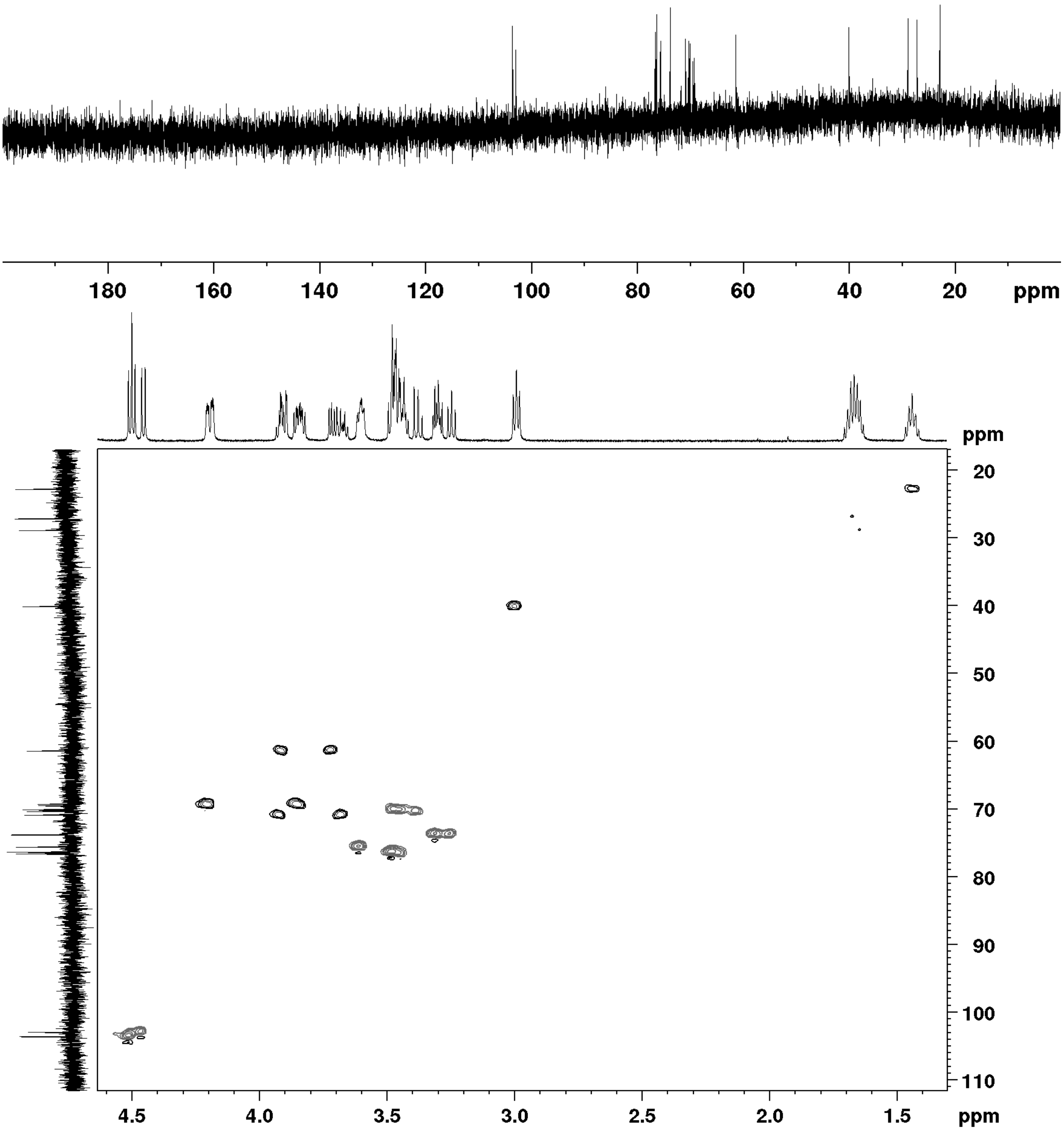
180
160
140
120
100
80
60
40
20
ppm
ppm
20
30
40
50
60
70
80
90
100
110
4.5
4.0
3.5
3.0
2.5
2.0
1.5
ppm

HPLC (ZIC-HILIC column; MeCN/water/HCOOH = 95/5/0.1 → 40/60/0.1) of trisaccharide **13**

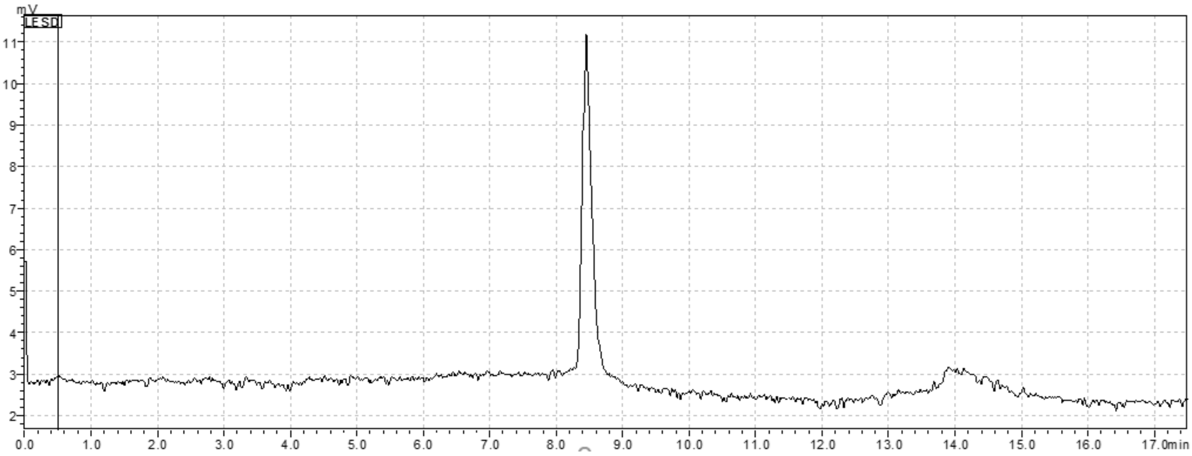

^{1}H, ^{13}C and HSQC NMR spectra of pentasaccharide **14**

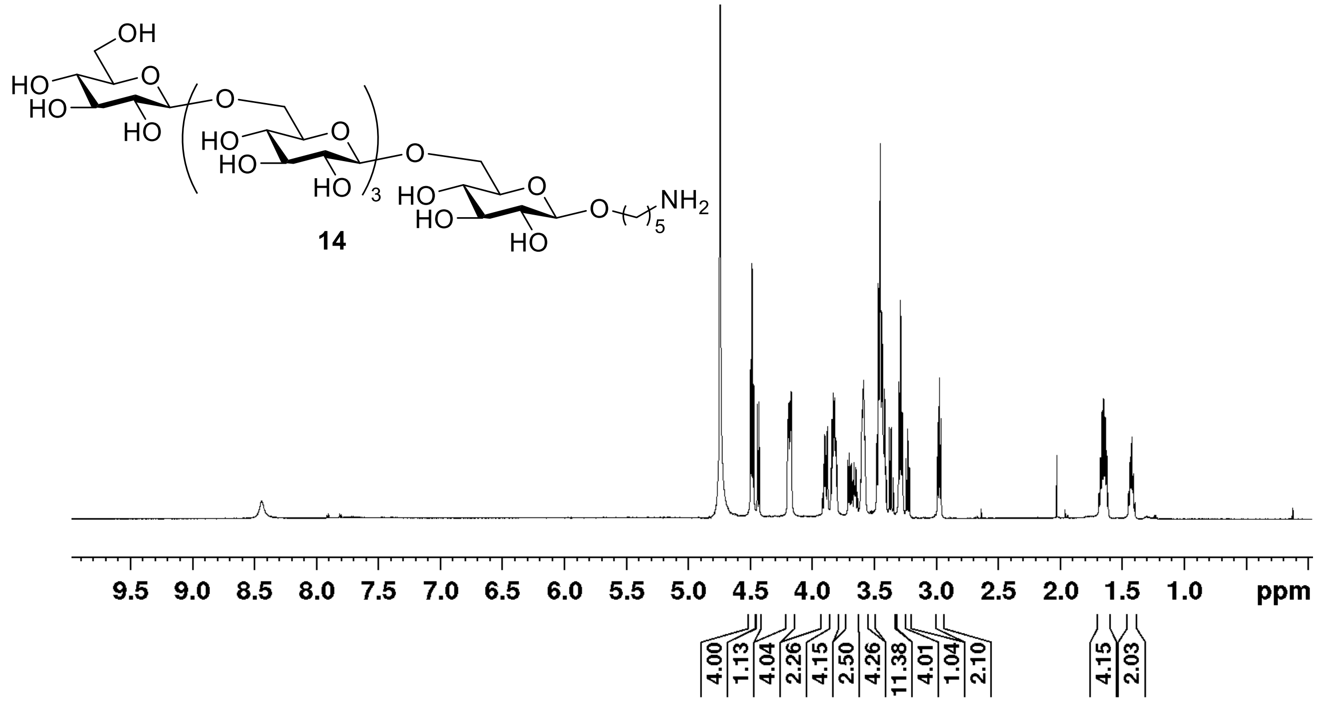

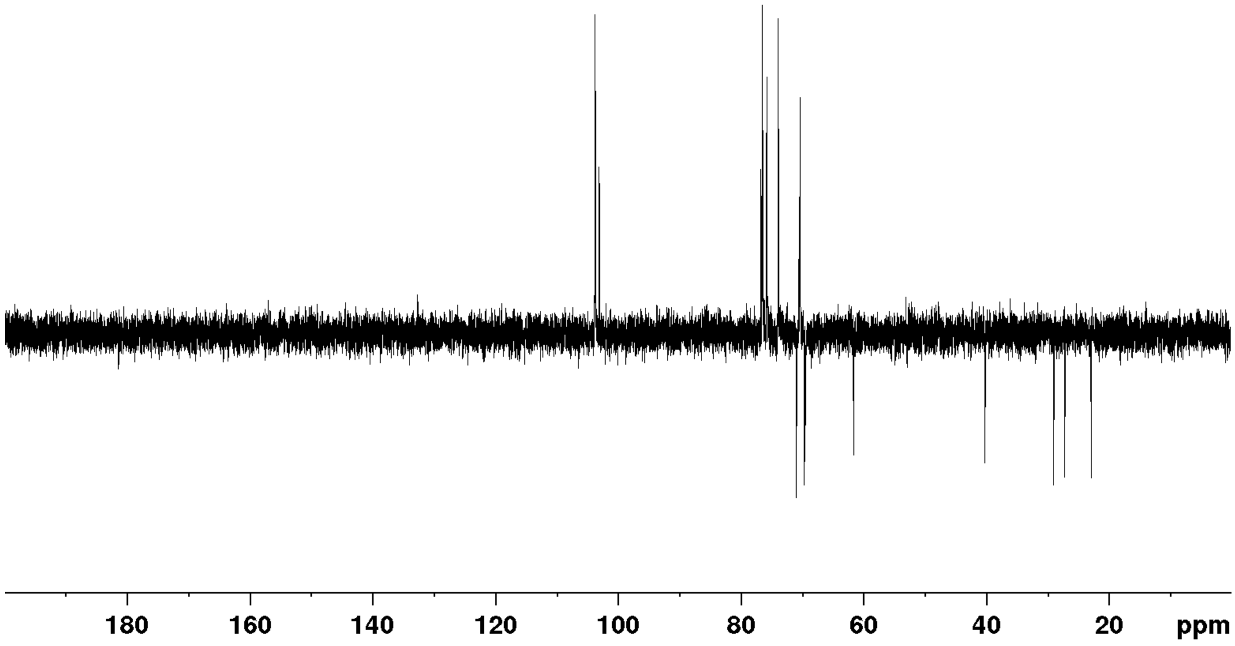

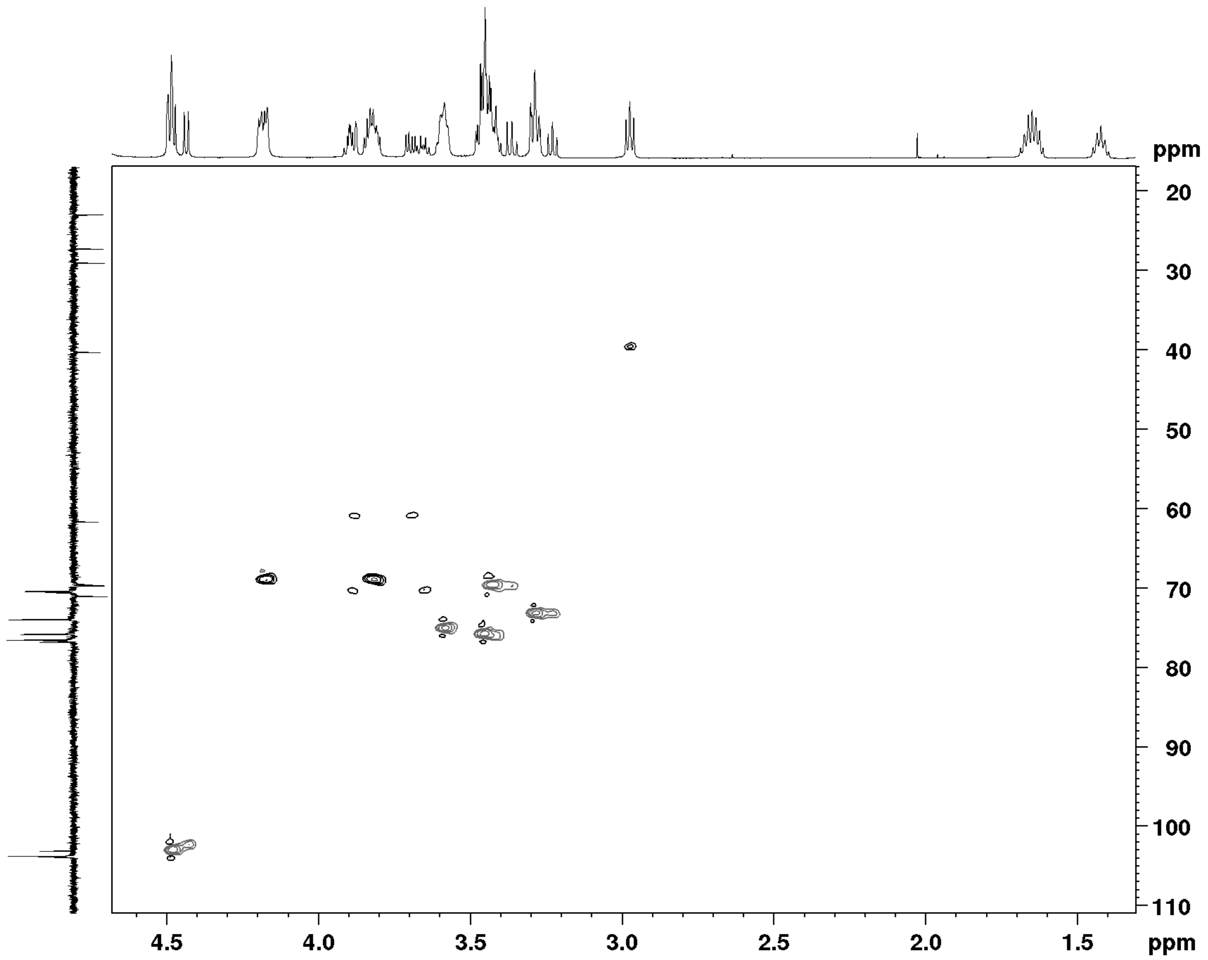

HPLC (ZIC-HILIC column; MeCN/water/HCOOH = 95/5/0.1 → 40/60/0.1) of pure pentasaccharide **14**

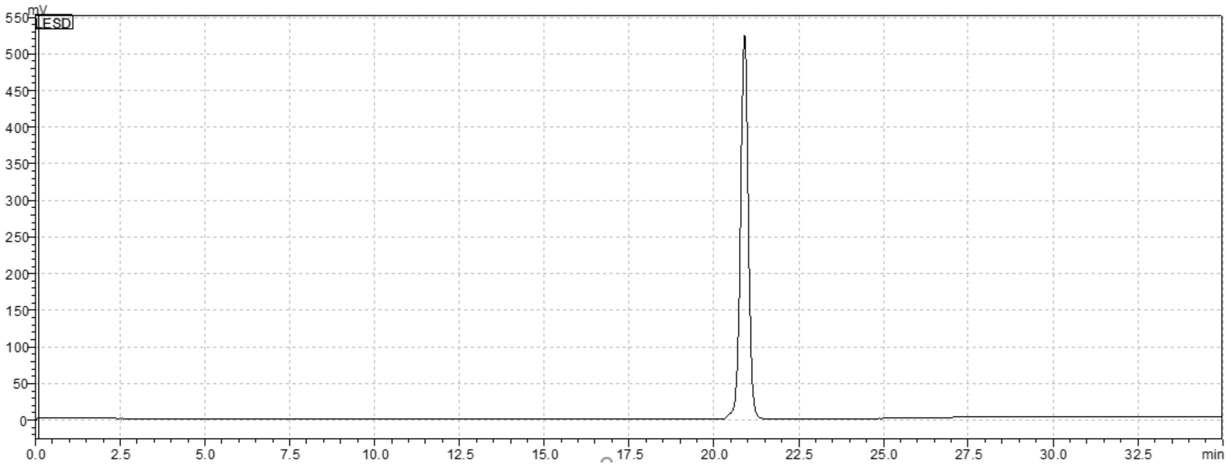

^{1}H, ^{13}C and HSQC NMR spectra of heptasaccharide **15**

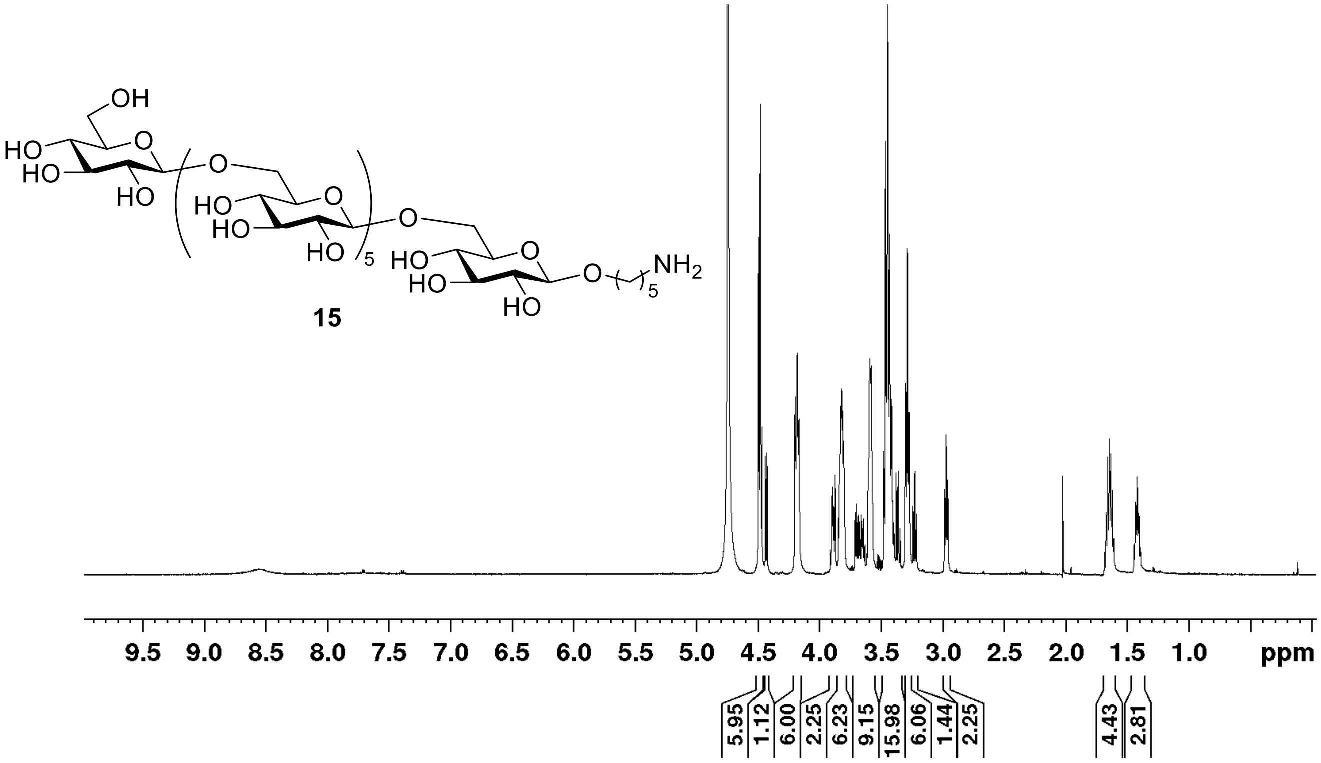

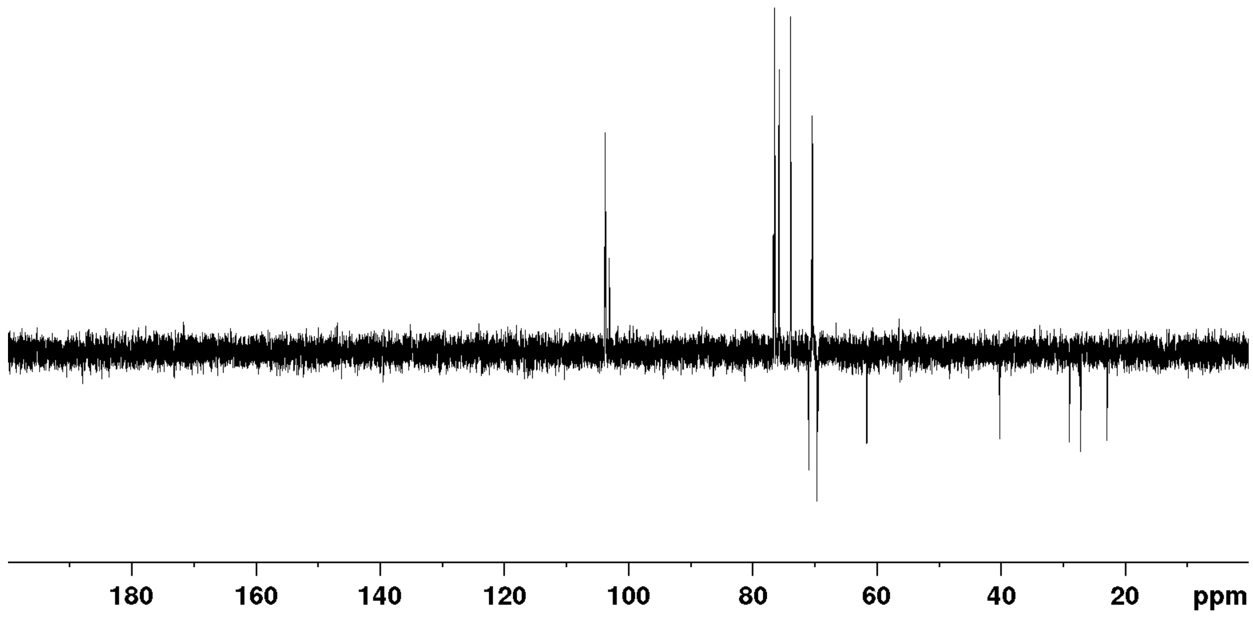

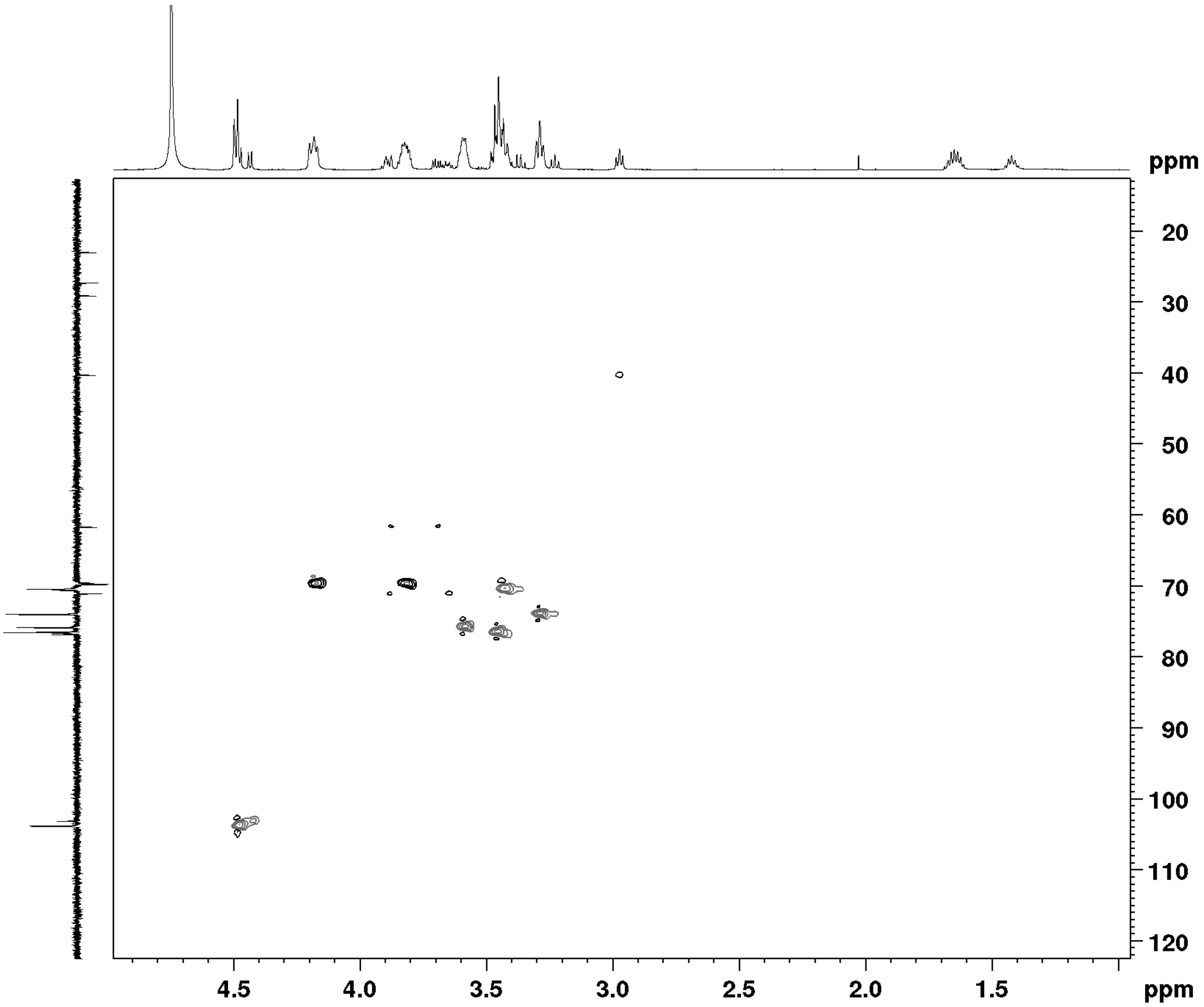

HPLC (ZIC-HILIC column MeCN/water/HCOOH = 95/5/0.1 → 40/60/0.1) of pure heptasaccharide **15**:

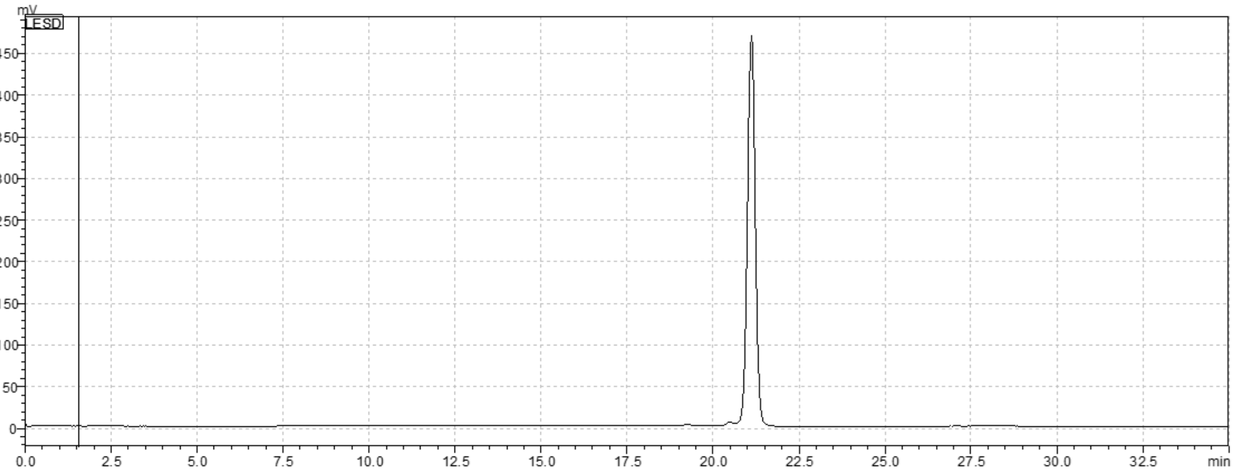

1.4 Analytical data of α-[1→3]-series

^{1}H and ^{13}C NMR of compound **18**

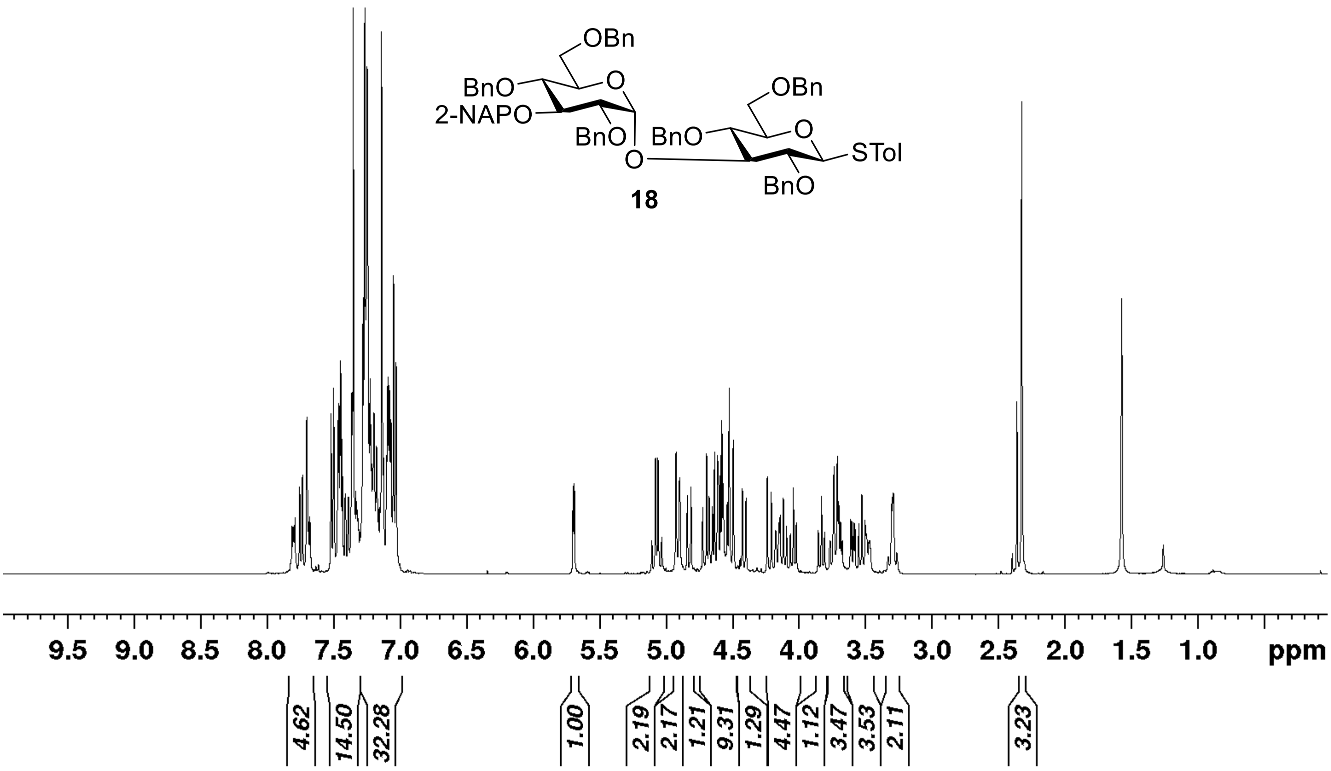

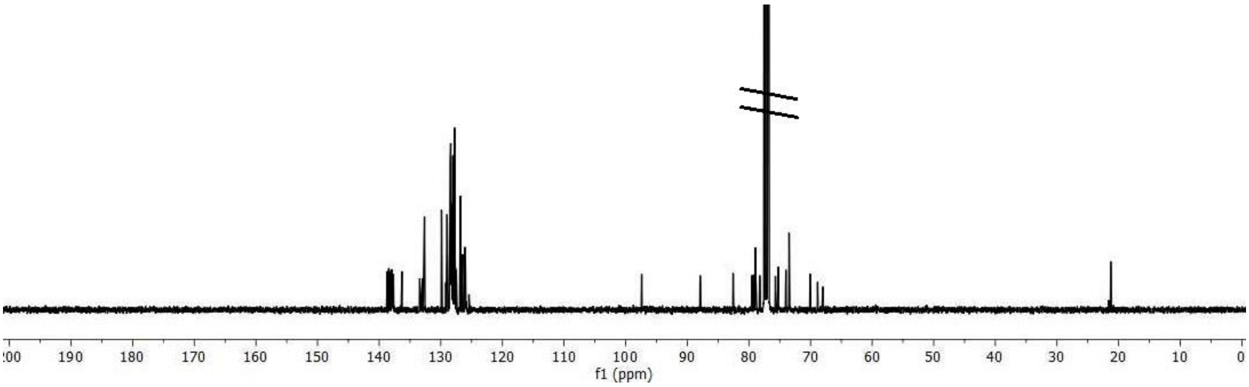

^{1}H and ^{13}C NMR of compound **19**

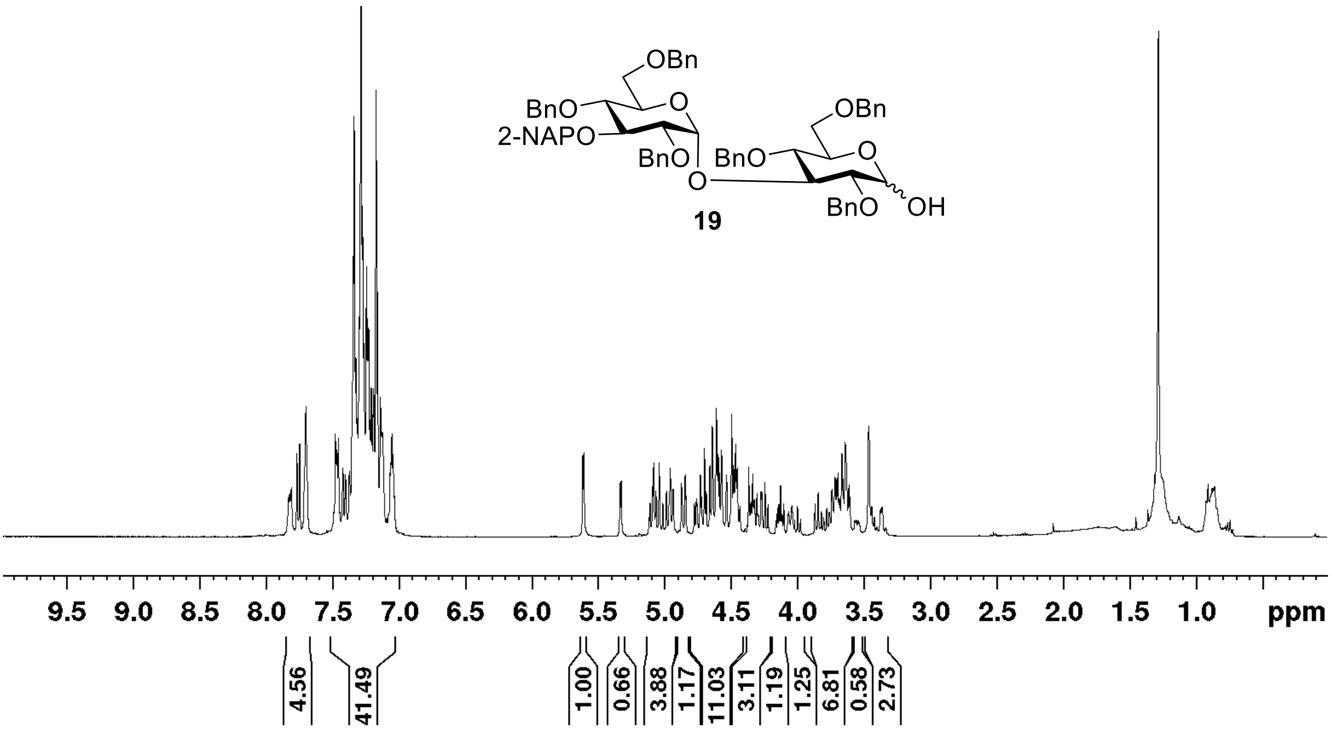

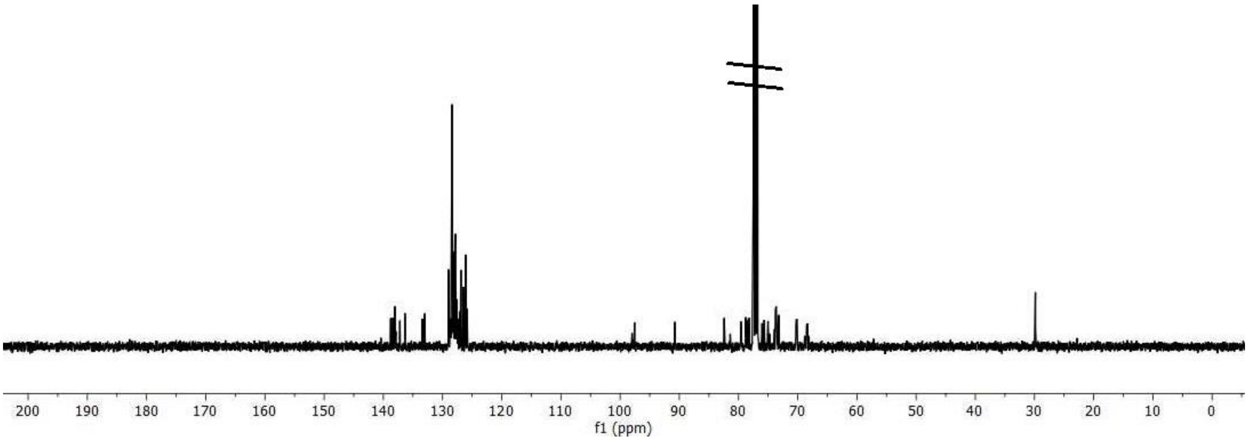

^{1}H and ^{13}C NMR of compound **20**

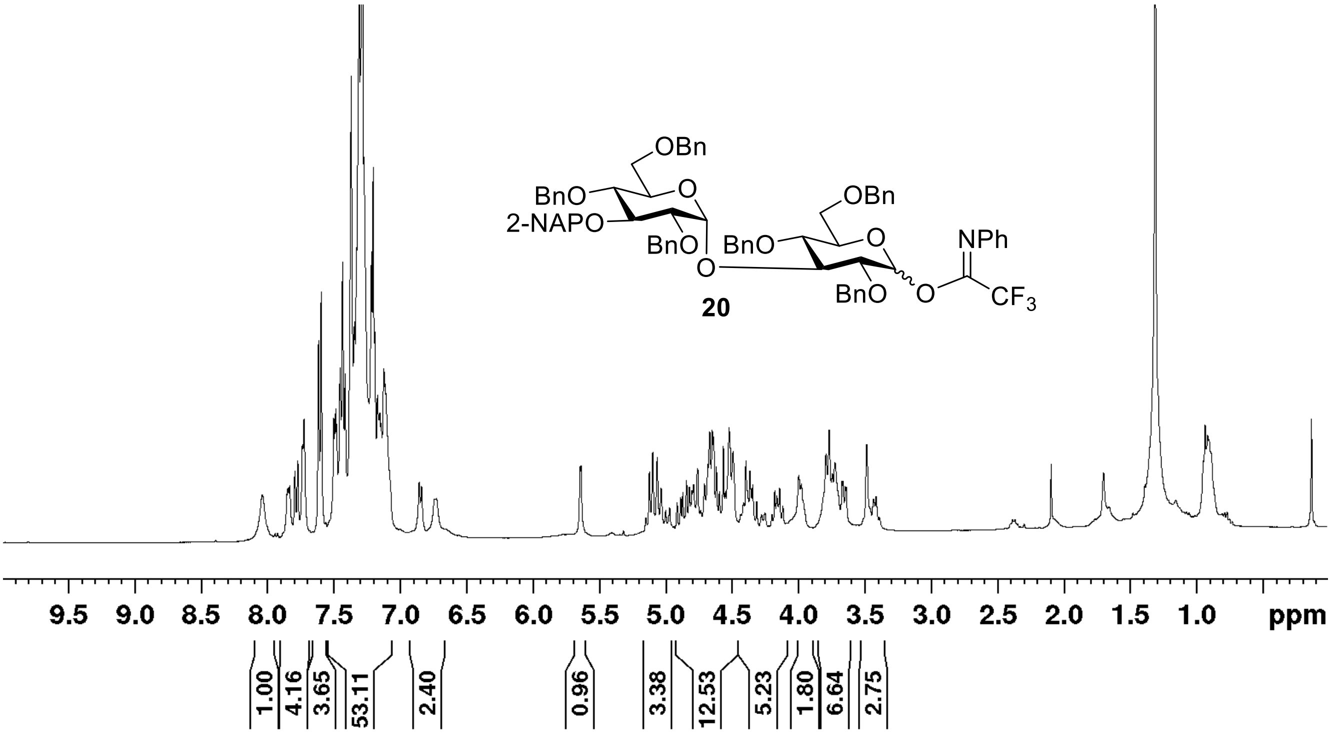

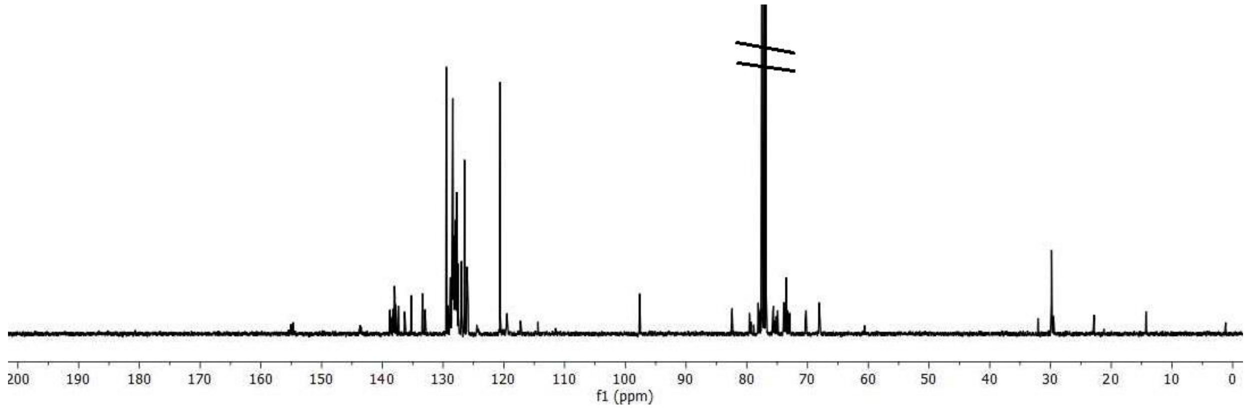

^{1}H and ^{13}C NMR of compound **21**

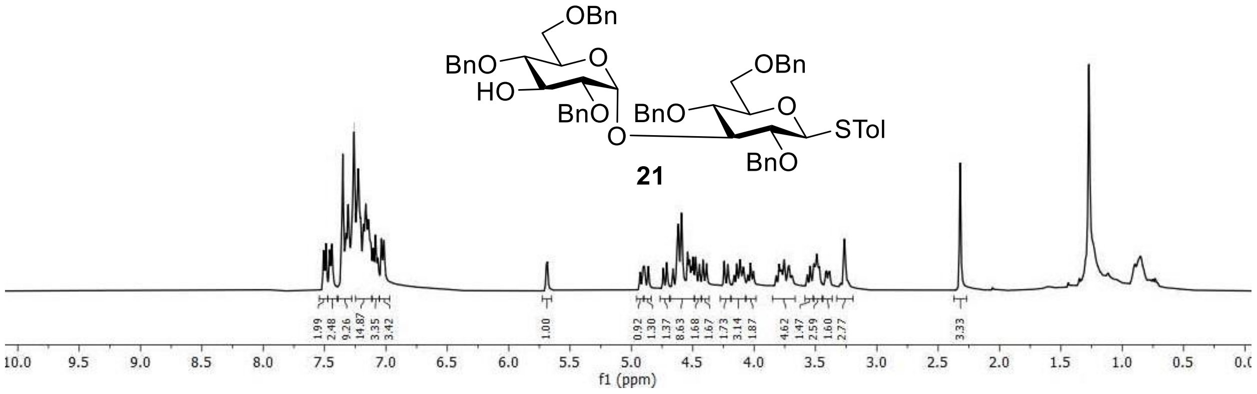

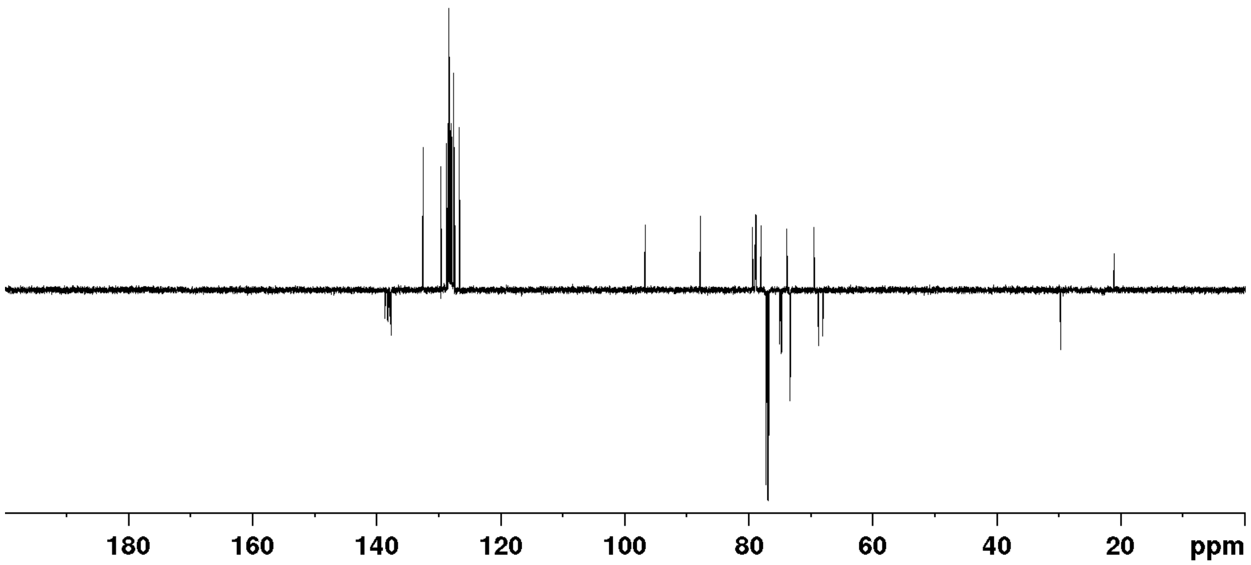

^{1}H and ^{13}C NMR of compound **22**

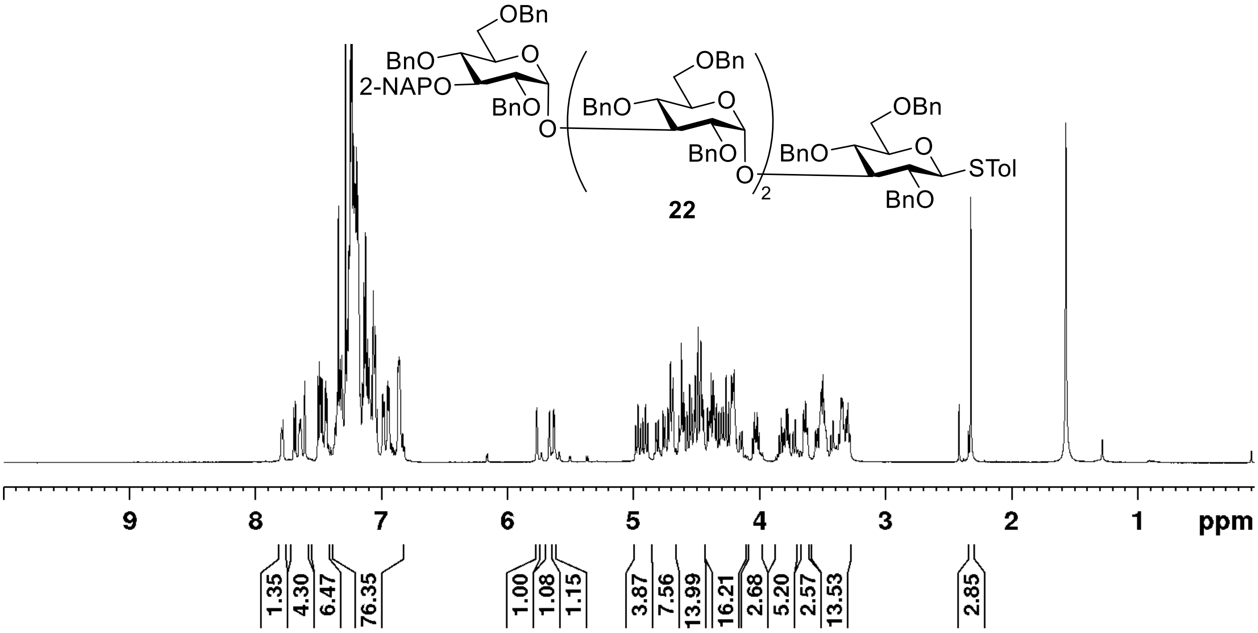

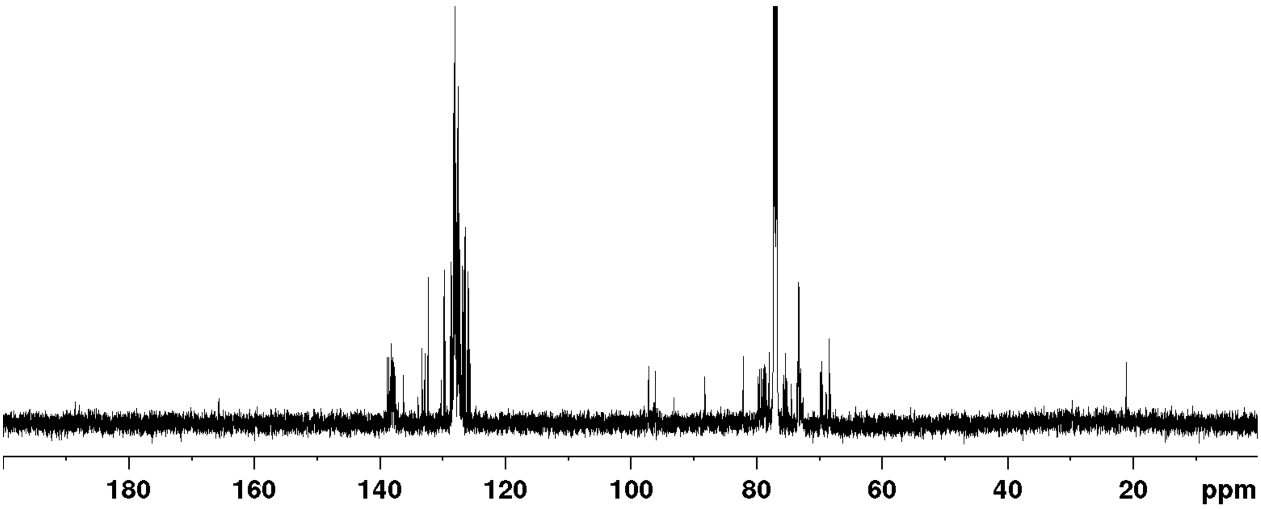

^{1}H and ^{13}C NMR of compound **23**

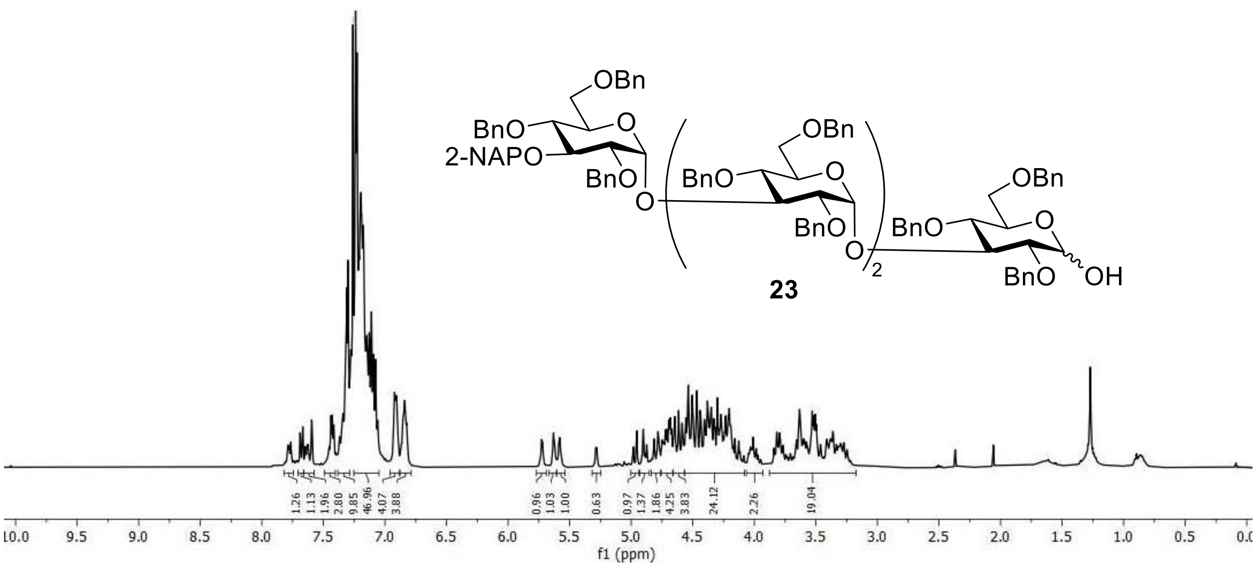

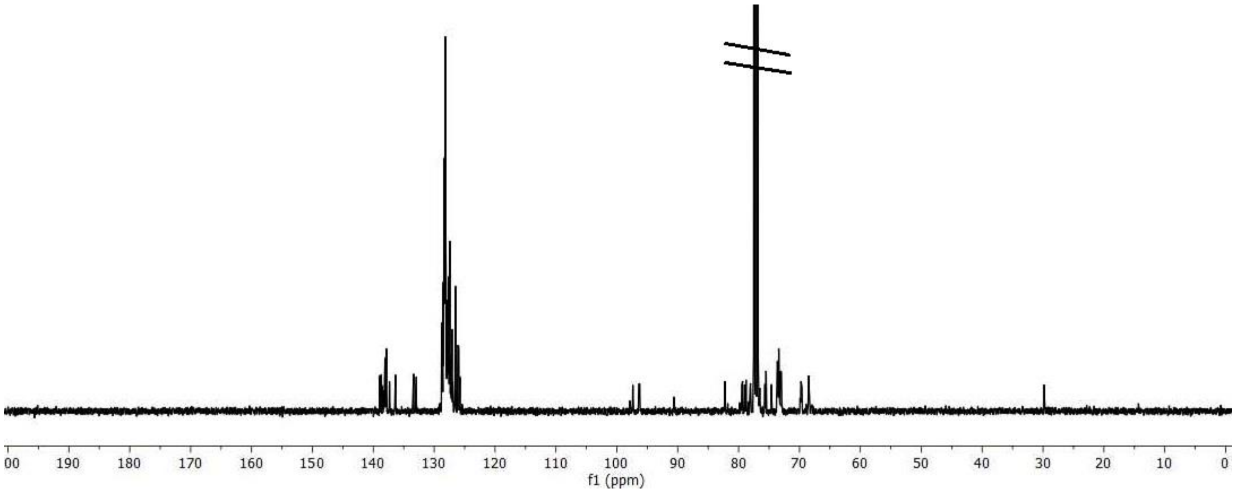

^1H and ^{13}C NMR of compound **24**

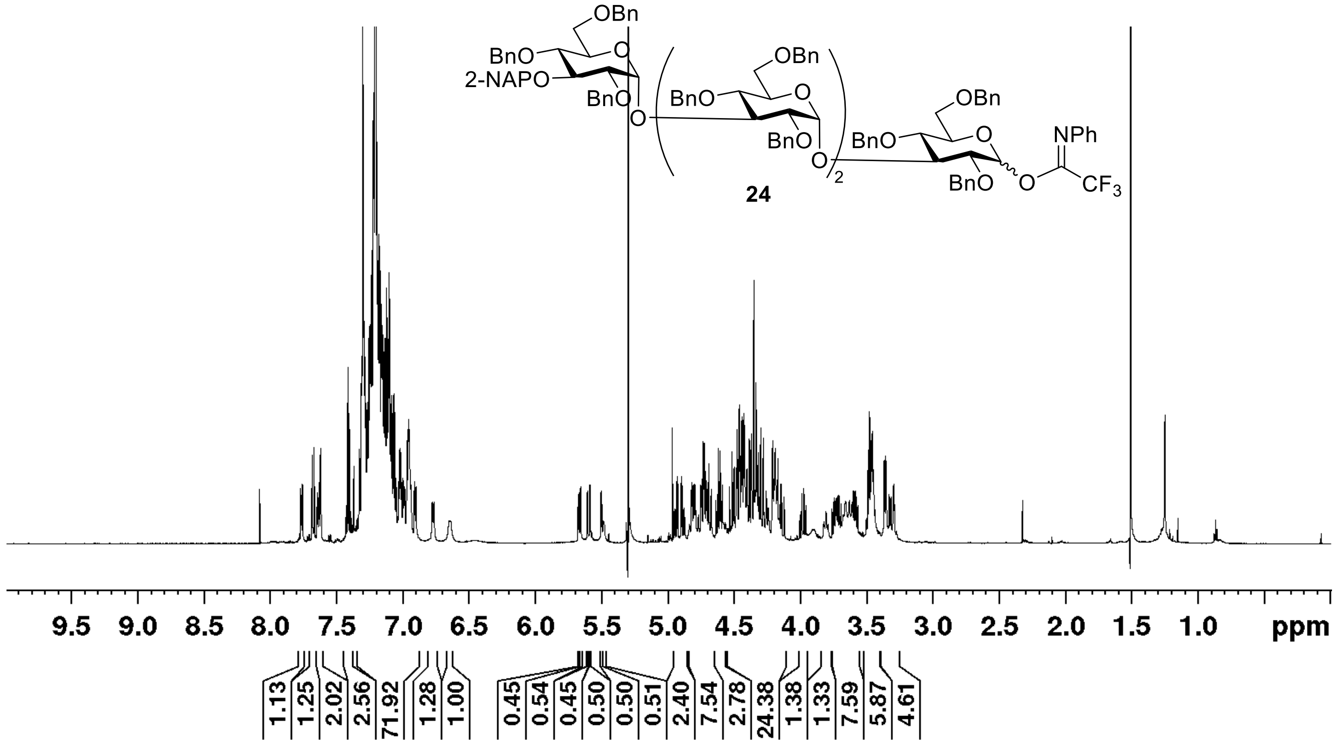

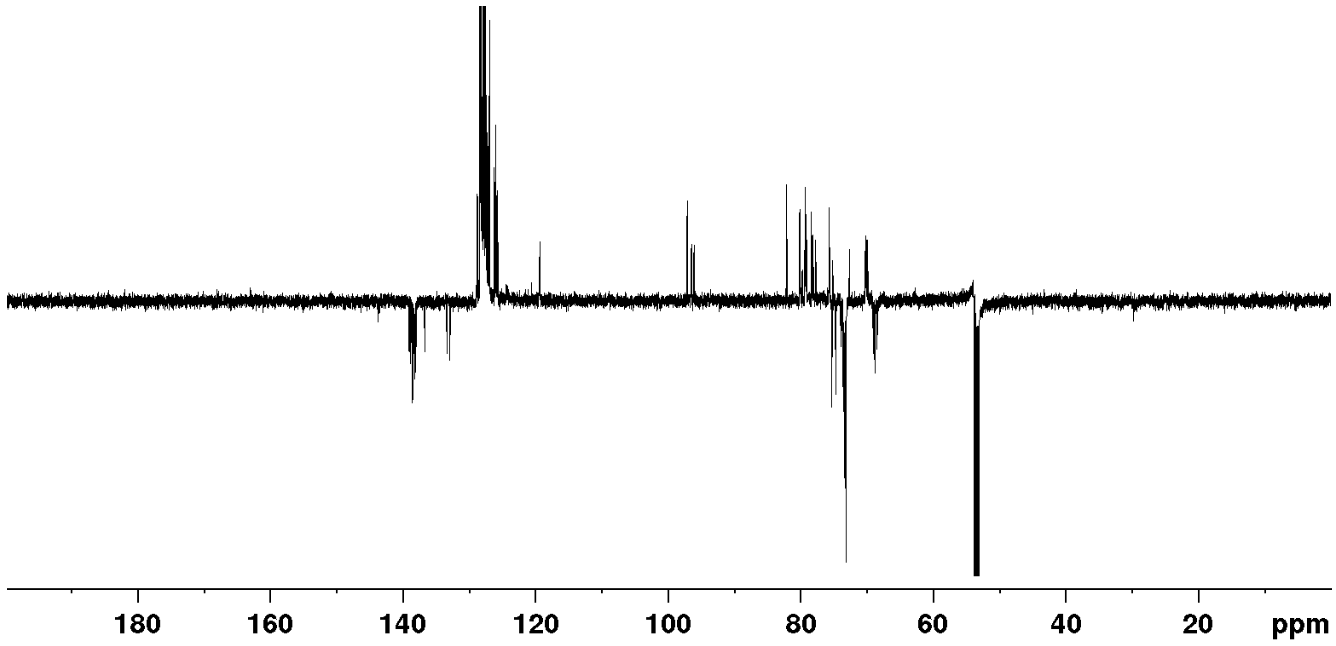

^{1}H and ^{13}C NMR of compound **25**

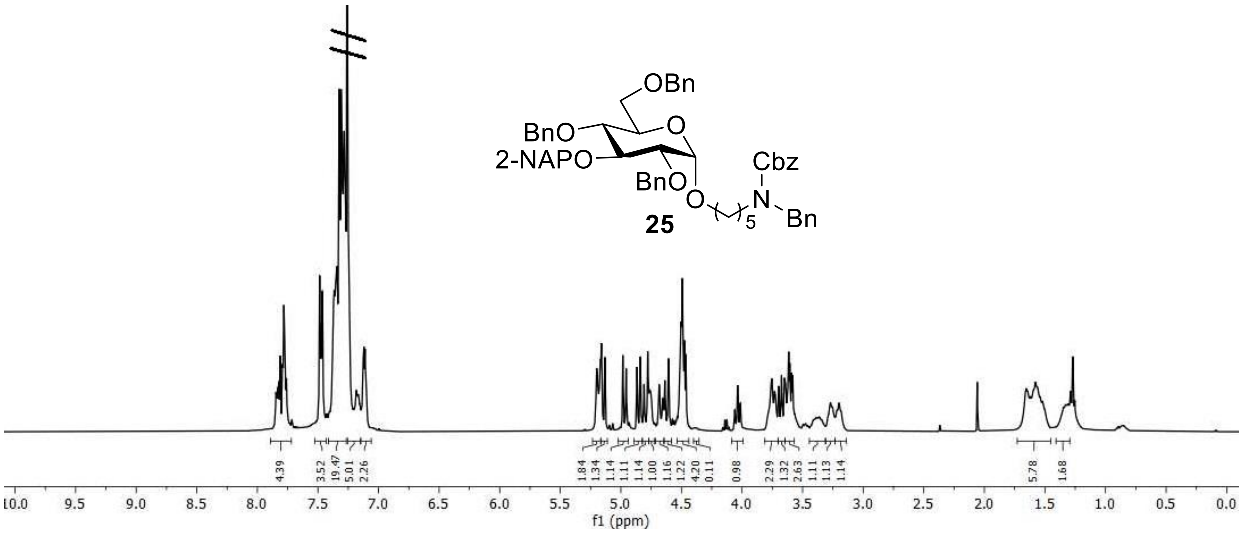

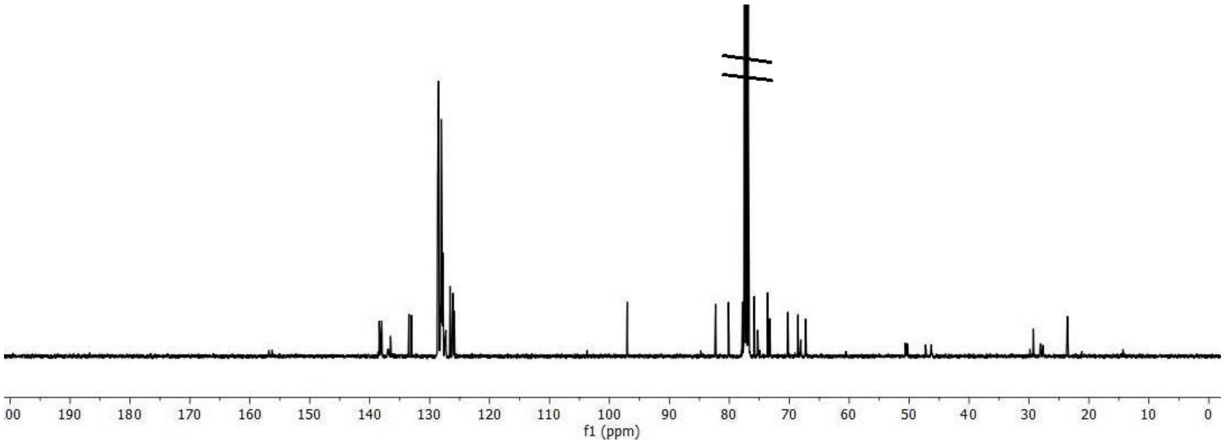

^{1}H and ^{13}C NMR of compound **26**

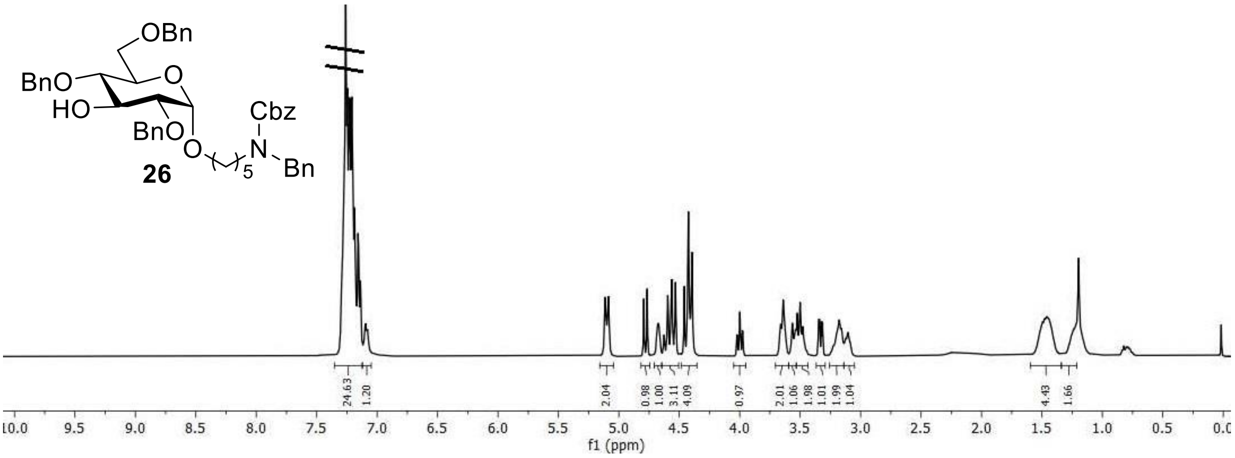

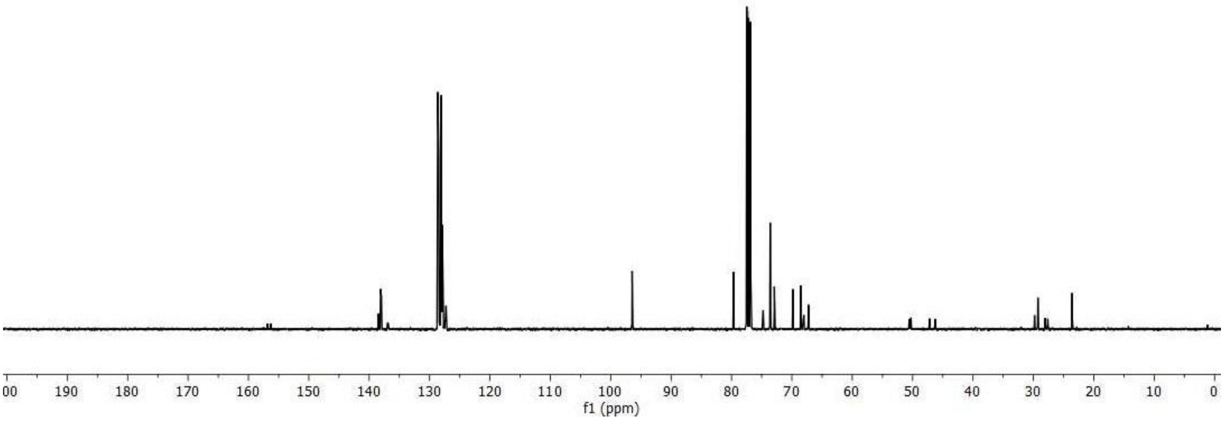

^{1}H and ^{13}C NMR of compound **27**

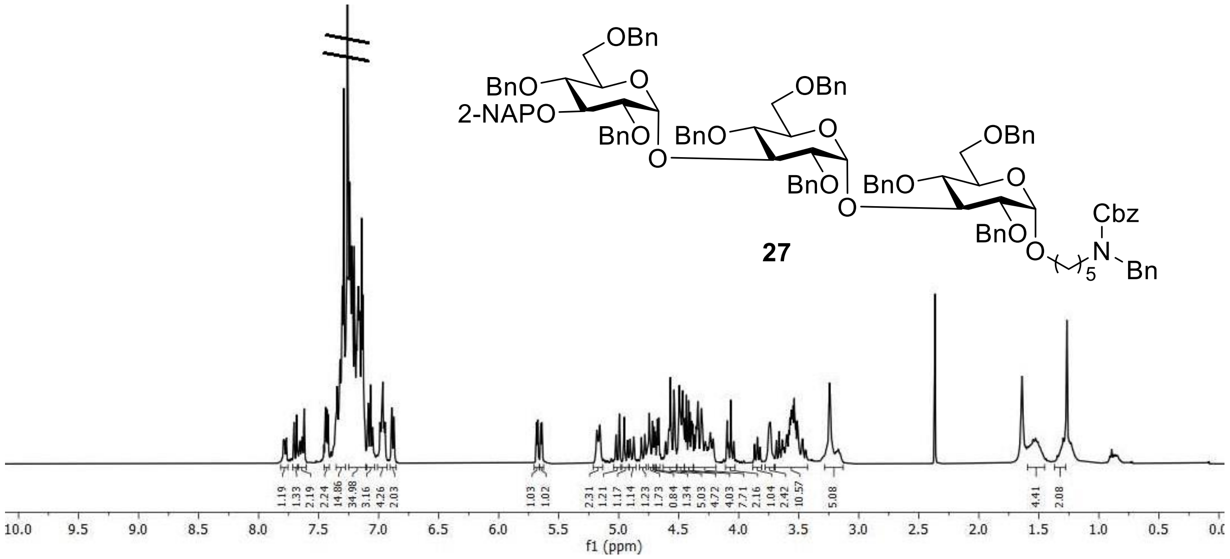

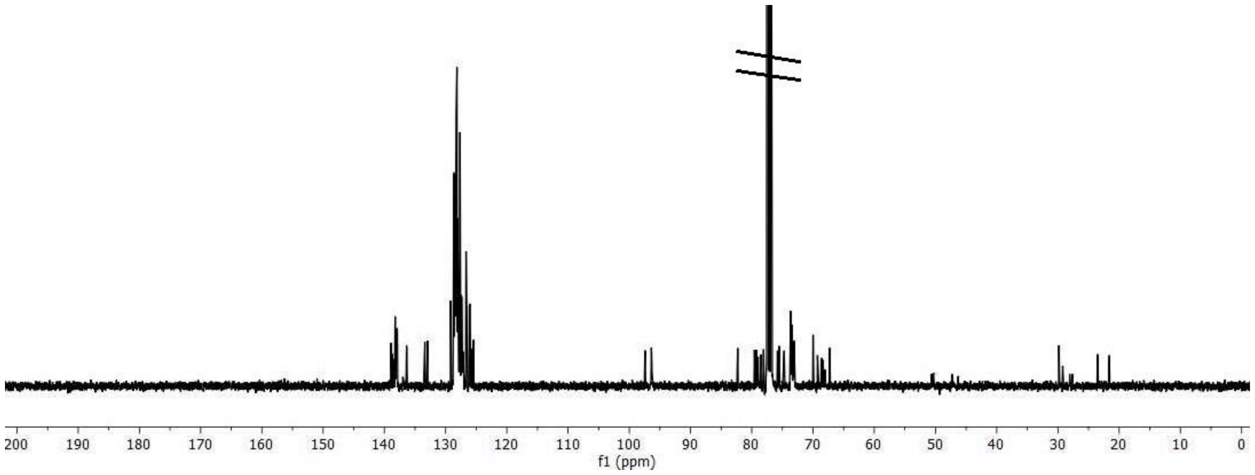

^{1}H and ^{13}C NMR of compound **28**

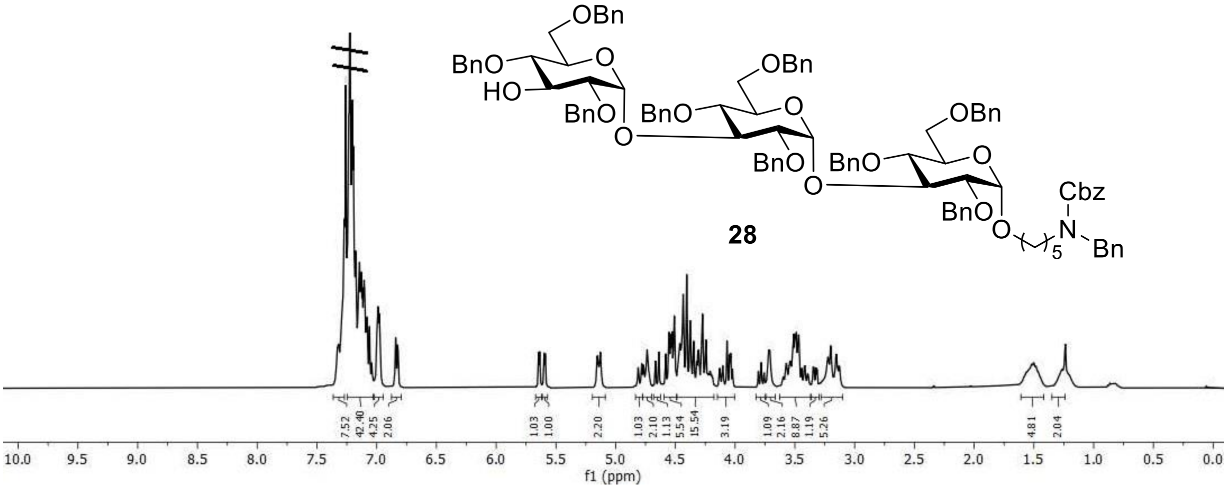

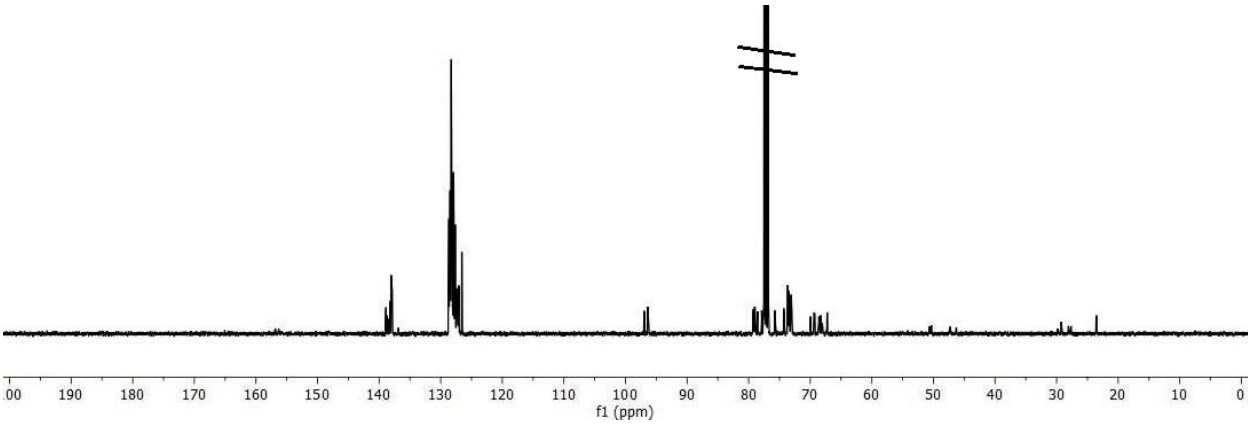

^{1}H and ^{13}C NMR of compound **29**

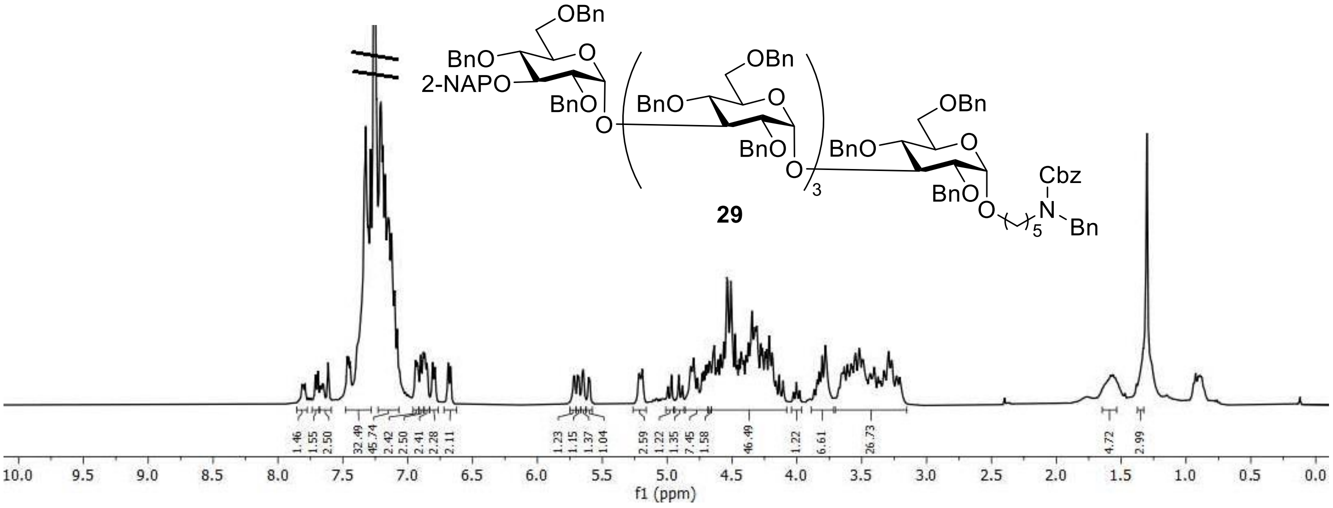

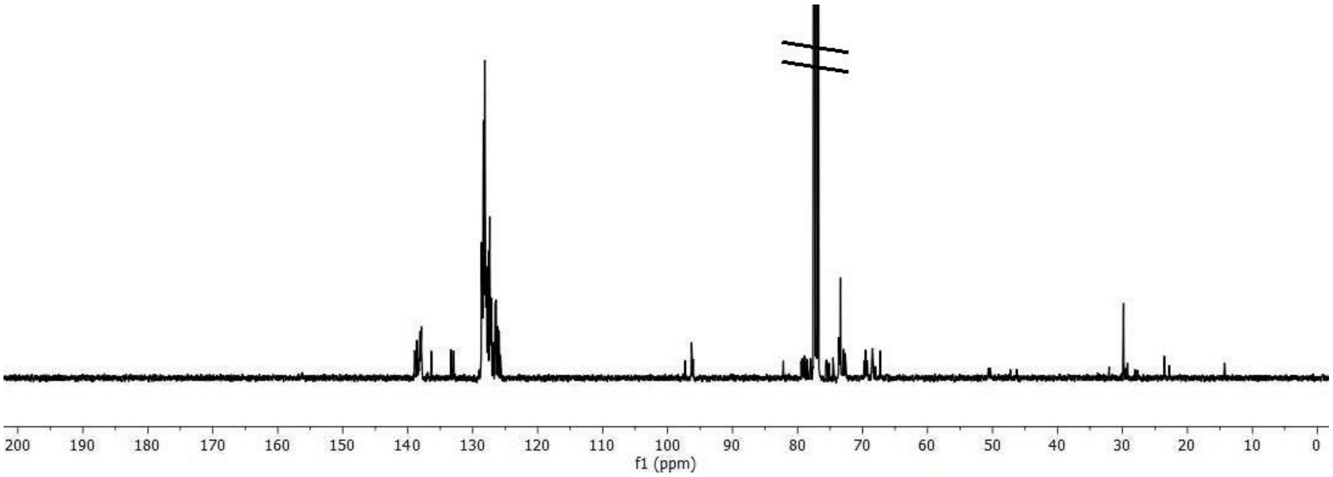

^{1}H and ^{13}C NMR of compound **30**

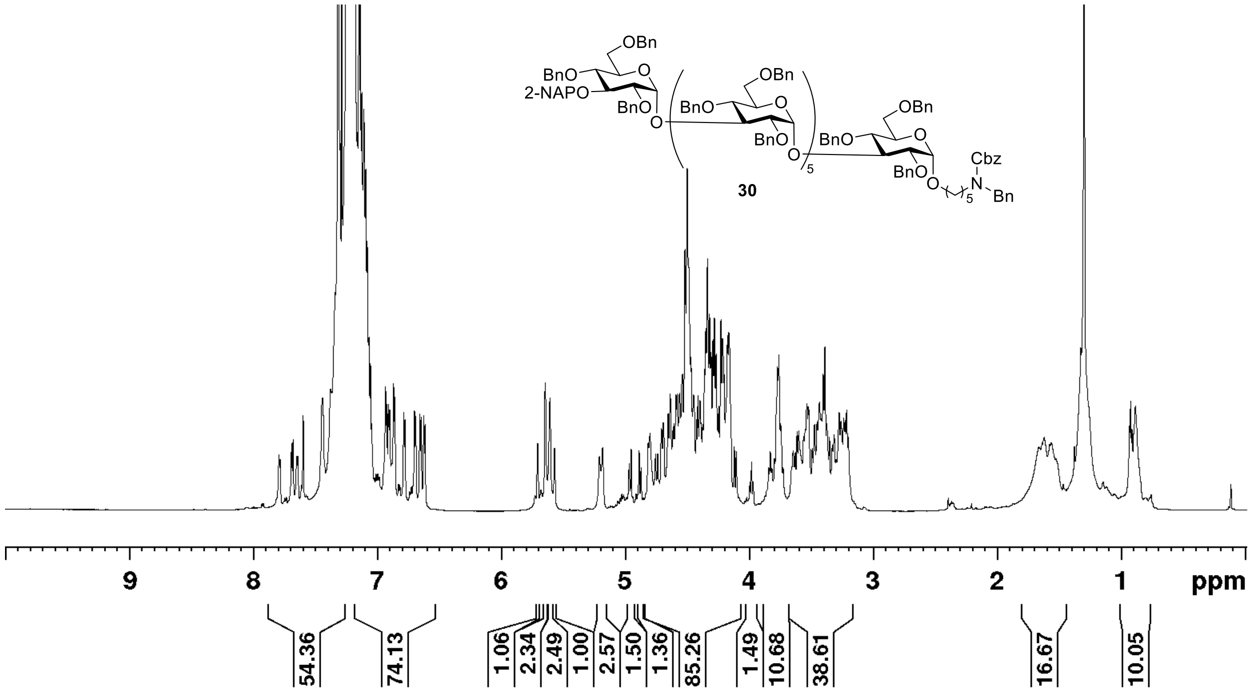

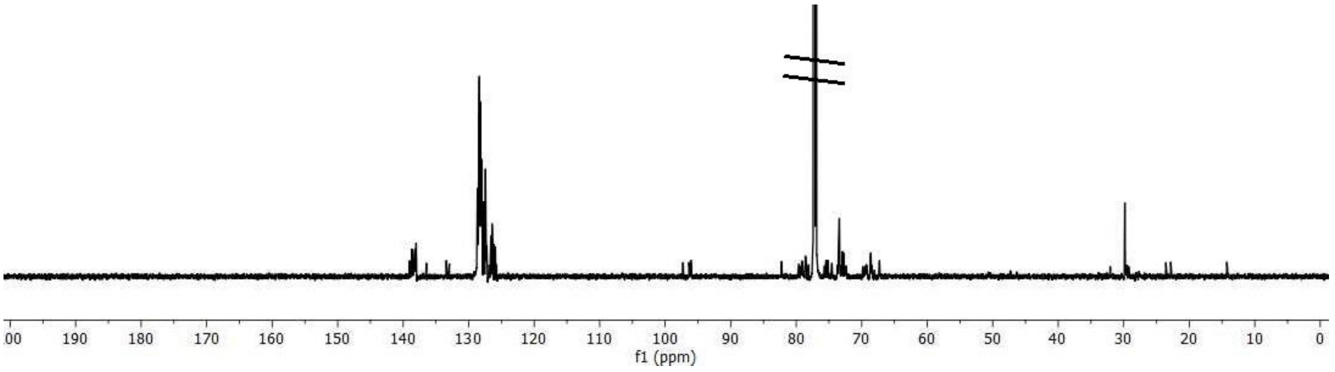

^{1}H, ^{13}C and HSQC-NMR of trisaccharide **31**

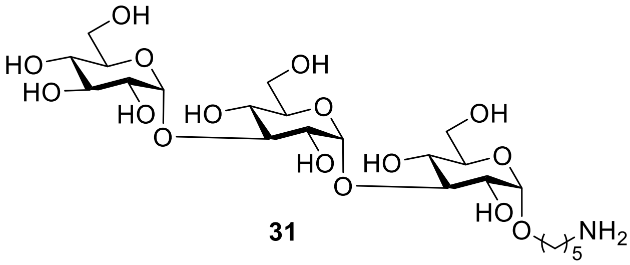

31

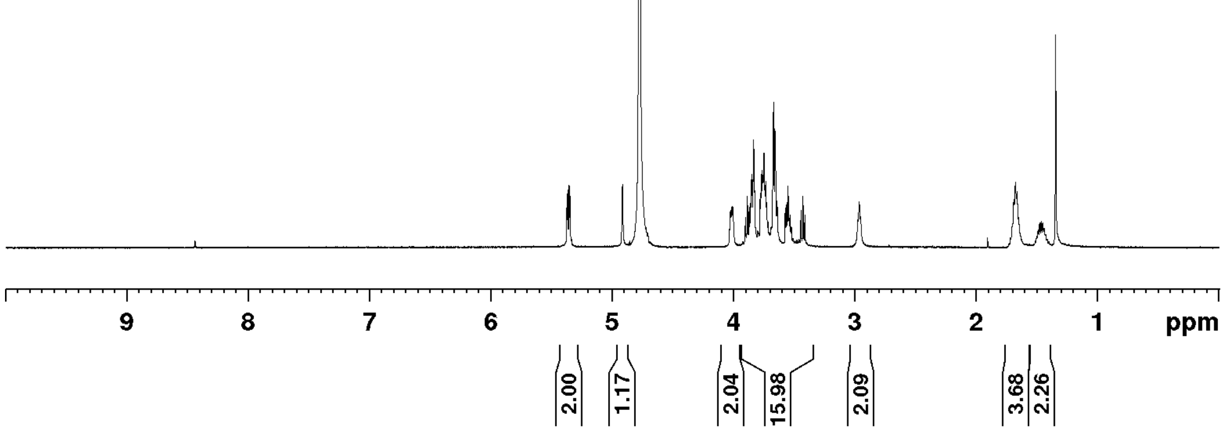

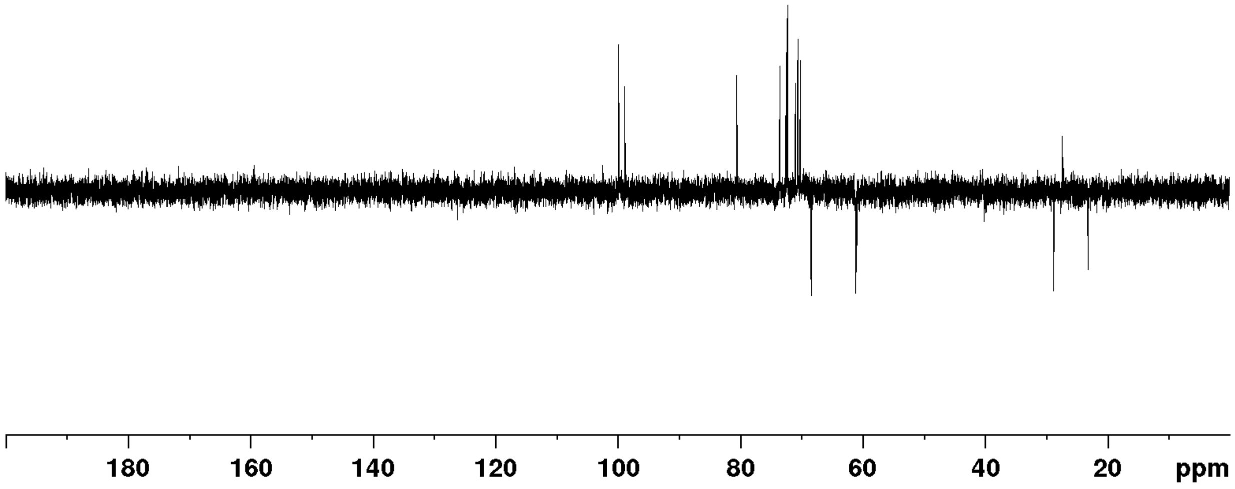

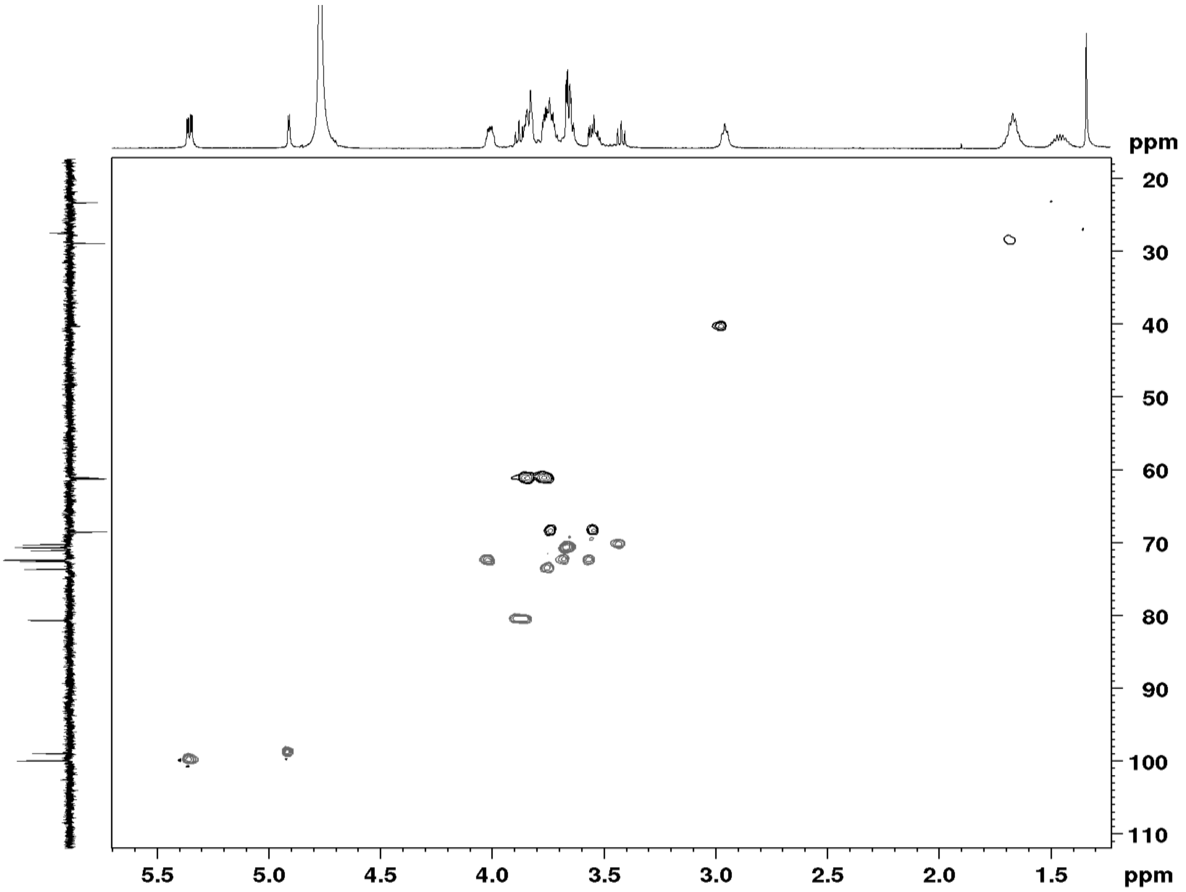

HPLC (ZIC-HILIC column; MeCN/water/HCOOH = 95/5/0.1 → 40/60/0.1) of trisaccharide **31**

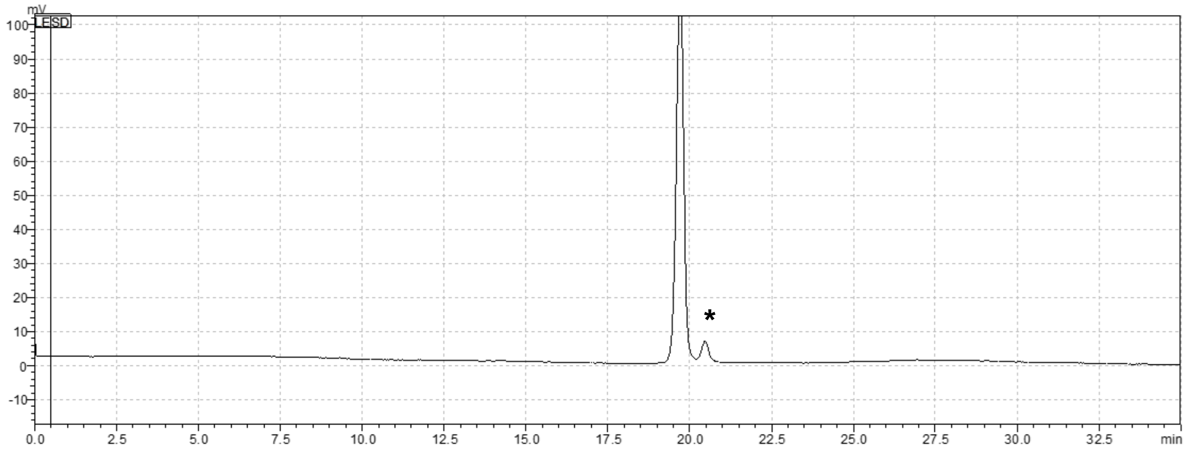

*Impurity from the ZIC-HILIC column

^{1}H, ^{13}C and HSQC-NMR of pentasaccharide **32**

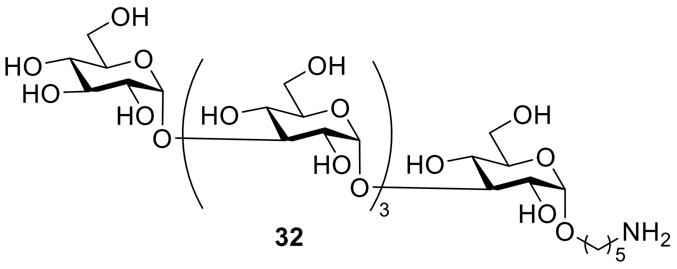

32

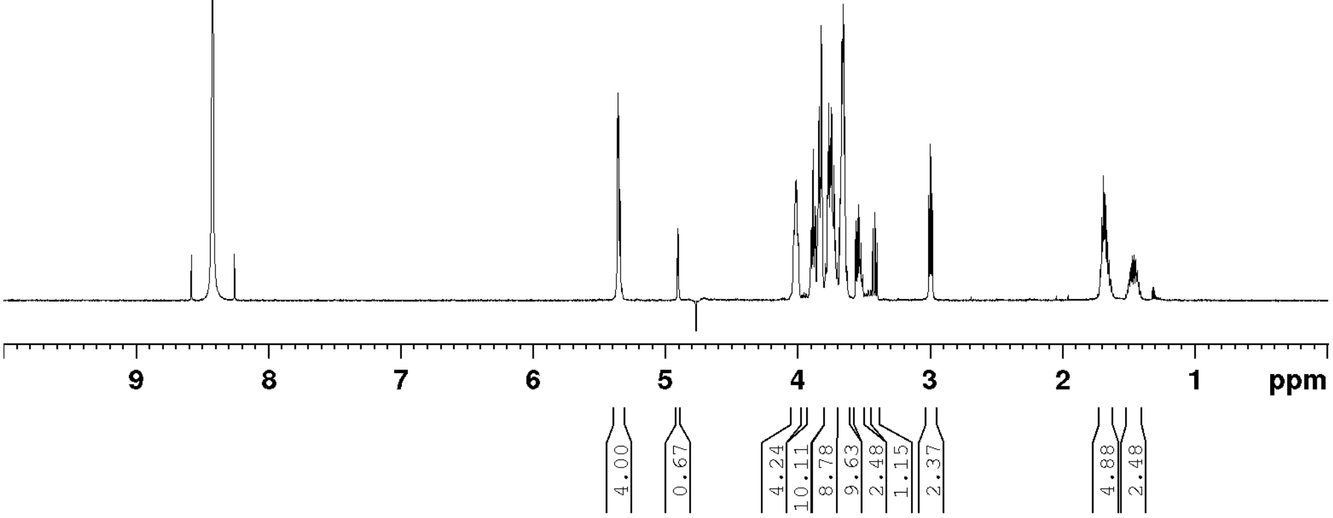

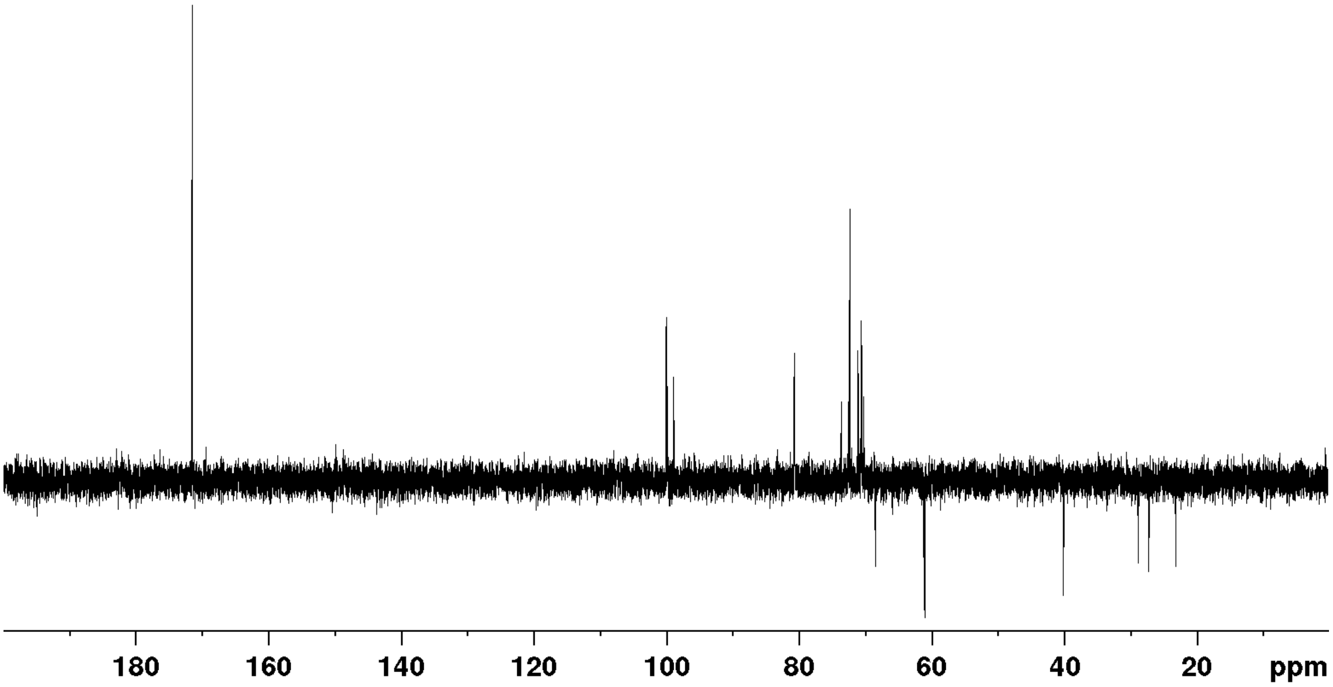

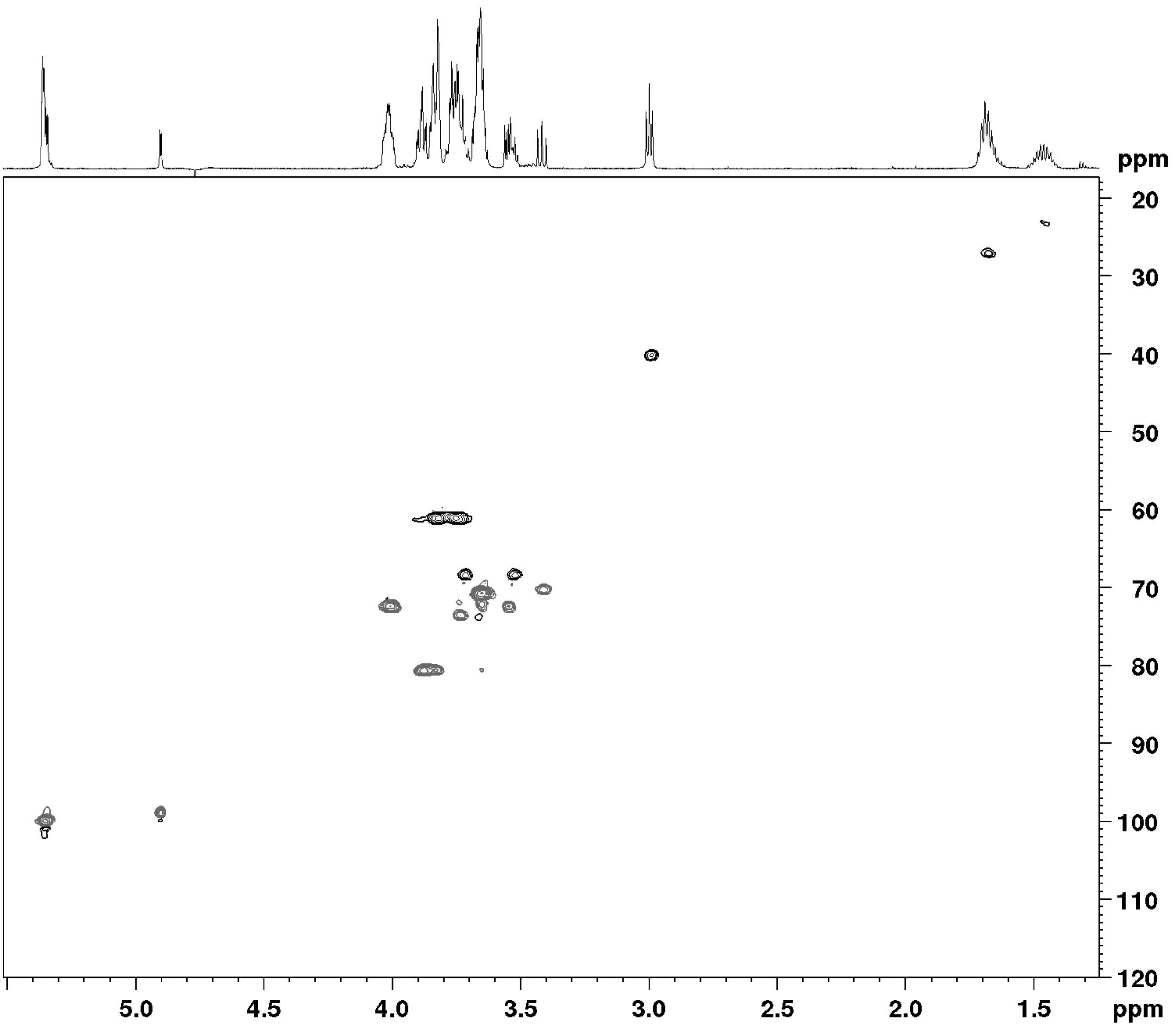

HPLC (ZIC-HILIC column MeCN/water/HCOOH = 95/5/0.1 → 40/60/0.1) of pentasaccharide **32**

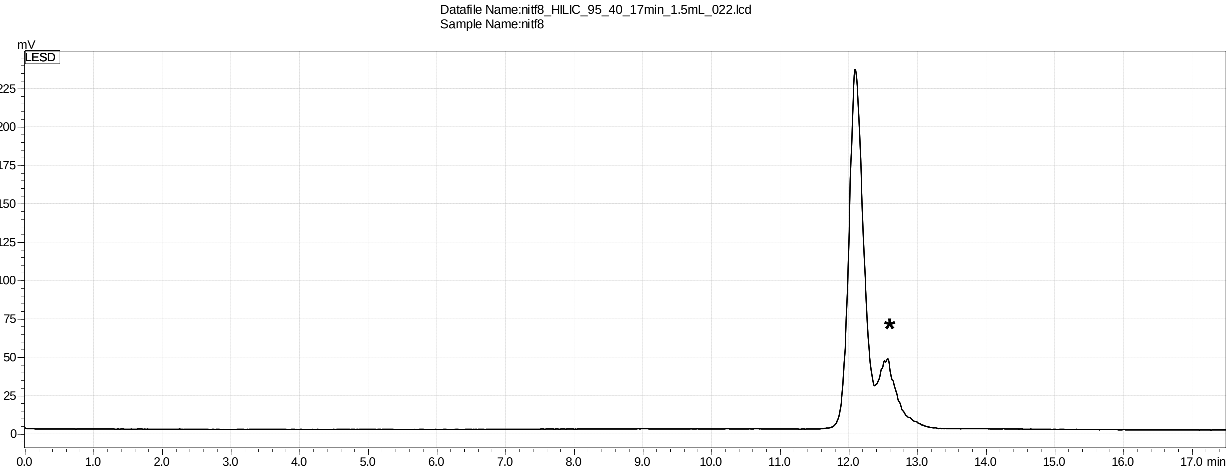

*Impurity from the ZIC-HILIC column

^{1}H, ^{13}C and HSQC-NMR of heptasaccharide **33**

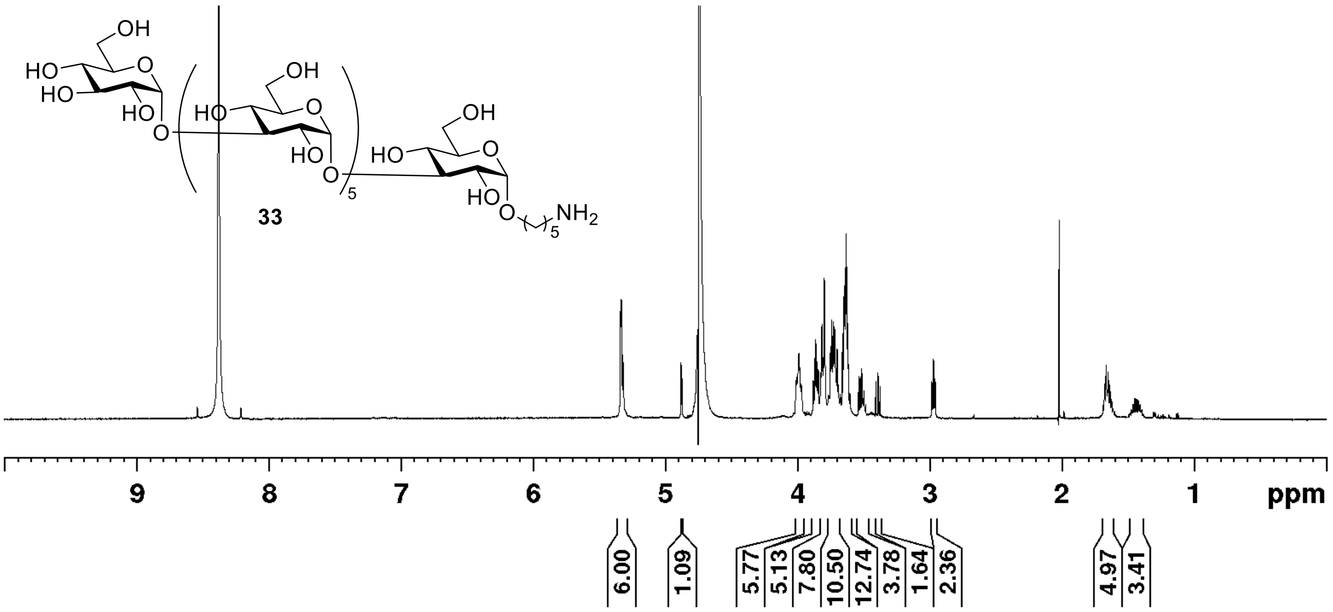

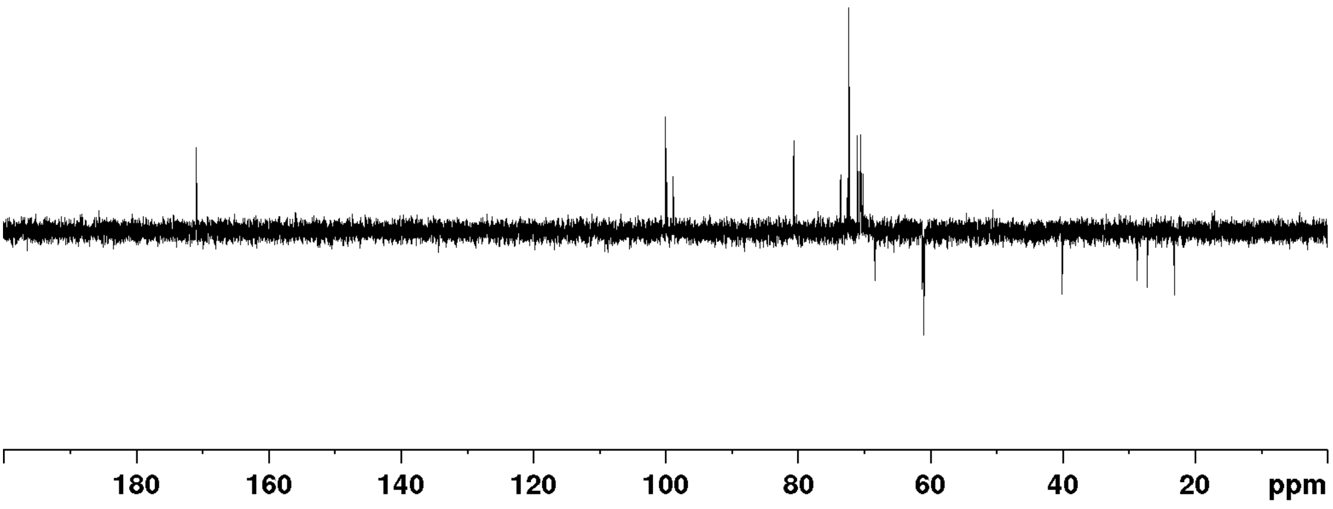

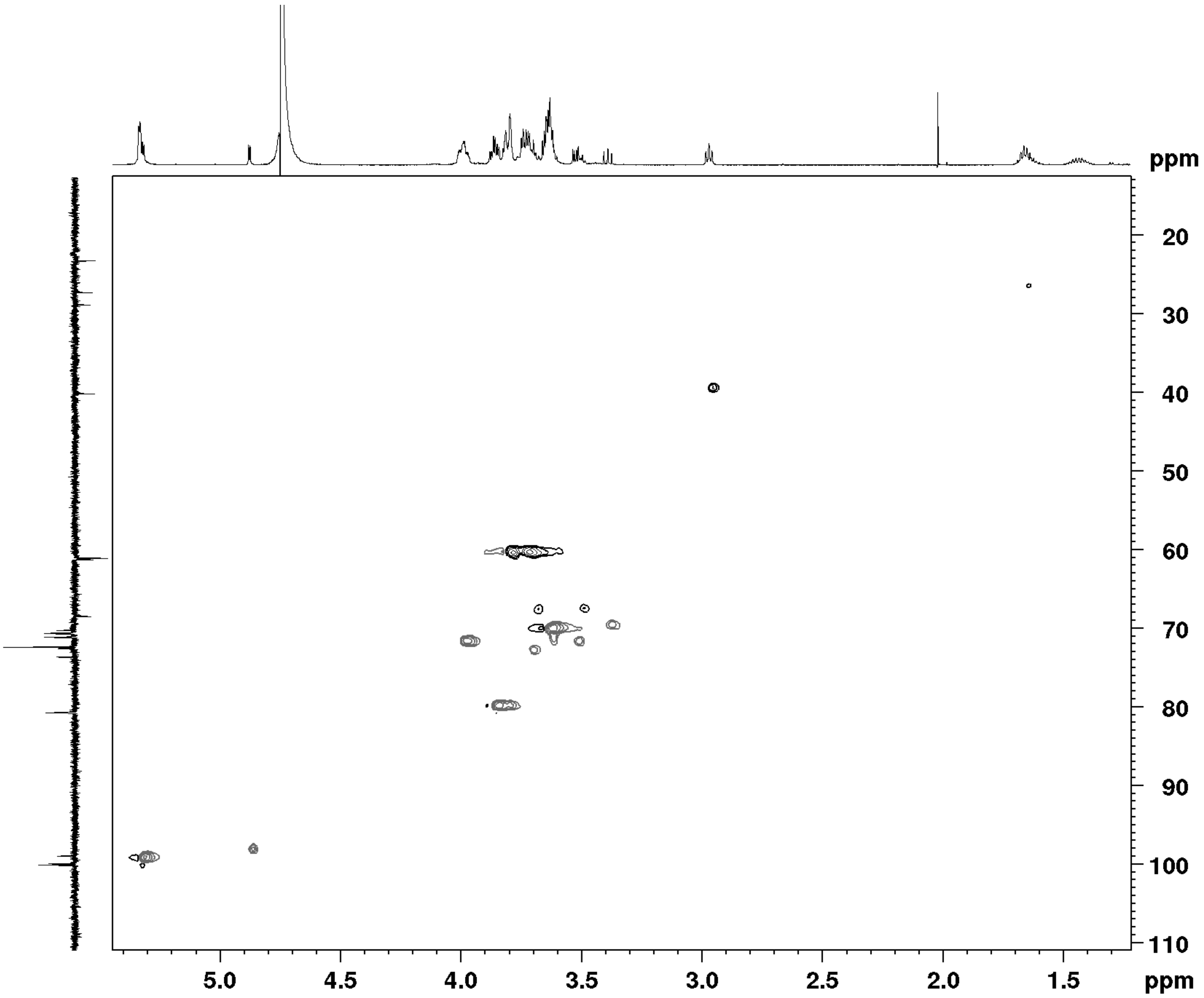

HPLC (ZIC-HILIC column MeCN/water/HCOOH = 95/5/0.1 → 40/60/0.1) of heptasaccharide **33**

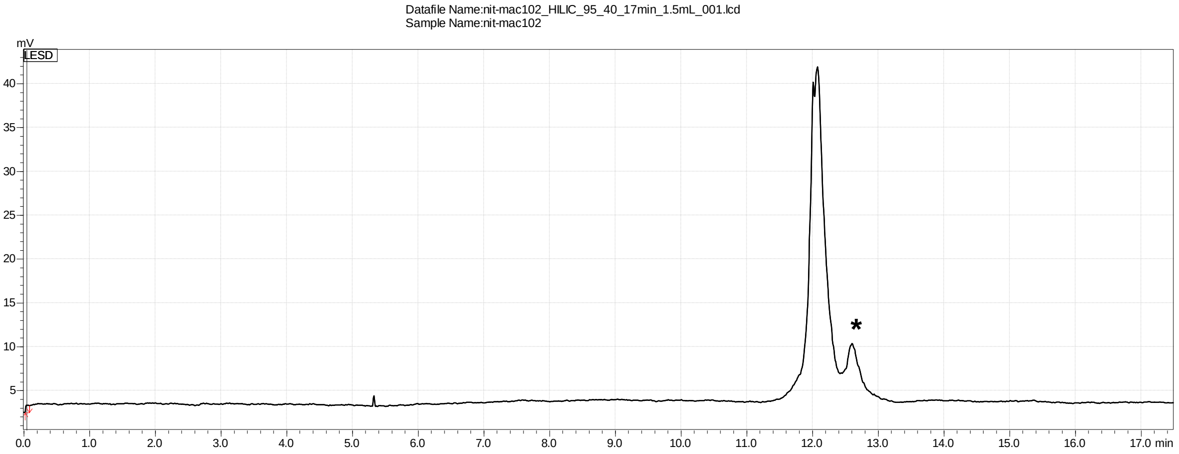

*Impurity from the ZIC-HILIC column

1.4 Analytical data of α-[1→4]-series

^{1}H and ^{13}C NMR of compound **36**

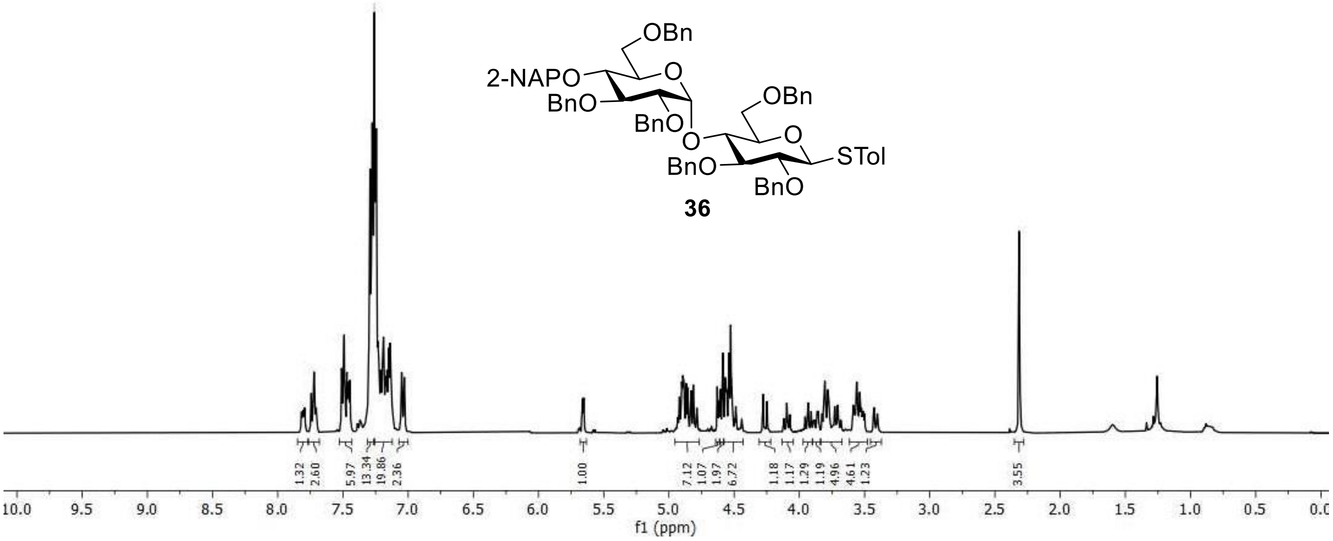

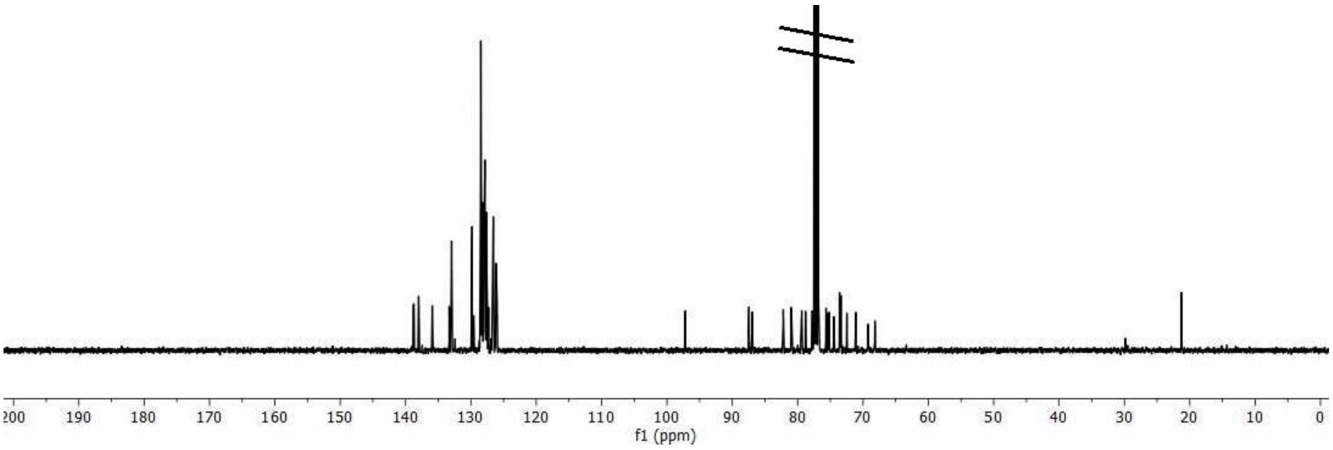

^{1}H and ^{13}C NMR of compound **37**

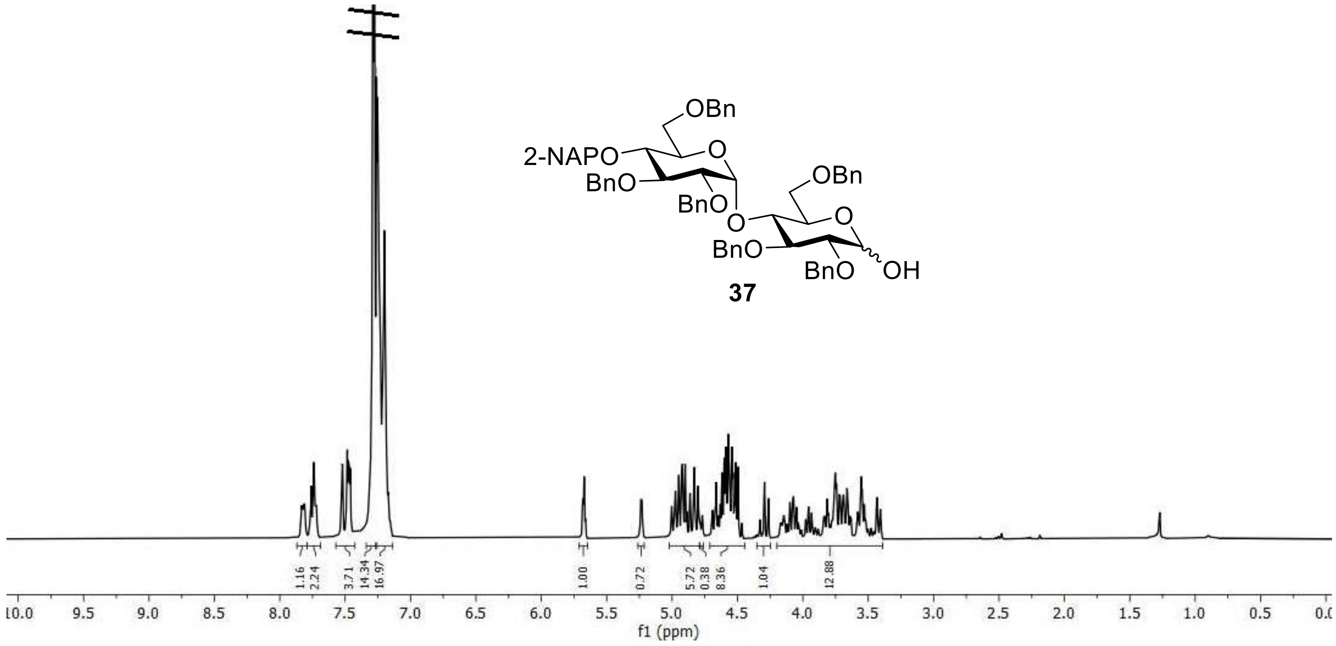

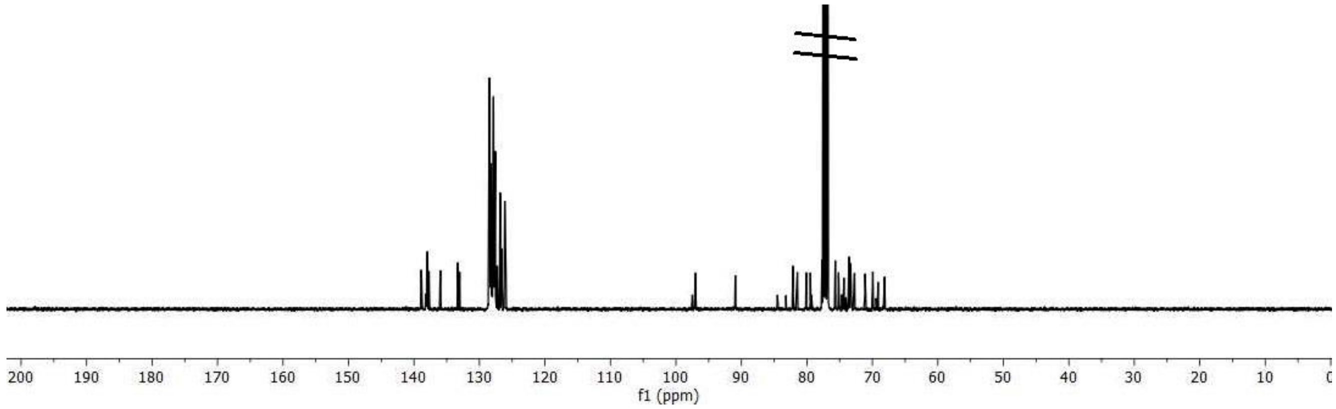

^{1}H and ^{13}C NMR of compound **38**

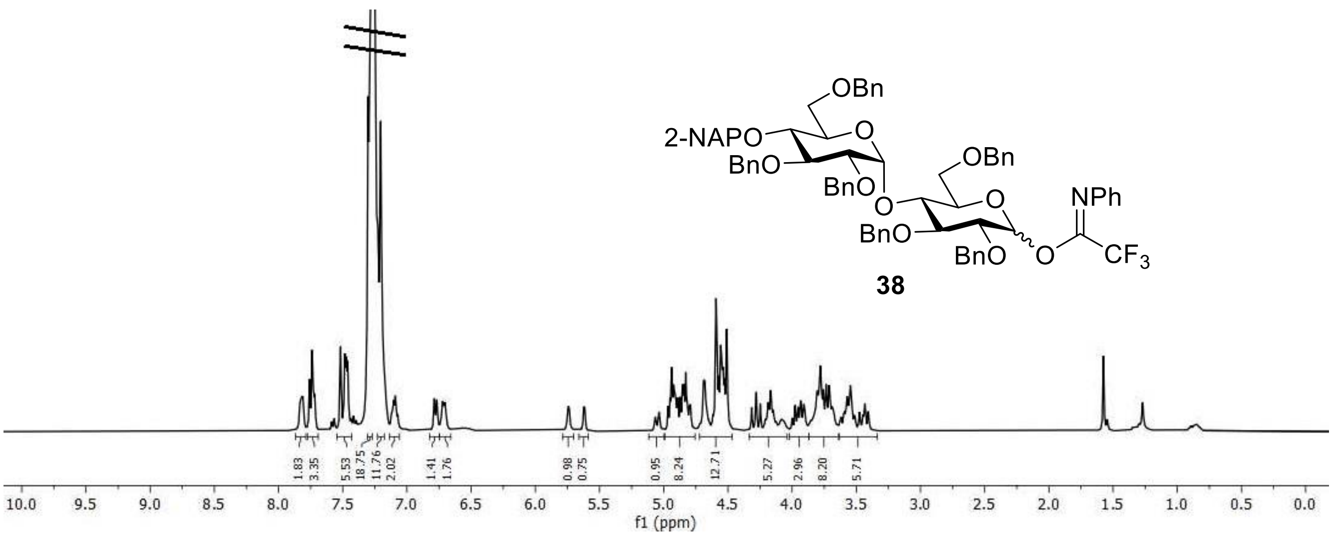

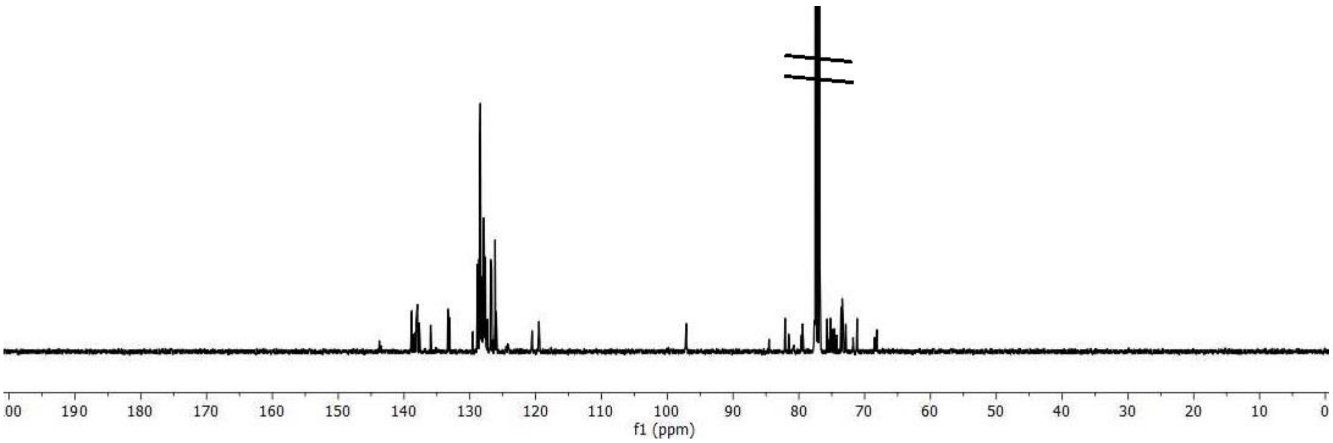

^{1}H and ^{13}C NMR of compound **39**

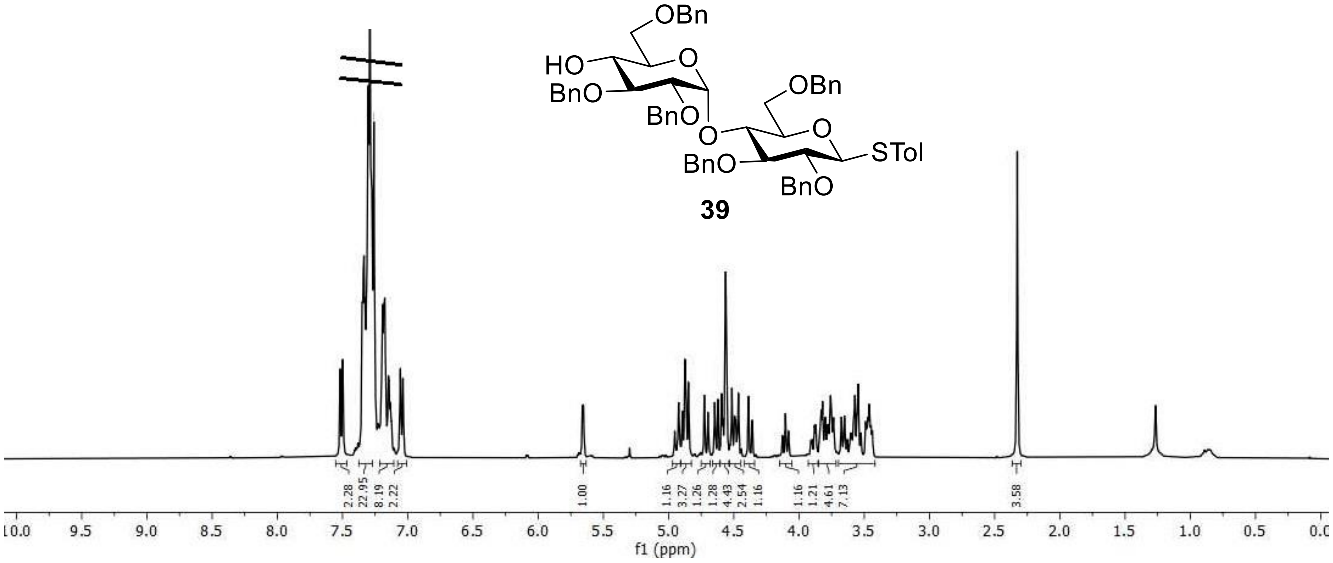

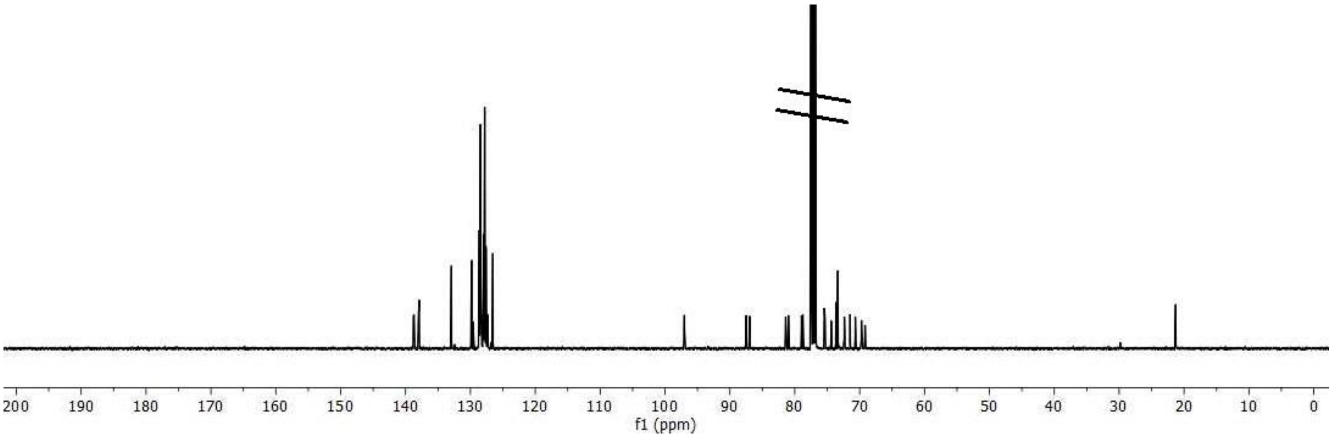

^{1}H and ^{13}C NMR of compound **40**

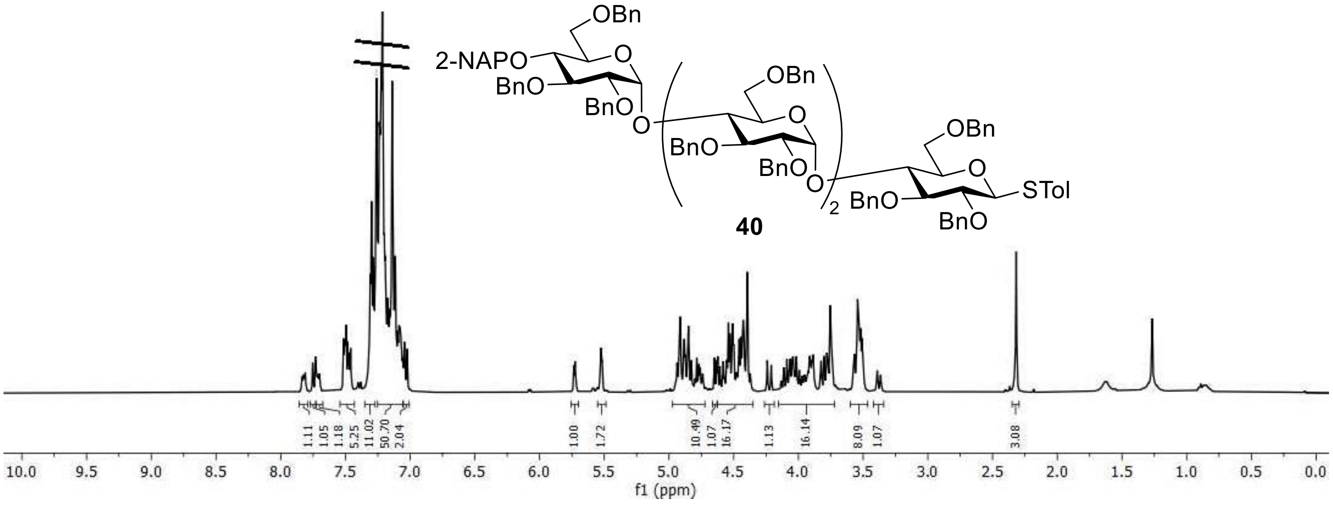

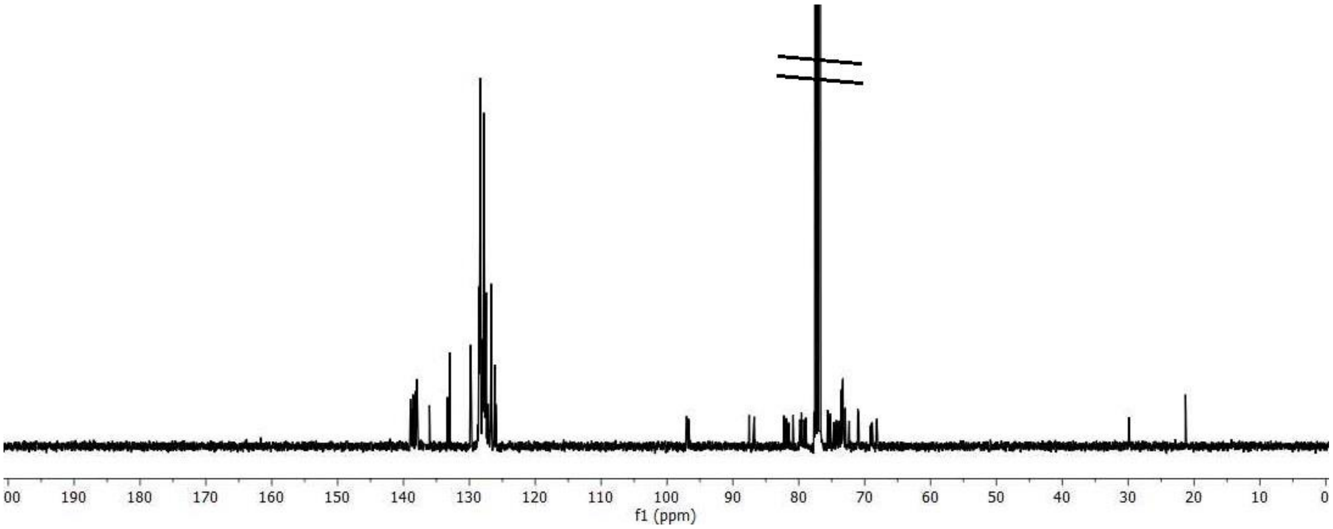

^{1}H and ^{13}C NMR of compound **41**

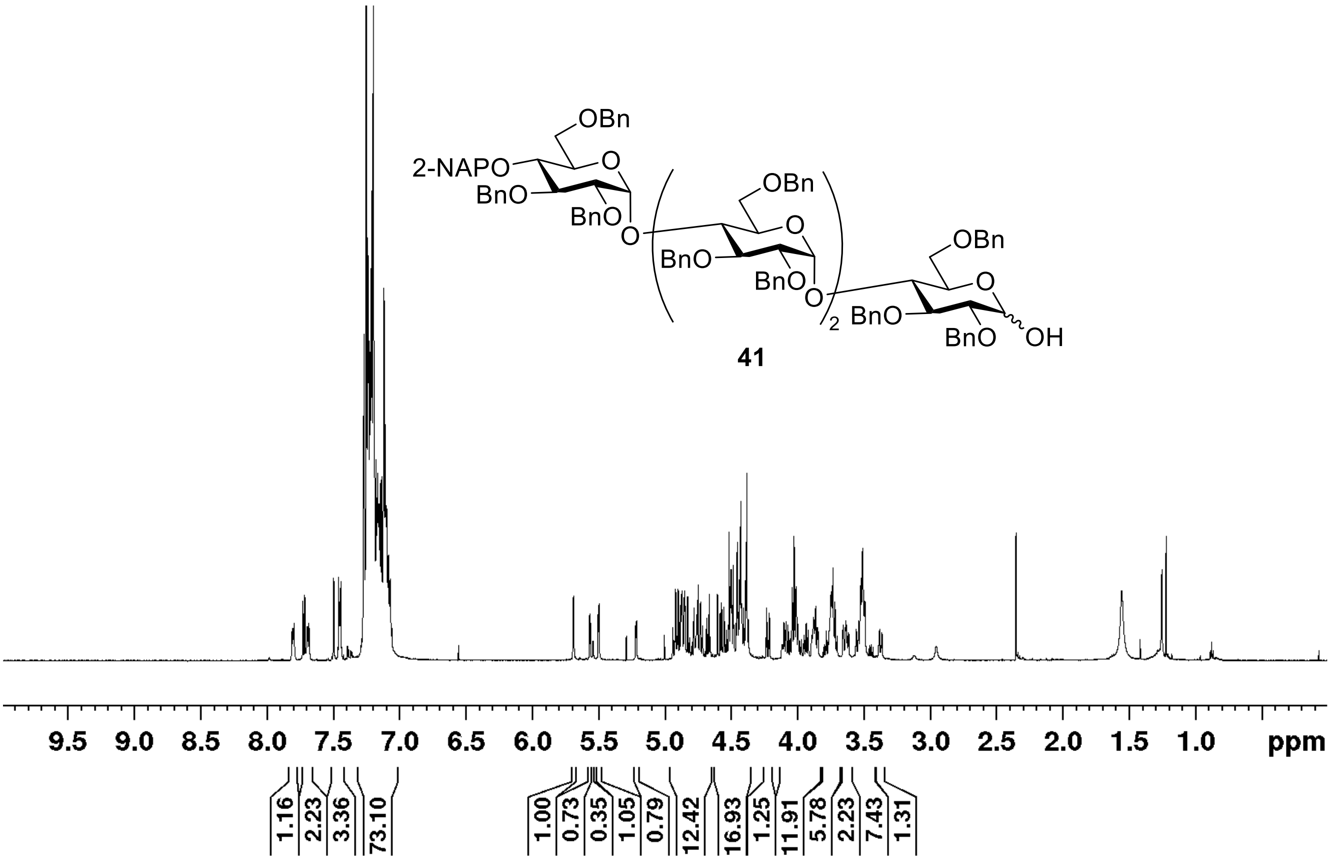

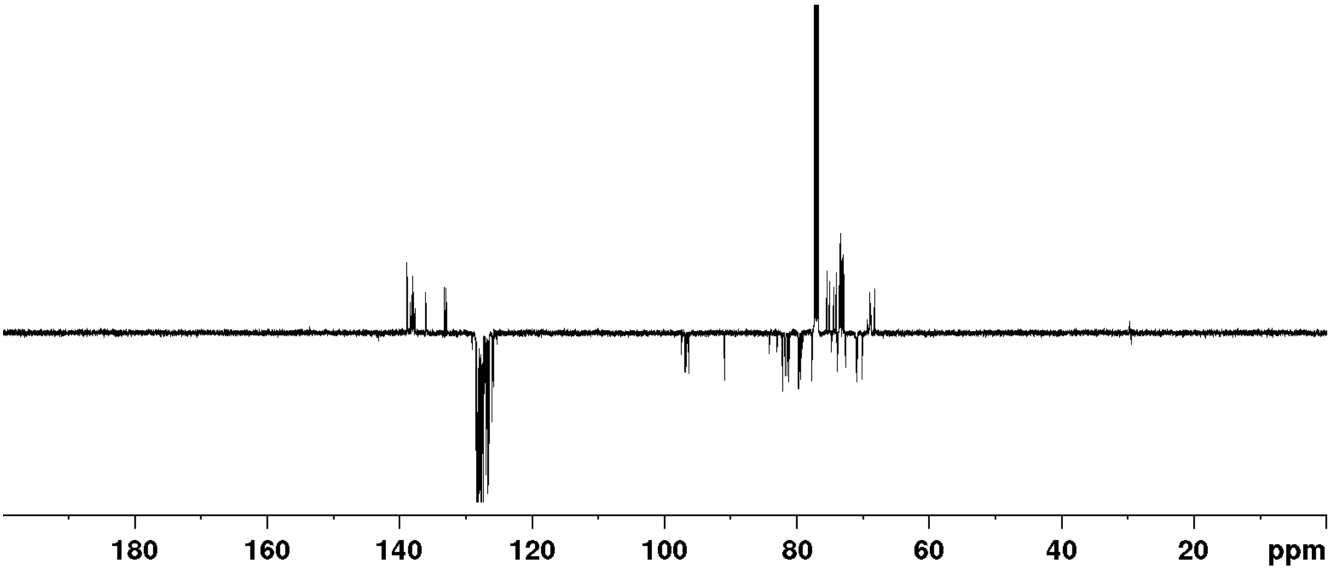

^{1}H and ^{13}C NMR of compound **42**

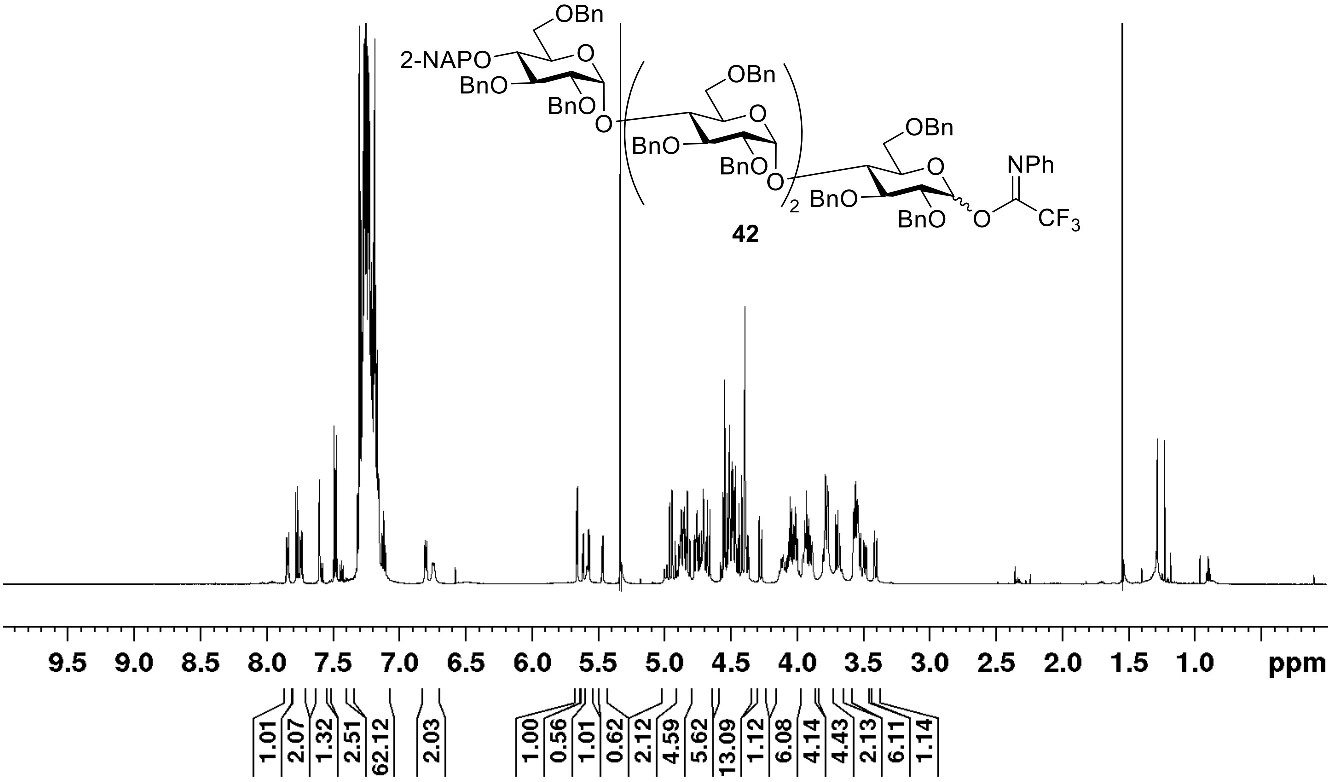

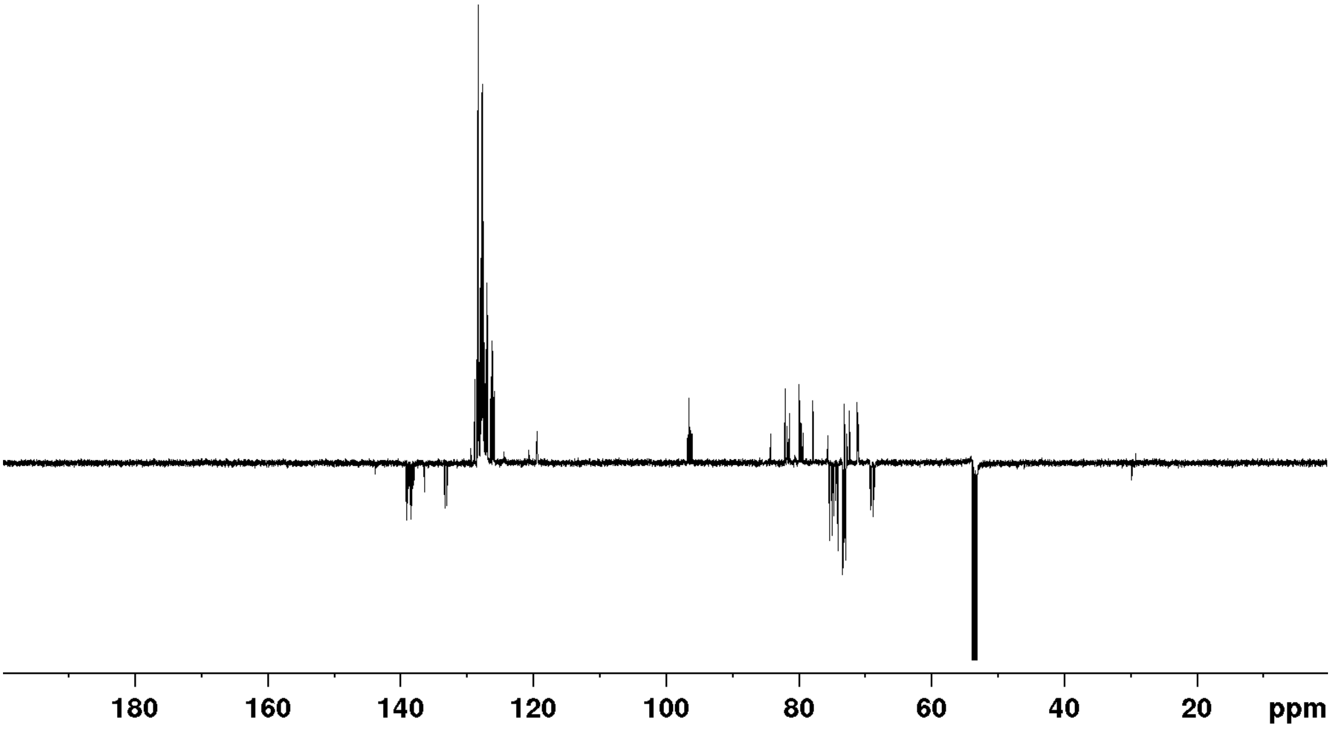

^{1}H and ^{13}C NMR of compound **43**

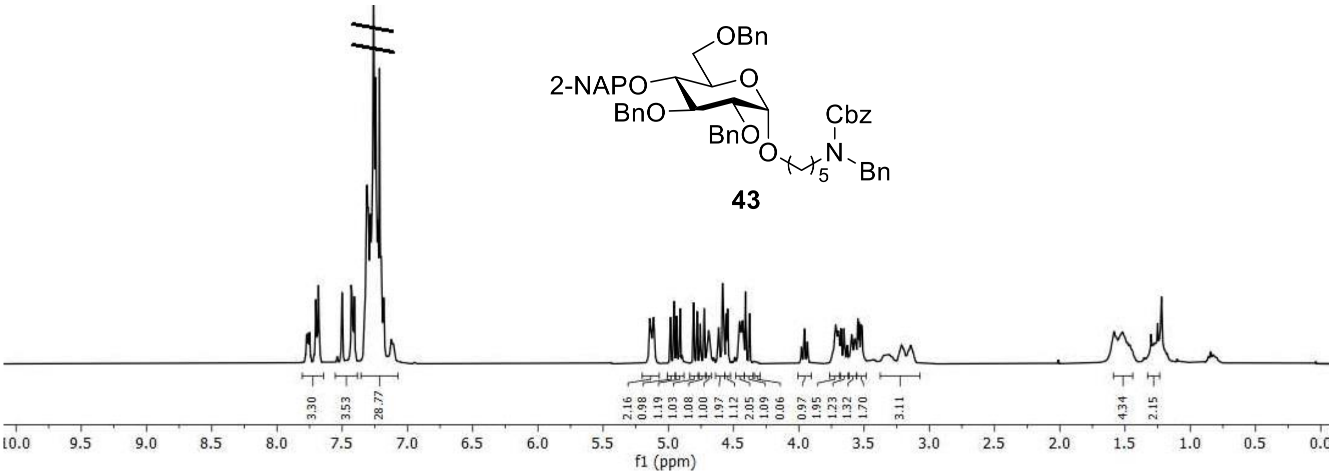

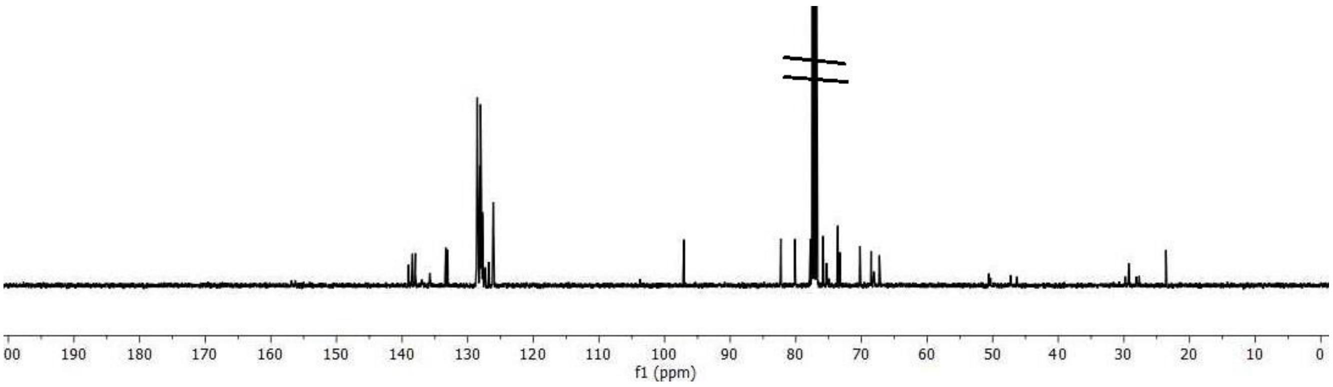

^{1}H and ^{13}C NMR of compound **44**

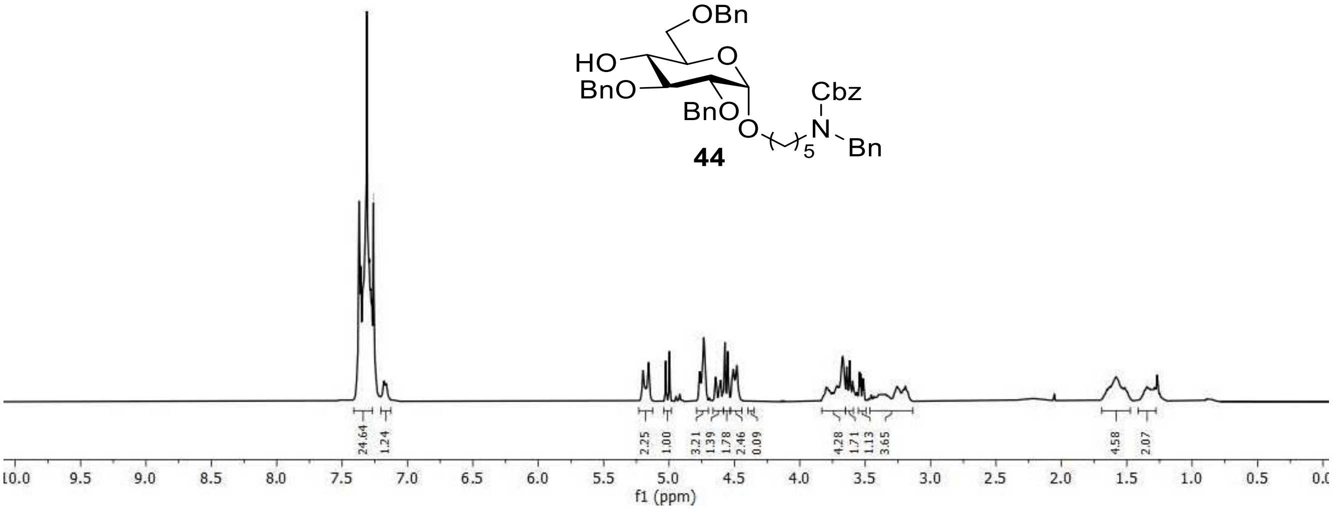

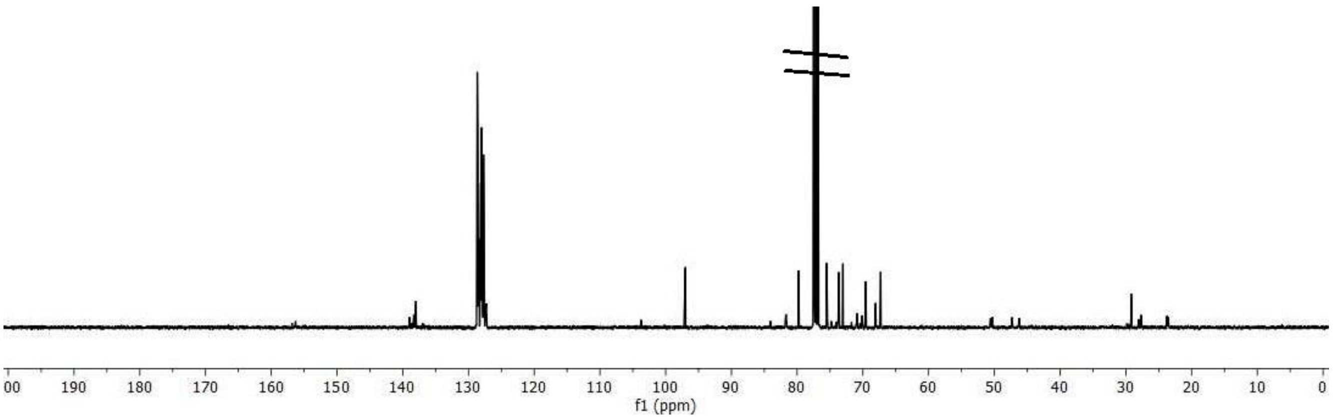

^{1}H and ^{13}C NMR of compound **45**

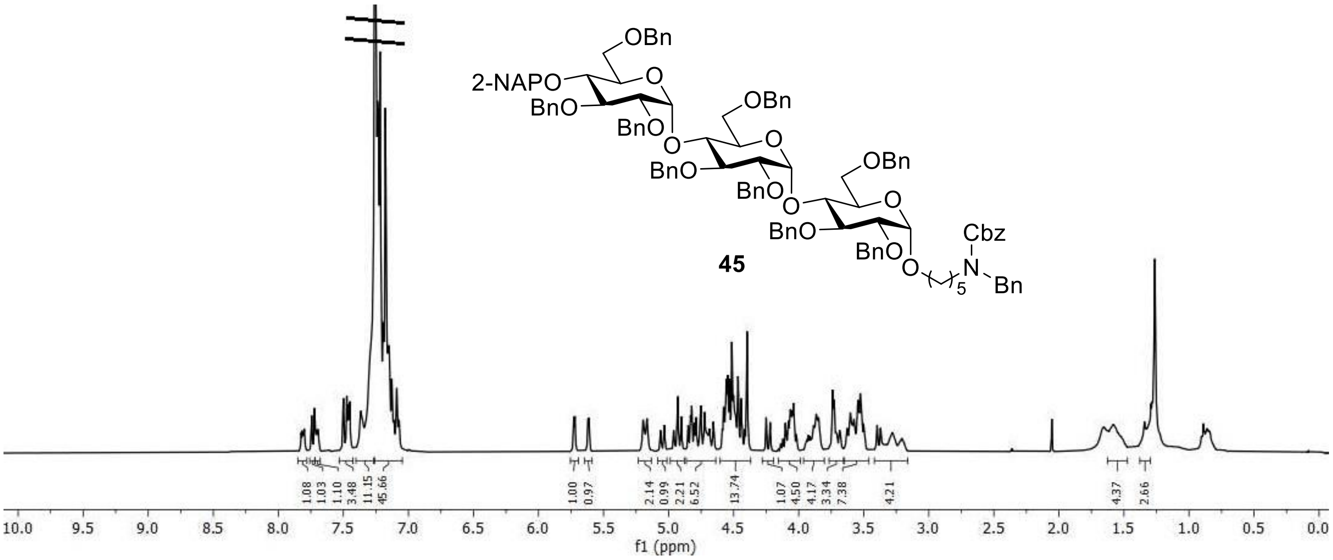

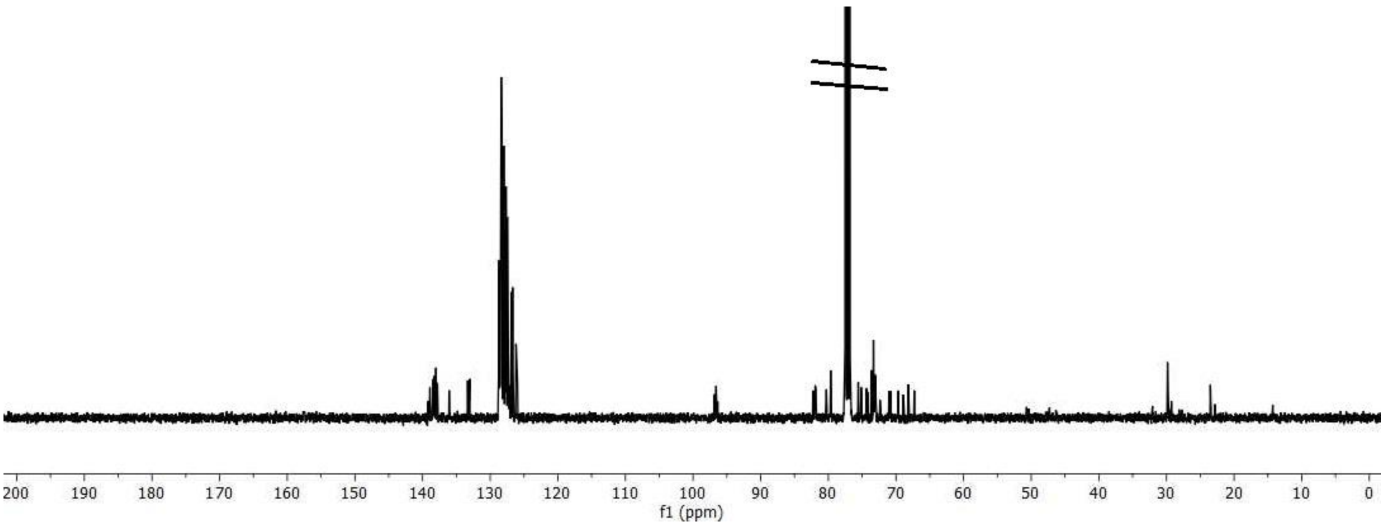

^{1}H and ^{13}C NMR of compound **46**

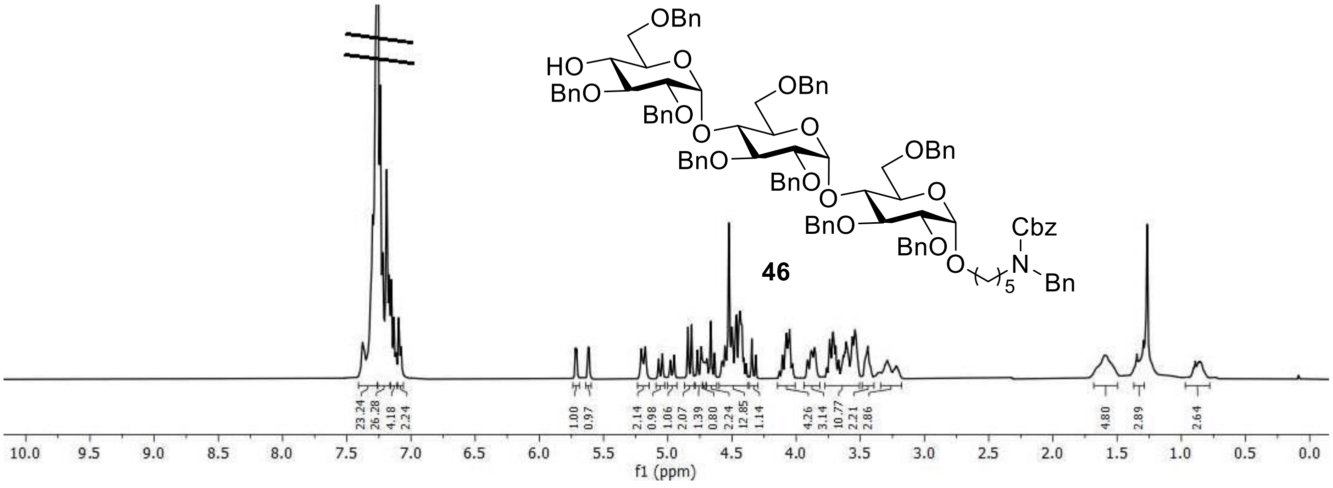

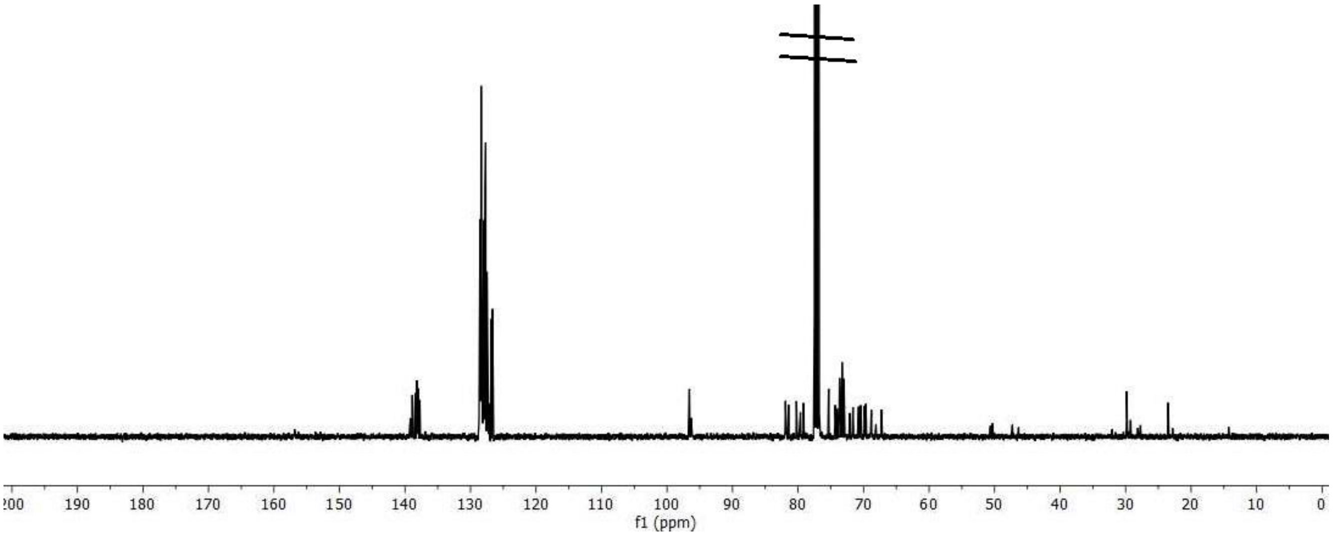

^{1}H and ^{13}C NMR of compound **47**

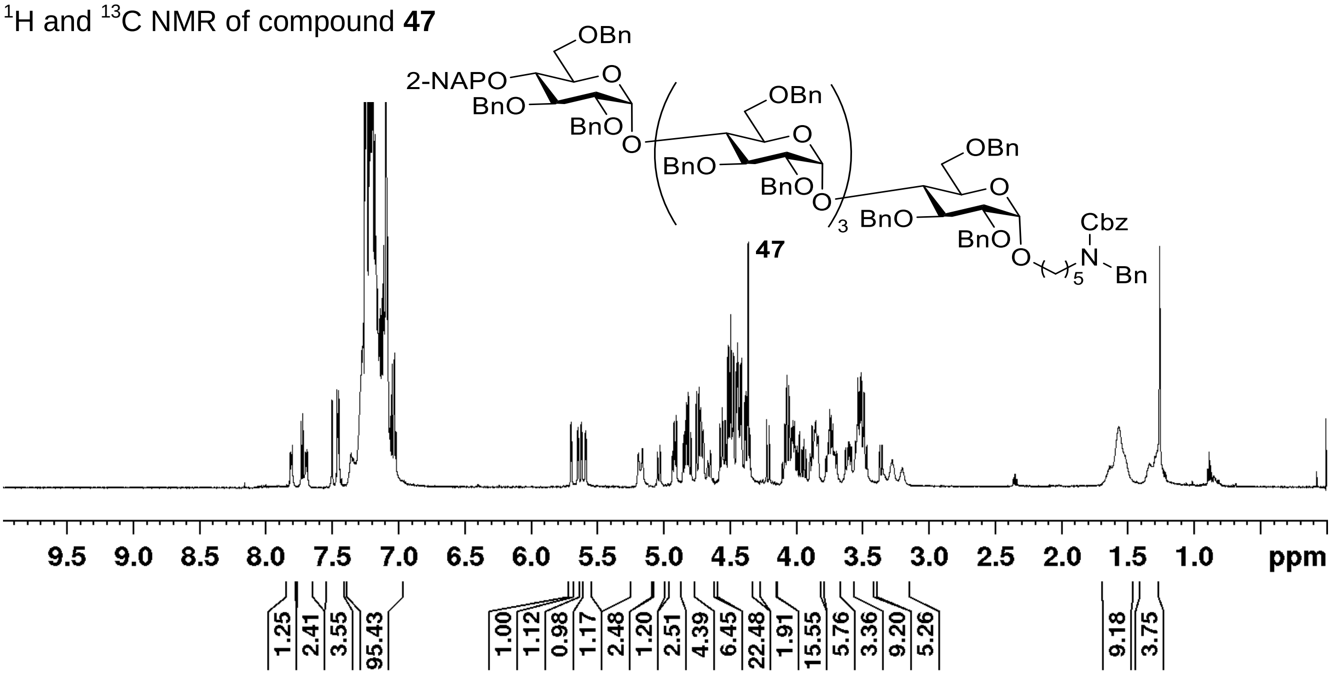

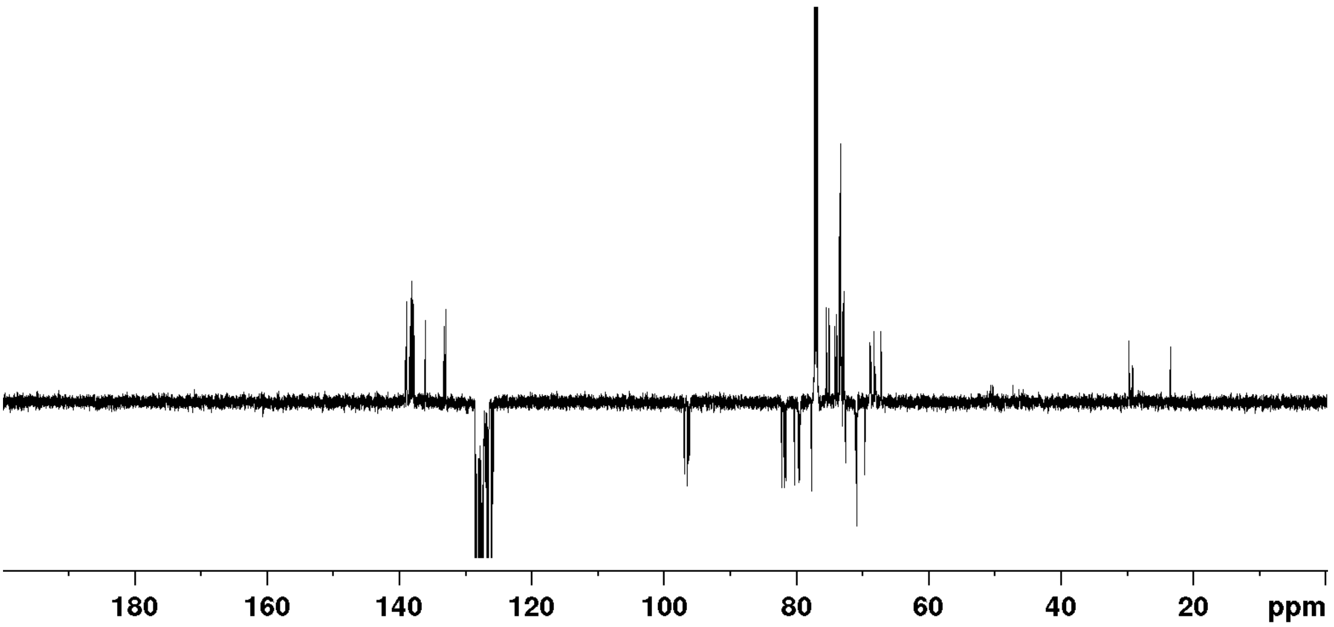

^{1}H and ^{13}C NMR of compound **48**

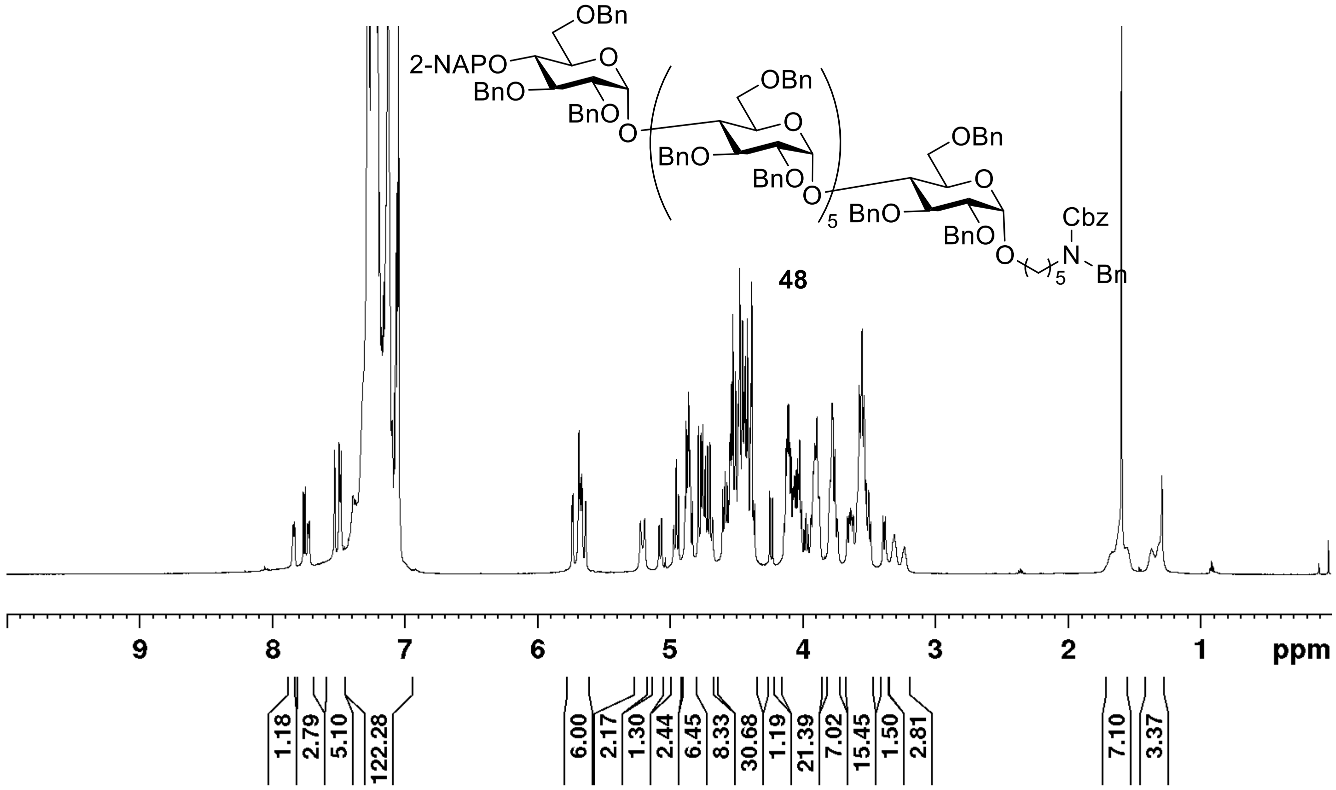

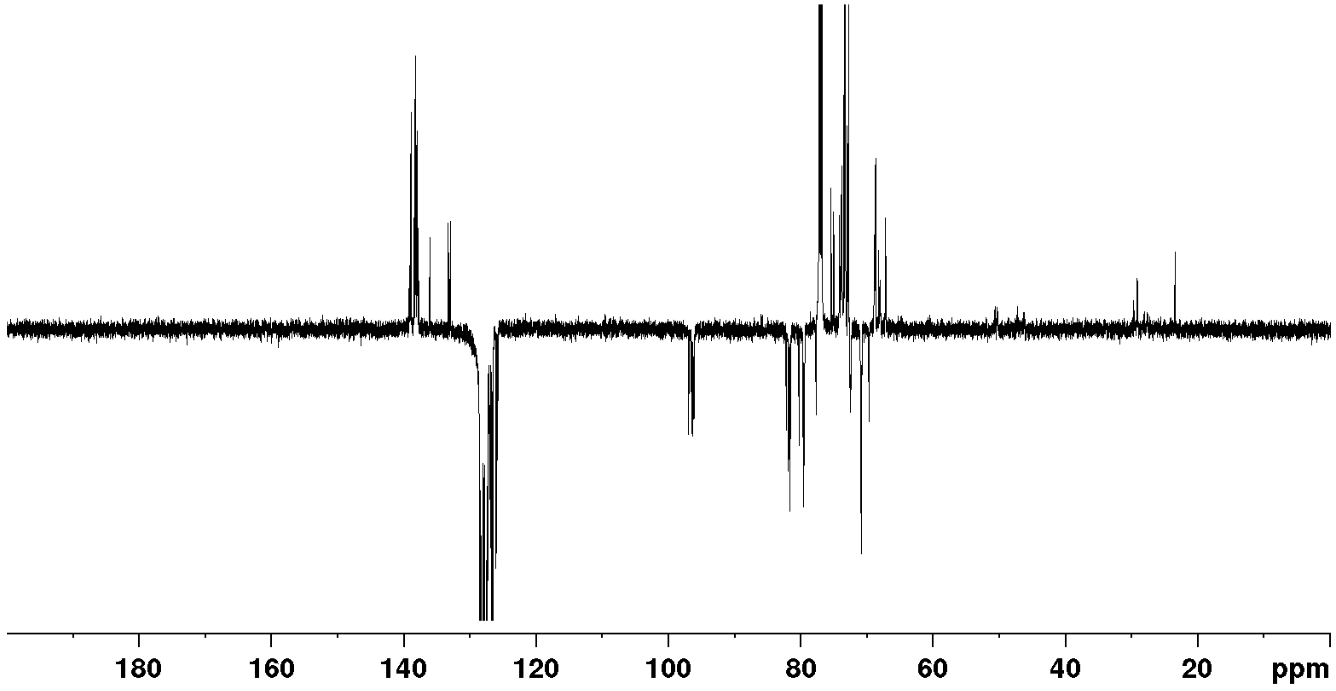

^{1}H, ^{13}C and HSQC-NMR of trisaccharide **49**

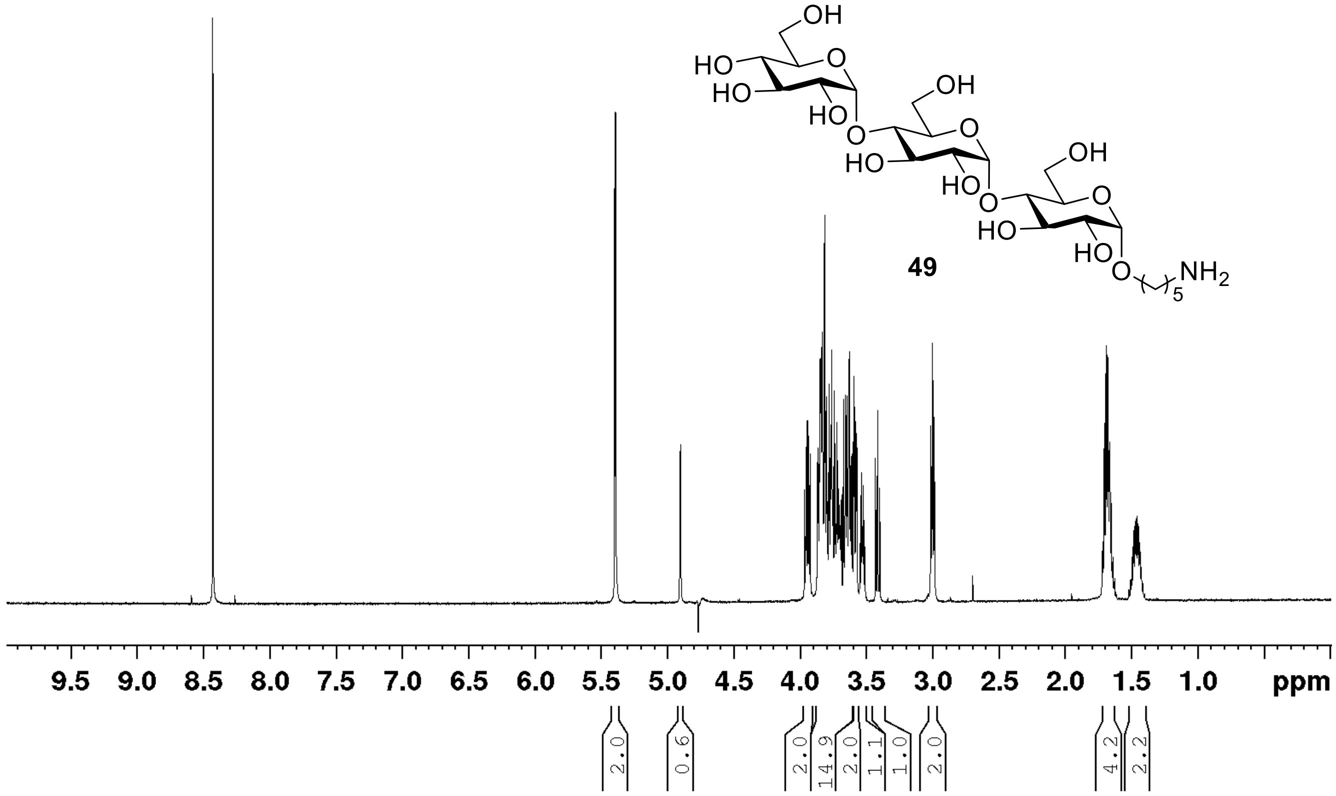

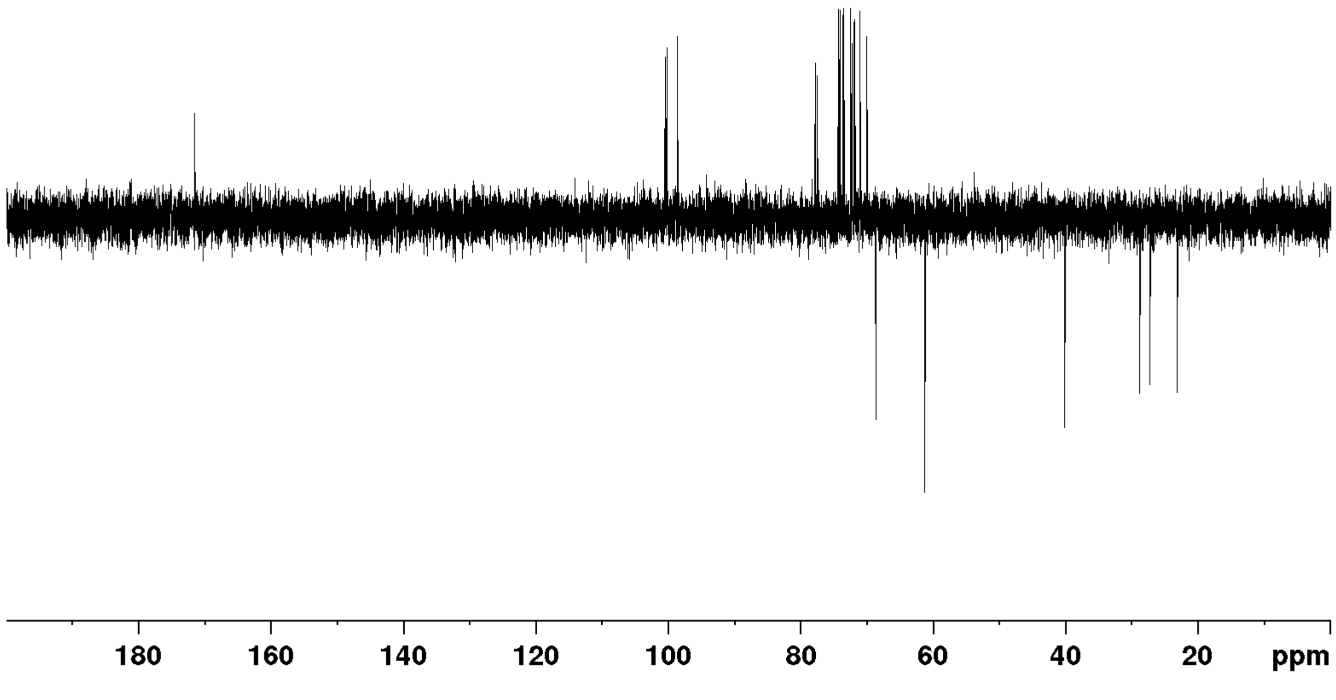

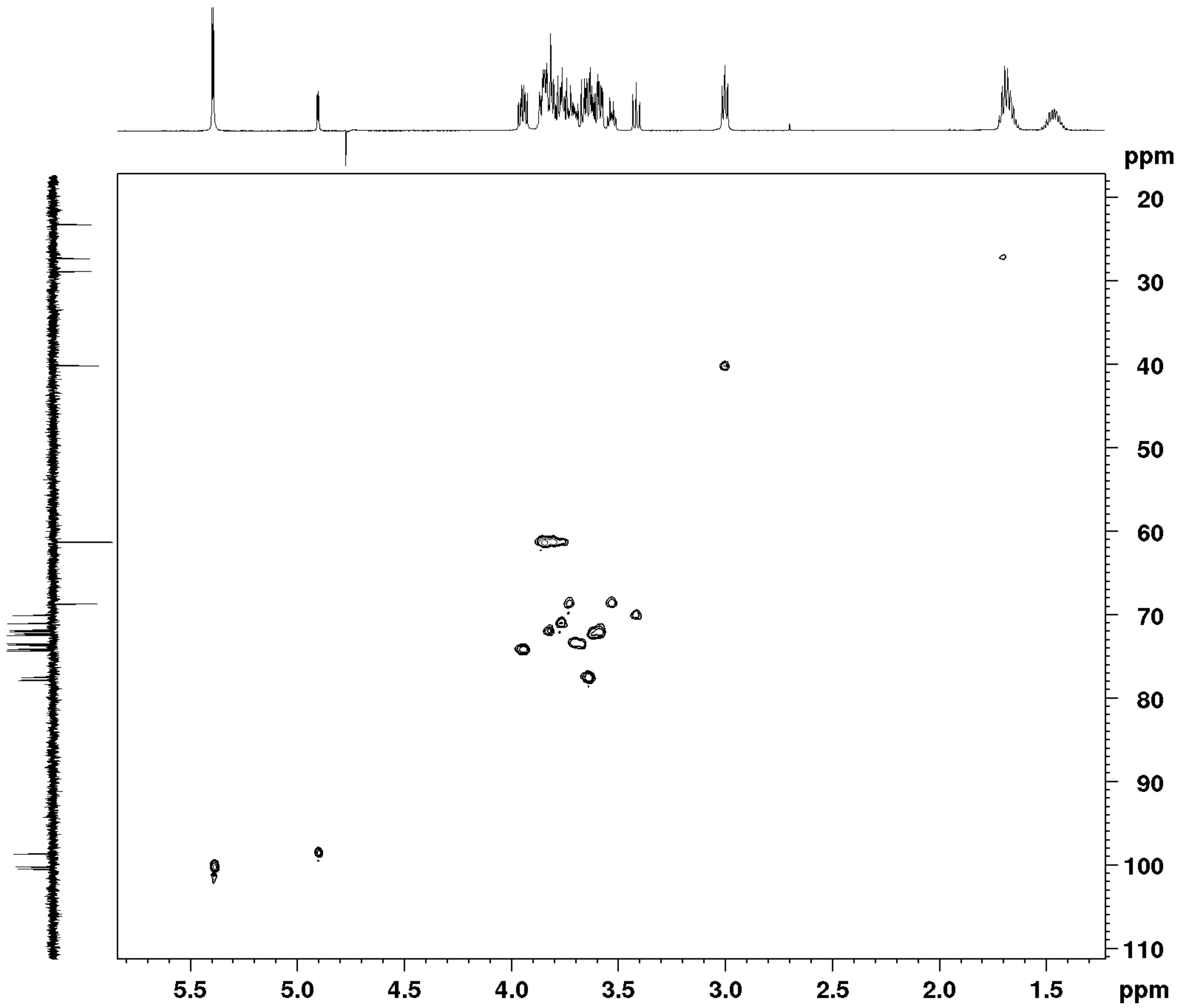

HPLC (ZIC-HILIC column MeCN/water/HCOOH = 95/5/0.1 → 40/60/0.1) of pure trisaccharide **49**

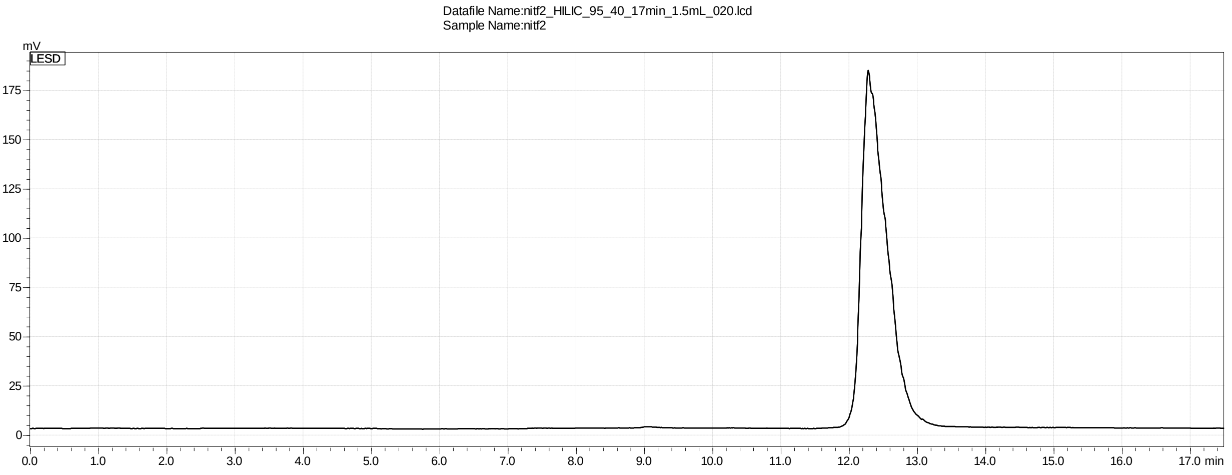

^{1}H, ^{13}C and HSQC-NMR of pentasaccharide **50**

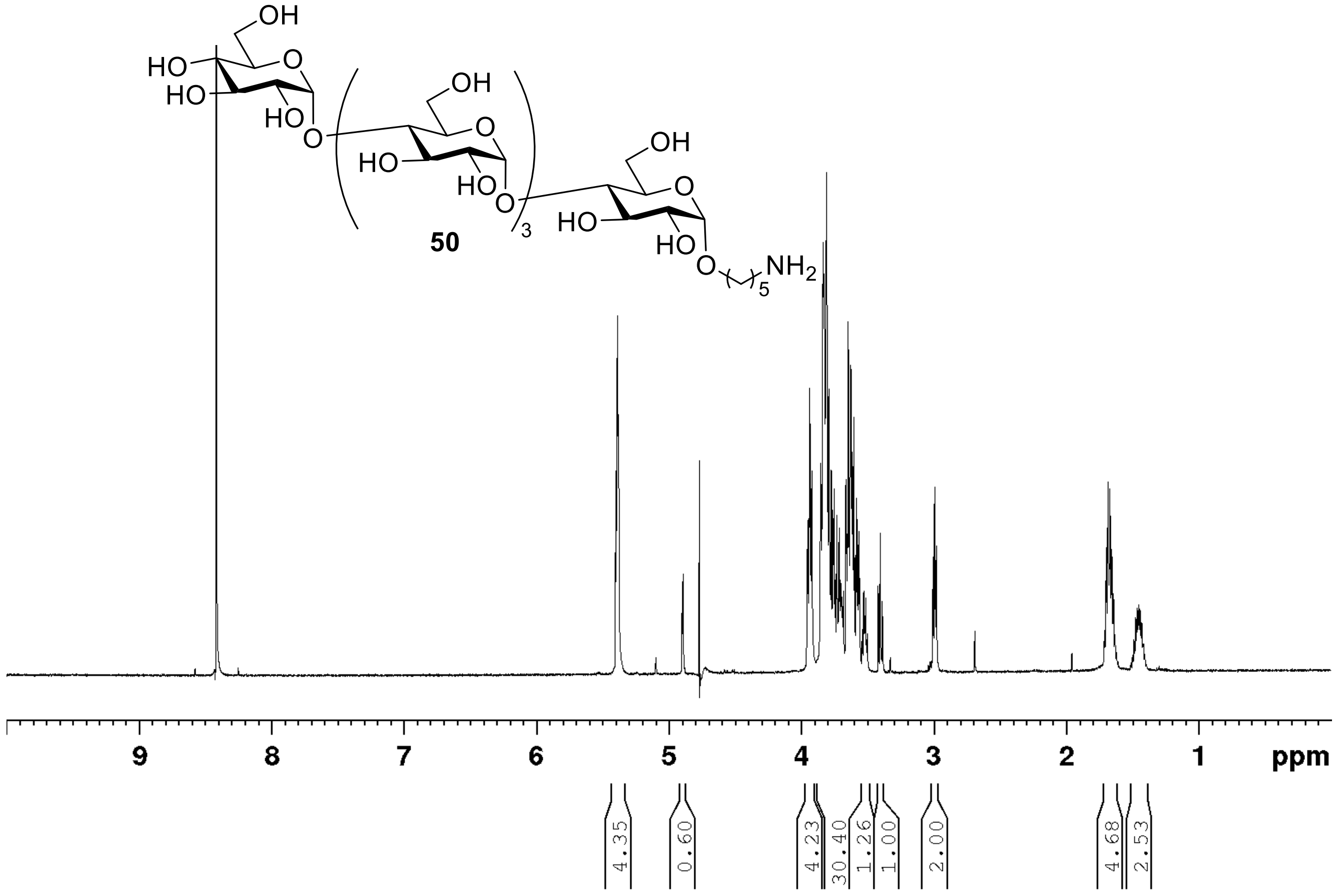

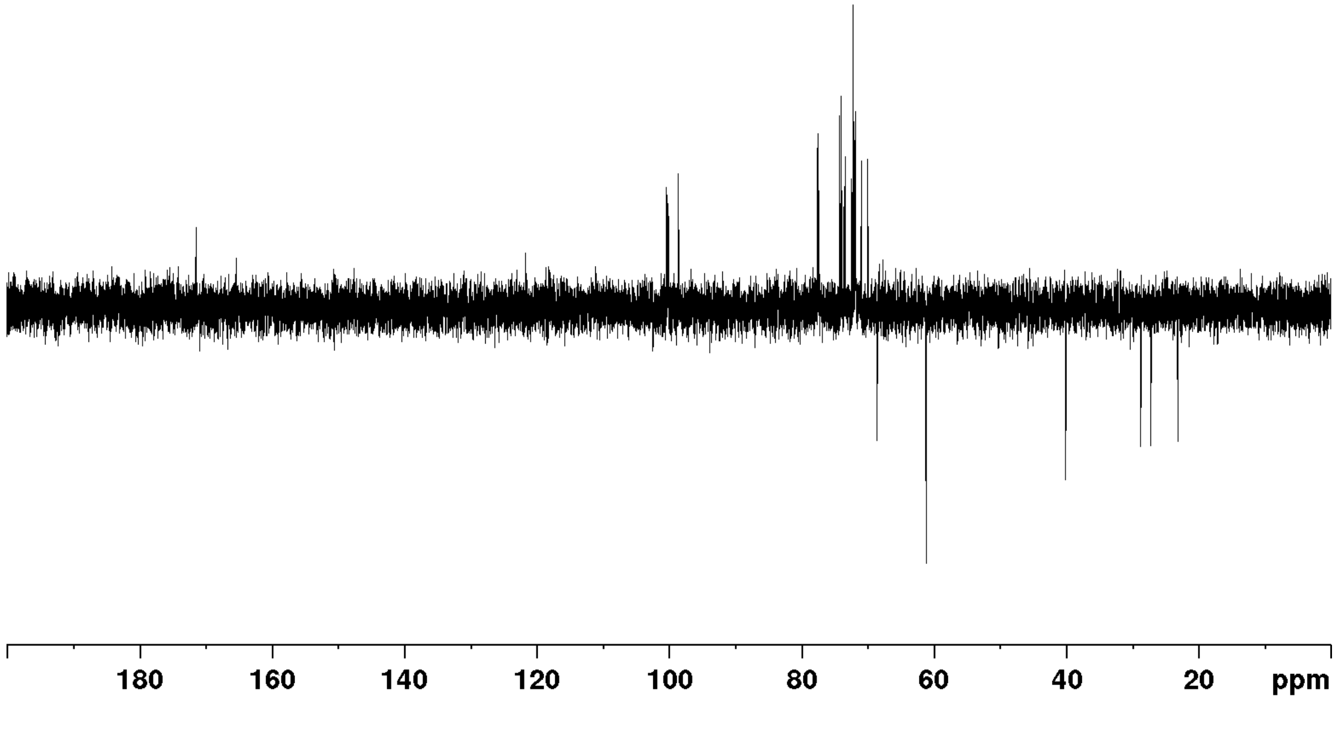

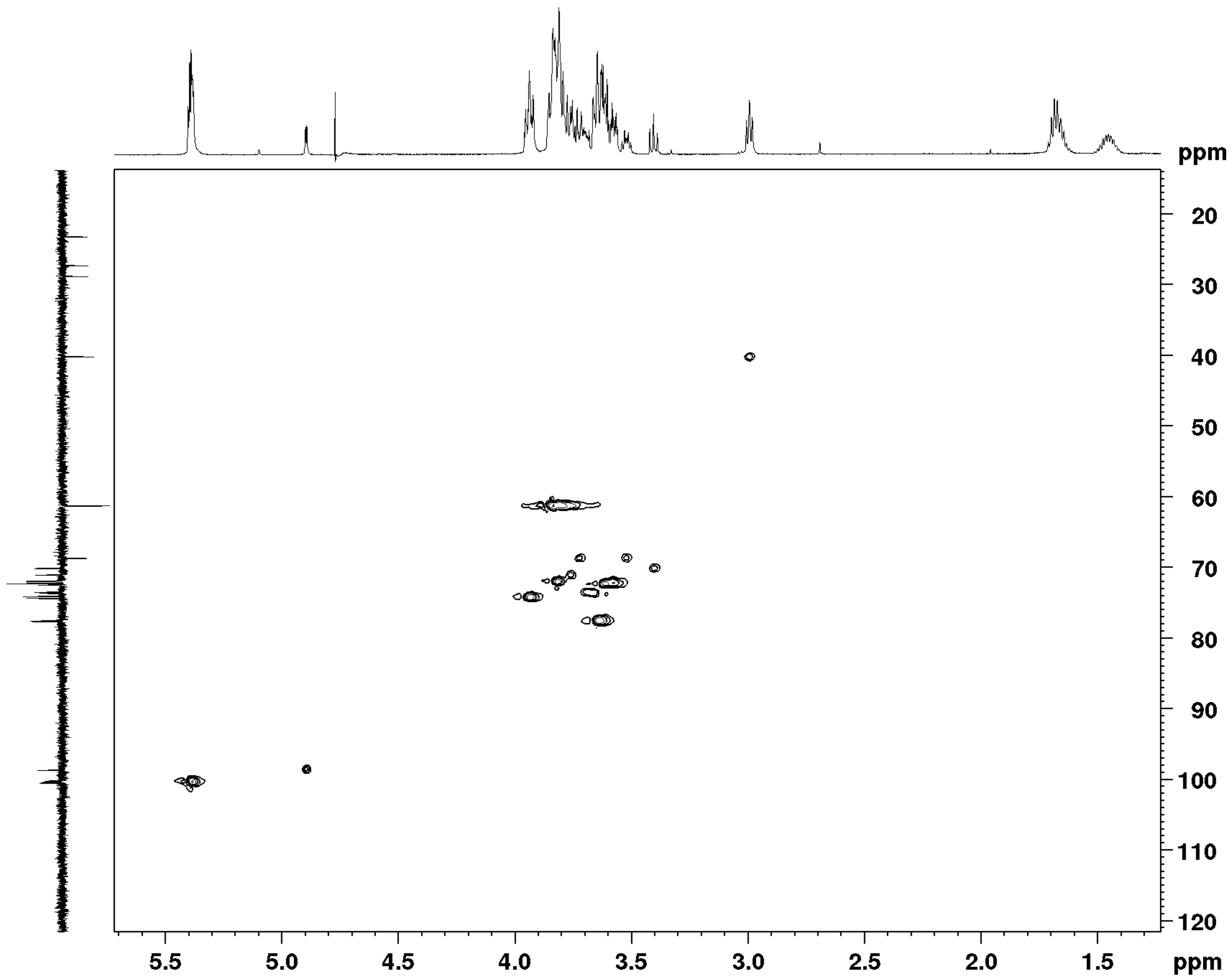

HPLC (ZIC-HILIC column MeCN/water/HCOOH = 95/5/0.1 → 40/60/0.1) of pentasaccharide **50**

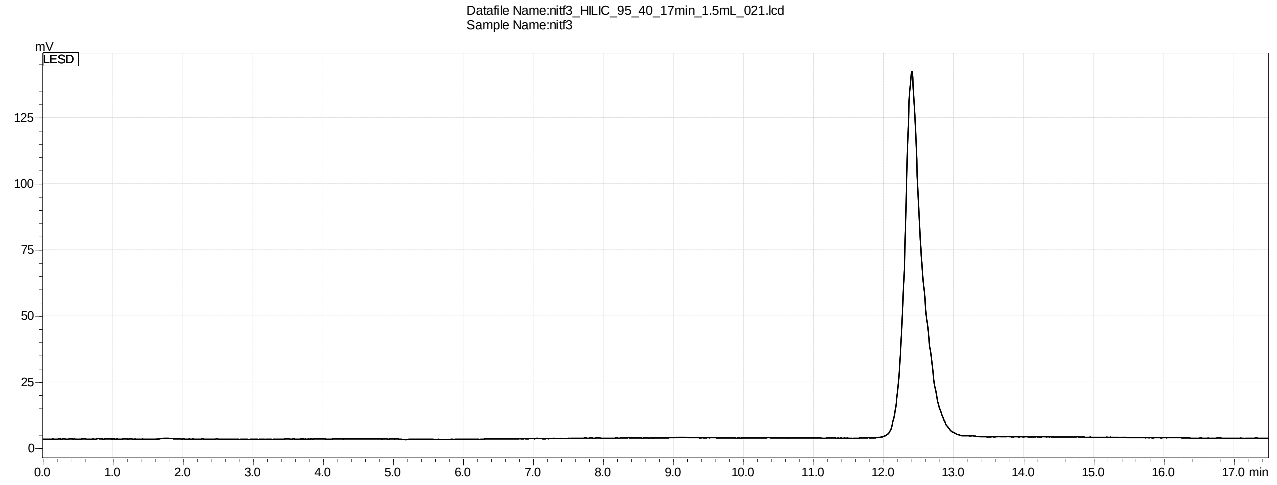

1H, ^{13}C and HSQC-NMR of heptasaccharide **51**

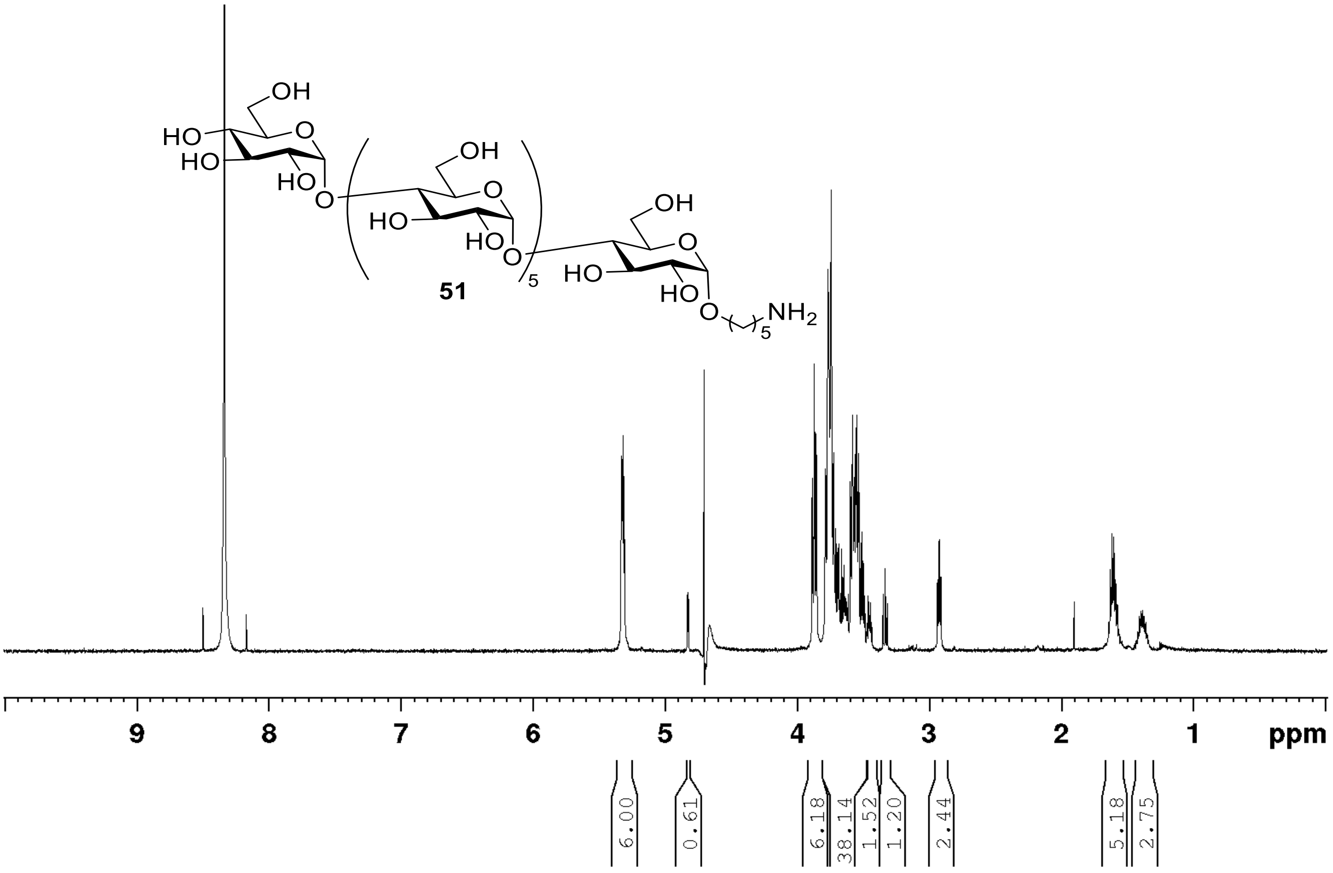

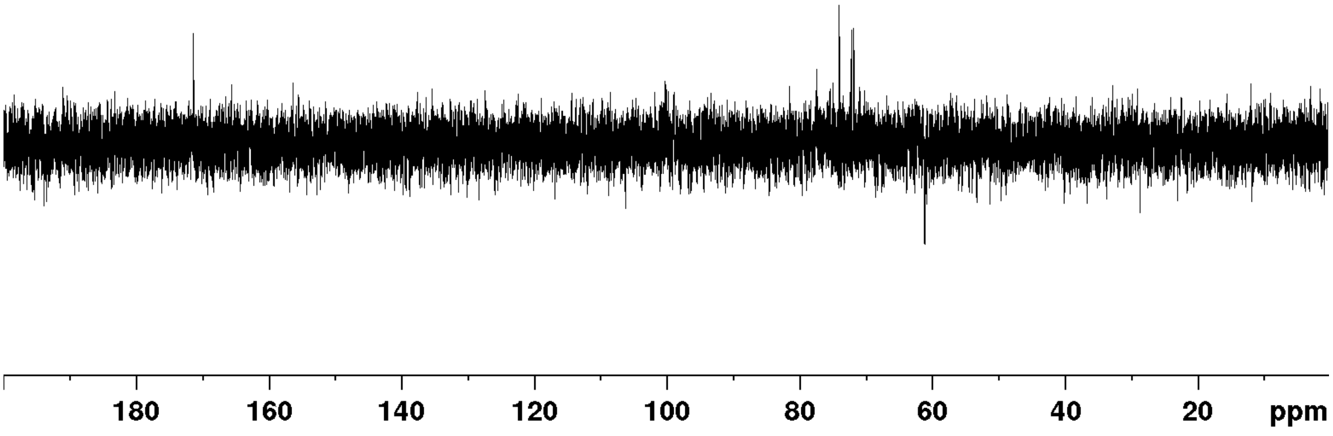

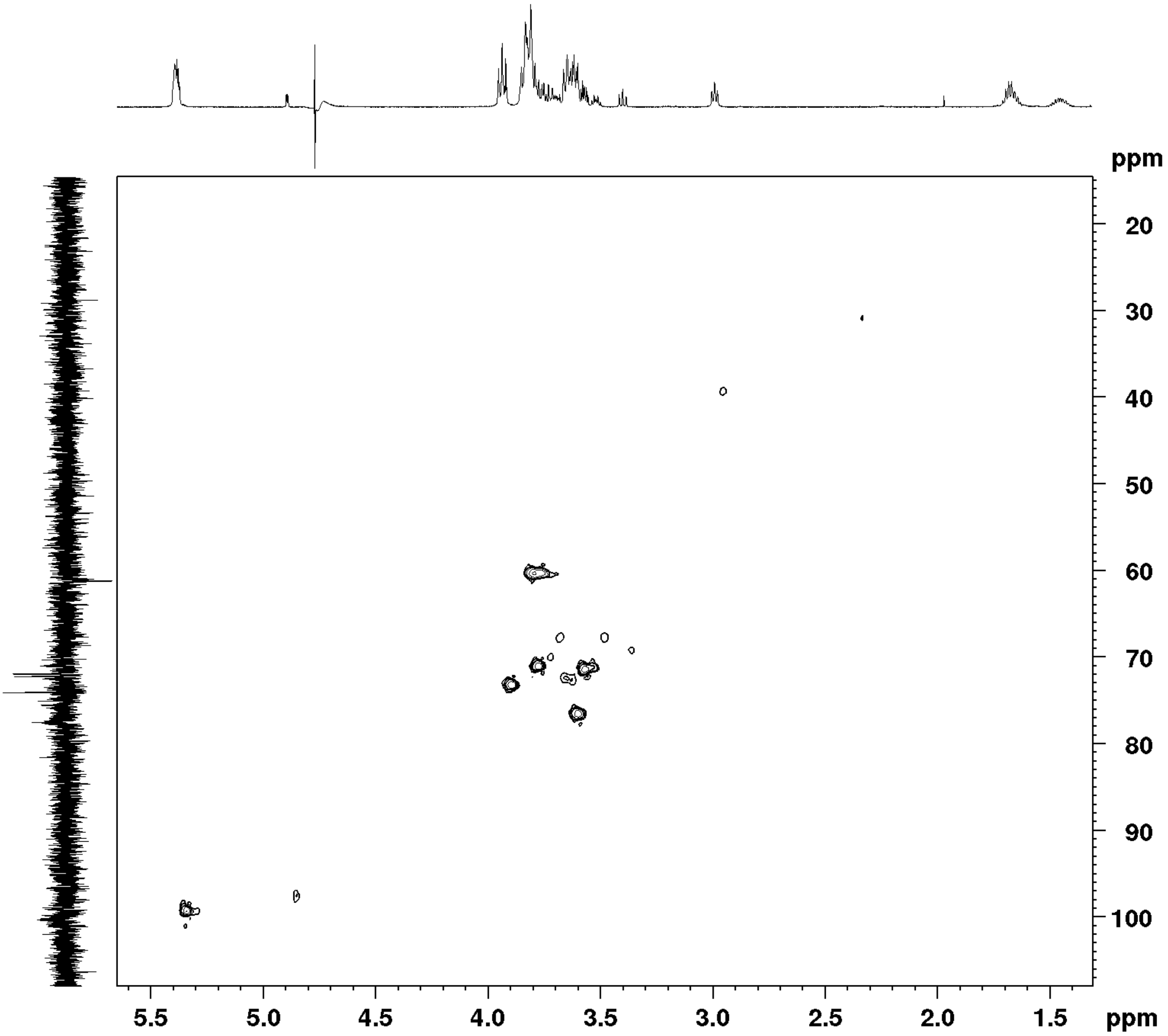

HPLC (ZIC-HILIC column MeCN/water/HCOOH = 95/5/0.1 → 40/60/0.1) of heptasaccharide **51**

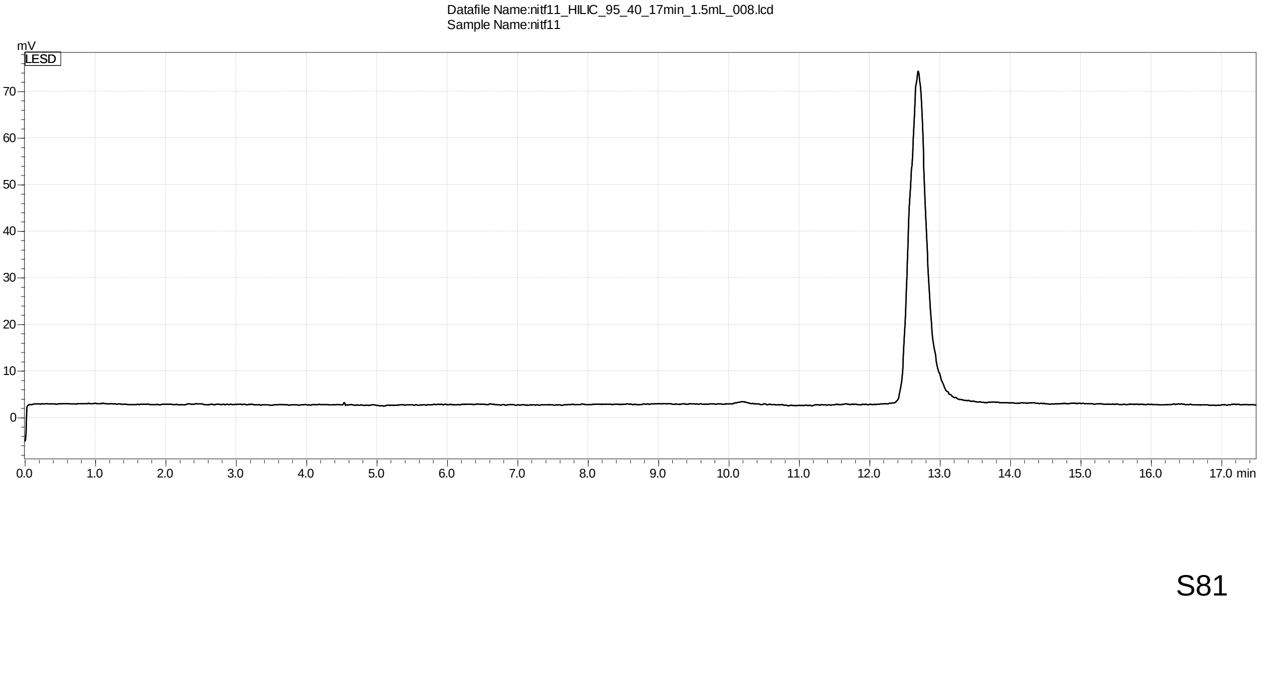

1.5 Biological replicate of MAPK phosphorylation assay

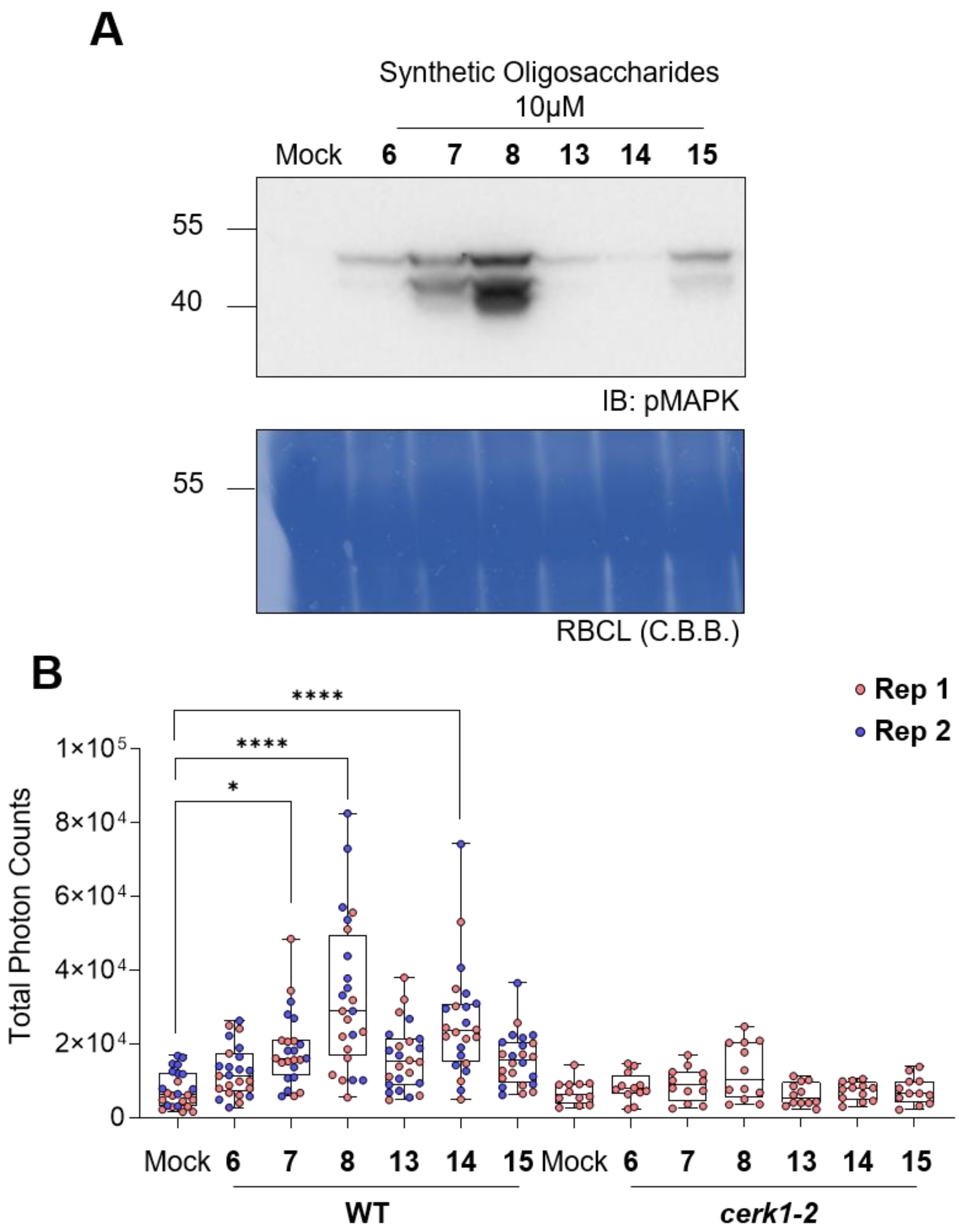

Figure 1. Synthetic oligosaccharides induce MAPK activation in plants. A. Biological replicate of immunoblotting with an anti-pMAPK antibody and Coomassie brilliant blue (C.B.B.) in Figure 1A. The activated MPK3/6 proteins were detected by immunoblotting and Rubisco large subunits (RBCLs) were stained by C.B.B. staining. B. Relative band intensities of pMAPK in Figure 1A and Figure S1A. The band intensities of pMAPK were normalized by the band intensities of RBCL (C.B.B.) and set the pMAPK/RBCL ratio of Mock as 1. Error bar indicate standard error.

1.6 References

[1] L. Kröck, D. Esposito, B. Castagner, C. C. Wang, P. Bindschädler, P. H. Seeberger, *Chem. Sci.* **2012**, *3*, 1617–1622.
